# Reducing haystacks to needles—ViralClust: a Nextflow pipeline to cluster viral sequences

**DOI:** 10.1093/gigascience/giag090

**Published:** 2026-09-14

**Authors:** Sandra Triebel, Kevin Lamkiewicz, Tom Eulenfeld, Manja Marz

**Affiliations:** RNA Bioinformatics & High-Throughput Analysis Group, Institute of Computer Science, Friedrich Schiller University Jena, Inselplatz 5, 07743 Jena, Germany; European Virus Bioinformatics Center, Friedrich Schiller University Jena, Inselplatz 5, 07743 Jena, Germany; RNA Bioinformatics & High-Throughput Analysis Group, Institute of Computer Science, Friedrich Schiller University Jena, Inselplatz 5, 07743 Jena, Germany; European Virus Bioinformatics Center, Friedrich Schiller University Jena, Inselplatz 5, 07743 Jena, Germany; RNA Bioinformatics & High-Throughput Analysis Group, Institute of Computer Science, Friedrich Schiller University Jena, Inselplatz 5, 07743 Jena, Germany; RNA Bioinformatics & High-Throughput Analysis Group, Institute of Computer Science, Friedrich Schiller University Jena, Inselplatz 5, 07743 Jena, Germany; European Virus Bioinformatics Center, Friedrich Schiller University Jena, Inselplatz 5, 07743 Jena, Germany

**Keywords:** virus, clustering, Nextflow, bioinformatics, viral evolution

## Abstract

**Background:**

The rapid accumulation of viral genome sequences presents major challenges for downstream analysis tools, including tools for multiple sequence alignments, phylogeny, and genome/alignment visualization, due to computational constraints and sampling biases caused by outbreak-driven over-representation. Selecting representative genomes through clustering offers a principled alternative to random subsampling, yet choosing appropriate clustering strategies remains non-trivial and context-dependent.

**Results:**

Here, we present ViralClust, a modular Nextflow pipeline for bias-aware representative selection from large viral genome datasets. ViralClust integrates 5 distinct clustering algorithms (CD-HIT-EST, SUMACLUST, VSEARCH, MMSeqs2, and HDBSCAN) within a unified workflow, enabling direct comparison of clustering outcomes and flexible adaptation to diverse biological questions, considering a balanced phylogenetic distribution of the selected sequences. We evaluated ViralClust on 6 RNA and DNA virus datasets ranging from 632 to 156,586 sequences and spanning genome lengths from 890 to 197,185 nucleotides. Across all datasets, clustering reduced dataset size by ~95% or more while preserving genetic diversity across species, genera, and families, and effectively mitigating biases introduced by outbreaks, partial genomes, and sequence orientation artifacts.

**Conclusions:**

By supporting whole-genome clustering and scalable representative selection, ViralClust enables efficient and reproducible downstream analyses that would otherwise be computationally infeasible. Rather than offering a prescriptive, guided analysis engine, our framework functions as a flexible comparative collection of complementary strategies, allowing users to empirically evaluate trade-offs and choose the ideal method tailored to their specific analytical endpoints.

## Background

Viral genome sequences are becoming increasingly available [1–5]. For example, for SARS-CoV-2 alone, nearly 9.2 million uploaded nucleotide sequences are accessible (NCBI Virus, 19.11.2025). The use of such large datasets of viral sequences often poses problems for downstream analyses, such as sequence alignment, phylogeny, and visualization, in terms of memory usage, run time, and visualization [6, 7]. Calculating a multiple sequence alignment (MSA) of available viral genomes of a family is often computationally not feasible. Disregarding the technical demands, an MSA with tens of thousands of entries may not be informative for most users, since the overall alignment quality potentially decreases. Several strategies have been proposed to handle large-scale viral datasets. Reference-based alignment approaches map sequences against a single reference genome, reducing computational overhead but potentially missing diversity in divergent strains [8–10]. Similarly, *k*-mer-based methods enable rapid, alignment-free comparison of sequences and have been widely used for taxonomic classification and similarity estimation [11–13]. However, both approaches are primarily designed for sequence comparison rather than dataset reduction, and neither directly addresses the problem of selecting a representative, diversity-preserving subset of genomes for downstream analysis. Besides the size of the datasets, the over-representation of a certain species due to an outbreak might also introduce a bias into the data. To reduce the number of entries, random sampling of genomes does not solve the introduced bias of over-represented species; it may even increase the effect of the bias. Consequently, the aim is to reduce the dataset of viral sequences without losing genetic diversity. In this context, cluster-based representative sequence selection offers a distinct advantage: by grouping sequences according to their similarity and retaining one representative per cluster, the resulting subset reflects the full breadth of genetic diversity present in the original dataset, regardless of sampling biases introduced by outbreaks or sequencing efforts. Instead of random selection, clustering viral genomes based on, e.g., sequence similarity and selecting a representative genome for each cluster results in a better-balanced subset of input viruses. While the idea of clustering is simple, the correct method is often hard to find and depends on the underlying scientific question. Depending on the biological question and distribution of biological data, the principle of *compactness* is most widely used, aiming to keep intra-cluster variation small. It is implemented in tools such as CD-HIT-EST [14], SUMACLUST [15], VSEARCH [16], and MMSeqs2 [17, 18]. In this context, the sequence identity determines whether a sequence belongs to a cluster or forms a new, independent cluster. Thus, compactness clustering is based on sequence homology. In most implementations, a sequence is only compared against one representative of established clusters. This method is used frequently for viral clades [19–21]. However, evolution is a continuous process, and many novel observed viruses might not be unambiguously assigned to the calculated clusters based on static sequence similarity thresholds alone. An algorithm following the principle of *connectedness* may be suitable for such continuous data. The principle of connectedness exploits the idea that neighboring data points should belong to the same cluster. Therefore, algorithms following this principle tend to detect smaller, local components within the data that are not concentrated around a center point [22]. For datasets with viral genomes such that the data points are not well-described by high-dimensional spheres, as is the case for compactness clusters, we propose a tool such as HDBSCAN [23]. Given the vast diversity of viral architectures and evolutionary dynamics, prescribing a single decision tree or a uniform set of rules for parameters would compromise analytical robustness. Therefore, ViralClust is explicitly not designed as a guided, automated agent, but rather as an exploratory, comparative toolbox that allows users to directly evaluate context-dependent performance. For bioinformaticians, evaluating which tool to choose presents its own challenges, as the absence of a definitive ground truth complicates the determination of which sequences best represent a cluster.

Here, we present ViralClust, a Nextflow pipeline designed to select representative viral genomes, by a streamline clustering analysis for virology research. Unlike conventional methods, ViralClust integrates clustering via 5 distinct existing tools, enabling direct and comprehensive comparison of the results. One key feature of ViralClust is its robust data preprocessing capabilities. We address common database errors, such as inconsistencies in sequence orientation (-ssRNA sequences found in both positive and negative orientations) and ambiguous gene arrangements in ambisense viruses. By correcting these mistakes, we enhance the accuracy of clustering outcomes. Moreover, ViralClust introduces innovative methodologies previously unexplored in virology research. For instance, full genome clustering based on *k*-mers of nucleotide sequences utilizing Hierarchical Density-Based Clustering (HDB) represents a novel approach in virus research, offering previously inaccessible insights. Finally, we provide comprehensive statistics, including cluster sizes, and visualize the sequence selection in rough phylogenetic representations without explicitly generating phylogenies of the viral families. Additionally, our HTML output facilitates easy cluster sorting, including direct links to corresponding NCBI genomes for further analysis. All parts of the developed pipeline are written modular, and the user may adjust parameters or exchange and add complete tools. ViralClust offers the possibility of adding manually additional viral **g**enomes **o**f **i**nterest (GOI) to the input data. We evaluated the performance of ViralClust on 6 different datasets of RNA and DNA viruses varying in number of available sequences, sequence length, and sequence diversity, such as segmentation and orientation.

## Data description

### Viral data

We downloaded viral genomes labeled as “complete” of *Orthoebolavirus, Hepacivirus hominis* (HCV), *Orthoflavivirus, Flaviviridae, Alphainfluenzavirus influenzae* (formerly *Influenza A virus*, IAV), and *Monkeypox virus* from the NCBI Virus database; see Table 1. All sequences used in this study are deposited in a Zenodo repository in fasta format [24].

**Table 1:** Overview of datasets used in this study

					Number of genomes after clustering						
Taxonomic clade	*∅* SeqLen	#Seq	PreProc	%nr	cd	suma	vse	mmseq	hdb	Time	Peak RAM
*Orthoebolavirus*	18.9 kb	632	443	70.09	8	7	7	11	11	12 min	11.4 G
*Hepacivirus hominis*	9.6 kb	1,075	1,071	99.63	55	30	58	55	23	14 min	10.7 G
*Orthoflavivirus*	9.2–11 kb	8,052	7,681	95.39	69	71	70	63	101	1 h 22 min	43.2 G
*Flaviviridae*	9–13 kb	9,895	9,478	95.79	220	180	221	211	194	2 h 47 min	52.6 G
IAV (1)	2,341 nt	83,543	56,973	68.20	66	390	90	280	1,412	3 h 27 min	273.9 G
IAV (2)	2,341 nt	82,569	56,212	68.07	56	338	73	216	1,311	3 h 24 min	270.9 G
IAV (3)	2,233 nt	83,400	55,862	66.98	63	305	75	265	1,371	3 h 16 min	268.8 G
IAV (4, H)	1,775 nt	156,586	108,514	69.30	300	1,237	464	1,574	2,307	8 h 36 min	510.0 G
IAV (5)	1,565 nt	84,653	49,465	58.43	46	262	76	179	1,218	2 h 28 min	238.3 G
IAV (6, N)	1,409 nt	114,639	73,786	64.36	228	773	329	640	1,622	4 h 9 min	572.2 G
IAV (7)	1,027 nt	96,928	46,371	47.84	25	185	38	269	981	1 h 47 min	225.1 G
IAV (8)	890 nt	85,944	42,308	49.23	51	218	69	264	802	1 h 28 min	207.2 G
*Monkeypox virus*	190–200 kb	1,077	999	92.76	14	1	3	23	2	16 h 0 min	6.5 G

ViralClust reduces the number of input genomes to a representative set of genomes. Data were downloaded from NCBI Virus. IAV—*Alphainfluenzavirus influenzae*; *∅*SeqLen—average sequence length; #Seq—number of input genomes/segments; contains only records labeled as “complete” ; PreProc—number of sequences after pre-processing; % nr—percentage of non-redundant sequences; Number of genomes after clustering—only considering clusters with a size greater than one; cd—CD-HIT-EST; sum—SUMACLUST; vse—VSEARCH; mmseq—MMSeqs2; hdb—HDBSCAN; Time—run time of ViralClust measured by Nextflow; Peak RAM—peak RSS (resident set size) of ViralClust measured by Nextflow. A detailed overview of time and memory consumption is shown in supplementary Supplementary Table S1.

### “Ground Truth” of virus phylogeny

To compare the results of ViralClust, we considered the classification of the ICTV [25, 26] as “ground truth” for *Orthoebolavirus, Orthoflavivirus, Flaviviridae*. For *Hepacivirus hominis* and *Monkeypox virus*, we included the NCBI taxonomy. The number of genera, subgenera, and/or species is derived from the ICTV taxonomy release [27]; see Supplementary Table S2. For *Alphainfluenzavirus influenzae*, we included the classification according to the Centers for Disease Control and Prevention (CDC) [28] into 18 different H subtypes and 11 different N subtypes. This theoretically results in 198 possible subtypes, but 136 subtypes are currently listed in the NCBI taxonomy (23.01.2026).

## Analyses

### The ViralClust pipeline

Our pipeline ViralClust takes a multiple fasta file as input and applies 5 different cluster tools to the data to select representative genomes. We calculate and report basic statistics for each of the cluster tools. Based on the respective representative genomes, ViralClust further calculates an MSA and a rough phylogenetic representation (parameter --eval). Figure 1 gives a general overview of the pipeline. Additionally to the clustering step, the user can retrieve an optional basic evaluation of the results. This evaluation is no recommendation for any algorithm or tool, as the choice of clustering depends on the underlying scientific question. However, it gives a broad overview of the data and may help determine the appropriate cluster method. If applicable, ViralClust finds GenBank accession IDs in the fasta records identifier and retrieves meta information from the NCBI database. Lastly, the user can specify a second, optional fasta file containing genomes that must be present in the final set of representative genomes. This may prove useful if comparative analyses between viruses are carried out and the user wants to include a specific, unpublished viral strain or isolate in the analysis.

**Figure 1:**
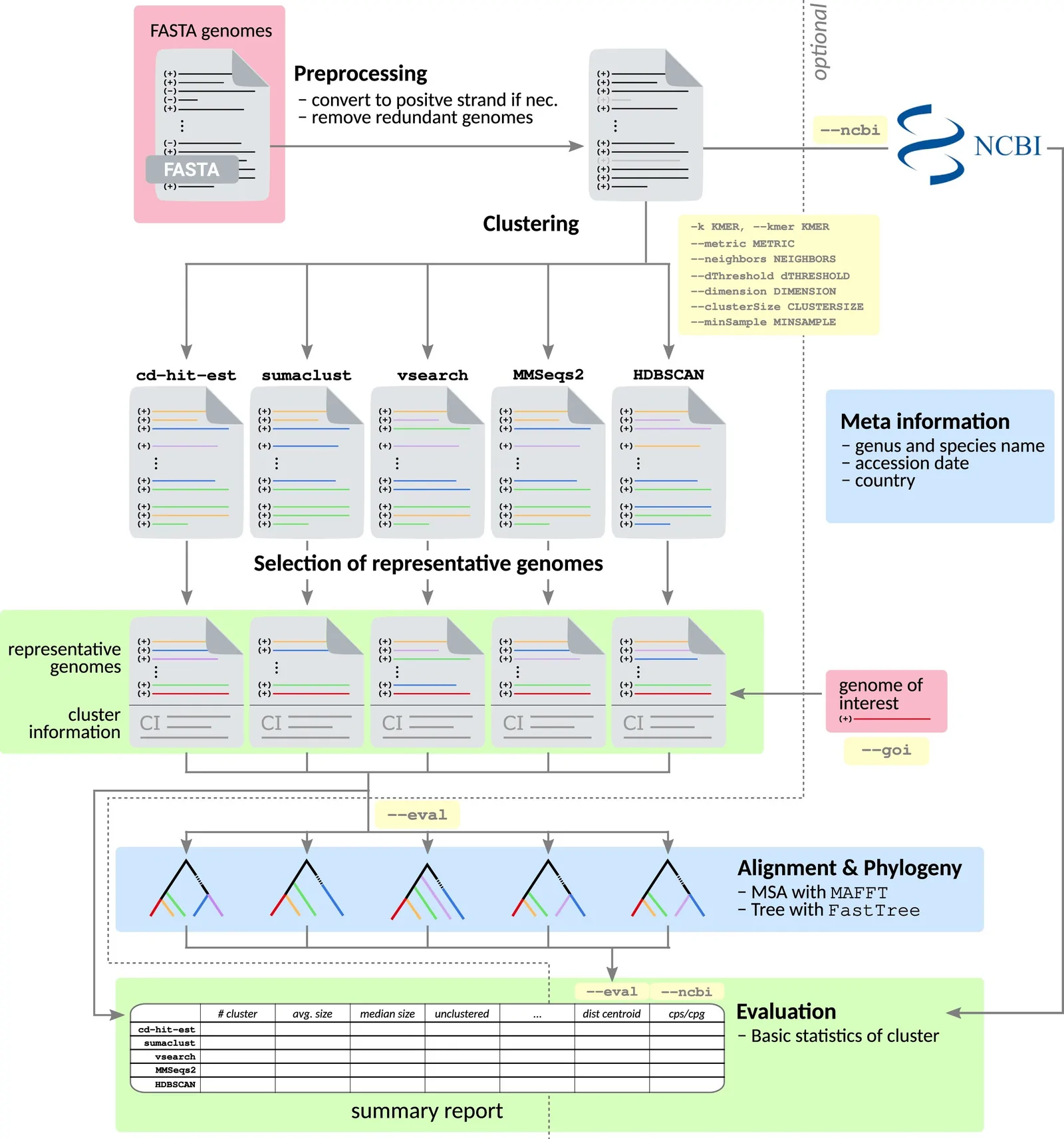
The general ViralClust pipeline. *Preprocessing*. Input genomes are filtered for redundant genomes and oriented to the positive strand determined via an ORF density scan. *Clustering*. Five different tools are pre-installed. However, each can be exchanged by other customized clustering tools. For each clustering method applied, ViralClust provides an overview of which sequence falls into which cluster. *Representative genomes*. Further, a fasta file with representative sequences for each cluster is provided for each tool. --goi— Genomes of interest (GOI) or unpublished genomes can be added to the representative genomes. *Evaluation*. The basic statistics of the cluster results, including average and median cluster size and the average distance of 2 representative genomes, are given for each cluster tool. *Optional parameters*. --ncbi—For each applicable sequence, i.e., for each fasta record with a valid GenBank accession ID in its identifier, ViralClust retrieves additional information from the NCBI database and includes it in the summary report. --eval—For each clustering method, ViralClust can provide an alignment of representative genomes and a phylogenetic representation of these genomes.

#### Workflow of ViralClust

We implemented ViralClust in Nextflow (v22.10.06) [29]. We developed our scripts with Python (compatible with v3.6+). We employ individual conda (v4.9.2) [30] environments for each step per default to guarantee no conflicts during installation and usage. ViralClust is publicly available at GitHub (link in “Availability of source code and requirements” section). We further provide a test dataset with a small tutorial on using ViralClust. In the following, we describe the pipeline depicted in Fig. 1 in detail, including implemented tools and parameter configurations.

##### Preprocessing

To ensure high-quality data for subsequent clustering, ViralClust discards sequences with more than 10% of non-ACGU characters. The user can adjust this filtering process using the --max_ambiguous parameter. Both strands are then scanned for ORF density to ensure that all sequences have the same orientation. Especially in the case of single-stranded negative RNA viruses, genomes are sometimes uploaded into the database in different orientations. This causes cluster algorithms to separate genomes based on this orientation, potentially jeopardizing the complete clustering approach. We calculate ORF density by translating an input genome into all 6 reading frames. We define an open reading frame (ORF) as a sequence of at least 200 amino acids between 2 stop codons or between a single stop codon and the 5′ or 3′ end of the sequence. This makes the procedure robust against missing stop codons at the end of the sequence and missing start codons. We calculate the forward-strand ORF density by summing the lengths of the ORFs in the 3 forward reading frames, then dividing by the sequence length. The backward-strand ORF density is calculated analogously. This method accounts for real or erroneous frameshifts. We use the strand with the higher ORF density for further calculations. The step is enabled by default; the user can disable it using the parameter sort_off. To remove 100% redundant genomes in the input dataset, we apply the easy-linclust module of MMSeqs2 (v14) [17, 18], with parameters --min-seq-id 1.0. The filtering during preprocessing is applied globally to the original dataset, and the filtered data serves as the input for each subsequent clustering tool.

##### Clustering

Per default, ViralClust applies 5 different clustering methods. Depending on which scientific virological question is asked, one of the tools might be more suitable than another. Due to the modularity of the pipeline, the user can also choose to switch off the predefined cluster tools and/or add individual cluster methods. Currently, we implemented CD-HIT-EST (v4.8.1) [14], SUMACLUST (v1.0.31) [15], VSEARCH (v2.15) [16] from vsearch, MMSeqs2 (v14) [17, 18], and HDBSCAN (v0.8.26) [23] as individual Nextflow modules. Whenever applicable, we set the parameters of the tools to match each other to make them comparable. By default, ViralClust instructs CD-HIT-EST, SUMACLUST, VSEARCH, and MMSeqs2 to cluster sequences with an identity of at least 90% (default value of CD-HIT-EST). Since MMSeqs2 offers a variety of clustering modules, we have integrated easy-linclust (default) and easy-cluster.

##### Connectedness clustering with HDBSCAN

As a novel approach to cluster viral genomes, we further implemented a combination of PCA and HDBSCAN [23] in ViralClust. (1) *k-mer distributions:* For this approach, we translate each input genome into a vector of *k*-mer frequencies (default *k=7*), neglecting *k*-mers consisting of non-canonical nucleotides. (2) *Dimension reduction:* For the PCA, we use the module as implemented in scikit-learn (v1.2.2) [31]. Instead of the dimension reduction with PCA, we further allow using UMAP for the reduction. (3) *Clustering:* For efficient clustering, we reduce the resulting $4^k$-dimensional (default *k=7*) vector space and thus provide an embedding for the clustering algorithm HDBSCAN. Per default, we either reduce the dimension to *d=50* or until the sum of explained variance of the components is at least 0.7. After dimension reduction, each viral genome is represented by a vector of maximal length *d* (*d=50*). HDBSCAN can be seen as a hybrid approach of hierarchical-based (connectivity) and density-based (compactness) clustering approaches. For 2 vectors, HDBSCAN first calculates mutual reachability distance. Per default, we use the cosine distance implemented in SciPy (v1.4.1) [32] as metric. However, we allow for a plethora of pre-defined distances, which can be used via the --metric parameter of our HDBSCAN module. We implemented the (standardized) Euclidean, Manhattan, Chebyshev, Minkowski, Canberra, Braycurtis, and Mahalanobis distance metrics via the SciPy library [32]. Since the used implementation of HDBSCAN cannot deal with the cosine distance, we normalized the vectors using the L2 normalization. Subsequently, we use HDBSCAN with the Euclidean distance on these normalized vectors as recommended [33].

The user can override or provide additional parameters for each tool via the nextflow run viralclust.nf command. Exemplarily, for HDBSCAN, the user can use the flag --hdbscan_help to get an overview of implemented options, which can be used using --hdbscan_params. Analogously, help messages and parameters for the other tools can be used and accessed via their respective commands.

##### Determination of representative genomes

The implemented tools report representative genomes based on sequence length (CD-HIT-EST, SUMACLUST), sequence abundance (VSEARCH), and selection within cascaded clustering (MMSeqs2). For the HDBSCAN module, we calculate centroid sequences based on the average pairwise distance between all vectors in the same cluster. The sequence with the minimum average distance is selected as the representative genome for this cluster. Given a distance metric *d*, the centroid sequence $\mathcal {C}$ of a cluster *C* with size *N* is the one that has the lowest average distance $\overline{d}$ to all other sequences $S_i$ (*i∈ [1,N]*) of the cluster: $\overline{d_\mathcal {C}} = \min _{i\in C} \frac{1}{N-1}\sum \limits _{j=1}^{N}d(S_i,S_j)$. Thus, each cluster is represented by an input genome, not an “artificial” median genome. Per default, we use the cosine distance as metric *d* but also allow for other metrics.

In ViralClust, only clusters with a size of more than one are defined as “real” clusters, and the corresponding representatives are saved in a file. Clusters of size one are therefore not considered clusters, but named “singletons” here, and are stored in a separate file. It is important to note that any sequence classified as noise from HDBSCAN is treated as a cluster of size one and is therefore considered an unclustered sequence in ViralClust. The combined set of cluster representatives and “singleton” sequences is provided in a third file. This allows the user to choose which set of sequences is most suitable for the research question.

##### Optional parameters

(1) *Inclusion of a genome of interest:* in addition to the general input file, the user can specify a second fasta file with the --goi flag. This fasta file should contain one (or more) **g**enome(s) **o**f **i**nterest (GOI). If the user provides such a file, ViralClust will include the contained genomes in the final set of representative genomes. Further, in the result reports of the individual cluster tools, the GOIs are specifically marked. With this option, users can add their unpublished in-lab strain or mutant to downstream analyses and can further determine the closest phylogenetic viruses based on the resulting clusters. This also enables comparisons between the representative sequences and specific reference genomes, which are not necessarily cluster representatives. (2) *Metadata information*: with the --ncbi flag ViralClust links additional information provided by the NCBI [34]. For this, we can sequence identifiers for GenBank accession ID. If found, we use the accession ID to retrieve additional information about the taxonomy (species and genus name), accession date, and the country the sequence originated from. ViralClust combines this metadata with the overview of clusters for each tool. (3) *Alignment and phylogeny*: if the user sets the flag --eval of ViralClust, we further create an MSA (default: MAFFT (v7.4) [35]) of the representative genomes for each cluster tool. We subsequently use the alignments to build phylogenetic representations (default: FastTree (v2.1.10) [36]), which we finally visualize using Newick Utilities (v1.6) [37]. If --eval is enabled, we include the average phylogenetic distance calculated by FastTree between 2 representative genomes in the final summary report. Per default, FastTree uses the Jukes–Cantor distance model [38].

### Benchmark ViralClust by a variety of datasets

We applied ViralClust to 6 viral datasets of single-stranded RNA and double-stranded DNA viruses varying in number of sequences, sequence length, and sequence diversity. For an overview of the datasets used in this study, we refer to Table 1, in which we provide the total number of available sequences, the number of sequences after the implemented filtering steps, and the number of clusters resulting from each tool. Given these input sets, we report the number of clusters as calculated by the individual tools in their respective column, run time, and memory usage; see Table 1 and Supplementary Table S1. All results generated by ViralClust are deposited in a Zenodo repository [24].

To provide an objective basis for comparing tool performance across datasets, we evaluated all 5 clustering algorithms using a scoring framework comprising 5 component scores: the reduction score (RS), singleton penalty score (SPS), clustering quality score (CQS), taxonomic concordance score (TCS), and cluster-to-taxonomy ratio (CTR), aggregated into an Overall Score (OS) with a maximum of 5. A detailed description of each metric is provided in the “Methods” section. The resulting scores are summarized in Table 2. All scores are shown in Supplementary Table S3.

**Table 2:** Overall score (OS) of the 5 clustering algorithms integrated into ViralClust across all evaluated datasets

	Overall score				
Taxonimic clade	cd	suma	vse	mmseq	hdb
*Orthoebolavirus*	4.622	4.682	4.687	3.940	3.720
*Hepacivirus hominis*	3.926	3.798	4.040	3.963	3.998
*Orthoflavivirus*	4.331	4.141	4.340	4.508	3.294
*Flaviviridae*	3.516	3.523	3.540	3.555	3.022
IAV (1)	4.422	4.852	4.930	4.424	4.384
IAV (2)	4.446	4.836	4.909	4.401	4.414
IAV (3)	4.396	4.829	4.911	4.502	4.365
IAV (4, H)	4.655	4.838	4.938	4.561	4.474
IAV (5)	4.385	4.871	4.925	4.415	4.239
IAV (6, N)	4.612	4.859	4.929	4.571	4.385
IAV (7)	4.545	4.687	4.785	4.693	4.131
IAV (8)	4.605	4.808	4.873	4.665	4.247
*Monkeypox virus*	2.690	2.311	4.161	2.763	4.996
Sum of scores	55.151	57.035	59.968	54.961	53.669

The OS is the sum of 5 component scores—reduction score (RS), singleton penalty score (SPS), clustering quality score (CQS), taxonomic concordance score (TCS), and cluster-to-taxonomy ratio (CTR)—each in the range [0,1], giving a maximum OS of 5. Higher values indicate better overall clustering performance with respect to dataset reduction, singleton rate, phylogenetic cluster separation, and taxonomic concordance. IAV—*Alphainfluenzavirus influenzae*; cd—CD-HIT-EST; sum—SUMACLUST; vse—VSEARCH; mmseq—MMSeqs2; hdb—HDBSCAN. All scores are shown in Supplementary Table S3.

The scores reveal a considerable spread both across tools and across datasets, with OS values ranging from 2.311 (SUMACLUST, *Monkeypox virus*) to 4.996 (HDBSCAN, *Monkeypox virus*), reflecting the diversity of the evaluated datasets rather than a consistent ranking of tools. No single tool dominates across all datasets, and tool choice should therefore be guided by the biological question and dataset composition. Several general trends emerge from the scores and are discussed in detail in the respective subsections below. Compactness-based tools (CD-HIT-EST, SUMACLUST, VSEARCH) tend to score consistently well across RNA virus datasets, producing fewer, more compact clusters that achieve strong dataset reduction, but may collapse rare or biologically meaningful lineages in taxonomically diverse datasets. Noteworthy, SUMACLUST discards sequences containing characters other than ACGU, making it even more dependent on the quality of the input data and possibly difficult to use the results as a representative genomic dataset for subsequent analyses. MMSeqs2 produces higher cluster granularity, better preserving sequence-level diversity at the cost of larger representative sets, though its behavior becomes less predictable at very large dataset sizes. HDBSCAN is particularly well suited for datasets with continuous diversity distributions, but carries a fundamental limitation for datasets containing rare subtypes, which may not form sufficiently dense regions to be recognized as independent clusters and are instead treated as noise (here defined as unclustered). These observations are intended as a starting point for tool selection; the scoring framework provided by ViralClust allows users to reproduce the same evaluation on their own datasets.

#### Small and biased dataset—*Orthoebolavirus*

We tested the *Orthoebolavirus* dataset consisting of only 632 genomes to see how fine-grained ViralClust can work. *Orthoebolavirus* is a genus within the *Filoviridae* family (ICTV phylogeny; see Supplementary Fig. S1). The genomes are negative single-stranded RNA and about 18.9 kb in length [39].

In comparison to other viral clades, this dataset includes a small number (6) of well-described species according to the ICTV; see Table 3, with a notable presence with 461/632 sequences of *Orthoebolavirus zairense* (Ebola virus, EBOV), a human pathogen over-represented due to several outbreaks. We aim to investigate the selection of representative genomes with ViralClust for such a small, well-defined, and unbalanced dataset. The preprocessing of ViralClust reduced the dataset size by 30% to 443 genomes. Subsequent clustering resulted in 7–11 representative genomes; see Table 1. In general, all 5 clustering tools could distinguish the species in individual clusters; see Fig. 2. A conventional representation of the phylogeny of the representative genomes and the corresponding ICTV classification can be found in Supplementary Fig. S2. Additionally, in Supplementary Fig. S3, a RAxML-NG phylogenetic tree based on a full-genome MAFFT alignment can be found as an overview of the 443 individual sequences. CD-HIT-EST, SUMACLUST, VSEARCH, and MMSeqs2 correctly revealed 1 cluster representative for the 5 species BOMV, BDBV, RESTV, SUDV, and TAFV each. A notable exception is HDBSCAN, which could not identify the species TAFV, Fig. 2B and Supplementary Fig. S2F . All tools clustered TAFV genomes (light blue) correctly together with BDBV (dark green). Each of the tools selected at least 1 natural EBOV strain (CD-HIT-EST:1, SUMACLUST:1, VSEARCH:1, MMSeqs2:4, HDBSCAN:7). Additionally, CD-HIT-EST, SUMACLUST, VSEARCH, and MMSeqs2 selected up to 2 representatives of EBOV derived from recombination or reverse genetics experiments (see Fig. 2 dashed gray box). HDBSCAN selected only natural EBOV sequences (isolated from viral infections). Interestingly, this comparatively high number of representative genomes represents geographic and temporal separation of EBOV outbreaks.

**Figure 2:**
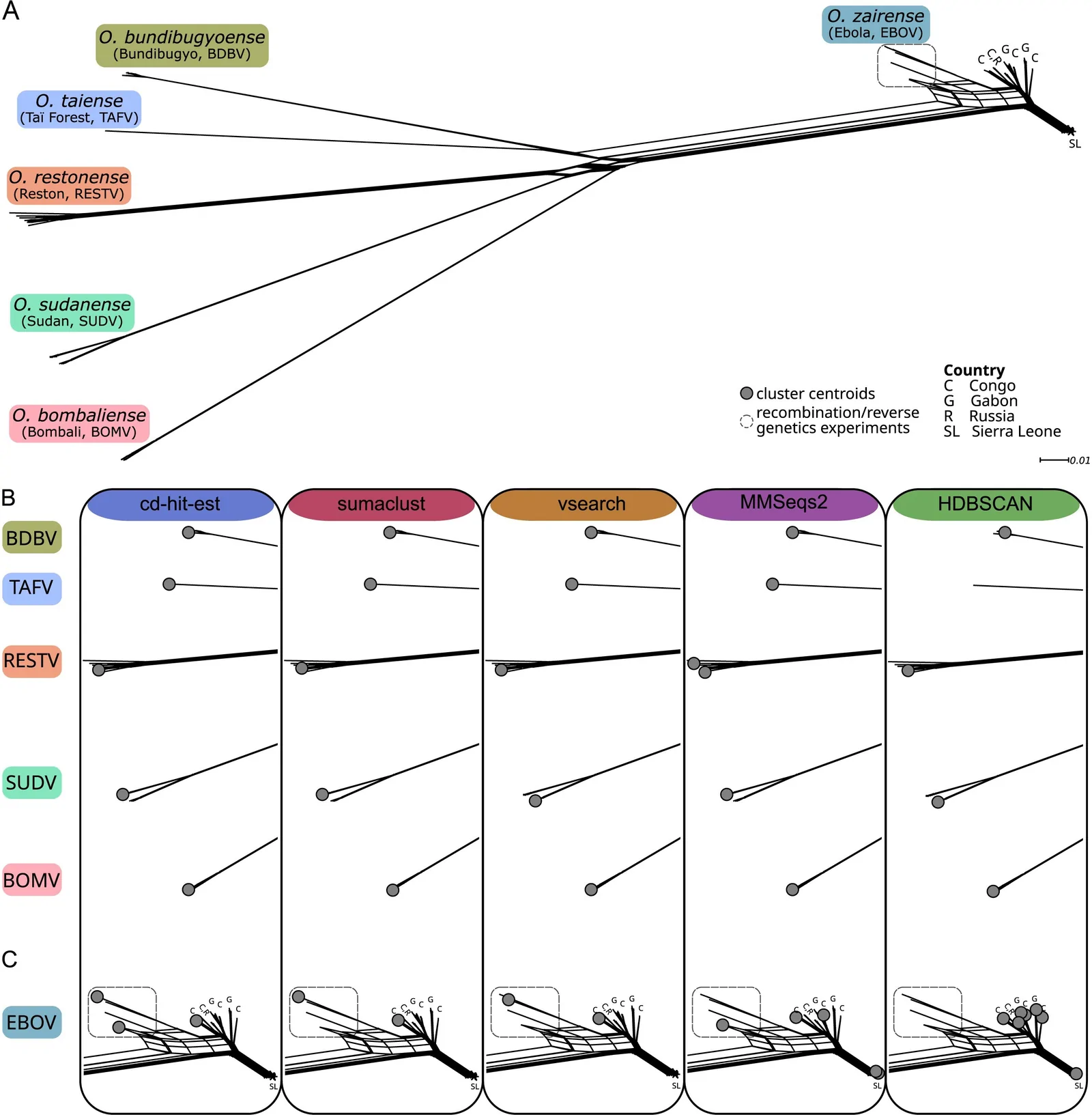
(A) Split graph of the *Orthoebolavirus* dataset with labeled species. (B) Cluster representatives of CD-HIT-EST, SUMACLUST, VSEARCH, MMSeqs2, and HDBSCAN in the subtrees of BDBV, TAFV, RESTV, SUDV, and BOMV (top to bottom). (C) Cluster representatives in the EBOV subtree. Gray dots display the cluster representative sequences selected by the individual tools. The Zaire *Orthoebolavirus* subtree shows a distinction by geographic origin, indicated as follows: C—Congo; G—Gabon; R—Russia; SL—Sierra Leone. The split graph is reconstructed using SplitsTree based on the MAFFT alignment of the complete genome sequences (443 genomes) resulting from the first filtering steps of ViralClust.

**Table 3:** *Orthoebolavirus* species including virus name and abbreviation (Abbr.) according to ICTV and number of downloaded sequences per species (#Seqs)

Species	Virus name	Abbr.	#Seqs
*O. bombaliense*	Bombali	BOMV	8
*O. bundibugyoense*	Bundibugyo	BDBV	24
*O. restonense*	Reston	RESTV	22
*O. sudanense*	Sudan	SUDV	21
*O. taiense*	Taï Forest	TAFV	3
*O. zairense*	Ebola	EBOV	461

A total of 93 sequences were unclassified regarding their species.

#### Species *Hepacivirus hominis* and genus *Orthoflavivirus* from the family *Flaviviridae*

*Flaviviridae* is a large family of positive single-stranded RNA viruses consisting of four genera: *Orthoflavivirus, Pestivirus, Pegivirus*, and *Hepacivirus* [40], with a total of 97 species (ICTV; see Supplementary Fig. S4). *Flaviviridae* includes human pathogenic viruses such as Zika virus, tick-borne encephalitis virus, dengue virus, and hepatitis C virus (HCV). Genomes are approximately 9–13 kb in length [40]. We chose 3 datasets to compare results on species, genus, and family levels. On the species level, we chose *Hepacivirus hominis* (HCV infecting humans) as a well-described and phylogenetically closely related species. On a genus level, we chose *Orthoflavivirus* due to the well-described dataset being very diverse (ICTV: 53 species). We chose *Flaviviridae* at the family level. The datasets *Hepacivirus hominis* and *Orthoflavivirus* are complete subsets of the dataset *Flaviviridae*. Finally, our focus was on how the selected representatives from species and genus studies are consistent with the selection from the entire virus family. Preprocessing within the ViralClust pipeline reduced the datasets by 1–5%; see Tables 1 and 4.

**Table 4:** Cluster statistics for *Hepacivirus hominis* (HCV), *Orthoflavivirus* (*Flavivirus*), and *Flaviviridae* datasets

	Algorithm	# clstr	min	max	avg	med	singl	d. cen	cps	cpg	PD ratio
HCV	cd	55	2	410	17.71	3.0	97	0.804	1.75	23.00	0.170
	suma	30	2	373	24.67	3.0	42	0.637	2.08	15.00	0.072
	vse	58	2	412	16.97	3.0	87	0.806	1.74	24.00	0.173
	mmseq	55	2	410	17.75	3.0	95	0.814	1.82	17.00	0.172
	hdb	23	5	412	40.87	14.0	131	0.594	1.61	21.00	0.058
*Flavivirus*	cd	69	2	1,642	110.14	5.0	81	1.510	1.44	46.00	0.275
	suma	71	2	1,622	96.44	5.0	86	1.663	1.57	49.00	0.322
	vse	70	2	1,431	108.63	6.0	77	1.493	1.42	46.00	0.308
	mmseq	63	2	1,798	120.68	6.0	78	1.704	1.32	30.00	0.289
	hdb	101	5	1,371	69.19	19.0	693	1.189	2.49	100.00	0.231
*Flaviviridae*	cd	220	2	1,642	41.54	3.0	339	2.466	1.64	20.60	0.302
	suma	180	2	1,622	44.81	3.0	267	2.739	1.73	21.00	0.268
	vse	221	2	1,431	41.46	3.0	316	2.280	1.62	27.75	0.316
	mmseq	211	2	1,798	43.33	3.0	336	2.520	1.54	18.00	0.311
	hdb	194	5	1,371	41.98	14.0	1,333	1.704	2.09	39.40	0.148

For each tool, the basic cluster statistics are listed. cd—CD-HIT-EST; sum—SUMACLUST; vse—VSEARCH; mmseq—MMSeqs2; hdb—HDBSCAN; # clstr—number of clusters (with a size greater than one); min/max—size of smallest/largest cluster; avg/med—average/median cluster size; d. cen—average phylogenetic distance between 2 representative genomes; singl—number of singletons (=cluster with only one element); cps—average cluster per species; cpg—average cluster per genus; PD ratio—ratio between Faith’s phylogenetic distance (PD) of the full tree and the representative tree. The values cps and cpg give a measurement of how much a single species/genus is divided into different groups.

##### Species *Hepacivirus hominis*

*Hepacivirus hominis* (HCV) consists of 8 genotypes [41–43], however, only 7 are listed in the ICTV; see Supplementary Fig. S4 yellow rectangle. With the neighbor-net method, we gained 13 clearly distinguishable clusters; see Fig. 3. This difference can also be explained by the observations of virologists in the past: (i) 2 additional clusters, besides the 7 downloaded genotypes, are formed by recombinants: the natural recombinants (e.g., 2k/1b or 2b/1b, Fig. 3—close related to genotype 1b) [44–46] and the artificial recombinants (Fig. 3—between genotype 1 and 2) [47–49]. Note: further recombinants are mixed into genotype 2. (ii) Three additional clusters are formed within genotype 1—in total, divided into genotype 1, 1a, 1b, and rare genotypes 1 (1c–1n). Currently, 14 subtypes are listed in the ICTV [40, 50, 51]; (iii) one additional cluster exists due to the diverse genotype 6: currently, 33 subtypes are known for genotype 6 according to the ICTV [40, 52], with subtype 6a being the first one discovered [53]. All sequences part of subtype 6a cluster are distinguishable from other subtypes, with the notable exception of subtype 6b, which is closer to 6a than other subtypes. The tools of ViralClust calculated between 23 (HDBSCAN) and 58 (VSEARCH) representative genomes (see Fig. 3 colored circles and Table 1). Although any of the methods represents 3–8 times more clusters than the currently recognized number of HCV genotypes, using more representatives may be necessary to capture the considerable diversity within genotypes. Among the evaluated methods, HDBSCAN produced the largest clusters (minimum size 5, average size 40.87, median size 14; see Table 4), resulting in the lowest total number of clusters. All clustering tools in ViralClust selected representative genomes distributed across the phylogenetic tree. However, substantial differences in the number of representatives were observed for the different genotypes; see Fig. 3. Interestingly, none of the tools was able to identify genotype 7 as an individual cluster.

**Figure 3:**
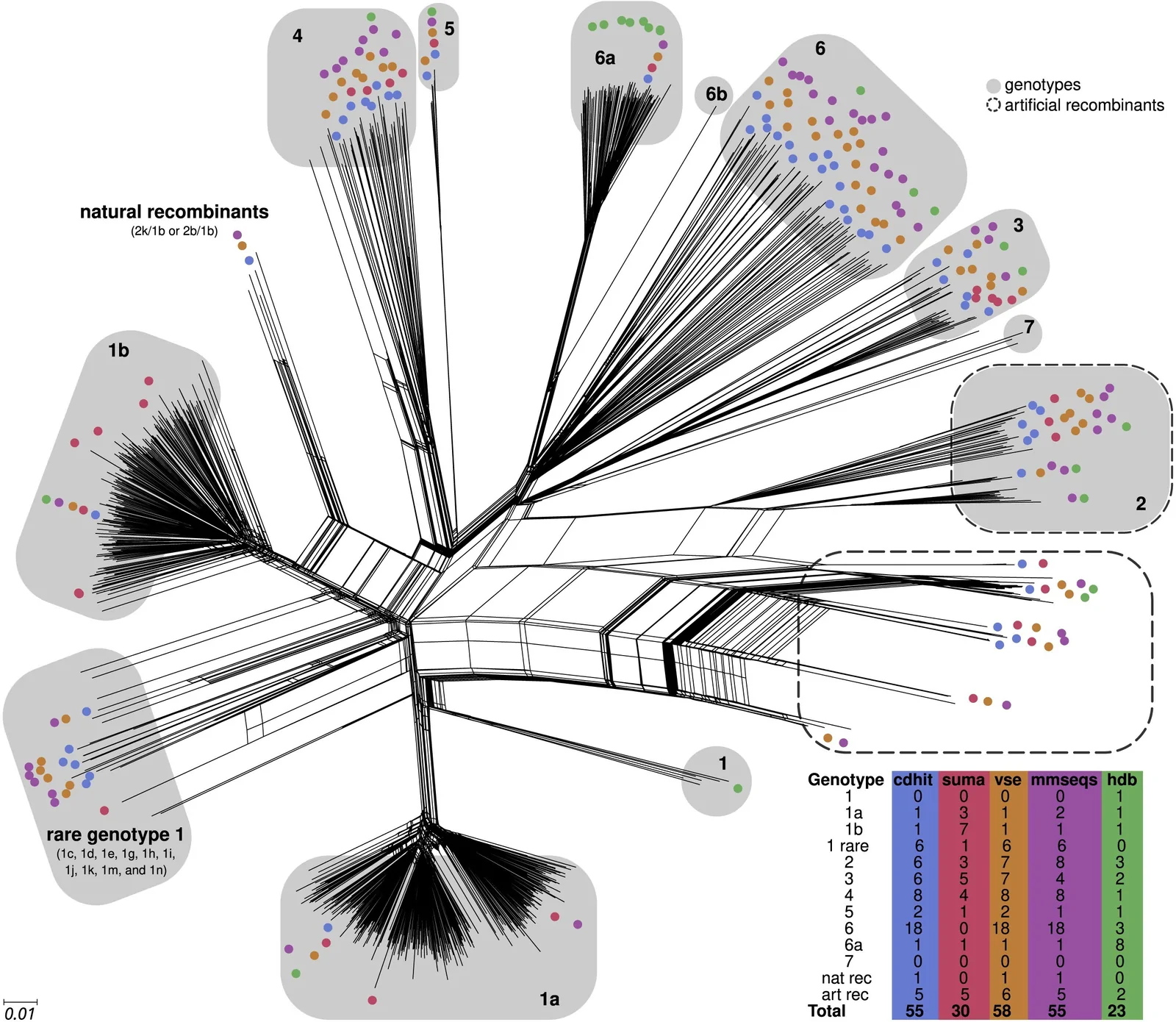
Splits graph of HCV with labeled genotypes (gray), artificial (dashed box), and natural recombinants, and cluster representatives of the 5 tools incorporated in ViralClust (CD-HIT-EST, SUMACLUST, VSEARCH, MMSeqs2, and HDBSCAN). Numbers in gray boxes represent genotypes. The tree can be divided into 13 subtrees, whereas genotype 1 consists of 4 subtrees (1, 1a, 1b, and the rare genotypes 1c–1n); and all subtype 6a genomes are clearly distinguishable from all other subtypes summarized here as genotype 6. All known natural recombinants (e.g., 2k/1b or 2b/1b) form a separate subtree most closely related to genotypes 1b and 4. Some artificial recombinants mix with genotype 2 genomes, whereas others form a subtree between genotypes 1 and 2. The number of representatives per genotype is shown in the table at the bottom right. The split graph is reconstructed using SplitsTree based on the MAFFT alignment of the complete genome sequences (1,071 genomes) resulting from the first filtering steps of ViralClust.

Across all 5 clustering algorithms, the largest cluster was consistently identified as genotype 1a. Each tool selected 1–3 representatives for this cluster. While 4 tools (CD-HIT-EST, VSEARCH, MMSeqs2, HDBSCAN) selected the same representative genome for genotype 1b, SUMACLUST identified 7. Notably, HDBSCAN did not select any rare subtypes of genotype 1, whereas the other tools included 1 (SUMACLUST) or 6 (CD-HIT-EST, VSEARCH, MMSeqs2) representatives. This is likely due to the *k*-mer–based similarity measure used by HDBSCAN, which appears to group the rare genotype 1 subtypes more tightly, leading to no additional representatives. A small subtree annotated as genotype 1 (Fig. 3, bottom right) was only found to be an independent cluster by HDBSCAN, which selected a single genome from these variants. The large number of subtypes within genotype 6 can be explained by the diversity of this genotype; see Fig. 3, even though the tools picked different representatives. The tools CD-HIT-EST, VSEARCH, and MMSeqs2 selected 18 representative genomes for genotype 6, whereas HDBSCAN identified substantially fewer representatives (3). These differences may be attributed to the distinct algorithmic strategies employed: HDBSCAN is based on *k*-mer similarity, while the other tools combine heuristic approaches with (pairwise) sequence alignments, which may increase sensitivity to finer sequence variation. Interestingly, SUMACLUST did not identify genotype 6 as a distinct cluster. This may be explained by the filtering step in SUMACLUST, which removes sequences containing non-ACGU characters and may have excluded genotype 6 genomes prior to clustering. In contrast, HDBSCAN selected 8 representatives for subtype 6a, whereas the remaining tools selected only 1. This difference may be influenced by the high abundance and relative homogeneity of subtype 6a genomes, in combination with the density-based clustering approach used by HDBSCAN. Several genomes in the dataset were labeled as recombinants, leading to a split in the bottom right part of the tree (Fig. 3, dashed box) and potentially inflating the number of clusters. These recombinant genomes are annotated as a mix between genotypes 1 and 2. Overall, all tools except HDBSCAN produced broadly comparable results. While HDBSCAN tended to select more representatives in densely connected regions (e.g., genotype 6a), it selected fewer genomes in other parts of the tree. When examining individual sequences, they are usually assigned to the same cluster, although the chosen representative may differ slightly; see Fig. 4.

**Figure 4:**
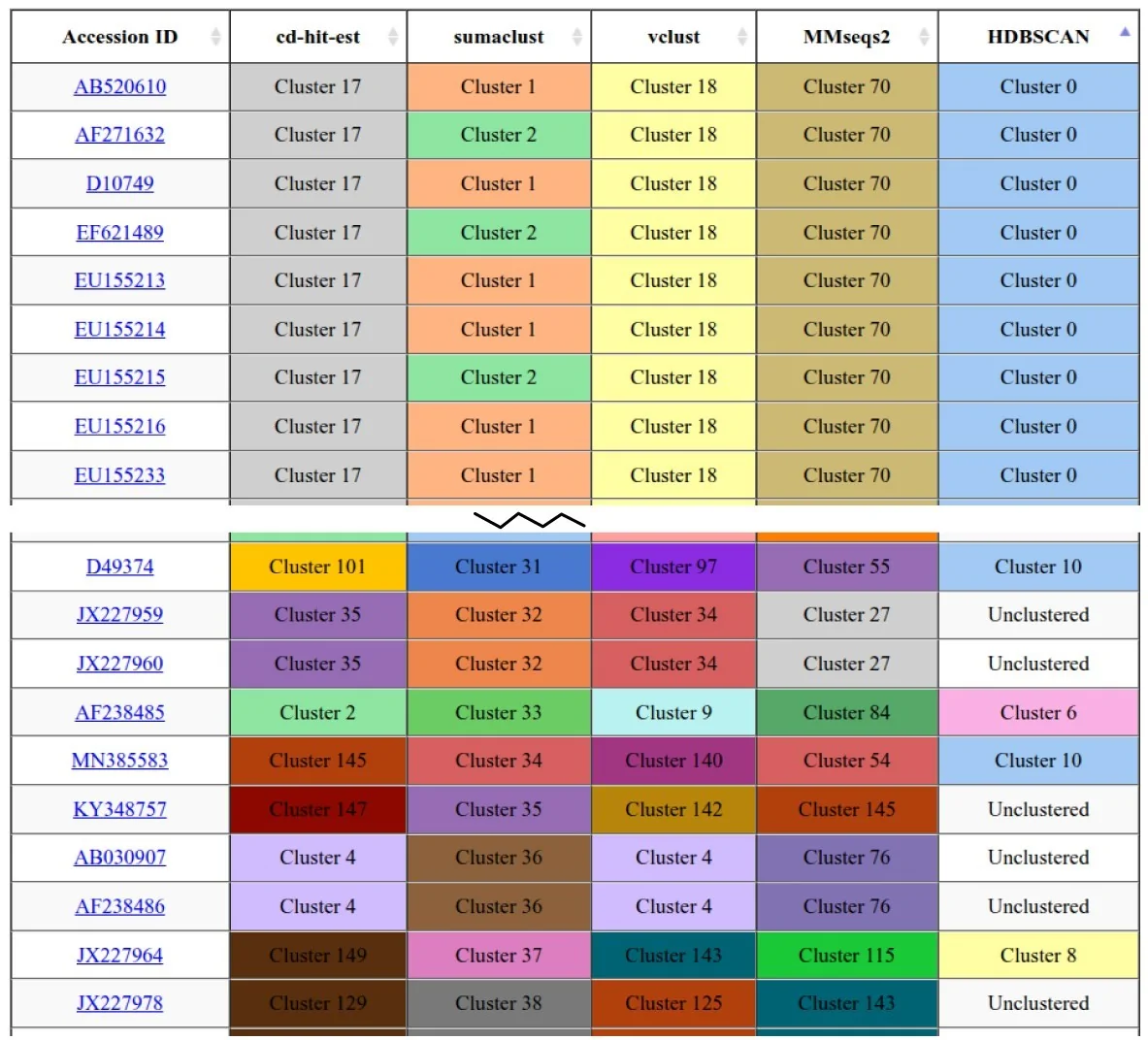
Optional output of ViralClust in web-based format (HTML) for the dataset HCV. The interactive visualization allows the user to (i) sort the table by tool results or accession IDs, (ii) view and compare color-coded clusters, and (iii) access the NCBI entry for each sequence (link, if available). For most sequences, the clustering results of the different tools are consistent (top). Differences between the tools become apparent (bottom), where some sequences form single-sequence clusters (e.g., D49374), others consistently form small clusters across tools (e.g., AB030907 and AF238486), and individual sequences are not assigned to any cluster by specific tools, such as the HDBSCAN “unclustered” sequences. The complete table is provided in the electronic supplement.

##### Genus *Orthoflavivirus*

The clustering of 8,052 *Orthoflavivirus* resulted in 63–101 clusters with the different tools, whereas MMSeqs2 yields the smallest number of clusters and HDBSCAN the highest; see Table 4. As for HCV, the number of clusters is higher than the number of species (53). The selection of more than one genome per species as representative can be sufficient to cover greater diversity across the whole genus of *Orthoflavivirus*. Despite minor differences, all 5 clustering algorithms produced broadly similar results, with representatives distributed across the phylogenetic representation of the dataset (see Supplementary Fig. S5).

When comparing the selected representatives with the ICTV species assignments, which contain exactly one reference genome per species, see Supplementary Fig. S4 violet background, we find (a) clusters which contain exactly one of the defining ICTV species, as expected; (b) ICTV species representatives not part of any cluster (= singletons); (c) clusters, which have no defining ICTV species, which might point to a generally more fine-grained clustering; and (d) some clusters have been merged by specific tools, so that 2 (or more) ICTV species could be found in one cluster, which should not have happened in order to represent the current phylogeny well.

The different tools found between 16 and 23 clusters containing exactly one ICTV species representative; see Table 5, whereas 18–23 ICTV reference genomes were not assigned to any cluster with more than one sequence, and thus remain as singletons within the ViralClust pipeline. This likely reflects recently added species for which only limited sequence data are currently available. An important detail is that SUMACLUST excludes any sequences containing characters other than A, C, G, or T. As a result, the ICTV representative genome for St. Louis encephalitis was removed from the dataset during preprocessing. Between 45 and 84 clusters contained no ICTV reference genome. These clusters likely represent intraspecific diversity or unclassified lineages, providing additional resolution beyond the current ICTV taxonomy. CD-HIT-EST, SUMACLUST, VSEARCH, and MMSeqs2 agreed to cluster 2 reference genomes of ICTV together into one cluster: Saboya (NC_033697) together with Potiskum (NC_029054). HDBSCAN also merged Saboya (NC_033697) together with other species, but in this case together with Modoc (NC_003635), Uganda S (NC_033698), and Wesselsbron (EU707555). For more details, see Supplementary Tables S4 and S5. However, if higher resolution is required, we recommend including singletons as well. In this case, users should be aware that incorporating singletons may lead to over-representation of certain regions within the dataset. HDBSCAN often adapts to the density of subtrees: for very dense clusters, it selects several representatives (see Supplementary Fig. S5 top right), whereas clusters consisting of a single sequence are typically not chosen as representatives and instead remain unassigned. If such outliers are of interest for downstream analysis, users should manually include these singletons in subsequent steps (as these sequences are saved in a separate file). As a result, HDBSCAN yields the lowest average cluster size (69.19) and the largest number of singletons (693). An important observation concerns the previously proposed subclassification within *Orthoflavivirus* according to their vector type [40]. These analyses are based solely on parts of the RdRp; see Supplementary Fig. S4. Our analysis is based on complete genomes, indicating that, at least for subtrees, the phylogeny cannot be drawn based on the vector type; see Fig. 5.

**Figure 5:**
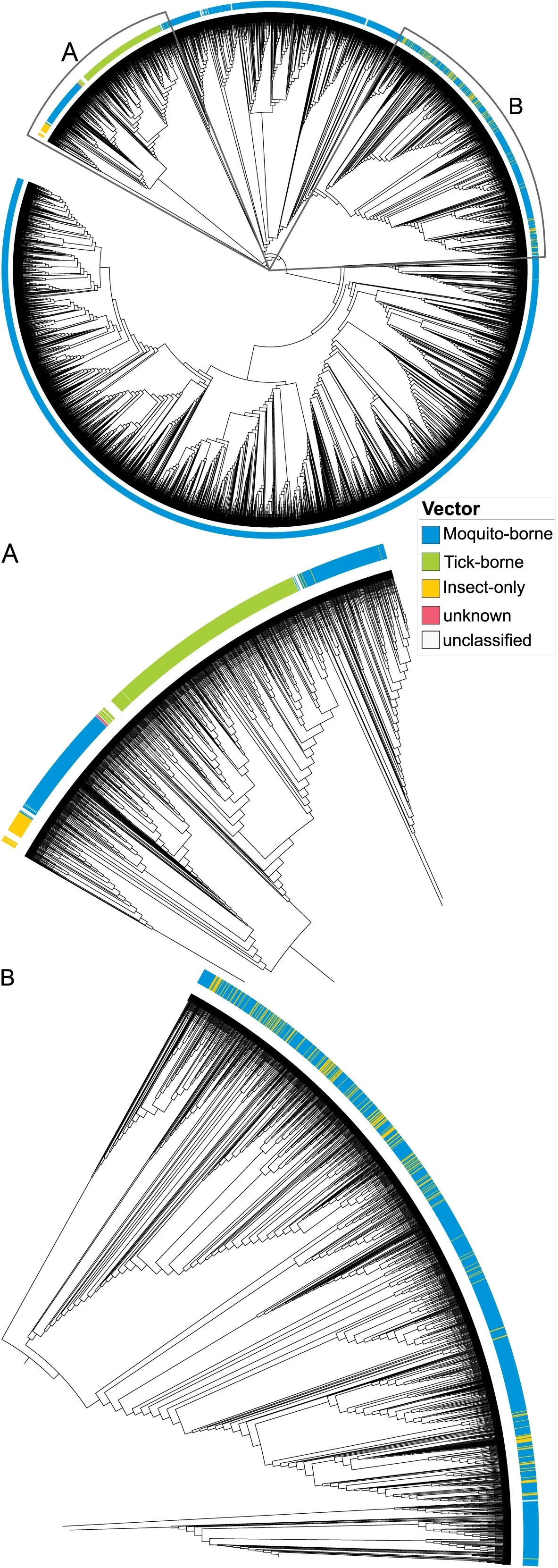
Phylogenetic representation based on the *Orthoflavivirus* dataset with color-coded vectors. Within the subtrees A and B, parts of the tree do not follow a vector-based classification. The complete and enlargeable tree is shown in Supplementary Fig. S6. The tree is reconstructed using FastTree based on the MAFFT alignment of the complete genome sequences (7,681 genomes) resulting from the first filtering steps of ViralClust.

**Table 5:** Comparing the clustering results of the *Orthoflavivirus* dataset with the species chosen for the phylogeny of ICTV; see Supplementary Fig. S4

	*\checkmark*	Singleton	*\emptyset*	Discrepancies
cd	23	18	45	1*×*2
suma	20	20	50	1*×*2
				St. Louis encephalitis (DQ525916) removed
vse	23	18	46	1*×*2
mmseq	23	18	39	1*×*2
hdb	16	23	84	1*×*4

*\checkmark* —number of clusters with exactly 1 ICTV representative (in agreement with known phylogeny); Singleton—number of clusters containing one ICTV representative; *\emptyset*—number of clusters without ICTV representative (showing additional information for downstream analysis); Discrepancies—(a) number of clusters with *> =2* ICTV representatives (e.g., “1x4” for 4 ICTV representatives in one cluster); (b) number of ICTV representatives that are “singletons” (these sequences are alone in a cluster); (c) other issues (e.g., SUMACLUST removes sequences including nucleotides $n \notin \lbrace A,C,G,T\rbrace$). For a more detailed overview, see Supplementary Tables S4 and S5.

##### Family *Flaviviridae*

Clustering of all 9,895 *Flaviviridae* species resulted in 180–221 clusters, with SUMACLUST generating the lowest number of clusters and VSEARCH the highest; see Table 4. These results are twice as many as the reported 97 species from ICTV [69], Supplementary Fig. S4, as for the subsets of HCV species and *Orthoflavivirus* genus.

The average cluster size remains about the same for all 5 algorithms. MMSeqs2 produced the overall largest cluster (1,798 sequences), whereas HDBSCAN (1,371) and SUMACLUST (1,431) produced the smallest, with 200–400 fewer sequences. HDBSCAN also produced the largest number of singletons, with a notably high fraction (1,333); see Table 4. The ICTV phylogeny (Supplementary Fig. S4) was constructed using the RdRp region of 125 sequences [40]. To evaluate how well this single-gene phylogeny reflects whole-genome evolutionary relationships, we compared it against a phylogeny inferred from 9,895 complete *Flaviviridae* genomes. While the overall topology of the full-genome-based phylogeny aligns broadly with the RdRp-based ICTV classification, Supplementary Fig. S7, several notable discrepancies emerged. Specifically, as visible in Fig. 6 (from left to right) (i) 25 genomes annotated as orthoflaviviruses clustered together in a distinct outlier subtree; (ii) 94 HCV genomes appeared more closely related to most of the pegiviruses than to other hepaciviruses; (iii) a single *Pestivirus* genome (Trinbago virus) was located within this divergent HCV cluster (not visible); (iv) another 3, more divergent, *Pegivirus* sequences clustered with the majority of HCV; and (v) 9 *Orthoflavivirus* genomes were placed nearer to basal pestiviruses (Fig. 6 A—blue part between yellow and green). These discrepancies may be partly explained by misannotations in public sequence databases, but they also highlight the inherent limitations of single-gene phylogenies for robust virus taxonomy.

**Figure 6:**
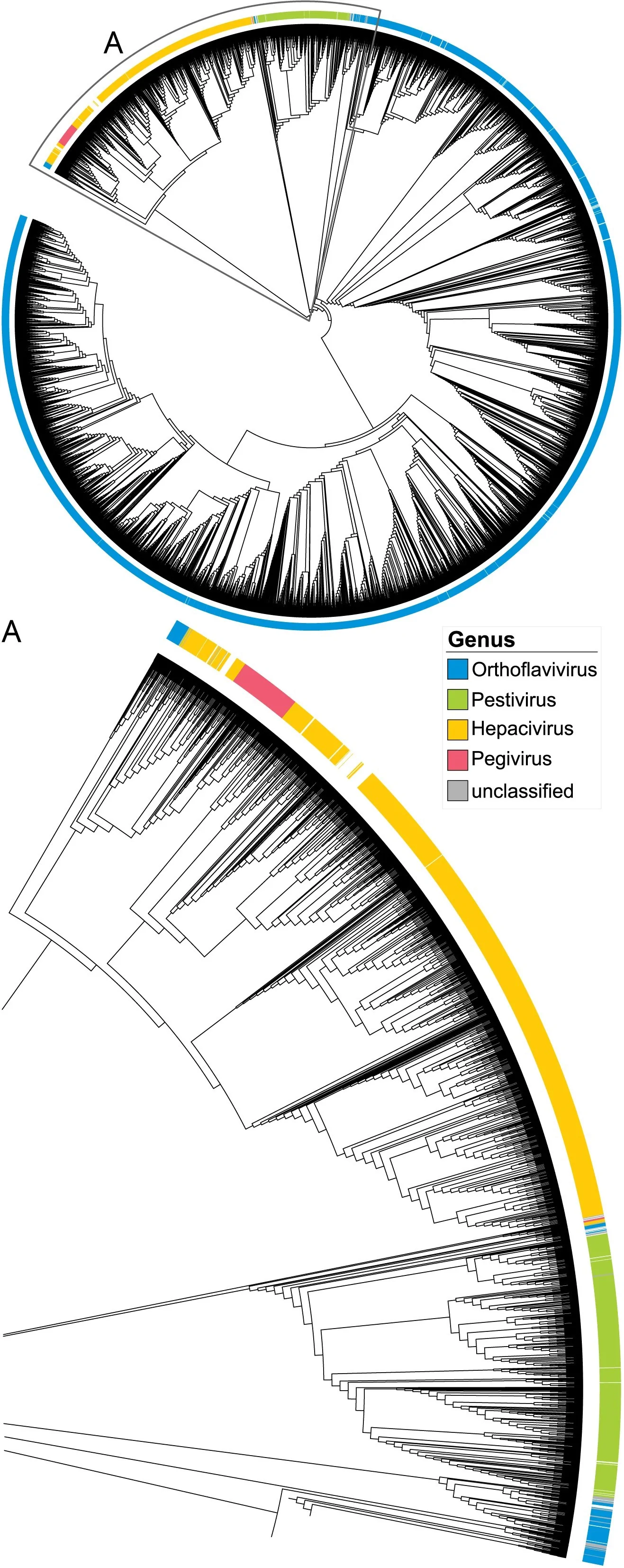
Phylogenetic representation of the *Flaviviridae* dataset with highlighted genera. (A) Parts of the tree that do not follow the classification by genus. Complete tree shown in Supplementary Fig. S7. The tree is reconstructed using FastTree based on the MAFFT alignment of the complete genome sequences (9,478 genomes) resulting from the first filtering steps of ViralClust.

##### Comparing the datasets

We chose *Flaviviridae* on the family level (9,895 genomes) to test the robustness of the different clustering approaches, especially in comparison to clustering on the species level (HCV, 1,075 genomes, a true subset of *Flaviviridae*) and on the genus level (*Orthoflavivirus*, 8,052 genomes, a true subset of *Flaviviridae*); see Table 1. The fine-grained clustering observed at the species level is for some clustering algorithms also preserved at the family level; see Table 6. For example, within genomes of HCV, the methods CD-HIT-EST, SUMACLUST, and VSEARCH consistently formed the same clusters and selected the same representatives when compared between the HCV only and HCV clustered together with all other *Flaviviridae* datasets. MMSeqs2 shows slight differences in the selection of representatives; see Supplementary Fig. S8. Clustering the entire *Flaviviridae* family with MMSeqs2 produced 56 clusters for HCV, compared to 55 clusters when only the HCV species were analyzed. Of these, 54 clusters were identical, while one cluster in the HCV clustering was split into 2 in the *Flaviviridae* analysis. Among the representatives, 49 were identical, and the remaining non-identical ones were phylogenetically close. In contrast, HDBSCAN showed larger differences in both cluster composition (only one identical cluster) and choice of representatives (6 identical). When restricted to HCV, HDBSCAN produced twice as many clusters (23) compared to when HCV was analyzed as part of the *Flaviviridae* dataset (12). Genotypes 1a and 1b are represented by multiple genomes in the clustering of HCV, while these are grouped together in the results of *Flaviviridae*. This suggests that HDBSCAN is more sensitive to the chosen level and number of input sequences and adapts its clustering accordingly. When switching to the comparison on a genus level at the example of *Orthoflavivirus* only vs. *Orthoflavivirus* within *Flaviviridae*, all 5 algorithms generated the same largest cluster, containing dengue virus 1; see Table 4. SUMACLUST and VSEARCH again produced identical clusters and representatives, indicating robustness to the number of input sequences; see Table 6. For the genus *Orthoflavivirus*, CD-HIT-EST and MMSeqs2 selected the same total number of representatives—69 and 63, respectively—as in the corresponding *Orthoflavivirus* subsets of the *Flaviviridae* analysis. While CD-HIT-EST produced identical cluster compositions, 8 representatives differ. Similarly, MMSeqs2 yielded 56 clusters with unchanged composition, but 48 identical representatives. In both cases, the differing representatives were phylogenetically close, and thus these discrepancies are considered minor (Supplementary Fig. S9). In contrast, HDBSCAN generated substantially more clusters for *Orthoflavivirus* when clustering within the full *Flaviviridae* dataset (156 representatives) compared to clustering *Orthoflavivirus* alone (101 representatives). Of these, 27 clusters and 80 representatives were identical. The largest differences were observed in the mosquito-borne clade, where HDBSCAN selected considerably more representatives than the other algorithms (Supplementary Fig. S9). The run time does not scale linearly with dataset size; see Supplementary Table S1. Although the *Orthoflavivirus* dataset accounts for 81% of the *Flaviviridae* sequences (7,681 of 9,478), ViralClust required only 48.5% of the total run time (1 h 22 min of 2 h 47 min; Table 1). For the HCV dataset, the effect was less pronounced: comprising 11.3% of the sequences in the *Flaviviridae* dataset, it required 8.3% of the run time (14 min of 2 h 47 min; Table 1).

**Table 6:** Comparison of the clustering results of *Hepacivirus hominis* only vs. *Hepacivirus hominis* within *Flaviviridae* and *Orthoflavivirus* only vs. *Orthoflavivirus* within the *Flaviviridae* analysis

*Hepacivirus hominis*: only vs. within *Flaviviridae*		
	clstr-comp.	repr
cd	*\checkmark* (56 clstr)	*\checkmark*
suma	*\checkmark* (31 clstr)	*\checkmark*
vse	*\checkmark* (59 clstr)	*\checkmark*
mmseq	54 id. (55/56)	49 id.
hdb	1 id. (23/12)	6 id.
*Orthoflavivirus*: only vs. within *Flaviviridae*		
	clstr-comp.	repr
cd	*\checkmark* (69 clstr)	57 id.
suma	*\checkmark* (71 clstr)	*\checkmark*
vse	*\checkmark* (70 clstr)	*\checkmark*
mmseq	56 id. (63/74)	48 id.
hdb	27 id. (101/158)	80 id.

clstr-comp.—cluster composition, i.e., number of identical clusters (number of clusters in *Hepacivirus hominis* or *Orthoflavivirus*/number of clusters in *Flaviviridae*); repr—identical representatives; *\checkmark*—results are identical (id.). cd—CD-HIT-EST; sum—SUMACLUST; vse—VSEARCH; mmseq—MMSeqs2; hdb—HDBSCAN.

The overall memory consumption scales almost linearly when comparing the *Flaviviridae* dataset with the *Orthoflavivirus* dataset (183.9 G, 164.3 G; Supplementary Table S1). In contrast, this is not the case for the HCV dataset, where memory usage is 4 times higher than for *Flaviviridae* when comparing with the size of the datasets.

#### *Alphainfluenzavirus influenzae* as an example for segmented viruses including partial and reverse-complement sequences

*Alphainfluenzavirus influenzae* (formerly *Influenza A virus*, IAV) provides a representative case for studying segmented viral genomes and handling truncated reverse-complement sequences. Here, we address several key questions relevant for sequence clustering and analysis of such viruses: we first confirm that genotype represents surprisingly well for serotype classification. For the 8 genome segments, sequences can be analyzed individually or concatenated; here, we show that independent analysis captures meaningful differences. Partial genomes are reliably placed in phylogenetic trees, demonstrating robustness. Finally, reverse-complement sequences are detected through characteristic patterns (in this section, highlighted with gray background); the current version of ViralClust automatically restores the positive orientation to simplify analysis.

IAV is a segmented (8 segments), negative-sense, single-stranded RNA virus belonging to the family *Orthomyxoviridae* (see Supplementary Fig. S10). Classification is traditionally based on the 2 surface proteins hemagglutinin (H, segment 4) and neuraminidase (N, segment 6), with 18 H and 11 N subtypes reported by the CDC (see Supplementary Figs S11 and S12). Our analyses confirm that segment data reflect subtype classification well, demonstrating that genotype approximates subtype and that segment-wise analysis captures meaningful biological differences.

Each of the 8 genome segments in our dataset contains on average 98,532 sequences, with the largest datasets corresponding to segments 4 (156,586) and 6 (114,639) and the smallest to segment 2 (82,569). Segment lengths range from 890 nt (segment 8) to 2,341 nt (segments 1 and 2), and non-redundant sequence fractions vary from 64 to 69% for segments 1–4 and 6 to 47–49% for segments 7 and 8.

Clustering results (90% sequence identity, *k*-mer length 7) vary widely depending on segment and method, ranging from 25 clusters (CD-HIT-EST, segment 7) to 2,307 (HDBSCAN, segment 4); see Table 1, with the largest datasets (segments 4 and 6) also yielding the highest number of clusters across all 5 tools. This wide range illustrates that tool choice has a substantial impact on the granularity of the resulting representative set, independent of the underlying biological diversity. CD-HIT-EST produced the lowest number of clusters for all segments, followed by VSEARCH. MMSeqs2 and SUMACLUST are close to each other in terms of the number of clusters. MMSeqs2 yielded a lower number of clusters for segments 1, 2, 3, 5, and 6, but more for 4, 7, and 8. HDBSCAN resulted in the highest number of clusters by far. The consistently lower cluster counts of CD-HIT-EST and VSEARCH reflect their more aggressive merging strategies, which may be advantageous when the goal is maximal dataset reduction.

The coverage of known subtypes provides a more biologically grounded basis for comparing tool performance than cluster counts alone. For segments 4 and 6, which encode the surface proteins, CD-HIT-EST, SUMACLUST, VSEARCH, and MMSeqs2 select representatives covering all known subtypes; see Table 7 and Figs 7 and 9. In contrast, HDBSCAN fails to select representatives for H17 and H18 (segment 4) and N10 and N11 (segment 6). These are among the rarest subtypes in the dataset, and their absence from the HDBSCAN representative set is likely a fundamental consequence of density-based clustering: subtypes represented by very few sequences may not form sufficiently dense regions to be recognized as independent clusters, and are instead treated as noise.

**Figure 7:**
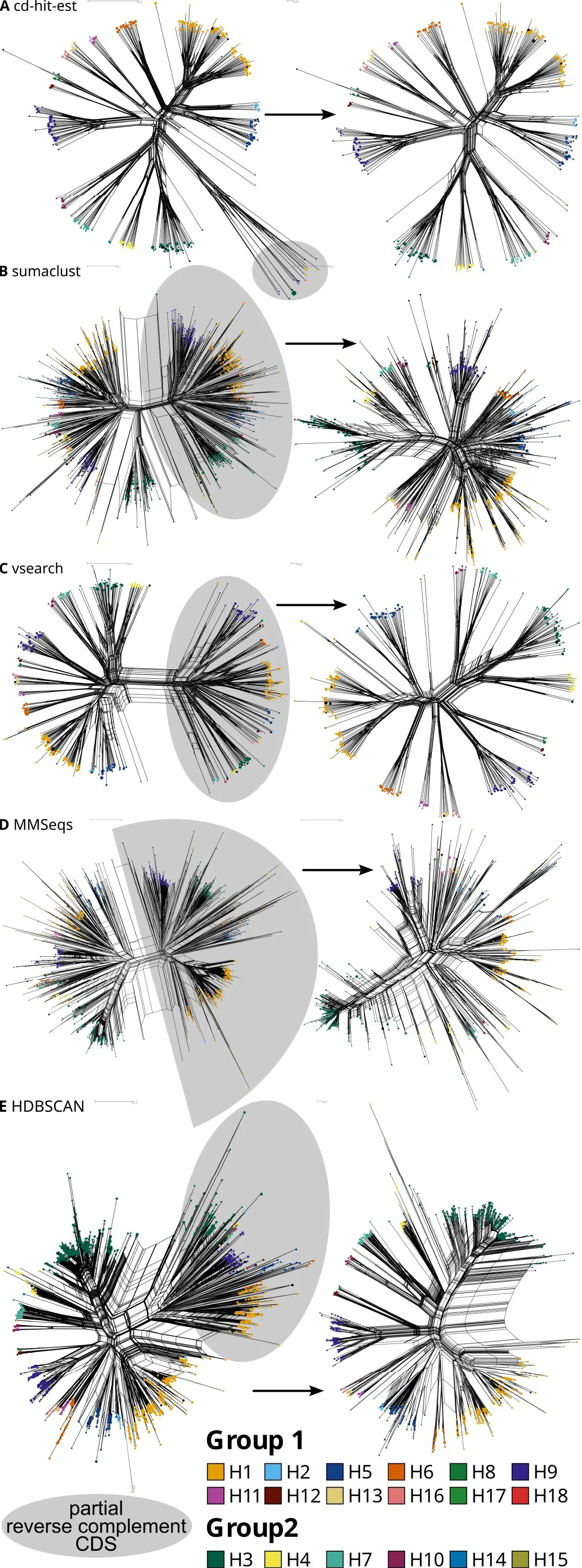
Split graphs of IAV segment 4 (H) representatives of (A) CD-HIT-EST, (B) SUMACLUST, (C) VSEARCH, (D) MMSeqs2, and (E) HDBSCAN. Left—all representatives (including partial and reverse-complement); Right—only complete genomes. The node size indicates cluster size. The split graphs are reconstructed using SplitsTree based on the MAFFT alignments. Gray background highlights reverse-complement sequences (19.22% of the non-redundant dataset). HDBSCAN did not select representatives for subtypes H17 and H18. They were manually marked as framed circles. For details, see Supplementary Figs S13–S17.

**Figure 8:**
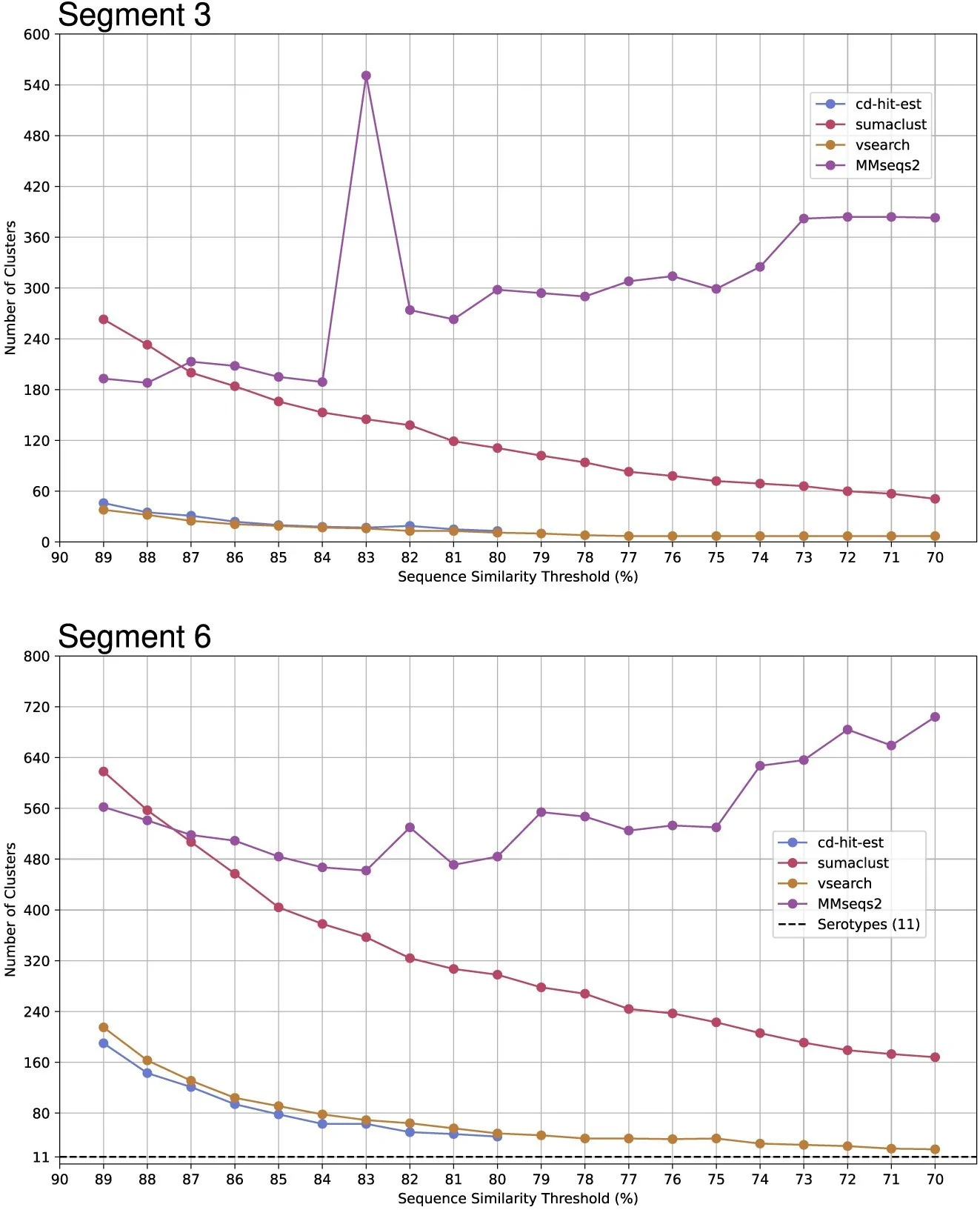
Number of clusters of IAV segments 3 and 6 with sequence similarity thresholds from 70 to 90%. CD-HIT-EST only allows a minimum of 80%. Only clusters with a size greater than 1 are considered. For more details, see Supplementary Tables S7 and S8.

**Figure 9:**
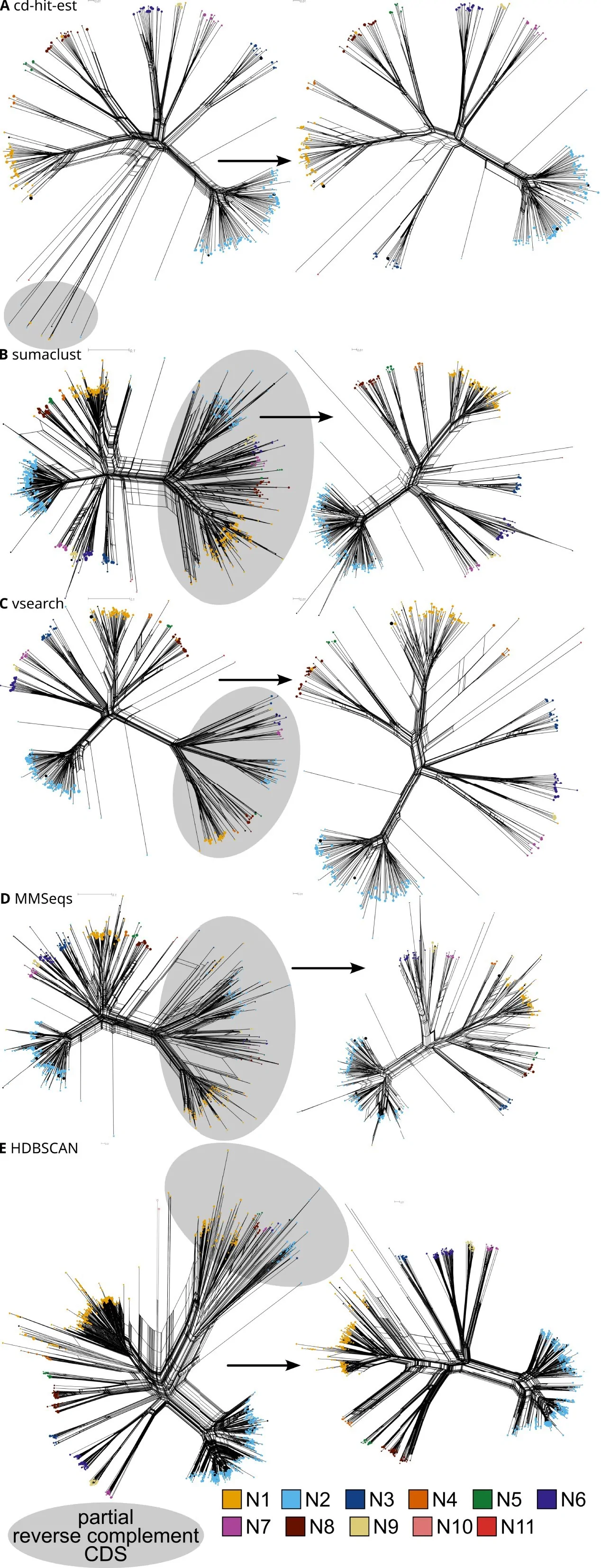
Split graphs of IAV segment 6 (N) representatives of (A) CD-HIT-EST, (B) SUMACLUST, (C) VSEARCH, (D) MMSeqs2, and (E) HDBSCAN. Left—all representatives (including partial and reverse-complement); Right—only complete genomes. The node size indicates cluster size. The split graphs are reconstructed using SplitsTree based on the MAFFT alignments. Gray background highlights reverse-complement sequences (11.37% of the non-redundant dataset). HDBSCAN did not select representatives for subtypes N10 and N11. They were manually and marked them as framed circles. For details, see Supplementary Figs S18–S22.

**Table 7:** Number of representatives per subtype for segments 4 (H) and 6 (N)

Subtype	#repr				
	cd	suma	vse	mmseq	hdb
Segment 4 (H)					
H1	83	352	128	527	613
H2	5	26	10	19	35
H3	34	215	59	460	654
H4	14	44	22	28	58
H5	27	145	43	107	210
H6	19	54	27	33	67
H7	16	37	22	23	100
H8	5	9	7	5	11
H9	44	193	71	242	347
H10	9	27	11	17	42
H11	7	16	10	13	19
H12	4	9	7	3	22
H13	8	20	11	13	21
H14	2	1	1	1	1
H15	2	2	2	2	2
H16	6	13	10	9	13
H17	1	1	1	1	0
H18	1	1	1	1	0
Segment 6 (N)					
N1	47	250	73	198	476
N2	90	280	130	276	733
N3	17	29	21	21	27
N4	6	15	10	5	17
N5	7	14	7	10	30
N6	15	45	24	33	94
N7	12	31	19	17	29
N8	20	52	30	26	103
N9	5	20	6	15	37
N10	1	1	1	1	0
N11	1	2	1	2	0

#repr—number of representatives (= number of clusters with a size greater than one); cd—CD-HIT-EST; sum—SUMACLUST; vse—VSEARCH; mmseq—MMSeqs2; hdb—HDBSCAN.

Partial genomes are reliably placed at the correct positions within phylogenetic trees, demonstrating the robustness of our approach even when sequences are incomplete. Reverse-complement sequences, however, present a more disruptive challenge. Comprising 19.22% of the segment 4 dataset and 11.37% of the segment 6 dataset, they arrange in a characteristic “mirror-like” configuration in the phylogenetic tree. Such a pattern, if undetected, would cause clustering algorithms to treat forward and reverse-complement copies of the same sequence as unrelated, artificially inflating cluster counts and distorting representative selection. Therefore, we implemented ViralClust to detect reverse-complement sequences and to automatically restore their original orientation, simplifying downstream analysis and ensuring that strand orientation artifacts do not propagate into downstream analyses. o quantify the impact of reverse-complement sequences on clustering results, we compared 3 scenarios for segments 4 (H) and 6 (N): clustering the full dataset including reverse-complement sequences, removing reverse-complement representatives post-hoc from the original results, and re-clustering after removing all reverse-complement sequences from the input (see Supplementary Table S6). The presence of reverse-complement sequences substantially inflated the number of representatives across all tools, demonstrating that a considerable fraction of clusters in the uncorrected results arose purely from strand orientation artifacts rather than genuine sequence diversity. Notably, post-hoc removal of reverse-complement representatives and full re-clustering produced comparable results for most tools, suggesting that ViralClust’s automatic orientation correction prior to clustering is sufficient to avoid these artifacts without requiring a separate filtering step.

Given the variable results across tools and segments, we adjusted parameter ranges of ViralClust accordingly, with sequence similarity threshold between 70 and 90%; see Supplementary Table S7. CD-HIT-EST only allows for a minimum of 80%. In the case of HDBSCAN, we tested *k*-mers from 1 to 7; see Supplementary Table S8. The choice of sequence similarity threshold directly reflects the biological question: a higher threshold (e.g., 90%) retains finer sequence-level differences and is appropriate when the goal is to collapse near-identical sequences while preserving all circulating variants within a subtype, such as for outbreak subsampling. A lower threshold (e.g., 70%) merges more divergent sequences into the same cluster and is better suited for broader comparative analyses, such as selecting one representative per subtype or lineage across a diverse dataset. For *Alphainfluenzavirus influenzae*, where 18 H and 11 N subtypes span considerable sequence diversity, the threshold choice determines whether the resulting representative set captures subtype-level or strain-level diversity. As expected, the number of clusters decreases with decreasing similarity threshold for CD-HIT-EST, SUMACLUST, and VSEARCH (see Fig. 8 and Supplementary Fig. S23), confirming that these tools respond predictably to threshold changes and offer users intuitive control over clustering granularity. This predictable behavior makes threshold selection straightforward for CD-HIT-EST, SUMACLUST, and VSEARCH: users can inspect the resulting cluster counts at different thresholds, as reported in Supplementary Table S7, and select the threshold that best matches the expected number of biological units for their dataset. MMSeqs2 using the default module implemented in ViralClust, however, shows an atypical pattern: cluster counts remain roughly stable or occasionally increase at lower thresholds. We hypothesize that this behavior stems from the easy-linclust mode used within ViralClust, which is optimized for speed and may not handle the full combinatorial space of pairwise comparisons in datasets of this scale, leading to threshold-insensitive results, despite the fact that this module is described as being designed to handle large datasets. Users working with similarly large virus datasets should be aware of this limitation and consider validating MMSeqs2 results against those of other tools. Moreover, users can choose the easy-cluster module in ViralClust instead (--mmseqs_mode cluster). Among the tools evaluated, CD-HIT-EST and VSEARCH offer the most predictable and computationally tractable behavior for large segmented virus datasets, while HDBSCAN provides the finest resolution but at the cost of potentially missing rare subtypes. SUMACLUST and MMSeqs2 occupy intermediate positions, with MMSeqs2 easy-linclust requiring additional caution at large dataset sizes.

#### *Monkeypox virus* as DNA virus example

To assess the challenges posed by long, repetitive DNA genomes to clustering algorithms, we downloaded 1,077 *Monkeypox virus* genomes (family *Poxviridae*; see Supplementary Fig. S24), with an average length of 197.185 kbp. All sequences passed the max_ambiguous parameter with a default allowance of 10% for non-ACGU characters. More details about the quality of the *Monkeypox virus* dataset regarding non-ambiguous characters under different parameter settings; see Supplementary Table S9. 92.76% of the sequences are non-redundant. The main challenge compared to the RNA virus datasets analyzed previously is the combination of much longer genome length and repetitive regions, which can affect both clustering performance and representative selection.

For VSEARCH and CD-HIT-EST, we increased the parameters to accommodate the longer *Monkeypox virus* genomes: --maxseqlength 300000 (default: 50,000) and -M 2500 (default: 400). This dataset required the most time and memory overall. Based on the 2 known genotypes (clades I and II), a biologically meaningful clustering would be expected to produce 2 or more clusters capturing this known diversity. SUMACLUST, HDBSCAN, and VSEARCH produced between 1 and 3 clusters, whereas CD-HIT-EST (14) and MMSeqs2 (23) generated substantially more; see Table 1. The behavior of SUMACLUST is particularly noteworthy: approximately 66% of sequences (662 genomes) were discarded during clustering due to non-ACGU characters in repetitive genomic regions. This behavior causes the results from SUMACLUST to be unusable for subsequent analyses, since the cluster representatives cannot serve as representatives for the original dataset. This additional filtering is embedded in SUMACLUST, so the global filtering applied by ViralClust cannot affect it here. Although CD-HIT-EST generated more clusters, it assigned 917 sequences to a single cluster. In contrast, HDBSCAN and VSEARCH produced the largest clusters containing 566 and 565 sequences, respectively. HDBSCAN successfully assigned all sequences to clusters, while the other algorithms left between 1 and 69 sequences unclustered (SUMACLUST and VSEARCH: 1, CD-HIT-EST: 10, MMSeqs2: 69). CD-HIT-EST and MMSeqs2 selected more representatives than the other tools. This difference likely reflects the underlying algorithmic principles: CD-HIT-EST uses a greedy incremental clustering approach that tends to select fewer representatives by aggressively merging similar sequences, whereas MMSeqs2 implements a more sensitive, iterative clustering procedure that preserves more distinct representatives, especially in large, repetitive genomes. Whether finer granularity is desirable depends on the research question. For genotype- or subtype-level analyses, the higher resolution of CD-HIT-EST or MMSeqs2 may be advantageous, while the compact output of HDBSCAN or VSEARCH may be preferable when the goal is maximal dataset reduction. The majority of the *Monkeypox virus* dataset consists of genotype IIb (see Fig. 10). The representatives selected by SUMACLUST and HDBSCAN reflect this dominant genotype, whereas VSEARCH representatives cover genotypes Ia and IIb. Both CD-HIT-EST and MMSeqs2 resulted in representatives covering all genotypes present in the dataset. This result highlights a broader principle: tools that produce more clusters are not inherently better or worse, but in outbreak-biased datasets dominated by a single genotype, higher cluster granularity may be necessary to ensure that minority lineages are retained in the representative set. For the *Monkeypox virus* dataset, VSEARCH offers the most complete genotypic coverage while preserving a number of clusters close to the number of genotypes. CD-HIT-EST and MMSeqs2 therefore offer a finer resolution, which may be because of their higher sensitivity to sequence-level variation in repetitive genomic regions.

**Figure 10:**
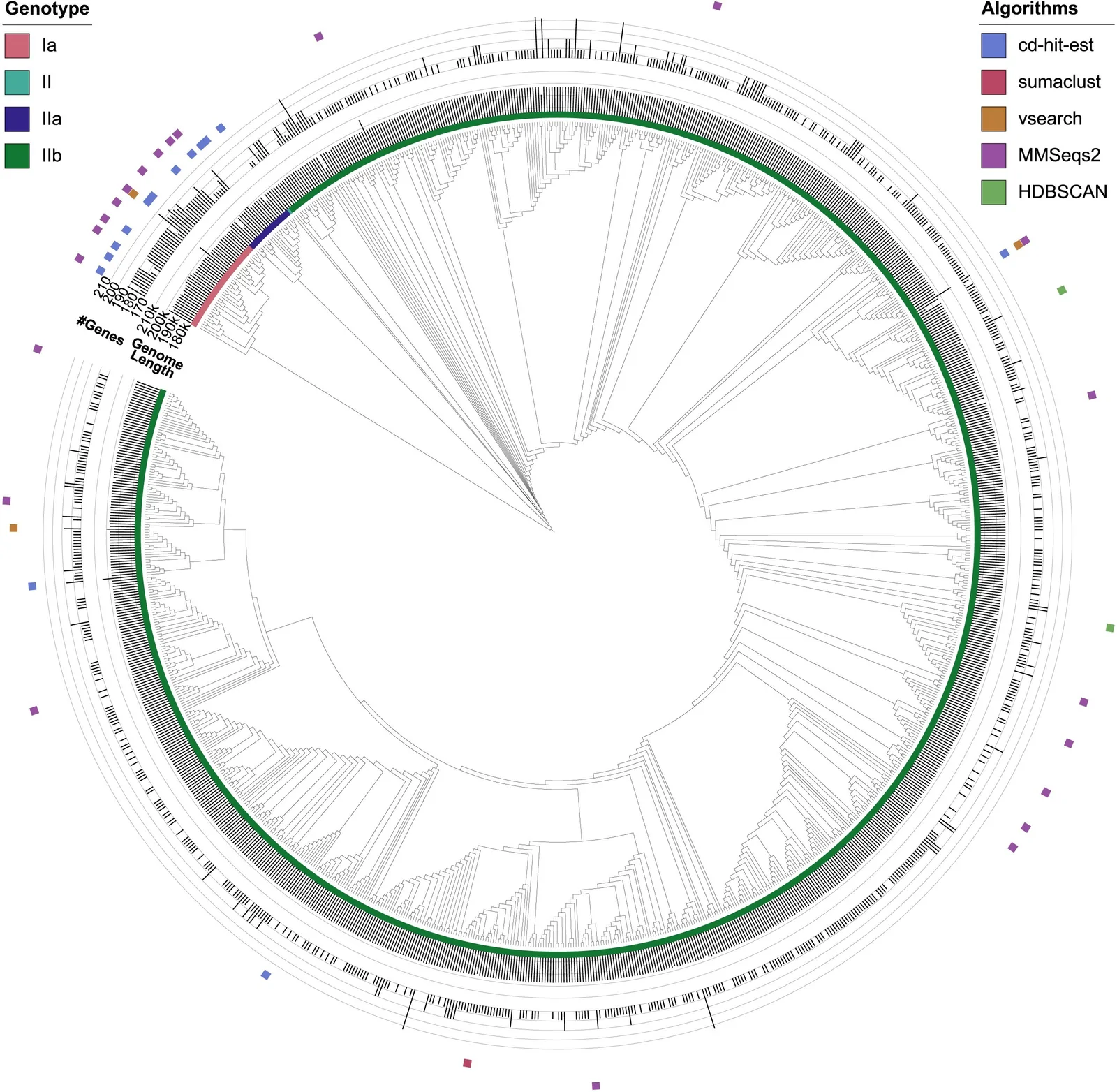
Phylogenetic reconstruction of the *Monkeypox virus* dataset with (i) genotypes, (ii) genome length, (iii) number of genes, and (iv) representative sequences selected by CD-HIT-EST, SUMACLUST, VSEARCH, MMSeqs2, and HDBSCAN. The tree is reconstructed using parsnp based on the complete genome sequences (999 genomes) resulting from the first filtering steps of ViralClust.

## Discussion

The rapid increase in publicly available viral genome sequences poses substantial challenges for downstream analyses, including MSA, phylogenetic inference, and comparative genomics. Large and unevenly sampled datasets are often computationally infeasible to analyze in full—particularly for analyses such as MSA, phylogenetic inference, and comparative genomics—and are prone to biases introduced by outbreak-driven over-representation and technical inconsistencies. In this study, we address these challenges by presenting ViralClust, a modular pipeline designed to reduce large viral genome collections through clustering-based representative selection while preserving genetic diversity.

A key outcome of our analyses is that clustering-based reduction provides a robust alternative to random subsampling. Across 6 diverse RNA and DNA virus datasets, representative genome sets retained biologically meaningful diversity while reducing dataset size by 95% on average. Importantly, clustering results were stable across taxonomic scales: representative sets generated at the family level still captured genotypes defined at the species level, as demonstrated for *Hepacivirus hominis* and members of the *Flaviviridae*. This observation indicates that representative selection does not necessarily obscure finer-scale evolutionary structure and can support comparative analyses across broad taxonomic ranges.

While the present evaluation focused on the preservation of phylogenetic topology and genetic diversity, the utility of representative selection extends to biologically relevant metadata such as host range, geographic distribution, temporal dynamics, and phenotypic characteristics. To the extent that such traits are reflected in genome sequence—as is the case, for example, for host-associated sequence signatures—clustering-based representative selection inherently captures this variation alongside genetic diversity. More broadly, the degree to which a representative set covers features of interest depends on the underlying research question, and we encourage users to evaluate their representative sets accordingly. As an example, we have previously shown in the context of pestiviruses [54] that representatives selected by clustering adequately covered the host range present in the full dataset, supporting the biological relevance of the reduced sequence set beyond phylogenetic structure alone.

The evaluation further shows that clustering whole viral genomes is feasible across a wide range of genome sizes and architectures. Even large DNA virus genomes, such as those of *Monkeypox virus*, could be processed efficiently, demonstrating the scalability of the approach. In addition, dedicated preprocessing steps proved essential for obtaining interpretable clustering results. These findings emphasize that representative selection is not solely dependent on the clustering algorithm itself but also on careful data preparation and evaluation.

We note that recombination can influence clustering outcomes by generating conflicting similarity patterns across genomic regions, potentially leading to context-dependent results. This limitation is not specific to ViralClust but affects similarity- and distance-based clustering approaches in general. The extent of this effect depends on the biological question, taxonomic scale, and analyzed genomic regions. Therefore, for highly recombinant viruses, preprocessing steps such as recombination detection, masking, or restriction to conserved regions may be advisable prior to clustering, and clustering results should always be interpreted in their biological context.

Rather than identifying a single optimal clustering strategy, ViralClust integrates multiple clustering algorithms based on different underlying principles, including compactness and connectedness. This design reflects the absence of a universal ground truth for (viral) clustering and acknowledges that different biological questions may require different clustering assumptions. By enabling direct comparison of clustering outcomes within a unified framework, ViralClust supports informed methodological choices without prescribing a “best” solution. Our framework is intentionally developed as an open collection of distinct computational mechanics rather than a rigid automated pipeline. Due to the biological variation inherent across distinct viral groups, a single guided analysis template or parameter recommendation list would oversimplify or obscure meaningful biological realities. Instead, by emphasizing case studies detailing the practical trade-offs, strengths, and limitations of these diverse algorithms, ViralClust provides users with a realistic and rigorous baseline to empirically select clustering configurations aligned with their specific biological questions. Through its modular design and integrated evaluation, ViralClust provides a flexible framework for virology research and serves as a reproducible starting point for downstream analyses of increasingly large viral genome collections. For different biological questions, other parameters of the integrated clustering tools might be tested further.

## Potential implications

Representative genome sets generated by ViralClust may facilitate a wide range of downstream analyses that are currently limited by dataset size or redundancy. For example, clustering-based representative selection can support the construction of high-quality MSAs and enable RNA secondary structure prediction using representative alignments [54–56] as employed in Rfam [57], and large-scale analyses of endogenous viral elements [58, 59]. Similarly, workflows for virus–host prediction and comparative framework analyses, such as VIDHOP [60] and POSEIDON [61], may benefit from reduced yet diverse input datasets that remain biologically representative.

More broadly, whole-genome clustering may complement gene-centric approaches commonly used in viral comparative studies. While this work does not aim to redefine virus classification, our results suggest that genome-wide similarity patterns can provide additional context for studying viral diversity across taxonomic levels. We strongly advise that clustering-based representative selection be considered as a standard preprocessing step in future viral genome analyses, enabling more balanced, interpretable, and computationally tractable studies while remaining adaptable to specific research questions and analytical goals.

## Methods

### Tools integrated into ViralClust

We used existing tools for several steps in the ViralClust pipeline we designed.

#### Preprocessing

To remove 100% redundant genomes in the input dataset, we apply the easy-linclust module of MMSeqs2 (v14) [17, 18], with parameters --min-seq-id 1.0.

#### Clustering

Per default, ViralClust applies 5 different clustering methods: CD-HIT-EST (v4.8.1) [14], SUMACLUST (v1.0.31) [15], VSEARCH (v2.15) [16] from vsearch, MMSeqs2 (v14) [17, 18], and HDBSCAN (v0.8.26) [23]. By default, ViralClust instructs CD-HIT-EST, SUMACLUST, VSEARCH, and MMSeqs2 to cluster sequences with an identity of at least 90% (default value of CD-HIT-EST). Since MMSeqs2 offers a variety of clustering modules, we have integrated easy-linclust (default) and easy-cluster.

#### Connectedness clustering

We have implemented a combination of dimension reduction and clustering in ViralClust. For this, we used PCA or UMAP (umap-learn v0.4.4) [62], both implemented in scikit-learn (v1.2.2) [31] for dimension reduction and HDBSCAN for clustering [23]. By default, we use PCA and reduce the dimension to either *d=50* or until the sum of the explained variance of the components is at least 0.7. When using UMAP, the dimensions are reduced to *d=50* with a minimum distance of 0.25, cosine distance as common usage in the context of embeddings and language [63], and random seed state of 42 as used in UMAP’s documentation, to ensure reproducibility within this stochastic algorithm [62]. HDBSCAN can be seen as a hybrid approach of hierarchical-based (connectivity) and density-based (compactness) clustering approaches. For 2 vectors, HDBSCAN first calculates mutual reachability distance. By default, we use the cosine distance implemented in SciPy (v1.4.1) [32] as metric. However, we allow a plethora of pre-defined distances, which can be used via the --metric parameter of our HDBSCAN module. We implemented the (standardized) cosine (=default), Euclidean, Manhattan, Chebyshev, Minkowski, Canberra, Bray–Curtis, and Mahalanobis distance metrics via the SciPy library [32]. To accommodate the limitations of the current implementation of HDBSCAN, we have employed L2 normalization to preprocess the vectors. This ensures compatibility with distance calculations, as cosine distance is not supported. Subsequently, we use HDBSCAN with the Euclidean distance on these normalized vectors as recommended [33].

### Visualization

Importantly, with ViralClust, our aim is not to construct a phylogeny of the viruses, but rather to ensure that the selected virus genomes are distributed evenly across the existing phylogenetic reconstruction, providing a rough overview of their diversity. Therefore, for the validation of our use cases, we additionally visualized the distribution of cluster representatives and NCBI RefSeq genomes across the phylogenetic representation with the Neighbor-net algorithm implemented in SplitsTree (v4.19.2) [64, 65], generating split graphs, FastTree (v2.1.10) [36] as time-efficient tool for phylogenetic reconstruction of large datasets, and parsnp (v2.1.5) [66] for core-genome-based phylogenetic reconstruction (not part of the pipeline for the original data).

### Benchmarking and scoring metrics

All ViralClust runs were conducted on a Linux-based x86_64 server equipped with 4 AMD Opteron 6376 processors comprising 64 logical CPU cores in total (8 cores per socket with 2 threads per core) and a maximum clock frequency of 2.3 GHz. The system architecture featured 8 NUMA nodes and supported AMD-V virtualization. Computational tasks were executed in a shared-memory environment using the available multi-core CPU resources.

ViralClust offers a first evaluation based on several statistics using the parameter --eval. To evaluate the performance of the 5 tools integrated into ViralClust across all 6 datasets presented here, we incorporated additional metrics determining the quality of the clustering output, summarized into one overall score. All scores are defined in the range *[0, 1]*, where higher values indicate better performance, and the overall score has a maximum of 5.

#### Reduction score

The RS measures how much a given tool reduces the input dataset through clustering, while accounting for sequences discarded by the clustering algorithm:


(1) \begin{eqnarray*}
\mathrm{ RS} = \left(1 - \frac{\#\mathrm{cluster}}{\#\mathrm{sequences}}\right) \cdot \frac{\#\mathrm{used\_sequences}}{\#\mathrm{sequences}}.
\end{eqnarray*}

The first factor captures the proportion of sequences replaced by cluster representatives, while the second factor penalizes tools that discard a large fraction of input sequences rather than assigning them to clusters. A score of 1 indicates maximal reduction with no sequence loss.

#### Singleton penalty score

The SPS penalizes algorithms that leave many sequences unclustered, i.e., assigned to singleton clusters of size one:


(2) \begin{eqnarray*}
\mathrm{ SPS} = 1 - \frac{\#\mathrm{unclustered}}{\#\mathrm{sequences}}.
\end{eqnarray*}

Singletons do not contribute to dataset reduction and may indicate over-fragmentation or, in the case of density-based methods such as HDBSCAN, sequences assigned to noise. A score of 1 indicates that no sequences were left unclustered.

#### Clustering quality score

The CQS measures the separation between clusters relative to their internal cohesion, based on pairwise phylogenetic distances derived from a user-provided tree and the cluster assignment file of each tool:


(3) \begin{eqnarray*}
\mathrm{ CQS }= 1 - \frac{\varnothing _{\mathrm{intra}}}{\varnothing _{\mathrm{intra}} + \varnothing _{\mathrm{inter}}},
\end{eqnarray*}

where $\varnothing _{\mathrm{intra}}$ and $\varnothing _{\mathrm{inter}}$ denote the average intra- and inter-cluster distances on the tree, respectively. A score close to 1 indicates well-separated clusters with low internal distances, whereas a score close to 0 indicates poor cluster separation. The CQS requires a Newick-formatted phylogenetic tree and the .clstr output file of each tool as input provided by ViralClust.

#### Taxonomic concordance score

The TCS measures how well the clustering reflects known taxonomy, based on NCBI taxonomic assignments of the input sequences:


(4) \begin{eqnarray*}
\mathrm{ TCS} = \frac{1}{\varnothing _{\text{cluster per species}}},
\end{eqnarray*}

where $\varnothing _{\text{cluster per species}}$ is the average number of clusters a single species is spread across. A score of 1 indicates that each species is entirely contained within a single cluster, with no fragmentation across cluster boundaries. Values below 1 indicate that species are split across multiple clusters. Note that TCS is sensitive to the completeness of NCBI taxonomic annotations and may be less reliable for novel or poorly characterized viruses.

#### Cluster-to-taxonomy ratio

The CTR evaluates how closely the number of clusters produced by a tool matches the number of species defined by the ICTV:


(5) \begin{eqnarray*}
\mathrm{ CTR} = 1 - \left|1 - \frac{\#\mathrm{cluster}}{\#\text{ICTV species}}\right|.
\end{eqnarray*}

A score of 1 indicates that the number of clusters exactly matches the expected number of ICTV-defined species. Scores decrease symmetrically for both over- and under-clustering relative to this expectation, and are clamped to [0, 1].

#### Overall score

The OS aggregates all 5 metrics into a single summary value:


(6) \begin{eqnarray*}
\mathrm{ OS} = \mathrm{ RS} + \mathrm{ SPS} + \mathrm{ CQS} + \mathrm{ TCS} + \mathrm{ CTR}.
\end{eqnarray*}

All 5 component scores contribute equally, each with a maximum of 1, giving an overall maximum of 5. A higher OS indicates a clustering result that simultaneously achieves strong dataset reduction, low singleton rate, good phylogenetic cluster separation, and concordance with known taxonomy at both the sequence annotation and ICTV species level.

## Availability of source code and requirements

- Project name: ViralClust
- Project home page: https://github.com/rnajena/viralclust
- License: GPL-3.0 license
- Operating system(s): Linux
- Programming language: Nextflow, Python
- Other requirements: Anaconda

## Additional files

**Supplementary Figure S1**: Phylogenetic tree of *Filoviridae* (ICTV) including *Orthoebolavirus* (cyan).

**Supplementary Figure S2**: (A) Phylogenetic representation of ebolaviruses retrieved from the NCBI RefSeq database. The tree is based on the complete nucleotide sequence as described in the literature [67, 68]. (B–F) Phylogenetic representation of all representative ebolaviruses determined with CD-HIT-EST, SUMACLUST, VSEARCH, MMSeqs2, and HDBSCAN, respectively. Genomes derived from recombination or reverse genetics experiments are labeled with a triangle. Multiple sequence alignment and tree were calculated with MAFFT and FastTree, respectively.

**Supplementary Figure S3**: Phylogenetic representation of the *Orthoebolavirus* dataset. The tree is reconstructed using RAxML-NG based on the MAFFT alignment of the complete genome sequences (443 genomes) resulting from the first filtering steps of ViralClust. For detailed listing of parameters, we refer to the “Methods” section.

The inner track around the tree represents the species information as retrieved from the NCBI database, serving as the “ground truth.” Empty rectangles mark genomes for which no species information is available in the metadata. The top 5 tracks show the resulting clusters from CD-HIT-EST, SUMACLUST, VSEARCH, MMSeqs2, and HDBSCAN, respectively. Colors indicate the individual clusters identified by each tool and highlighting their level of agreement. We present singletons with empty rectangles.

**Supplementary Figure S4**: Phylogenetic tree of the family *Flaviviridae* (ICTV), including genus *Orthoflavivirus* (violet background) and species *Hepacivirus hominis* (HCV, yellow background). *Flaviviridae* consists of 4 genera, including 97 species; *Orthoflavivirus* comprises 53 species; and *Hepacivirus hominis* includes 8 genotypes (only 7 genotypes are displayed in the tree). This tree is based on the partial gene sequences between positions 8,040 and 8,897 (numbered using positions in the HCV sequence, AF011751).

**Supplementary Figure S5**: Split graph of *Orthoflavivirus* with highlighted cluster representatives of CD-HIT-EST, SUMACLUST, VSEARCH, MMSeqs2, and HDBSCAN. The split graph is reconstructed using SplitsTree based on the MAFFT alignment of the complete genome sequences (7,681 genomes) resulting from the first filtering steps of ViralClust.

**Supplementary Figure S6**: Phylogenetic representation of *Orthoflavivirus* showing vectors (mosquito-borne, tick-borne, insect-only, and unknown). The tree is reconstructed using FastTree based on the MAFFT alignment of the complete genome sequences (7,681 genomes) resulting from the first filtering steps of ViralClust. More details in Fig. 5.

**Supplementary Figure S7**: Phylogenetic representation of *Flaviviridae* with labeled genera: *Orthoflavivirus, Pestivirus, Hepacivirus*, and *Pegivirus*. The tree is reconstructed using FastTree based on the MAFFT alignment of the complete genome sequences (9,478 genomes) resulting from the first filtering steps of ViralClust. More details in Fig. 6.

**Supplementary Figure S8**: Phylogenetic representation of HCV showing the cluster representatives of MMSeqs2 and HDBSCAN since the results show differences in the clustering of HCV only and HCV clustered together with all other *Flaviviridae*. The tree is reconstructed using FastTree based on the MAFFT alignment of the complete genome sequences (1,071 genomes) resulting from the first filtering steps of ViralClust.

**Supplementary Figure S9**: Phylogenetic representation of *Orthoflavivirus* showing the cluster representatives and labeled vectors (mosquito-borne, tick-borne, unknown vector, and insect-only). The clustering of the *Orthoflavivirus* only and the *Orthoflavivirus* among the entire *Flaviviridae* dataset showed differences in the results of CD-HIT-EST, MMSeqs2, and HDBSCAN. The tree is reconstructed using FastTree based on the MAFFT alignment of the complete genome sequences (7,681 genomes) resulting from the first filtering steps of ViralClust.

**Supplementary Figure S10**: Phylogenetic tree of *Orthomyxoviridae* (ICTV) including IAV.

**Supplementary Figure S11**: Phylogenetic tree of IAV segment 4 (H) with highlighted subtypes H1–H18. Gray area marks genomes with partial reverse complement CDS. That subtree “mirrors” the rest of the tree. The tree is reconstructed using FastTree based on the MAFFT alignment of the complete genome sequences (108,514 genomes) resulting from the first filtering steps of ViralClust.

**Supplementary Figure S12**: Phylogenetic tree of IAV segment 6 (N) with highlighted subtypes N1–N11. Gray area marks genomes with partial reverse complement CDS. That subtree “mirrors” the rest of the tree. The tree is reconstructed using FastTree based on the MAFFT alignment of the complete genome sequences (73,786 genomes) resulting from the first filtering steps of ViralClust.

**Supplementary Figure S13**: Split graphs of IAV segment 4 (H) representatives of (A) CD-HIT-EST. Left—all representatives (including partial CDS); Right—only complete genomes. The node size indicates the cluster size. Subtypes (H1–H18) are color-coded. The split graph is reconstructed using SplitsTree based on the MAFFT alignment of the complete genome sequences (108,514 genomes) resulting from the first filtering steps of ViralClust.

**Supplementary Figure S14**: Split graphs of IAV segment 4 (H) representatives of (B) SUMACLUST. Left—all representatives (including partial CDS); Right—only complete genomes. The node size indicates the cluster size. Subtypes (H1–H18) are color-coded. The split graph is reconstructed using SplitsTree based on the MAFFT alignment of the complete genome sequences (108,514 genomes) resulting from the first filtering steps of ViralClust.

**Supplementary Figure S15**: Split graphs of IAV segment 4 (H) representatives of (C) VSEARCH. Left—all representatives (including partial CDS); Right—only complete genomes. The node size indicates the cluster size. Subtypes (H1–H18) are color-coded. The split graph is reconstructed using SplitsTree based on the MAFFT alignment of the complete genome sequences (108,514 genomes) resulting from the first filtering steps of ViralClust.

**Supplementary Figure S16**: Split graphs of IAV segment 4 (H) representatives of (D) MMSeqs2. Left—all representatives (including partial CDS); Right—only complete genomes. The node size indicates the cluster size. Subtypes (H1–H18) are color-coded. The split graph is reconstructed using SplitsTree based on the MAFFT alignment of the complete genome sequences (108,514 genomes) resulting from the first filtering steps of ViralClust. HDBSCAN did not select any representatives for subtypes H17 and H18. We added these manually to the alignment for split graph reconstruction and marked them as framed circles.

**Supplementary Figure S17**: Split graphs of IAV segment 4 (H) representatives of (E) HDBSCAN. Left—all representatives (including partial CDS); Right—only complete genomes. The node size indicates the cluster size. Subtypes (H1–H18) are color-coded. The split graph is reconstructed using SplitsTree based on the MAFFT alignment of the complete genome sequences (108,514 genomes) resulting from the first filtering steps of ViralClust.

**Supplementary Figure S18**: Split graphs of IAV segment 6 (N) representatives of (A) CD-HIT-EST. Left—all representatives (including partial CDS); Right—only complete genomes. The node size indicates the cluster size. Subtypes (N1–N11) are color-coded. The split graph is reconstructed using SplitsTree based on the MAFFT alignment of the complete genome sequences (108,514 genomes) resulting from the first filtering steps of ViralClust.

**Supplementary Figure S19**: Split graphs of IAV segment 6 (N) representatives of (B) SUMACLUST. Left—all representatives (including partial CDS); Right—only complete genomes. The node size indicates the cluster size. Subtypes (N1–N11) are color-coded. The split graph is reconstructed using SplitsTree based on the MAFFT alignment of the complete genome sequences (108,514 genomes) resulting from the first filtering steps of ViralClust.

**Supplementary Figure S20**: Split graphs of IAV segment 6 (N) representatives of (C) VSEARCH. Left—all representatives (including partial CDS); Right—only complete genomes. The node size indicates the cluster size. Subtypes (N1–N11) are color-coded. The split graph is reconstructed using SplitsTree based on the MAFFT alignment of the complete genome sequences (108,514 genomes) resulting from the first filtering steps of ViralClust.

**Supplementary Figure S21**: Split graphs of IAV segment 6 (N) representatives of (D) MMSeqs2. Left—all representatives (including partial CDS); Right—only complete genomes. The node size indicates the cluster size. Subtypes (N1–N11) are color-coded. The split graph is reconstructed using SplitsTree based on the MAFFT alignment of the complete genome sequences (108,514 genomes) resulting from the first filtering steps of ViralClust.

**Supplementary Figure S22**: Split graphs of IAV segment 6 (N) representatives of (E) HDBSCAN. Left—all representatives (including partial CDS); Right—only complete genomes. The node size indicates the cluster size. Subtypes (N1–N11) are color-coded. The split graph is reconstructed using SplitsTree based on the MAFFT alignment of the complete genome sequences (108,514 genomes) resulting from the first filtering steps of ViralClust. HDBSCAN did not select any representatives for subtypes N10 and N11. We added these manually to the alignment for split graph reconstruction and marked them as framed circles.

**Supplementary Figure S23**: Number of clusters of IAV segments 1, 2, 4, 5, 7, and 8 with sequence similarity thresholds from 70 to 90%. CD-HIT-EST only allows a minimum of 80%. Only clusters with a size greater than one are considered.

**Supplementary Figure S24**: Phylogenetic tree of *Orthopoxvirus* (ICTV) including *Monkeypox virus*.

**Supplementary Table S1**: Run time and memory usage of ViralClust steps measured by Nextflow. cd—CD-HIT-EST; sum –SUMACLUST; vse—VSEARCH; mmseq—MMSeqs2; hdb—HDBSCAN; #Sequences—number of input genomes/segments with accession date; only records labeled as “complete”; rem.-red.—remove redundancy; *∅* aln—average time/memory of alignment construction of representative genomes using MAFFT; *∅* tree—average time/memory of phylogenetic tree reconstruction of representative genomes using FastTree; eval—average time/memory of calculating basic cluster statistics; Peak RAM—peak RSS (resident set size) of ViralClust measured by Nextflow.

**Supplementary Table S2**: “Ground truth” of virus phylogeny. Number of genera, subgenera, species, subtypes, and/or genotypes according to ICTV, NCBI, and CDC. g—genera; sg—subgenera; s—species; st—subtypes; gt–genotypes; *— https://www.cdc.gov/flu/about/viruses-types.html. Last reviewed: 18.09.2024.

**Supplementary Table S3**: Scoring metric sorted by dataset. RS—reduction score; SPS—singleton penalty score; CQS—clustering quality score; TCS—taxonomic concordance score (based on NCBI species); overall score—weighted sum of all scores above; IAV—*Alphainfluenzavirus influenzae*; cd—CD-HIT-EST; sum—SUMACLUST; vse—VSEARCH; mmseq—MMSeqs2; hdb—HDBSCAN.

**Supplementary Table S4**: Comparing the clustering results of the *Orthoflavivirus* dataset with the ICTV. *\checkmark*—cluster with 1 ICTV representative; *\emptyset*—number of clusters without ICTV representatives; Problems—cluster with *> =2* ICTV representatives.

**Supplementary Table S5**: *Orthoflavivirus* species, their ICTV representatives and NCBI RefSeqs, and the cluster number in which they were clustered. GenBank—GenBank accession ID; NCBI RefSeq—NCBI RefSeq accession ID; ICTV—category in the ICTV (E–exemplar genome, A—additional genome); Nucl Comp—Nucleotide completeness (C—Complete, CC—Coding-complete P—Partial); dataset—presence of the genome in our *Orthoflavivirus* dataset (G—GenBank in our dataset, N—NCBI RefSeq in our dataset); cd—CD-HIT-EST; sum—SUMACLUST; vse—VSEARCH; mmseq—MMSeqs2; hdb—HDBSCAN.

**Supplementary Table S6**: Clustering IAV with and without (truncated) reverse-complement sequences. Dataset—dataset used as input; rc rep rem—reverse-complement representatives were removed from the representative dataset and used as new results; rc rem—reverse-complement sequences were removed from the non-redundant dataset and used as input for a new clustering run; #Seq—number of input genomes for clustering with ViralClust; Number of genomes after clustering—only considering clusters with a size greater than one; cd—CD-HIT-EST; sum—SUMACLUST; vse—VSEARCH; mmseq—MMSeqs2; hdb—HDBSCAN.

**Supplementary Table S7**: Overview of IAV clustering with HDBSCAN and *k*-mer length 1–7. Seg–segment; Number of genomes after clustering—only considering clusters with a size greater than one.

**Supplementary Table S8**: Overview of IAV clustering with HDBSCAN and *k*-mer length 1–7. Seg–segment; Number of genomes after clustering—only considering clusters with a size greater than one.

**Supplementary Table S9**: Number of sequences of the *Monkeypox virus* dataset passing the filtering of non-ACGU characters (--max_ambiguous) with several parameter settings. The default setting is highlighted in bold.

## List of abbreviations

BDBV: *O. bundibugyoense* (Bundibugyo); BOMV: *O. bombaliense* (Bombali); cd: CD-HIT-EST; CDC: Centers for Disease Control and Prevention; CQS: clustering quality score; CTR: cluster-to-taxonomy ratio; EBOV: *O. zairense* (Ebola); GOI: **g**enomes **o**f **i**nterest; HCV: hepatitis C virus; HDB: hierarchical density-based clustering; hdb: HDBSCAN; IAV: *Alphainfluenzavirus influenzae* (formerly *Influenza A virus*); ICTV: International Committee on Taxonomy of Viruses; mmseq: MMSeqs2; MSA: multiple sequence alignment; NCBI: National Center for Biotechnology Information; ORF: open reading frame; OS: overall score; PCA: principal component analysis; RESTV: *O. restonense* (Reston); RS: reduction score; SPS: singleton penalty score; ssRNA: single-stranded RNA; SUDV: *O. sudanense* (Sudan); suma: SUMACLUST; TAFV: *O. taiense* (Taï Forest); TCS: taxonomic concordance score; UMAP: Uniform Manifold Approximation and Projection; vse: VSEARCH.

## Supplementary Material

giag090_Supplemental_File

giag090_Authors_Response_To_Reviewer_Comments_original_submission

giag090_Authors_Response_To_Reviewer_Comments_revision_1

giag090_GIGA-D-26-00034_original_submission

giag090_GIGA-D-26-00034_Revision_1

giag090_GIGA-D-26-00034_Revision_2

giag090_Reviewer_1_Report_original_submission Reviewer 1 -- 2/11/2026

giag090_Reviewer_1_Report_Revision_1 Reviewer 1 -- 6/16/2026

giag090_Reviewer_1_Report_Revision_2 Reviewer 1 -- 8/6/2026

giag090_Reviewer_2_Report_original_submission Reviewer 2 -- 3/4/2026

giag090_Reviewer_2_Report_Revision_1 Reviewer 2 -- 6/4/2026

## Data Availability

The raw sequences and datasets supporting the results of this article are available in the Zenodo repository [24].
