# Supplementary material for: Reducing haystacks to needles—ViralClust: a Nextflow pipeline to cluster viral sequences
Source: Gigascience. 2026 Sep 14;15:giag090. doi: 10.1093/gigascience/giag090 (PMC13660479; doi:10.1093/gigascience/giag090)
Supplement: Supplementary file 1 [file giag090_authors_response_to_reviewer_comments_original_submission.pdf]

Author's Response To Reviewer Comments

Dear Editor and Reviewers,

We would like to express our gratitude for your thoughtful and constructive comments on our paper titled "Reducing haystacks to needles - ViralClust: A Nextflow pipeline to cluster viral sequences".

In the following sections, we outline your specific comments and provide detailed explanations of the changes we have implemented to address each point. We have highlighted our answers as well as the additions in the manuscript in two colors (blue for reviewer one and magenta for reviewer two).

Reviewer 1
The authors present ViralClust, a modular Nextflow pipeline designed to address the computational challenges and sampling biases associated with the rapid accumulation of viral genome sequences. By integrating five distinct clustering algorithms (CD-HIT-EST, SUMACLUST, VSEARCH, MMSeqs2, and HDBSCAN), the pipeline offers a complete workflow from data pre-processing to representative sequence selection. The authors evaluated the tool on six viral datasets of varying size and type, including Orthoebolavirus, Hepacivirus, Influenza A, and Monkeypox virus.
This is a highly practical tool, particularly given the context of rapidly expanding viral genomic databases. The manuscript is well-structured, and the test datasets are representative, covering diverse viral characteristics. However, there is room for improvement regarding the analysis of specific algorithm performance, user guidance, and the presentation of results.

Specific Comments
1. Currently, ViralClust functions more as a "toolbox" of aggregated methods rather than a mature, user-friendly "solution." Although five clustering algorithms are provided, the manuscript lacks sufficient guidance on the specific application scenarios for each. I recommend that the authors design a comprehensive scoring metric to rank the clustering results of each algorithm, providing a reference for users. Alternatively, please provide recommended algorithms or parameter presets (profiles) tailored to different analysis scenarios (e.g., specific profiles for RNA viruses, DNA viruses, or segmented viruses). This would significantly aid users in selecting the most appropriate strategy.

We thank the reviewer for this valuable comment! We agree that guidance on tool selection is an important aspect of a user-friendly methods paper, and we have addressed this in two complementary ways.
First, we have incorporated a comprehensive scoring metric into the benchmarking of ViralClust, comprising five component scores - the Reduction Score (RS), Singleton Penalty Score (SPS), Clustering Quality Score (CQS), Taxonomic Concordance Score (TCS), and Cluster-to-Taxonomy Ratio (CTR) - aggregated into an Overall Score (OS) with a maximum of 5, shown in Table 2 and S3. These metrics are described in detail in the Methods section and applied across all six datasets presented in this study.
Second, rather than prescribing a single recommended tool, we use the scoring results together with the per-dataset observations to provide explicit guidance on the strengths and limitations of each algorithm under different biological scenarios. Specifically, based on our evaluation across RNA viruses, segmented viruses, and large DNA virus genomes, we discuss:
the tendency of compactness-based tools (CD-HIT-EST, SUMACLUST, VSEARCH) to produce fewer, more compact clusters, which is advantageous for maximal dataset reduction but may collapse rare lineages
the higher granularity of MMSeqs2, which better preserves sequence-level diversity at the cost of larger representative sets, with the caveat that its behavior becomes less predictable at very large dataset sizes
the suitability of HDBSCAN for datasets with continuous diversity distributions, while noting its fundamental limitation in detecting rare subtypes represented by few sequences, which may be missed as noise.
We believe this approach is more informative than fixed parameter presets, as the appropriate clustering strategy depends critically on the biological question, taxonomic scope, and dataset composition - all of which vary substantially across virology research contexts. The scoring metric provides users with an objective, reproducible basis for making this choice with their own data, while the per-scenario discussion in the Results offers concrete guidance grounded in empirical evaluation.

2. The Results section is currently somewhat verbose and repetitive. As this is a Method/Tool paper, the core focus should be on demonstrating the tool's generalizability, accuracy, and the comparative logic between different algorithms, rather than a monotonous recitation of statistics for every dataset. Please consolidate the Results into 1-2 focused sections. Avoid listing exhaustive statistics for every case study and instead focus on summarizing trends and algorithmic differences.

We appreciate the reviewer's suggestion to streamline the Results section. However, because this is a methods/tool paper comparing clustering approaches, we believe that evaluating only overall generalizability or accuracy would not sufficiently capture the practical differences between algorithms. The performance and reliability of clustering methods strongly depend on the underlying biological question and dataset characteristics.
For this reason, we deliberately structured the Results around several representative scenarios that reflect common questions encountered in virology research. The six datasets were specifically selected to cover distinct biological and technical contexts, including: (1) a small and biased dataset, (2) analyses across different taxonomic levels (species, genus, and family), highlighting how tool behavior changes depending on context, (3) a segmented virus dataset, and (4) a large DNA virus dataset.
We therefore believe that retaining the separate sections is important to demonstrate not only overall performance trends, but also the reliability and limitations of each clustering approach under biologically relevant conditions. Nevertheless, in response to the reviewer's concern, we have revised the text to reduce repetitive reporting of statistics and instead emphasize the overarching trends and methodological differences between algorithms within each scenario.

3. The authors mention filtering out sequences containing less than 90% ACGT characters. Ambiguous bases (e.g., N, R, Y) are very common in consensus sequences, and strict filtering may be inappropriate. For instance, in the Monkeypox analysis, SUMACLUST discarded approximately 66% of the sequences, which is a significant loss of data. Consider relaxing this filter or modifying the strategy to calculate non-ACGT content only within Open Reading Frames (ORFs) to avoid discarding potentially valuable genomic data.

Thank you very much for your valuable comment and suggestion. Sequences containing more than 10% non-ACGT characters are filtered out before the actual clustering process begins. We initially opted for a fixed, high threshold to ensure higher data quality for clustering. We've integrated a parameter (--max_ambiguous) so the user can adjust this filtering process and mentioned this option in the main manuscript.
You are absolutely right. SUMACLUST imposes very strict requirements on the input sequences and allows only ACGT characters. Since this is specific to the SUMACLUST algorithm, we cannot change this within our pipeline. Nevertheless, we have integrated SUMACLUST into our ViralClust pipeline because it is a widely used clustering tool. However, we have mentioned this limitation in the manuscript to provide readers and users with all the information they need to select the clustering results appropriate for their individual research questions.

4. The authors noted that the easy-linclust module of MMSeqs2 performed poorly on the large Influenza A dataset and hypothesized that easy-linclust is unsuitable for such data. Since ViralClust is explicitly aimed at handling "large-scale" data, it is insufficient to merely report this failure. I suggest the authors attempt to integrate other MMSeqs2 clustering modules, such as the easy-cluster, to see if results improve. If an alternative module performs better, it should be implemented as an alternative option or fallback within the pipeline.

We thank the reviewer for this constructive suggestion. We would like to clarify the rationale for our original choice of easy-linclust: the MMseqs2 user guide explicitly recommends it for large datasets, stating that "For larger datasets, easy-linclust offers an efficient clustering workflow, scaling linearly with input size.". Accordingly, easy-linclust was selected as the default MMseqs2 module in ViralClust to ensure applicability on standard hardware, including laptops and workstations without high-memory compute environments.
Nevertheless, we acknowledge the reviewer's point that the unexpected behavior of easy-linclust on the

IAV dataset, where cluster counts did not decrease monotonically with lower similarity thresholds, represents a relevant limitation for large-scale datasets and warrants a practical alternative. We have therefore implemented easy-cluster as an optional alternative to easy-linclust within the ViralClust pipeline. We have briefly tested the easy-cluster module on the IAV segment 4 dataset, and based on the number of clusters, easy-cluster might be a good alternative. The easy-linclust module remains the default for computational efficiency, while easy-cluster can be selected via a pipeline parameter (--mmseqs_module cluster) for users requiring more robust clustering behavior on large datasets. This has been noted in the manuscript accordingly.

5. The evaluation reveals a critical limitation in HDBSCAN, which tends to misclassify biologically significant but low-abundance lineages as noise—evidenced by the failure to identify TAFV and rare Influenza subtypes (H17/H18, N10/N11), alongside a disproportionately high generation of singletons (e.g., 1,333 in Flaviviridae). As the current requirement to manually recover these files undermines the pipeline's automation and poses a risk of data loss, I strongly recommend implementing a parameter (e.g., --keep_singletons) to automatically include unclustered sequences in the final representative output, while also expanding the Discussion/User guidance to explicitly warn users about the trade-offs of using density-based clustering for rare variant detection.

Currently, the results are divided into three Fasta files, and therefore the request of the reviewer is already implemented:
Cluster representatives with a cluster size > 1
Cluster representatives with a cluster size = 1 (singletons)
Cases 1 and 2 are combined into a single file.
The user can choose which file to use for the next steps of their research project. We have described this file generation in the text "Analyses - Determination of representative genomes" - "Clusters of size one are therefore not considered clusters, but named "singletons" here, and are stored in a separate file. The combined set of cluster representatives and "singleton" sequences is provided in a third file. This allows the user to choose which set of sequences is most suitable for the research question." We have highlighted this section in the main manuscript.

6. Please include the maximum memory consumption (Peak RAM) for each dataset in Table 1. While runtimes are provided, memory usage is a critical factor for users handling large datasets.

Thank you for this comment. We've integrated the maximum memory consumption (Peak RAM) in Table 1 and S1 for every dataset.

Reviewer 2
Sandra Triebel et al. build a modular Nextflow pipeline—ViralClust for clustering large viral genome datasets and selecting representative sequences in a bias-aware way. The pipeline integrates five clustering tools (CD-HIT-EST, SUMACLUST, VSEARCH, MMSeqs2, and HDBSCAN), offers optional evaluation outputs (MSA + rough phylogenetic representations), and produces HTML summaries and per-tool representative sets. While the pipeline sounds a good tool to addresses a genuine need in viral genomics, several major concerns regarding validation, methodological clarity, and practical implementation need to be addressed.

Major Comments
1. Although the author demonstrated that clustering preserves the phylogenetic topology, they did not adequately verify whether the selected representative strains retained biologically relevant traits such as host range, geographic distribution, temporal dynamics, or phenotypic characteristics.

We thank the reviewer for this valuable suggestion. We agree that the biological relevance of representative selection extends beyond phylogenetic topology, and we have added a paragraph to the Discussion addressing this point.
We note that to the extent biologically relevant traits such as host range, geographic distribution, or temporal dynamics are reflected in genome sequence, clustering-based representative selection inherently captures this variation alongside genetic diversity. Furthermore, the degree to which a representative set

covers features of interest will always depend on the underlying research question, and we encourage users to evaluate their representative sets with respect to the metadata most relevant to their specific study.
To illustrate this, we point to our previously published work on Pestiviruses, in which representatives selected by clustering were shown to adequately cover the host range present in the full dataset [1]. This example supports the biological relevance of clustering-based representative selection beyond phylogenetic structure alone, and we have now made this explicit in the revised Discussion.

[1] Triebel S, Eulenfeld T, Ontiveros-Palacios N, Sweeney B, Tautz N, Marz M. First full-genome alignment representative for the genus Pestivirus. RNA. 2026 Feb 17;32(3):311-327. doi: 10.1261/rna.080732.125. PMID: 41436245; PMCID: PMC12908451.

2. The author present results under various parameter combinations but does not adequately explain how users should select appropriate parameters based on specific research questions, which can be confusing for those unfamiliar with the context. For example, when should users use a 70% rather than a 90% sequence similarity threshold? How does this choice affect different downstream analyses?

We thank the reviewer for this important suggestion. We agree that parameter selection is one of the most practically relevant aspects of a clustering tool for users without a deep computational background, and we have addressed this in the revision in several ways.
First, we want to acknowledge that parameter selection in viral genome clustering is inherently context-dependent: the appropriate sequence similarity threshold is not a fixed value but depends critically on the biological question, the taxonomic scope of the dataset, and the evolutionary diversity of the viral clade under study. A 90 % threshold may be appropriate for within-outbreak subsampling of a highly conserved virus, where the goal is to collapse near-identical sequences while retaining all circulating variants. In contrast, a 70 % threshold may be more appropriate for family-level analyses spanning multiple genera, where the aim is to retain one representative per major lineage rather than per sequence cluster. Specifying a universally applicable threshold would therefore be misleading, as it would imply a level of generalizability that does not exist.
That said, we acknowledge the reviewer's point that users need more concrete guidance than "it depends". We have therefore addressed this in two complementary ways in the revision. First, we incorporated a comprehensive scoring framework comprising five component scores (RS, SPS, CQS, TCS, CTR) aggregated into an Overall Score (OS, maximum 5), which allows users to objectively compare clustering results across parameter settings and tools for their specific dataset. This scoring framework is described in the Methods section and applied across all six datasets in this study, providing empirical reference points for parameter choices under different biological scenarios. Second, we have expanded the Results section to explicitly discuss the effect of varying similarity thresholds for each dataset, highlighting where threshold choice had a strong effect on cluster number and representative selection, and where results were robust to parameter variation.
Together, these additions provide users with both a quantitative tool for parameter evaluation and concrete biological context for interpreting their results, without prescribing thresholds that may not generalize beyond the scenarios evaluated here.

3. Recombination occurs across multiple viral families, and the authors did not adequately explain how recombinant sequences are handled during the clustering process. Depending on the genomic regions used to calculate similarity, recombinant genomes may yield divergent clustering outcomes. The authors should discuss whether their method is applicable to highly recombinant viruses and clarify whether additional preprocessing steps (such as recombination detection or removal) are necessary.

We thank the reviewer for highlighting the important issue of recombination in viral genomes. We agree that recombinant sequences can substantially influence clustering outcomes, particularly because similarity-based approaches may produce different results depending on the genomic regions analyzed.
To specifically assess this aspect, we included Deformed wing virus (DWV), a highly recombinant virus, as one of the evaluated datasets. Our analyses showed that none of the tested clustering methods produced consistently robust or biologically stable results for this dataset. This observation highlights the challenges that recombination poses for sequence similarity–based clustering approaches in general and demonstrates an important limitation of the evaluated methods.

Our framework itself does not explicitly detect or correct for recombination events prior to clustering. Instead, clustering reflects the sequence relationships present in the input data and is therefore inherently influenced by the genomic composition of recombinant viruses. Consequently, highly recombinant viruses may exhibit unstable or context-dependent clustering patterns, especially when different genomic regions are analyzed separately.
We further note that this limitation is not unique to our approach, but broadly affects most similarity- and distance-based clustering strategies. The impact of recombination strongly depends on the biological question, the taxonomic level under investigation, and whether analyses are performed on complete genomes or selected genomic regions. For viral groups with frequent recombination, additional preprocessing steps such as recombination detection, masking, or restriction to conserved genomic regions may therefore be advisable prior to clustering.
Following the reviewer's valuable suggestion, we have expanded and improved the Discussion/Conclusion section to more explicitly address the applicability and limitations of clustering approaches for highly recombinant viruses and to clarify under which circumstances additional preprocessing may be necessary.

4. The author mentions that HDBSCAN is not suitable for large datasets and that MMseqs2 is faster but does not provide specific data on computational requirements under different dataset sizes and parameter combinations. For users handling ultra-large datasets, such information is very important for analysis planning.

Thank you for your comment. We have included information about computational requirements in section "Methods - Benchmarking and Scoring Metrics". Further information about time and memory usage is displayed in the supplementary Table S1. To better integrate this information into the main text, we have referenced this table in the caption for Table 1.

Minor Comments
1. The introduction would be more appealing if it briefly discusses existing methods for handling large-scale viral datasets (such as reference-based alignment and k-mer approaches) and explains why cluster-based representative sequence selection offers advantages in certain applications.

Thank you for making our manuscript stronger! We've included a brief description of existing methods in the Background section.

2. The table 4 comparing clustering results would be more informative if it included a column showing the percentage of total diversity captured by each method, perhaps using a metric like phylogenetic diversity (PD) or sum of branch lengths in the representative tree versus the full tree.

Thank you for this suggestion! We included the ratio of the phylogenetic distance (PD) of the full tree and the representative tree.

3. The split graphs of IAV segments showing partial and reverse complement sequences (Figure 7) are informative, but it would benefit from quantitative metrics on how many sequences were affected and how correction improved clustering results. Provide a table summarizing the number of sequences in each category before and after correction.

We thank the reviewer for this suggestion. We have added Table S6 to the manuscript, summarising the number of representatives produced by each tool under three conditions for IAV segments 4 (H) and 6 (N): clustering the full non-redundant dataset including reverse-complement sequences, post-hoc removal of reverse-complement representatives from the original results, and full re-clustering after removing all reverse-complement sequences from the input.
The results confirm that reverse-complement sequences have a substantial quantitative impact on clustering outcomes, demonstrating that a considerable fraction of clusters in the uncorrected results arose from strand orientation artifacts rather than genuine sequence diversity. Comparable results between post-hoc correction and full re-clustering further support that ViralClust's automatic orientation correction prior to clustering is sufficient to avoid these artifacts without requiring a separate manual filtering step by the user.

The corresponding text has been added to the IAV subsection of the Results.

4. The Figure 8 showing the relationship between sequence similarity threshold and number of clusters are useful, but they only show results for segments 3 and 6. Either show results for all segments in supplementary materials or explain why these two segments were chosen as representatives.

We created these plots for all segments, but decided to include only segments 3 and 6 in the main manuscript, as these show the most divergent results. The results for the remaining segments are presented in the supplementary Figure S23.

5. Discussion the statement "Large and unevenly sampled datasets are often computationally infeasible to analyze", is somewhat vague. Specify what types of analyses become infeasible, such as multiple sequence alignment.

Thank you very much for this suggestion! We have included more detailed information in the discussion and highlighted it in the main text.

We are confident that our revised manuscript aligns more closely with the objectives of GigaScience, and we hope that it will merit your consideration for publication.

Sincerely,

on behalf of all authors
Manja Marz (corresponding author) and Sandra Triebel (first author)

manja@uni-jena.de
sandra.triebel@uni-jena.de

RNA Bioinformatics & High-Throughput Analysis Jena
Friedrich Schiller University Jena
Faculty of Mathematics and Computer Science
Jena, Germany
www.rna.uni-jena.de

Editorial Manager®

Cookie Preference Center

We use cookies which are necessary to make our site work. We may also use additional cookies to analyze, improve and personalize our content and your digital experience. For more information, see our Cookie Policy.

You may choose not to allow some types of cookies. However, blocking some types may impact your experience of our site and the services we are able to offer. See the different category headings below to find out more or change your settings.

Allow all

Manage Consent Preferences

Strictly Necessary Cookies

Always active

These cookies are necessary for the website to function and cannot be switched off in our systems. They are usually only set in response to actions made by you which amount to a request for services, such as setting your privacy preferences, logging in or filling in forms. You can set your browser to block or alert you about these cookies, but some parts of the site will not then work.

Functional Cookies

These cookies enable the website to provide enhanced functionality and personalisation. They may be set by us or by third party providers whose services we have added to our pages. If you do not allow these cookies then some or all of these services may not function properly.

Performance Cookies

These cookies allow us to count visits and traffic sources so we can measure and improve the performance of our site. They help us to know which pages are the most and least popular and see how visitors move around the site.

Targeting Cookies

These cookies may be set through our site by our advertising partners. They may be used by those companies to build a profile of your interests and show you relevant adverts on other sites. If you do not allow these cookies, you will experience less targeted advertising.

Cookie List

Clear

Apply Cancel

Consent Leg.Interest

Confirm my choices

Power
