# Supplementary material for: Reducing haystacks to needles—ViralClust: a Nextflow pipeline to cluster viral sequences
Source: Gigascience. 2026 Sep 14;15:giag090. doi: 10.1093/gigascience/giag090 (PMC13660479; doi:10.1093/gigascience/giag090)
Supplement: Supplementary file 2 [file giag090_authors_response_to_reviewer_comments_revision_1.pdf]

Author's Response To Reviewer Comments

Dear Editor and Reviewers,

Thanks again for the valuable comments on our paper titled "Reducing haystacks to needles - ViralClust: A Nextflow pipeline to cluster viral sequences".

In the following sections, we outline your specific comments and provide detailed explanations of the changes we have implemented to address each point. We have highlighted our answers as well as the additions in the manuscript as follows: blue for reviewer one (revision round two in bold) and magenta for reviewer two.

Reviewer 1

I appreciate the authors' efforts to revise the manuscript, but I do not think that the revision fully resolves several major concerns raised in the previous review.

My main concern is that ViralClust still appears more like a collection of clustering methods than a sufficiently guided analysis solution. The newly introduced scoring framework is useful, but it does not replace practical guidance for users. I recommend adding a concise decision table or workflow that suggests suitable tools and parameter ranges for common scenarios, such as outbreak-scale subsampling, segmented viruses, large DNA viruses, highly recombinant viruses, and datasets where rare lineages should be retained.
Our intention with ViralClust is not to provide a guided analysis solution or prescribe a single decision workflow for different virological scenarios. Given the diversity of viral genomes, evolutionary processes, and research questions, we do not believe that a universal decision framework or parameter recommendations would be appropriate or sufficiently robust. Instead, ViralClust is deliberately designed as a collection of complementary clustering approaches that allows users to select the method best suited to their specific dataset and analysis goals.
To support this without imposing an overgeneralized automated solution, we provide case studies within the manuscript that illustrate the practical application of the different approaches, discussing their respective strengths, trade-offs, and limitations. We believe that presenting these practical pros and cons provides users with a more realistic and scientifically rigorous basis for selecting an appropriate strategy than a prescriptive decision table, which could easily oversimplify complex biological scenarios.
We have clarified this objective in the manuscript to better communicate that ViralClust is intended as a flexible framework rather than a guided analysis pipeline.

The ambiguous-base filtering issue is also not fully resolved. Although the new `--max_ambiguous` parameter is helpful, the authors should report how many sequences are removed under default and relaxed settings, particularly for the Monkeypox dataset. The manuscript should also clarify whether this filtering is applied globally or in a tool-specific manner, and whether ORF-aware ambiguity filtering was considered.

We thank the reviewer for the continued attention to this point. We have clarified in the Materials and Methods section that ambiguous-base filtering is applied globally to the dataset prior to clustering, and that the filtered dataset serves as the shared input for all subsequent clustering tools. The relevant sentence now reads: "This filtering is applied globally to the original dataset and the filtered data serves as the input for each subsequent clustering tool." This ensures that all tools operate on an identical input and that differences in clustering output reflect algorithmic behavior rather than differences in input composition.
We have added a supplementary table reporting the number of sequences retained under different --max_ambiguous thresholds for the Monkeypox dataset. The results show that varying the threshold between 5% and 30% does not affect the number of sequences retained, indicating that the Monkeypox dataset contains very few sequences with moderate levels of ambiguous characters. Only a threshold of 0% (i.e. no ambiguous characters permitted) results in sequence removal, reflecting the presence of non-ACGU characters in repetitive genomic regions that are characteristic of large DNA virus genomes. We

clarified in the manuscript that the extra filtering of SUMACLUST is specific for this tool and is independent from the global filtering of ViralClust.

The response regarding HDBSCAN is also unclear. My concern was that rare lineages may be treated as noise and excluded from the final representative output. However, the authors mainly discuss singleton files, while singleton clusters and HDBSCAN noise points are not necessarily equivalent. The manuscript and documentation should explicitly explain how HDBSCAN noise points are handled, whether they are included in the combined representative FASTA by default, and whether users can force inclusion of all noise or singleton sequences.

We thank the reviewer for this clarification. We have revised the manuscript and documentation accordingly.
HDBSCAN noise points are conceptually distinct from singleton clusters produced by other tools, where a sequence forms its own cluster due to insufficient similarity to any existing cluster representative. However, in the context of ViralClust, both are handled equivalently: any sequence classified as noise by HDBSCAN is treated as a cluster of size one and is therefore considered an unclustered sequence. This is now explicitly stated in the manuscript: "It is important to note that any sequence classified as noise from HDBSCAN is treated as a cluster of size one and is therefore considered an unclustered sequence in ViralClust."
Unclustered sequences are included in the combined representative FASTA output by default. This means that rare lineages assigned as noise by HDBSCAN are not silently discarded but are retained as their own representative in the final output, ensuring that no input sequence is lost from the representative set solely due to being classified as noise. We have made this behavior explicit in the documentation to avoid ambiguity.
The current implementation does not provide a separate parameter to force inclusion or exclusion of noise or singleton sequences independently of the main clustering output, as they are included by default. We have added a note to the documentation clarifying this behavior so that users are aware that the representative FASTA always contains both cluster representatives and unclustered sequences, and that the distinction is reflected in the per-tool statistics reported by ViralClust.

Reviewer 2

The authors have addressed my concerns.

We would like to thank Reviewer 2 again for the valuable comments and we're happy that we were able to address all concerns.

We are confident that our revised manuscript aligns more closely with the objectives of GigaScience, and we hope that it will merit your consideration for publication.

Sincerely,

on behalf of all authors
Manja Marz (corresponding author) and Sandra Triebel (first author)

manja@uni-jena.de
sandra.triebel@uni-jena.de

RNA Bioinformatics & High-Throughput Analysis Jena
Friedrich Schiller University Jena
Faculty of Mathematics and Computer Science
Jena, Germany
www.rna.uni-jena.de

Cookie Preference Center

We use cookies which are necessary to make our site work. We may also use additional cookies to analyze, improve and personalize our content and your digital experience. For more information, see our Cookie Policy.

You may choose not to allow some types of cookies. However, blocking some types may impact your experience of our site and the services we are able to offer. See the different category headings below to find out more or change your settings.

Allow all

Manage Consent Preferences

Strictly Necessary Cookies

Always active

These cookies are necessary for the website to function and cannot be switched off in our systems. They are usually only set in response to actions made by you which amount to a request for services, such as setting your privacy preferences, logging in or filling in forms. You can set your browser to block or alert you about these cookies, but some parts of the site will not then work.

Functional Cookies

These cookies enable the website to provide enhanced functionality and personalisation. They may be set by us or by third party providers whose services we have added to our pages. If you do not allow these cookies then some or all of these services may not function properly.

Performance Cookies

These cookies allow us to count visits and traffic sources so we can measure and improve the performance of our site. They help us to know which pages are the most and least popular and see how visitors move around the site.

Targeting Cookies

These cookies may be set through our site by our advertising partners. They may be used by those companies to build a profile of your interests and show you relevant adverts on other sites. If you do not allow these cookies, you will experience less targeted advertising.

Cookie List

Clear

Apply Cancel

Consent Leg.Interest

Confirm my choices

Powe
