# Supplementary material for: Reducing haystacks to needles—ViralClust: a Nextflow pipeline to cluster viral sequences
Source: Gigascience. 2026 Sep 14;15:giag090. doi: 10.1093/gigascience/giag090 (PMC13660479; doi:10.1093/gigascience/giag090)
Supplement: Supplementary file 3 [file giag090_giga-d-26-00034_original_submission.pdf]

GigaScience

Reducing haystacks to needles - ViralClust: A Nextflow pipeline to cluster viral sequences

--Manuscript Draft--

**Manuscript Number:**	GIGA-D-26-00034
**Full Title:**	Reducing haystacks to needles - ViralClust: A Nextflow pipeline to cluster viral sequences
**Article Type:**	Research
**Funding Information:**	Deutsche Forschungsgemeinschaft (Germany´s Excellence Strategy – EXC 2051 – Project-ID 390713860) — Dr. Manja Marz<br>Bundesministerium für Forschung und Technologie (ADAPTI-M - Projetc-ID 031L0322H) — Dr. Manja Marz<br>Deutsche Forschungsgemeinschaft (NFDI 28/1 - Project-ID 460129525) — M. Sc. Sandra Triebel
**Abstract:**	Background: The rapid accumulation of viral genome sequences presents major challenges for downstream analysis tools, including tools for multiple sequence alignments, phylogeny, and genome/alignment visualization, due to computational constraints and sampling biases caused by outbreak-driven over-representation. Selecting representative genomes through clustering offers a principled alternative to random subsampling, yet choosing appropriate clustering strategies remains non-trivial and context dependent.<br>Results: Here, we present ViralClust, a modular Nextflow pipeline for bias-aware representative selection from large viral genome datasets. ViralClust integrates five distinct clustering algorithms (CD-HIT-EST, SUMACLUST, VSEARCH, MMSeqs2, and HDBSCAN) within a unified workflow, enabling direct comparison of clustering outcomes and flexible adaptation to diverse biological questions, considering a balanced phylogenic distribution of the selected sequences. We evaluated ViralClust on six RNA and DNA virus datasets ranging from 632 to 156,586 sequences and spanning genome lengths from 890 to 197,185 nucleotides. Across all datasets, clustering reduced dataset size by 95% or more while preserving genetic diversity across species, genera, and families, and effectively mitigating biases introduced by outbreaks, partial genomes, and sequence orientation artifacts.<br>Conclusions: By supporting whole-genome clustering and scalable representative selection, ViralClust enables efficient and reproducible downstream analyses that would otherwise be computationally infeasible. Our framework provides a flexible foundation for large-scale viral genomics and supports future applications in comparative analysis and virus classification.
**Corresponding Author:**	Sandra Triebel<br>Friedrich Schiller University Jena: Friedrich-Schiller-Universitat Jena<br>Jena, GERMANY
**Corresponding Author Secondary Information:**	
**Corresponding Author's Institution:**	Friedrich Schiller University Jena: Friedrich-Schiller-Universitat Jena
**Corresponding Author's Secondary Institution:**	
**First Author:**	Sandra Triebel
**First Author Secondary Information:**	
**Order of Authors:**	Sandra Triebel<br>Kevin Lamkiewicz<br>Manja Marz
**Order of Authors Secondary Information:**	

Powered by Editorial Manager® and ProduXion Manager® from Aries Systems Corporation

Additional Information:	
**Question**	**Response**
Are you submitting this manuscript to a special series or article collection?	No
**Experimental design and statistics**<br><br>Full details of the experimental design and statistical methods used should be given in the Methods section, as detailed in our Minimum Standards Reporting Checklist. Information essential to interpreting the data presented should be made available in the figure legends.<br><br>Have you included all the information requested in your manuscript?	Yes
**Resources**<br><br>A description of all resources used, including antibodies, cell lines, animals and software tools, with enough information to allow them to be uniquely identified, should be included in the Methods section. Authors are strongly encouraged to cite Research Resource Identifiers (RRIDs) for antibodies, model organisms and tools, where possible.<br><br>Have you included the information requested as detailed in our Minimum Standards Reporting Checklist?	Yes
**Availability of data and materials**<br><br>All datasets and code on which the conclusions of the paper rely must be either included in your submission or deposited in publicly available repositories (where available and ethically appropriate), referencing such data using a unique identifier in the references and in the “Availability of Data and Materials” section of your manuscript.	Yes

Have you have met the above requirement as detailed in our Minimum Standards Reporting Checklist?	
GigaScience has policies and guidelines in place for the use of generative AI-writing tools such as ChatGPT. If you have used such writing tools to assist with writing the manuscript this must be declared and cited in the text. Authors should not list AI-writing tools and other AI-assisted technologies as an author or co-author and should acknowledge that they are fully responsible for text generated or refined by AI-writing tools.<p><br><br>A summary of use (particularly in the introduction or among methods) needs to be included at the end of the paper, and the outputs should also be included as a supplementary file hosted in GigaDB or other open repositories. Please read our guidelines for more information. <p><br><br>By submitting to GigaScience, you are aware of the journal's AI-writing tools policy, and if you have declared use of such tools below, you have acknowledged this where appropriate in your manuscript and have made a summary of use and outputs available. <p><br><b>AI-assisted writing tools have been used in the preparation of this manuscript?	No

Manuscript

Click here to access/download;Manuscript;STriebel_ViralClust.pdf

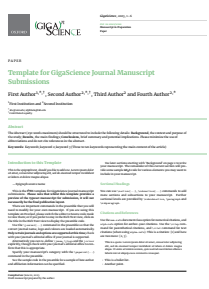

GigaScience, 2026, 1–S26

doi: xx.xxxx/xxxx
Manuscript in Preparation
Paper

PAPER

Reducing haystacks to needles – ViralClust: A Nextflow pipeline to cluster viral sequences

Sandra Triebel[1,2], Kevin Lamkiewicz[1,2] and Manja Marz[1,2,*]

[1]RNA Bioinformatics and High-Throughput Analysis, Friedrich Schiller University Jena, Jena, 07743, Germany and [2]European Virus Bioinformatics Center, Friedrich Schiller University Jena, Jena, 07743, Germany

[*] manja@uni-jena.de

Abstract

Background The rapid accumulation of viral genome sequences presents major challenges for downstream analysis tools, including tools for multiple sequence alignments, phylogeny, and genome/alignment visualization, due to computational constraints and sampling biases caused by outbreak-driven over-representation. Selecting representative genomes through clustering offers a principled alternative to random subsampling, yet choosing appropriate clustering strategies remains non-trivial and context dependent.
Results Here, we present `ViralClust`, a modular `Nextflow` pipeline for bias-aware representative selection from large viral genome datasets. `ViralClust` integrates five distinct clustering algorithms (`CD-HIT-EST`, `SUMACLUST`, `VSEARCH`, `MMSeqs2`, and `HDBSCAN`) within a unified workflow, enabling direct comparison of clustering outcomes and flexible adaptation to diverse biological questions, considering a balanced phylogenic distribution of the selected sequences. We evaluated `ViralClust` on six RNA and DNA virus datasets ranging from 632 to 156,586 sequences and spanning genome lengths from 890 to 197,185 nucleotides. Across all datasets, clustering reduced dataset size by ~95 % or more while preserving genetic diversity across species, genera, and families, and effectively mitigating biases introduced by outbreaks, partial genomes, and sequence orientation artifacts.
Conclusions By supporting whole-genome clustering and scalable representative selection, `ViralClust` enables efficient and reproducible downstream analyses that would otherwise be computationally infeasible. Our framework provides a flexible foundation for large-scale viral genomics and supports future applications in comparative analysis and virus classification.

Key words: Virus; Clustering; Nextflow; Bioinformatics; Viral Evolution

Background

Viral genome sequences are becoming increasingly available [1, 2, 3, 4, 5]. For example, for SARS-CoV-2 alone, nearly 9.2 million uploaded nucleotide sequences are accessible (NCBI Virus, 19.11.2025). The use of such large datasets of viral sequences often poses problems for downstream analyses, such as sequence alignment, phylogeny, and visualization, in terms of memory usage, run time, and visualization [6, 7]. Calculating a multiple sequence alignment (MSA) of available viral genomes of a family is often computationally not feasible. Disregarding the technical demands, an MSA with tens of thousands of entries may not be informative for most users, since the overall alignment quality potentially decreases. Besides the size of the datasets, the over-representation of a certain species due to an outbreak might also introduce a bias into the data. To reduce the number of entries, random sampling of genomes does not solve the introduced bias of over-represented species it may even increase the effect of the bias. Consequently, the aim is to reduce the dataset of viral sequences without losing genetic diversity. Instead of random selection, clustering viral genomes based on, e.g., sequence similarity and selecting a representative genome for each cluster results in a better-balanced subset of input viruses. While the idea of clustering is simple, the correct method is often hard to find and depends on the underlying scientific question. Depending on the biological question and distribution of biological data, the principle of *compactness* is most widely used, aiming to keep intra-cluster variation small. It is implemented in tools such as `CD-HIT-EST` [8], `SUMACLUST` [9], `VSEARCH` [10], and `MMSeqs2` [11, 12]. In

Compiled on: January 31, 2026.
Draft manuscript prepared by the author.

Key Points

- ViralClust is a modular Nextflow pipeline for selecting representative viral genomes from (very) large sequence datasets.
- It combines multiple clustering approaches to reduce dataset size while minimizing bias and preserving genetic diversity.
- Tests on six RNA and DNA virus datasets show reductions of ~95 % across a wide range of genome sizes and sequence counts.
- ViralClust enables efficient and reproducible downstream analyses that are otherwise impractical with full viral genome collections.

this context, the sequence identity determines whether a sequence belongs to a cluster or forms a new, independent cluster. Thus, compactness clustering is based on sequence homology. In most implementations, a sequence is only compared against one representative of established clusters. This method is used frequently for viral clades [13, 14, 15]. However, evolution is a continuous process, and many novel observed viruses might not be unambiguously assigned to the calculated clusters based on static sequence similarity thresholds alone. An algorithm following the principle of *connectedness* may be suitable for such continuous data. The principle of connectedness exploits the idea that neighboring data points should belong to the same cluster. Therefore, algorithms following this principle tend to detect smaller, local components within the data that are not concentrated around a center point [16]. For datasets with viral genomes such that the data points are not well-described by high-dimensional spheres, as is the case for compactness clusters, we propose a tool such as HDBSCAN [17]. Virologists, mostly lacking computational backgrounds, often find themselves unable to navigate the intricacies of algorithmic advantages and disadvantages, leaving them uncertain about selecting the most suitable tool for their analyses. For bioinformaticians, evaluating which tool to choose presents its own challenges, as the absence of a definitive ground truth complicates the determination of which sequences best represent a cluster.

Here, we present ViralClust, a Nextflow pipeline designed to select representative viral genomes, by a streamline clustering analysis for virology research. Unlike conventional methods, ViralClust integrates clustering via five distinct existing tools, enabling direct and comprehensive comparison of the results. One key feature of ViralClust is its robust data pre-processing capabilities. We address common database errors, such as inconsistencies in sequence orientation (-ssRNA sequences found in both positive and negative orientations) and ambiguous gene arrangements in ambisense viruses. By correcting these mistakes, we enhance the accuracy of clustering outcomes. Moreover, ViralClust introduces innovative methodologies previously unexplored in virology research. For instance, full genome clustering based on k-mers of nucleotide sequences utilizing Hierarchical Density-Based Clustering (HDB) represents a novel approach in virus research, offering previously inaccessible insights. Finally, we provide comprehensive statistics, including cluster sizes, and visualize the sequence selection in rough phylogenetic representations without explicitly generating phylogenies of the viral families. Additionally, our HTML output facilitates easy cluster sorting, including direct links to corresponding NCBI genomes for further analysis. All parts of the developed pipeline are written modular, and the user may adjust parameters or exchange and add complete tools. ViralClust offers the possibility of adding manually additional viral genomes of interest (GOI) to the input data. We evaluated the performance of ViralClust on six different datasets of RNA and DNA viruses varying in number of available sequences, sequence length, and sequence diversity, such as segmentation and orientation.

Data Description

Viral data

We downloaded viral genomes labeled as "complete" of *Orthoebolavirus*, *Hepacivirus hominis* (HCV), *Orthoflavivirus*, *Flaviviridae*, *Alphainfluenzavirus influenzae* (formerly *Influenza A virus*, IAV), and *Monkeypox virus* from the NCBI Virus database, see Tab. 1. All sequences used in this study are deposited in the zenodo repository (Link: https://doi.org/10.5281/zenodo.18413373) in fasta format.

"Ground Truth" of Virus Phylogeny

To compare the results of ViralClust, we considered the classification of the ICTV [18, 19] as "ground truth" for *Orthoebolavirus*, *Orthoflavivirus*, *Flaviviridae*. For *Hepacivirus hominis* and *Monkeypox virus* we included the NCBI taxonomy. The number of genera, subgenera and/or species is derived from https://ictv.global/taxonomy, see Tab. S2. For *Alphainfluenzavirus influenzae* we included the classification according to the Centers for Disease Control and Prevention (CDC)[1] into 18 different H subtypes and 11 different N subtypes. This theoretically results in 198 possible subtypes, but 136 subtypes are currently listed in the NCBI taxonomy (23.01.2026).

Analyses

The ViralClust pipeline

Our pipeline ViralClust takes a multiple fasta file as input and applies five different cluster tools to the data to select representative genomes. We calculate and report basic statistics for each of the cluster tools. Based on the respective representative genomes, ViralClust further calculates a multiple sequence alignment (MSA) and a rough phylogenetic representation (parameter --eval). Fig. 1 gives a general overview of the pipeline. Additionally to the clustering step, the user can retrieve an optional basic evaluation of the results. This evaluation is no recommendation for any algorithm or tool, as the choice of clustering depends on the underlying scientific question. However, it gives a broad overview of the data and may help determine the appropriate cluster method. If applicable, ViralClust finds GenBank accession IDs in the fasta records identifier and retrieves meta information from the NCBI database. Lastly, the user can specify a second, optional fasta file containing genomes that must be present in the final set of representative genomes. This may prove useful if comparative analyses between viruses are carried out and the user wants to include a specific, unpublished viral strain or isolate in the analysis.

Workflow of ViralClust
We implemented ViralClust in Nextflow (v22.10.06) [20]. We developed our scripts with Python (compatible with v3.6+). We employ in-

1 https://www.cdc.gov/flu/about/viruses-types.html Last Reviewed: 23.01.2026

dividual conda (v4.9.2) [21] environments for each step per default to guarantee no conflicts during installation and usage. ViralClust is publicly available at https://github.com/rnajena/viralclust. We further provide a test dataset with a small tutorial on using ViralClust. In the following, we describe the pipeline depicted in Fig. 1 in detail, including implemented tools and parameter configurations.

Pre-processing. ViralClust only considers sequences that consist of at least 90 % ACGU characters. Both strands are then scanned for ORF density to ensure that all sequences have the same orientation. Especially in the case of single-stranded negative RNA viruses, genomes are sometimes uploaded into the database in different orientations. This causes cluster algorithms to separate genomes based on this orientation, potentially jeopardizing the complete clustering approach. We calculate ORF density by translating an input genome into all six reading frames. We define an open reading frame (ORF) as a sequence of at least 200 amino acids between two stop codons or between a single stop codon and the 5' or 3' end of the sequence. This makes the procedure robust against missing stop codons at the end of the sequence and missing start codons. We calculate the forward-strand ORF density by summing the lengths of the ORFs in the three forward reading frames, then dividing by the sequence length. The backward-strand ORF density is calculated analogously. This method accounts for real or erroneous frameshifts. We use the strand with the higher ORF density for further calculations. The step is enabled by default; the user can disable it using the parameter sort_off. To remove 100 % redundant genomes in the input dataset, we apply the easy-linclust module of MMSeqs2 (v14) [11, 12], with parameters --min-seq-id 1.0.

Compactness Clustering. Per default, ViralClust applies five different clustering methods. Depending on which scientific virological question is asked, one of the tools might be more suitable than another. Due to the modularity of the pipeline, the user can also choose to switch off the predefined cluster tools and/or add individual cluster methods. Currently, we implemented CD-HIT-EST (v4.8.1) [8], SUMACLUST (v1.0.31) [9], VSEARCH (v2.15) [10] from vsearch, MMSeqs2 (v14) [11, 12], and HDBSCAN (v0.8.26) [17] as individual Nextflow modules. Whenever applicable, we set the parameters of the tools to match each other to make them comparable. By default, ViralClust instructs CD-HIT-EST, SUMACLUST, VSEARCH, and MMSeqs2 to cluster sequences with an identity of at least 90 % (default value of CD-HIT-EST).

Connectedness Clustering with HDBSCAN. As a novel approach to cluster viral genomes, we further implemented a combination of PCA and HDBSCAN [17] in ViralClust. *(1) k-mer distributions:* For this approach, we translate each input genome into a vector of k-mer frequencies (default $k = 7$), neglecting k-mers consisting of non-canonical nucleotides. *(2) Dimension reduction:* For the PCA, we use the module as implemented in scikit-learn (v1.2.2) [22]. Instead of the dimension reduction with PCA, we further allow using UMAP for the reduction. *(3) Clustering:* For efficient clustering, we reduce the resulting 4^k-dimensional (default $k = 7$) vector space and thus provide an embedding for the clustering algorithm HDBSCAN. Per default, we either reduce the dimension to $d = 50$ or until the sum of explained variance of the components is at least 0.7. After dimension reduction, each viral genome is represented by a vector of maximal length d ($d = 50$). HDBSCAN can be seen as a hybrid approach of hierarchical-based (connectivity) and density-based (compactness) clustering approaches. For two vectors, HDBSCAN first calculates mutual reachability distance. Per default, we use the cosine distance implemented in SciPy (v1.4.1) [23] as metric. However, we allow for a plethora of pre-defined distances, which can be used via the --metric parameter of our HDBSCAN module. We implemented the (standardized) Euclidean, Manhattan, Chebyshev, Minkowski, Canberra, Braycurtis, and Mahalanobis distance metrics via the SciPy library [23]. Since the used implementation of HDBSCAN cannot deal with the cosine distance, we normalized the vectors using the L2 normalization. Subsequently, we use HDBSCAN with the Euclidean distance on these normalized vectors[2].

The user can override or provide additional parameters for each tool via the nextflow run viralclust.nf command. Exemplarily, for HDBSCAN, the user can use the flag --hdbscan_help to get an overview of implemented options, which can be used using --hdbscan_params. Analogously, help messages and parameters for the other tools can be used and accessed via their respective commands.

Determination of representative genomes. The implemented tools report representative genomes based on sequence length (CD-HIT-EST, SUMACLUST), sequence abundance (VSEARCH), and selection within cascaded clustering (MMSeqs2). For the HDBSCAN module, we calculate centroid sequences based on the average pairwise distance between all vectors in the same cluster. The sequence with the minimum average distance is selected as the representative genome for this cluster. Given a distance metric d, the centroid sequence c of a cluster C with size N is the one that has the lowest average distance $\overline{d}$ to all other sequences S_i ($i \in [1, N]$) of the cluster: $\overline{d_C} = \min_{i \in C} \frac{1}{N-1} \sum_{j=1}^{N} d(S_i, S_j)$. Thus, each cluster is represented by an input genome, not an "artificial" median genome. Per default, we use the cosine distance as metric d but also allow for other metrics.

In ViralClust, only clusters with a size of more than one are defined as "real" clusters and the corresponding representatives are saved in a file. Clusters of size one are therefore not considered clusters, but named 'singletons' here, and are stored in a separate file. The combined set of cluster representatives and 'singleton' sequences is provided in a third file. This allows the user to choose which set of sequences is most suitable for the research question.

Optional parameters. (1) Inclusion of a genome of interest: In addition to the general input file, the user can specify a second fasta file with the --goi flag. This fasta file should contain one (or more) genome(s) of interest (GOI). If the user provides such a file, ViralClust will include the contained genomes in the final set of representative genomes. Further, in the result reports of the individual cluster tools, the GOIs are specifically marked. With this option, users can add their unpublished in-lab strain or mutant to downstream analyses and can further determine the closest phylogenetic viruses based on the resulting clusters. This also enables comparisons between the representative sequences and specific reference genomes, which are not necessarily cluster representatives. *(2) Metadata Information:* With the --ncbi flag ViralClust links additional information provided by the NCBI [24]. For this, we can sequence identifiers for GenBank accession ID. If found, we use the accession ID to retrieve additional information about the taxonomy (species and genus name), accession date, and the country the sequence originated from. ViralClust combines this metadata with the overview of clusters for each tool. *(3) Alignment and Phylogeny:* If the user sets the flag --eval of ViralClust, we further create a multiple sequence alignment (default: MAFFT (v7.4) [25]) of the representative genomes for each cluster tool. We subsequently use the alignments to build phylogenetic representations (default: FastTree (v2.1.10) [26]), which we finally visualize using Newick Utilities (v1.6) [27]. If --eval is enabled, we include the average phylogenetic distance calculated by FastTree between two representative genomes in the final summary report. Per default, FastTree uses the Jukes-Cantor distance model [28].

2 as communicated in: https://github.com/scikit-learn-contrib/hdbscan/issues/69#issuecomment-317742362

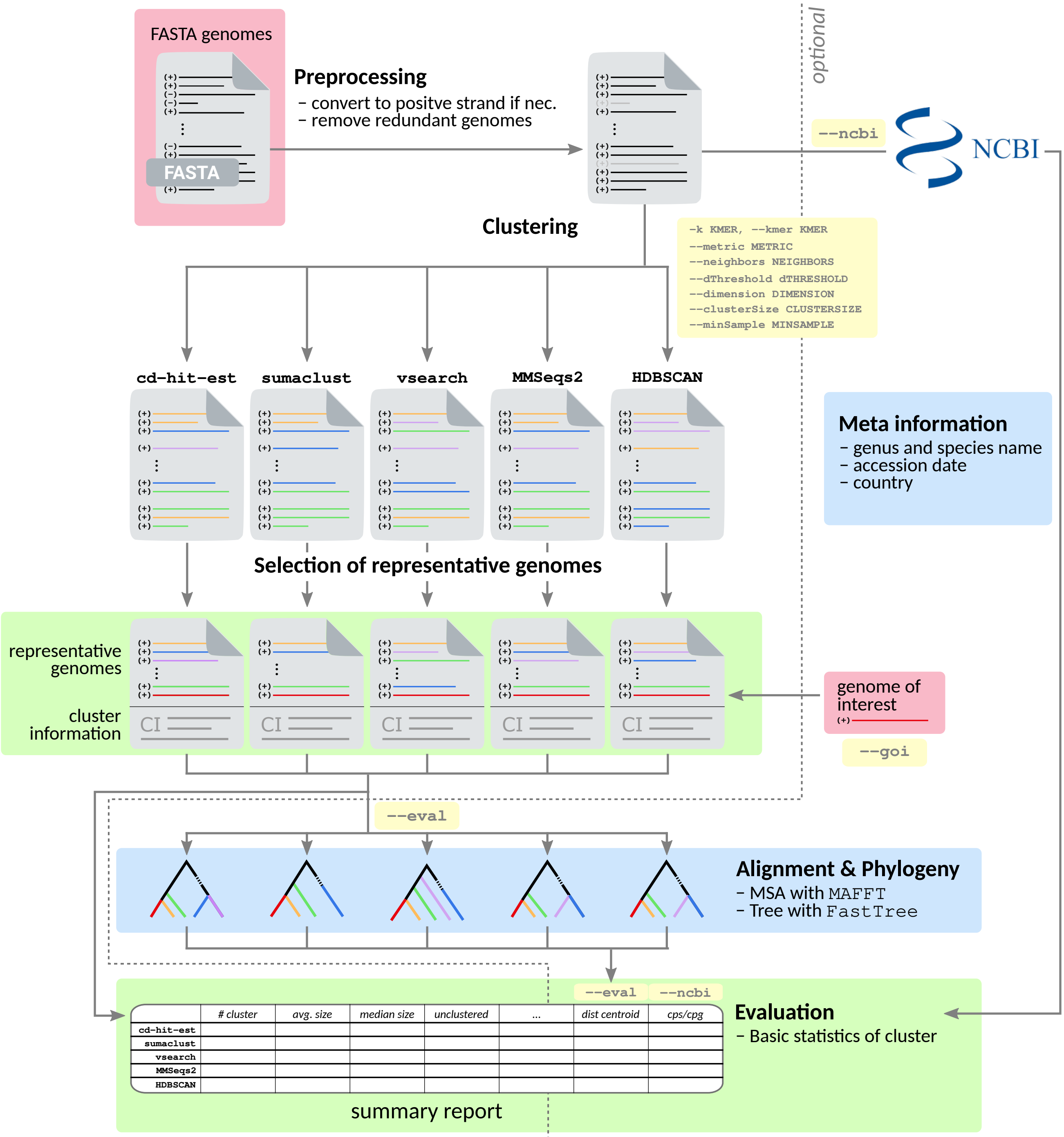

Figure 1. The general `ViralClust` pipeline. *Pre-processing.* Input genomes are filtered for redundant genomes and oriented to the positive strand determined via an ORF density scan. *Clustering.* Five different tools are pre-installed. However, each can be exchanged by other customized clustering tools. For each clustering method applied, `ViralClust` provides an overview of which sequence falls into which cluster. *Representative genomes.* Further, a `fasta` file with representative sequences for each cluster is provided for each tool. `--goi` – Genomes of interest (GOI) or unpublished genomes can be added to the representative genomes. *Evaluation.* The basic statistics of the cluster results, including average and median cluster size and the average distance of two representative genomes are given for each cluster tool. *Optional parameters.* `--ncbi` – For each applicable sequence, *i.e.* for each `fasta` record with a valid GenBank accession ID in its identifier, `ViralClust` retrieves additional information from the NCBI database and includes it in the summary report. `--eval` – For each clustering method `ViralClust` can provide an alignment of representative genomes and a phylogenetic representation of these genomes.

Benchmark `ViralClust` by a variety of datasets

We applied `ViralClust` to six viral datasets of single-stranded RNA and double-stranded DNA viruses varying in number of sequences, sequence length, and sequence diversity. For an overview of the datasets used in this study, we refer to Tab. 1, in which we provide the total number of available sequences, the number of sequences after the implemented filtering steps, and the number of clusters resulting from each tool. Given these input sets, we further report the number of clusters as calculated by the individual tools in their respective column. Additionally, we report the overall run-time of the complete `ViralClust` pipeline for each dataset. We present the run-time and memory usage for each pipeline step in Supplement Tab. S1. All results generated by `ViralClust` are deposited in the zenodo repository: https://doi.org/10.5281/zenodo.18413373.

Table 1. Overview of datasets used in this study. `ViralClust` reduces the number of input genomes to a representative set of genomes. Data was downloaded from NCBI Virus. ⌀ SeqLen – average sequence length; #Seq – number of input genomes / segments; contains only records labeled as "`complete`"; PreProc – number of sequences after pre-processing; %nr – percentage of non-redundant sequences; Number of genomes after clustering – only considering clusters with a size greater than one; cd – CD-HIT-EST; sum – SUMACLUST; vse – VSEARCH; mmseq – MMSeqs2; hdb – HDBSCAN; Time – run time of `ViralClust` measured by `Nextflow`.

Taxonomic clade	⌀ SeqLen	#Seq	PreProc	%nr	Number of genomes after clustering					Time
					cd	suma	vse	mmseq	hdb	
Orthoebolavirus	18.9 kb	632	443	70.09	8	7	7	11	11	12m
Hepacivirus hominis	9.6 kb	1,075	1,071	99.63	55	30	58	55	23	14m
Orthoflavivirus	9.2–11 kb	8,052	7,681	95.39	69	71	70	63	101	1h 22m
Flaviviridae	9–13 kb	9,895	9,478	95.79	220	180	221	211	194	2h 47m
Alphainfluenzavirus influenzae (1)	2,341 nt	83,543	56,973	68.20	66	390	90	280	1,412	3h 27m
Alphainfluenzavirus influenzae (2)	2,341 nt	82,569	56,212	68.07	56	338	73	216	1,311	3h 24m
Alphainfluenzavirus influenzae (3)	2,233 nt	83,400	55,862	66.98	63	305	75	265	1,371	3h 16m
Alphainfluenzavirus influenzae (4, H)	1,775 nt	156,586	108,514	69.30	300	1,237	464	1,574	2,307	8h 36m
Alphainfluenzavirus influenzae (5)	1,565 nt	84,653	49,465	58.43	46	262	76	179	1,218	2h 28m
Alphainfluenzavirus influenzae (6, N)	1,409 nt	114,639	73,786	64.36	228	773	329	640	1,622	4h 9m
Alphainfluenzavirus influenzae (7)	1,027 nt	96,928	46,371	47.84	25	185	38	269	981	1h 47m
Alphainfluenzavirus influenzae (8)	890 nt	85,944	42,308	49.23	51	218	69	264	802	1h 28m
Monkeypox virus	190–200 kb	1,077	999	92.76	14	1	3	23	2	16h 0m

Small and biased dataset - Orthoebolavirus

We tested the *Orthoebolavirus* dataset consisting of only 632 genomes to see how fine-grained `ViralClust` can work. *Orthoebolavirus* is a genus within the *Filoviridae* family (ICTV phylogeny see Fig. S1). The genomes are negative single-stranded RNA and about 18.9 kb in length [29]. In comparison to other viral clades, this dataset includes a small number (six) of well-described species according to the ICTV, see Tab. 2, with a notable presence with 461/632 sequences of *Orthoebolavirus zairense* (Ebola virus, EBOV), a human pathogen over-represented due to several outbreaks. We aim to investigate the selection of representative genomes with `ViralClust` for such a small, well-defined, and unbalanced dataset. The pre-processing of `ViralClust` reduced the dataset size by 30 % to 443 genomes. Subsequent clustering resulted in 7 to 11 representative genomes, see Tab. 1. In general, all five clustering tools could distinguish the species in individual clusters, see Fig. 2. A conventional representation of the phylogeny of the representative genomes and the corresponding ICTV classification can be found in Fig. S2. Additionally, in Fig. S3 a RAxML-NG phylogenetic tree based on a full-genome MAFFT alignment can be found as an overview for the 443 individual sequences. CD-HIT-EST, SUMACLUST, VSEARCH and MMSeqs2 revealed correctly one cluster representative for the five species BOMV, BDBV, RESTV, SUDV, and TAFV each. A notable exception is HDBSCAN, which could not identify the species TAFV, Fig. 2B and Fig. S2F. All tools clustered TAFV genomes (light blue) correctly together with BDBV (dark green). Each of the tools selected at least one natural EBOV strains (CD-HIT-EST:1, SUMACLUST:1, VSEARCH:1, MMSeqs2:4, HDBSCAN:7). Additionally, CD-HIT-EST, SUMACLUST, VSEARCH and MMSeqs2 selected up to two representatives of EBOV derived from recombination or reverse genetics experiments (see Fig. 2 dashed gray box). HDBSCAN selected only natural EBOV sequences (isolated from viral infections). Interestingly, these comparatively high number of representative genomes represent geographic and temporal separation of EBOV outbreaks.

Table 2. *Orthoebolavirus* species including virus name and abbreviation (Abbr.) according to ICTV and number of downloaded sequences per species (#Seqs). 93 sequences were unclassified regarding their species.

Species	Virus name	Abbr.	#Seqs
O. bombaliense	Bombali	BOMV	8
O. bundibugyoense	Bundibugyo	BDBV	24
O. restonense	Reston	RESTV	22
O. sudanense	Sudan	SUDV	21
O. taiense	Taï Forest	TAFV	3
O. zairense	Ebola	EBOV	461

Species Hepacivirus hominis and genus Orthoflavivirus from the family Flaviviridae

Flaviviridae is a large family of positive single-stranded RNA viruses consisting of four genera: *Orthoflavivirus*, *Pestivirus*, *Pegivirus* and *Hepacivirus* [30], with a total of 97 species (ICTV, see Fig. S4). *Flaviviridae* includes human pathogenic viruses such as Zika virus, tick-borne encephalitis virus (TBEV), dengue virus, and hepatitis C virus (HCV). Genomes are approximately 9–13 kb in length [30]. We chose three datasets to compare results on species, genus, and family level. On the species level, we chose *Hepacivirus hominis* (HCV infecting humans) as a well-described and phylogenetically closely related species. On a genus level, we chose *Orthoflavivirus* due to the well-described dataset being very diverse (ICTV: 53 species). We chose *Flaviviridae* on the family level. The datasets *Hepacivirus hominis* and *Orthoflavivirus* are complete subsets of the dataset *Flaviviridae*. Finally, our focus was on how the selected representatives from species and genus study are consistent within the selection from the entire virus family. Preprocessing within the `ViralClust` pipeline reduced the datasets by 1–5 %, see Tab. 1 and 3.

Species Hepacivirus hominis. *Hepacivirus hominis* (HCV) consists of eight genotypes [31, 32, 33], however, only seven are listed in the ICTV, see Fig. S4 yellow rectangle. With the neighbour-net method we gained 13 clearly distinguishable clusters, see Fig. 3. This difference can also be explained by the observations of virologists in the past: (i) Two additional clusters, besides the seven downloaded genotypes, are formed by recombinants: the natural recombinants (e.g. 2k/1b or 2b/1b, Fig. 3 – close related to genotype 1b) [34, 35, 36] and the artificial recombinants (Fig. 3 – between genotype 1 and 2) [37, 38, 39]. Note: further recombinants are mixed into the genotype 2. (ii) Three additional clusters are formed within genotype 1 – in total, divided into genotype 1, 1a, 1b, and rare genotypes 1 (1c–1n). Currently, 14 subtypes are listed in the ICTV [30, 40, 41]; (iii) One additional cluster exists due to the diverse genotype 6: Currently, 33 subtypes are known for genotype 6 according to the ICTV [30, 42], with subtype 6a being the first one discovered [43]. All sequences part of subtype 6a cluster distinguishable from other subtypes, with the notable exception of subtype 6b, which more close to 6a than other subtypes. The tools of `ViralClust` calculated between 23 (HDBSCAN) and 58 (VSEARCH) representative genomes (see Fig. 3 colored circles, and Tab. 1). Al-

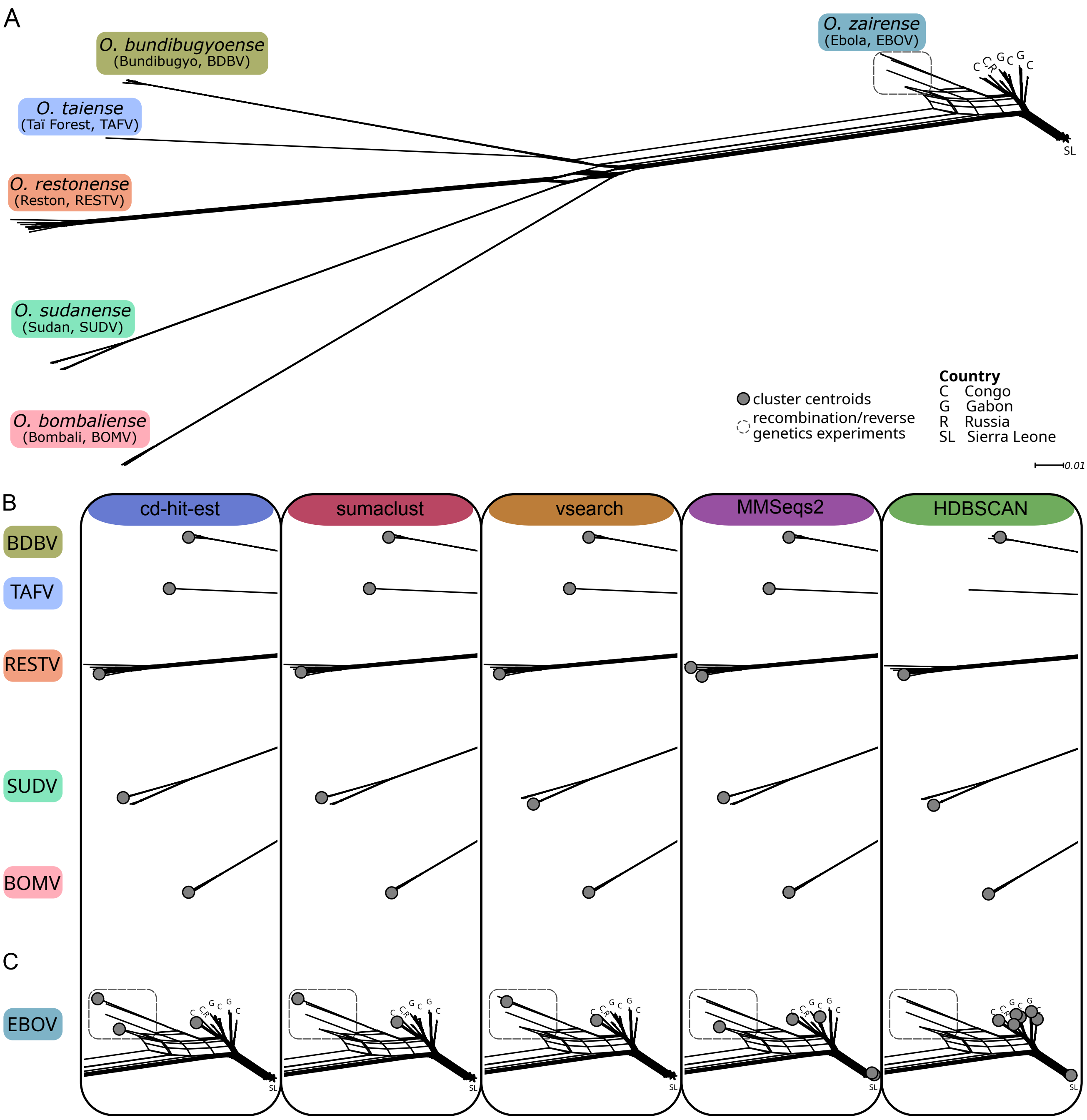

Figure 2. (A) Split graph of the *Orthoebolavirus* dataset with labeled species. (B) Cluster representatives of CD-HIT-EST, SUMACLUST, VSEARCH, MMSeqs2, and HDBSCAN in the subtrees of BDBV, TAFV, RESTV, SUDV, and BOMV (top to bottom). (C) Cluster representatives in the EBOV subtree. Gray dots display the cluster representative sequences selected by the individual tools. The Zaire *Orthoebolavirus* subtree shows a distinction by geographic origin, indicated as follows: C – Congo; G – Gabon; R – Russia; SL – Sierra Leone. The split graph is reconstructed using SplitsTree based on the MAFFT alignment of the complete genome sequences (443 genomes) resulting from the first filtering steps of ViralClust.

though any of the methods represents 3–8 times more clusters than the currently recognized number of HCV genotypes, using more representatives may be necessary to capture the considerable diversity within genotypes. Among the evaluated methods, HDBSCAN produced the largest clusters (minimum size 5, average size 40.87, median size 14; see Tab. 3), resulting in the lowest total number of clusters. All clustering tools in ViralClust selected representative genomes distributed across the phylogenetic tree. However, substantial differences in the number of representatives were observed for the different genotypes, see Fig. 3. Interestingly, none of the tools was able to identify genotype 7 as an individual cluster. Across all five clustering algorithms, the largest cluster was consistently identified as genotype 1a. Each tool selected 1–3 representatives for this cluster. While four tools (CD-HIT-EST, VSEARCH, MMSeqs2, HDBSCAN) selected the same representative genome for genotype 1b, SUMACLUST identified seven. Notably, HDBSCAN did not select any rare subtypes of genotype 1, whereas the other tools included one (SUMACLUST) or six (CD-HIT-EST, VSEARCH, MMSeqs2) representatives. This is likely due to the k-mer–based similarity measure used by HDBSCAN, which appears to group the rare genotype 1 subtypes more tightly, leading to no additional representatives. A small subtree annotated as genotype 1 (Fig. 3, bottom right) was only found to be an independent cluster by HDBSCAN, which selected a single genome from these variants. The large number of subtypes within genotype 6 can be explained by the diversity of this genotype, see Fig. 3, even tough the tools showed picked different representatives. The

Table 3. Cluster statistics for *Hepacivirus hominis* (HCV), *Orthoflavivirus* (*Flavivirus*), and *Flaviviridae* datasets. For each tool, the basic cluster statistics are listed. cd – `CD-HIT-EST`; sum – `SUMACLUST`; vse – `VSEARCH`; mmseq – `MMSeqs2`; hdb – `HDBSCAN`; # clstr – number of clusters (with a size greater than one); min/max – size of smallest/largest cluster; avg/med – average/median cluster size; d. cen – average phylogenetic distance between two representative genomes; singl – number of singletons (= cluster with only one element); cps – average cluster per species; cpg – average cluster per genus. The last two columns give a measurement of how much a single species/genus is divided into different groups.

	algorithm	# clstr	min	max	avg	med	singl	d. cen	cps	cpg
HCV	cd	55	2	410	17.71	3.0	97	0.804	1.75	23.00
	suma	30	2	373	24.67	3.0	42	0.637	2.08	15.00
	vse	58	2	412	16.97	3.0	87	0.806	1.74	24.00
	mmseq	55	2	410	17.75	3.0	95	0.814	1.82	17.00
	hdb	23	5	412	40.87	14.0	131	0.594	1.61	21.00
Flavivirus	cd	69	2	1,642	110.14	5.0	81	1.510	1.44	46.00
	suma	71	2	1,622	96.44	5.0	86	1.663	1.57	49.00
	vse	70	2	1,431	108.63	6.0	77	1.493	1.42	46.00
	mmseq	63	2	1,798	120.68	6.0	78	1.704	1.32	30.00
	hdb	101	5	1,371	69.19	19.0	693	1.189	2.49	100.00
Flaviviridae	cd	220	2	1,642	41.54	3.0	339	2.466	1.64	20.60
	suma	180	2	1,622	44.81	3.0	267	2.739	1.73	21.00
	vse	221	2	1,431	41.46	3.0	316	2.280	1.62	27.75
	mmseq	211	2	1,798	43.33	3.0	336	2.520	1.54	18.00
	hdb	194	5	1,371	41.98	14.0	1,333	1.704	2.09	39.40

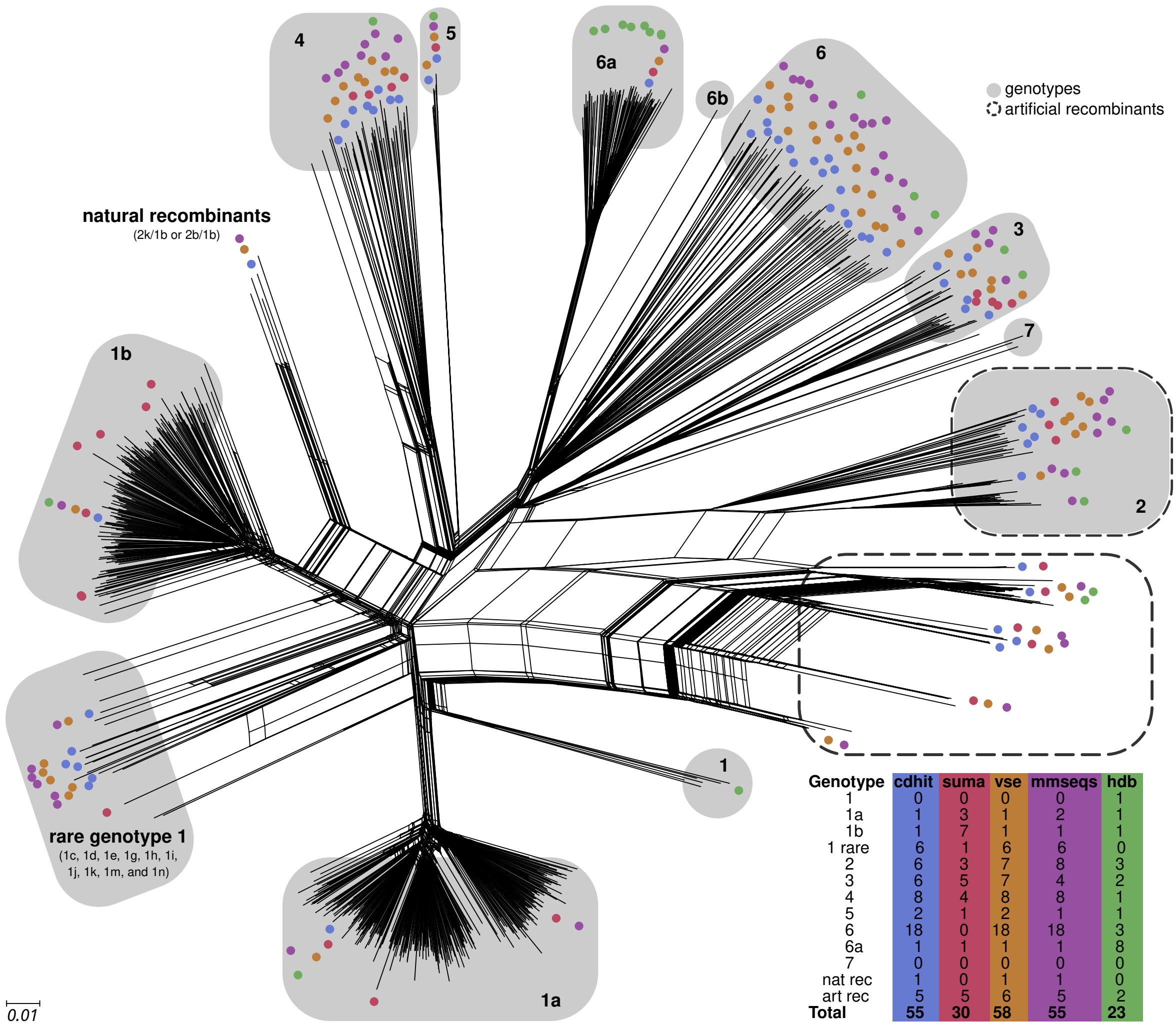

Figure 3. Splits graph of HCV with labeled genotypes (gray), artificial (dashed box) and natural recombinants, and cluster representatives of the five tools incorporated in `ViralClust` (`CD-HIT-EST`, `SUMACLUST`, `VSEARCH`, `MMSeqs2`, and `HDBSCAN`). Numbers in gray boxes represent genotypes. The tree can be divided into 13 subtrees, whereas genotype 1 consists of four subtrees (1, 1a, 1b, and the rare genotypes 1c–1n); and all subtype 6a genomes are clearly distinguishable from all other subtypes summarized here as genotype 6. All known natural recombinants (e.g. 2k/1b or 2b/1b) form a separate subtree most closely related to genotypes 1b and 4. Some artificial recombinants mix with genotype 2 genomes, whereas others form a subtree between genotypes 1 and 2. The number of representatives per genotype is shown in the table at the bottom right. The split graph is reconstructed using `SplitsTree` based on the `MAFFT` alignment of the complete genome sequences (1,071 genomes) resulting from the first filtering steps of `ViralClust`.

Accession ID	cd-hit-est	sumaclust	vclust	MMseqs2	HDBSCAN
AB520610	Cluster 17	Cluster 1	Cluster 18	Cluster 70	Cluster 0
AF271632	Cluster 17	Cluster 2	Cluster 18	Cluster 70	Cluster 0
D10749	Cluster 17	Cluster 1	Cluster 18	Cluster 70	Cluster 0
EF621489	Cluster 17	Cluster 2	Cluster 18	Cluster 70	Cluster 0
EU155213	Cluster 17	Cluster 1	Cluster 18	Cluster 70	Cluster 0
EU155214	Cluster 17	Cluster 1	Cluster 18	Cluster 70	Cluster 0
EU155215	Cluster 17	Cluster 2	Cluster 18	Cluster 70	Cluster 0
EU155216	Cluster 17	Cluster 1	Cluster 18	Cluster 70	Cluster 0
EU155233	Cluster 17	Cluster 1	Cluster 18	Cluster 70	Cluster 0
D49374	Cluster 101	Cluster 31	Cluster 97	Cluster 55	Cluster 10
JX227959	Cluster 35	Cluster 32	Cluster 34	Cluster 27	Unclustered
JX227960	Cluster 35	Cluster 32	Cluster 34	Cluster 27	Unclustered
AF238485	Cluster 2	Cluster 33	Cluster 9	Cluster 84	Cluster 6
MN385583	Cluster 145	Cluster 34	Cluster 140	Cluster 54	Cluster 10
KY348757	Cluster 147	Cluster 35	Cluster 142	Cluster 145	Unclustered
AB030907	Cluster 4	Cluster 36	Cluster 4	Cluster 76	Unclustered
AF238486	Cluster 4	Cluster 36	Cluster 4	Cluster 76	Unclustered
JX227964	Cluster 149	Cluster 37	Cluster 143	Cluster 115	Cluster 8
JX227978	Cluster 129	Cluster 38	Cluster 125	Cluster 143	Unclustered

Figure 4. Optional output of ViralClust in web-based format (HTML) for the dataset HCV. The interactive visualization allows the user to (i) sort the table by tool results or accession IDs, (ii) view and compare color-coded clusters, and (iii) access the NCBI entry for each sequence (link, if available). For most sequences, the clustering results of the different tools are consistent (top). Differences between the tools become apparent (bottom), where some sequences form single-sequence clusters (e.g. D49374), others consistently form small clusters across tools (e.g. AB030907 and AF238486), and individual sequences are not assigned to any cluster by specific tools, such as the HDBSCAN 'unclustered' sequences. The complete table is provided in the electronic supplement.

tools CD-HIT-EST, VSEARCH, and MMSeqs2 selected 18 representative genomes for genotype 6, whereas HDBSCAN identified substantially fewer representatives (3). These differences may be attributed to the distinct algorithmic strategies employed: HDBSCAN is based on k-mer similarity, while the other tools combine heuristic approaches with (pairwise) sequence alignments, which may increase sensitivity to finer sequence variation. Interestingly, SUMACLUST did not identify genotype 6 as a distinct cluster. This may be explained by the filtering step in SUMACLUST, which removes sequences containing non-ACGU characters and may have excluded genotype 6 genomes prior to clustering. In contrast, HDBSCAN selected eight representatives for subtype 6a, whereas the remaining tools selected only one. This difference may be influenced by the high abundance and relative homogeneity of subtype 6a genomes, in combination with the density-based clustering approach used by HDBSCAN. Several genomes in the dataset were labeled as recombinants leading to a split in the bottom right part of the tree (Fig. 3, dashed box) and potentially inflating the number of clusters. These recombinant genomes are annotated as a mix between genotypes 1 and 2. Overall, all tools except HDBSCAN produced broadly comparable results. While HDBSCAN tended to select more representatives in densely connected regions (e.g. genotype 6a), it selected fewer genomes in other parts of the tree. When examining individual sequences, they are usually assigned to the same cluster, although the chosen representative may differ slightly, see Fig. 4.

Genus Orthoflavivirus. The clustering of 8,052 *Orthoflavivirus* resulted in 63–101 clusters with the different tools, whereas MMSeqs2 serves with the smallest number of clusters and HDBSCAN with the highest, see Tab. 3. As for HCV, the number of clusters are higher than the number of species (53). The selection of more than one genome per species as representative can be sufficient to cover greater diversity across the whole genus of *Orthoflavivirus*. Despite minor differences, all five clustering algorithms produced broadly similar results, with representatives distributed across the phylogenetic representation of the dataset (see Fig. S5).

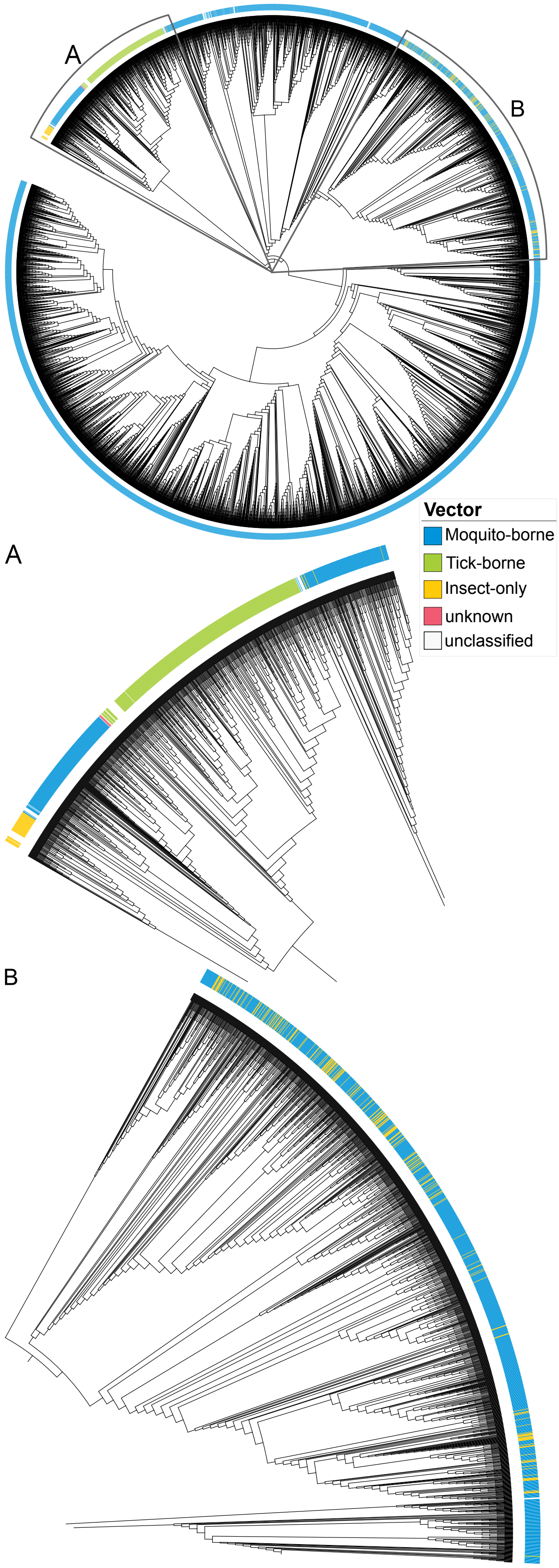

Figure 5. Phylogenetic representation based on the *Orthoflavivirus* dataset with color-coded vectors. Within the subtrees A and B, parts of the tree do not follow a vector-based classification. The complete and enlargeable tree is shown in Fig. S6. The tree is reconstructed using FastTree based on the MAFFT alignment of the complete genome sequences (7,681 genomes) resulting from the first filtering steps of ViralClust.

When comparing the selected representatives with the ICTV species assignments, which contains exactly one reference genome per species, see Fig. S4 violet background, we find (a) clusters which contain exactly one of the defining ICTV species, as expected; (b) ICTV species representatives not part of any cluster (= singletons); (c) clusters, which have no defining ICTV species, which might point to a generally more fine-grained clustering; and (d) some clusters have been merged by specific tools, so that two (or more) ICTV species could be found in one cluster, which should not have happened in order to represent the current phylogeny well.

The different tools found between 16 and 23 clusters containing exactly one ICTV species representative, see Tab. 4, whereas 18–23 ICTV reference genomes were not assigned to any cluster with more than one sequence, and thus remain as singletons within the ViralClust pipeline. This likely reflects recently added species for which only limited sequence data are currently available. An important detail is that SUMACLUST excludes any sequences containing characters other than A, C, G, or T. As a result, the ICTV representative genome for St. Louis encephalitis was removed from the dataset during preprocessing. Between 45 and 84 clusters contained no ICTV reference genome. These clusters likely represent intraspecific diversity or unclassified lineages, providing additional resolution beyond the current ICTV taxonomy. CD-HIT-EST, SUMACLUST, VSEARCH, and MMSeqs2 agreed to cluster two reference genomes of ICTV together into one cluster: Saboya (NC_033697) together with Potiskum (NC_029054). HDBSCAN also merged Saboya (NC_033697) together with other species, but in this case together with Modoc (NC_003635), Uganda S (NC_033698), and Wesselsbron (EU707555). For a more details see Tab. S3 and S4. However, if higher resolution is required, we recommend including singletons as well. In this case, users should be aware that incorporating singletons may lead to over-representation of certain regions within the dataset. HDBSCAN often adapts to the density of subtrees: for very dense clusters, it selects several representatives (see Fig. S5 top right), whereas clusters consisting of a single sequence are typically not chosen as representatives and instead remain unassigned. If such outliers are of interest for downstream analysis, users should manually include these singletons in subsequent steps (as these sequences are saved in a separate file). As a result, HDBSCAN yields the lowest average cluster size (69.19) and the largest number of singletons (693). An important observation concerns the previously proposed subclassification within *Orthoflavivirus* according to their vector type [30]. These analyses are based solely on parts of the the RdRp, see Fig. S4. Our analysis is based on complete genomes, indicating at least for subtrees, that the phylogeny can not be drawn based on the vector-type, see Fig. 5.

Table 4. Comparing the clustering results of the *Orthoflavivirus* dataset with the species chosen for the phylogeny of ICTV, see Fig. S4. ✓ – number of clusters with exactly 1 ICTV representative (in agreement with known phylogeny); Singleton – Number of clusters on containing one ICTV representative; ∅ – number of clusters without ICTV representative (showing additional information for downstream analysis); Discrepancies – (a) number of cluster with >= 2 ICTV representatives (e.g., '1x4' for four ICTV representatives in one cluster); (b) number of ICTV representatives that are 'singletons' (these sequences are alone a cluster); (c) other issues (e.g., SUMACLUST removes sequences including nucleotides $n \notin \{A, C, G, T\}$). For a more detailed overview see Tab. S3 and S4.

	✓	Singleton	∅	Discrepancies
cd	23	18	45	1×2
suma	20	20	50	1×2<br>St. Louis encephalitis (DQ525916) removed
vse	23	18	46	1×2
mmseq	23	18	39	1×2
hdb	16	23	84	1×4

Family Flaviviridae. Clustering of all 9,895 *Flaviviridae* species resulted in 180–221 clusters with SUMACLUST generating the lowest number of clusters and VSEARCH the highest, see Tab. 3. These results are twice as much as the reported 97 species from ICTV, Fig. S4, as for the subsets HCV species and *Orthoflavivirus* genus.

The average cluster size remains about the same for all five algorithms. MMSeqs2 produced the overall largest cluster (1,798 sequences), whereas HDBSCAN (1,371) and SUMACLUST (1,431) produced the smallest, with 200–400 fewer sequences. HDBSCAN also produced the largest number of singletons, with a notably high fraction (1,333), see Tab. 3. The ICTV phylogeny (Fig. S4) was constructed using the RdRp region of 125 sequences [30]. To evaluate how well this single-gene phylogeny reflects whole-genome evolutionary relationships, we compared it against a phylogeny inferred from 9,895 complete *Flaviviridae* genomes. While the overall topology of the full genome based phylogeny aligns broadly with the RdRp based ICTV classification, Fig. S7, several notable discrepancies emerged. Specifically, as visible in Fig. 6 (from left to right) (i) 25 genomes annotated as orthoflaviviruses clustered together in a distinct outlier subtree; (ii) 94 HCV genomes appeared more closely related to most of the pegiviruses than to other hepaciviruses; (iii) a single *Pestivirus* genome (Trinbago virus) was located within this divergent HCV cluster (not visible); (iv) another three, more divergent, *Pegivirus* sequences clustered with the majority of HCV; and (v) nine *Orthoflavivirus* genomes were placed nearer to basal pestiviruses (Fig. 6A – blue part between yellow and green). These discrepancies may be partly explained by misannotations in public sequence databases, but they also highlight the inherent limitations of single-gene phylogenies for robust virus taxonomy.

Comparing the datasets. We chose *Flaviviridae* on the family level (9,895 genomes) to test the robustness of the different clustering approaches, especially in comparison to clustering on the species level (HCV, 1,075 genomes true subset of *Flaviviridae*) and on the genus level (*Orthoflavivirus*, 8,052 genomes true subset of *Flaviviridae*), see Tab. 1. The fine-grained clustering observed at the species level is for some clustering algorithms also preserved at the family level, see Tab. 5. For example, within genomes of HCV, the methods CD-HIT-EST, SUMACLUST, and VSEARCH consistently formed the same clusters and selected the same representatives when compared between the HCV only and HCV clustered together with all other *Flaviviridae* datasets. MMSeqs2 shows slight differences in the selection of representatives, see Fig. S8. Clustering the entire *Flaviviridae* family with MMSeqs2 produced 56 clusters for HCV, compared to 55 clusters when only the HCV species was analyzed. Of these, 54 clusters were identical, while one cluster in the HCV clustering was split into two in the *Flaviviridae* analysis. Among the representatives, 49 were identical, and the remaining non-identical ones were phylogenetically close. In contrast, HDBSCAN showed larger differences in both cluster composition (only one identical cluster) and choice of representatives (six identical). When restricted to HCV, HDBSCAN produced twice as many clusters (23) compared to when HCV was analyzed as part of the *Flaviviridae* dataset (12). Genotypes 1a and 1b are represented by multiple genomes in the clustering of HCV, while these are grouped together in the results of *Flaviviridae*. This suggests that HDBSCAN is more sensitive to the chosen level and number of input sequences and adapts its clustering accordingly. When switching to the comparison on a genus level at the example of *Orthoflavivirus* only vs. *Orthoflavivirus* within *Flaviviridae*, all five algorithms generated the same largest cluster, containing dengue virus 1, see Tab. 3. SUMACLUST and VSEARCH again produced identical clusters and representatives, indicating robustness to the number of input sequences, see Tab. 5. For the genus *Orthoflavivirus*, CD-HIT-EST and MMSeqs2 selected the same total number of representatives – 69 and 63 respectively, as in the corresponding *Orthoflavivirus* subsets of the *Flaviviridae* analysis. While CD-HIT-EST produced identical cluster compositions, 8 representatives differ. Similarly, MMSeqs2 yielded 56 clusters with

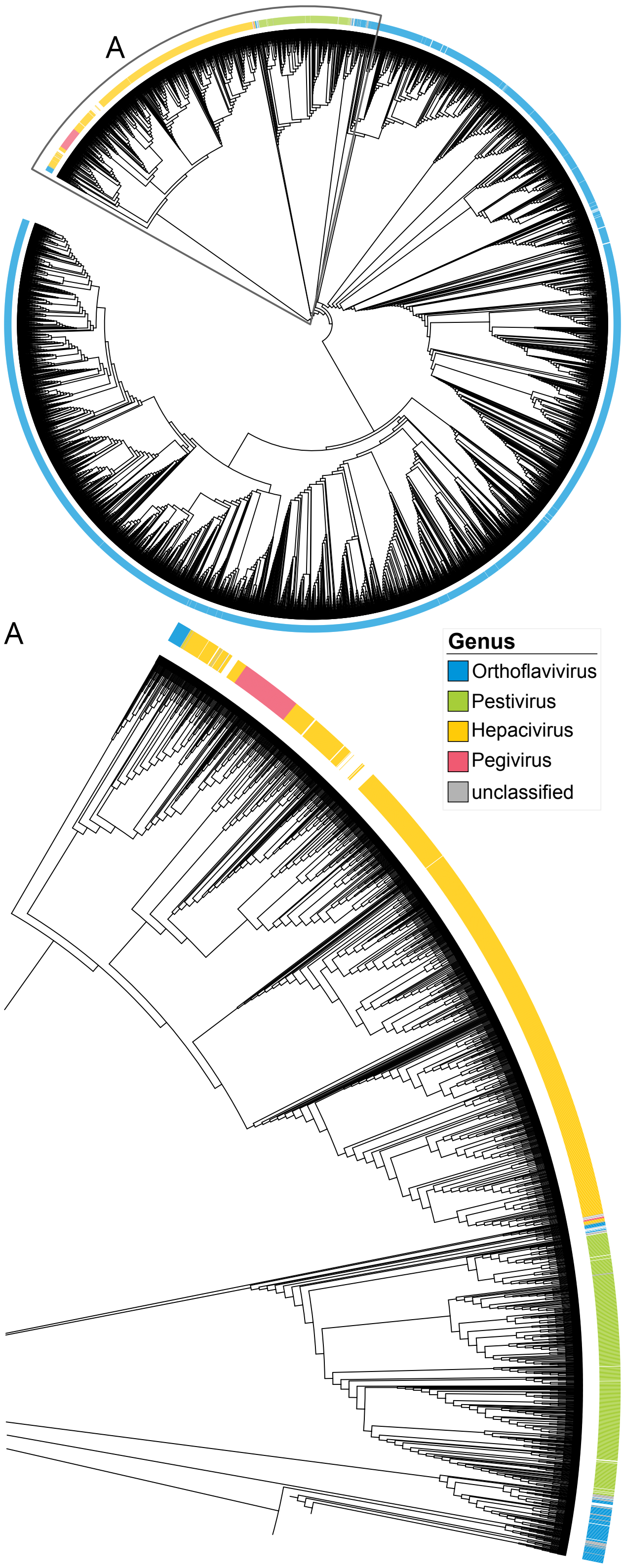

Figure 6. Phylogenetic representation of the *Flaviviridae* dataset with highlighted genera. A) parts of the tree that do not follow the classification by genus. Complete tree shown in Fig. S7. The tree is reconstructed using `FastTree` based on the `MAFFT` alignment of the complete genome sequences (9 478 genomes) resulting from the first filtering steps of `ViralClust`.

unchanged composition, but 48 identical representatives. In both cases, the differing representatives were phylogenetically close, and thus these discrepancies are considered minor (Fig. S9). In contrast, `HDBSCAN` generated substantially more clusters for *Orthoflavivirus* when clustering within the full *Flaviviridae* dataset (156 representatives) compared to clustering *Orthoflavivirus* alone (101 representatives). Of these, 27 clusters and 80 representatives were identical. The largest differences were observed in the mosquito-borne clade, where `HDBSCAN` selected considerably more representatives than the other algorithms (Fig. S9). The runtime does not scale linearly with dataset size, see Tab. S1. Although the *Orthoflavivirus* dataset accounts for 81 % of the *Flaviviridae* sequences (7,681 of 9,478), `ViralClust` required only 48.5 % of the total runtime (1 h 22 m of 2 h 47 m; Tab. 1). For the HCV dataset, the effect was less pronounced: comprising 11.3 % of the sequences in the *Flaviviridae* dataset, it required 8.3 % of the runtime (14 m of 2 h 47 m; Tab. 1). Memory consumption scales almost linearly when comparing the *Flaviviridae* dataset with the *Orthoflavivirus* dataset (183.9 G, 164.3 G; Tab. S1). In contrast, this is not the case for the HCV dataset, where memory usage is four times higher than for *Flaviviridae* when comparing with the size of the datasets.

Table 5. Comparison of the clustering results of *Hepacivirus hominis* only vs. *Hepacivirus hominis* within *Flaviviridae* and *Orthoflavivirus* only vs. *Orthoflavivirus* within the *Flaviviridae* analysis. clstr-comp. – cluster composition, i.e. number of identical clusters (number of cluster in *Hepacivirus hominis* or *Orthoflavivirus*/number of cluster in *Flaviviridae*); repr – identical representatives; ✓ – results are identical (id.). cd – `CD-HIT-EST`; sum – `SUMACLUST`; vse – `VSEARCH`; mmseq – `MMSeqs2`; hdb – `HDBSCAN`.

Hepacivirus hominis: only vs. within *Flaviviridae*		
	clstr-comp.	repr
cd	✓ (56 clstr)	✓
suma	✓ (31 clstr)	✓
vse	✓ (59 clstr)	✓
mmseq	54 id. (55/56)	49 id.
hdb	1 id. (23/12)	6 id.
Orthoflavivirus: only vs. within *Flaviviridae*		
	clstr-comp.	repr
cd	✓ (69 clstr)	57 id.
suma	✓ (71 clstr)	✓
vse	✓ (70 clstr)	✓
mmseq	56 id. (63/74)	48 id.
hdb	27 id. (101/158)	80 id.

Alphainfluenzavirus influenzae as an example for segmented viruses including partial and reverse complement sequences

Alphainfluenzavirus influenzae (formerly *Influenza A virus*, IAV) provides a representative case for studying segmented viral genomes and handling truncated reverse complement sequences. Here, we address several key questions relevant for sequence clustering and analysis of such viruses: We first confirm that genotype represents surprisingly well to serotype classification. For the eight genome segments, sequences can be analyzed individually or concatenated; here we show that independent analysis captures meaningful differences. Partial genomes are reliably placed in phylogenetic trees, demonstrating robustness. Finally, reverse complement sequences are detected through characteristic patterns (in this section highlighted with gray background); the current version of `ViralClust` automatically restores the positive orientation to simplify analysis.

IAV is a segmented (8 segments), negative-sense, single-stranded RNA virus belonging to the family *Orthomyxoviridae* (see Fig. S10). Classification is traditionally based on the two surface

proteins hemagglutinin (H, segment 4) and neuraminidase (N, segment 6), with 18 H and 11 N subtypes reported by the CDC (see Fig. S11 and S12). Our analyses confirm that segment data reflect subtype classification well, demonstrating that genotype approximates subtype and that segment-wise analysis captures meaningful biological differences.

Each of the eight genome segments in our dataset contains on average 98,532 sequences, with the largest datasets corresponding to segments 4 (156,586) and 6 (114,639) and the smallest to segment 2 (82,569). Segment lengths range from 890 nt (segment 8) to 2,341 nt (segments 1 and 2), and non-redundant sequence fractions vary from 64—69 % for segments 1–4 and 6 to 47–49 % for segments 7 and 8.

Clustering results (90 % sequence identity, k-mer length 7) vary widely depending on segment and method, ranging from 25 clusters (CD-HIT-EST, segment 7) to 2,307 (HDBSCAN, segment 4), see Tab. 1, with the largest datasets (segments 4 and 6) also yielding the highest number of clusters across all five tools. CD-HIT-EST produced the lowest number of clusters for all segments, followed by VSEARCH. MMSeqs2 and SUMACLUST are close to each other in terms of the number of clusters. MMSeqs2 yielded a lower number of clusters for segments 1, 2, 3, 5 and 6, but more for 4, 7 and 8. HDBSCAN resulted in the highest number of clusters by far. For segments 4 and 6, which encode the surface proteins, CD-HIT-EST, SUMACLUST, VSEARCH, and MMSeqs2 select representatives covering all known subtypes, see Tab. 6 and Fig. 7 and 9, whereas HDBSCAN misses certain subtypes (H17/H18 for segment 4, N10/N11 for segment 6).

Table 6. Number of representatives per subtype for segment 4 (H) and 6 (N). #repr - number of representatives (= number of clusters with a size greater than one); cd – CD-HIT-EST; sum – SUMACLUST; vse – VSEARCH; mmseq – MMSeqs2; hdb – HDBSCAN.

Subtype	#repr				
	cd	suma	vse	mmseq	hdb
**Segment 4 (H)**					
H1	83	352	128	527	613
H2	5	26	10	19	35
H3	34	215	59	460	654
H4	14	44	22	28	58
H5	27	145	43	107	210
H6	19	54	27	33	67
H7	16	37	22	23	100
H8	5	9	7	5	11
H9	44	193	71	242	347
H10	9	27	11	17	42
H11	7	16	10	13	19
H12	4	9	7	3	22
H13	8	20	11	13	21
H14	2	1	1	1	1
H15	2	2	2	2	2
H16	6	13	10	9	13
H17	1	1	1	1	0
H18	1	1	1	1	0
**Segment 6 (N)**					
N1	47	250	73	198	476
N2	90	280	130	276	733
N3	17	29	21	21	27
N4	6	15	10	5	17
N5	7	14	7	10	30
N6	15	45	24	33	94
N7	12	31	19	17	29
N8	20	52	30	26	103
N9	5	20	6	15	37
N10	1	1	1	1	0
N11	1	2	1	2	0

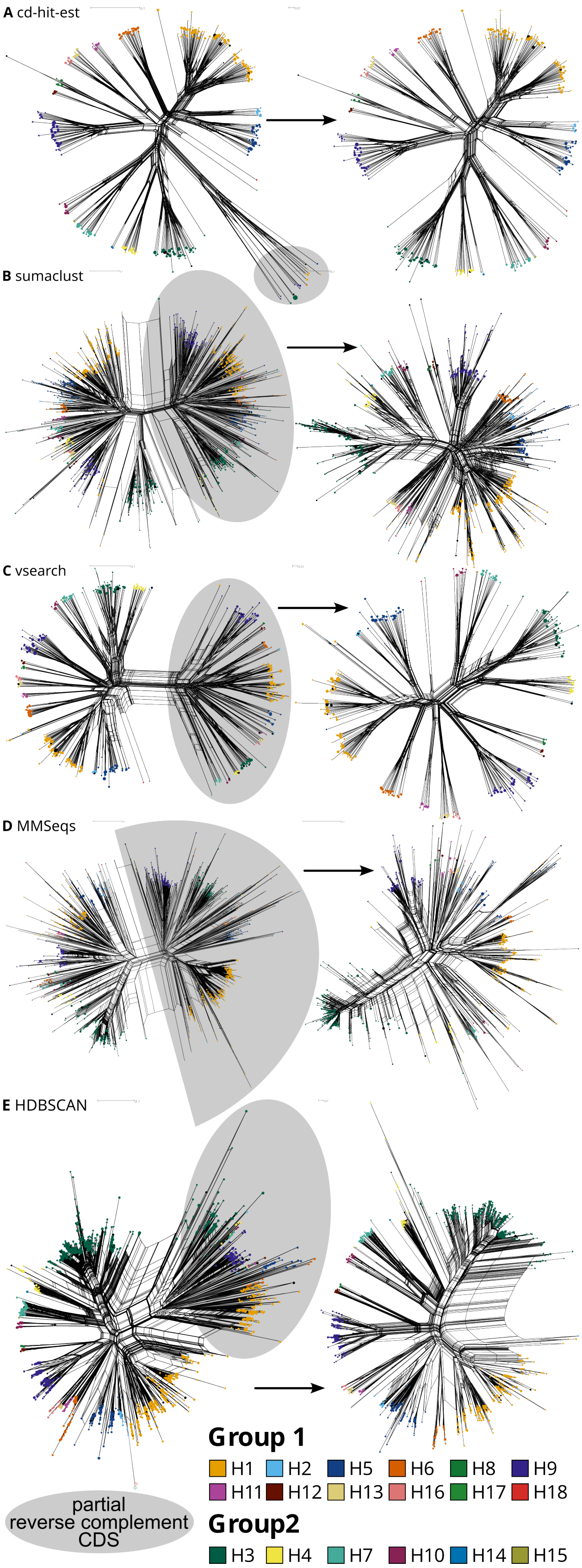

Figure 7. Split graphs of IAV segment 4 (H) representatives of (A) CD-HIT-EST, (B) SUMACLUST, (C) VSEARCH, (D) MMSeqs2, and (E) HDBSCAN. Left – all representatives (including partial and reverse complement); Right – only complete genomes. The node size indicates cluster size. The split graphs are reconstructed using SplitsTree based on the MAFFT alignments. HDBSCAN did not select representatives for subtypes H17 and H18. They were manually and marked them as framed circles. For details see Fig. S13–S17.

Partial genomes are reliably placed at the correct positions within phylogenetic trees, demonstrating the robustness of our approach even when sequences are incomplete. In each of the figures the truncated reverse-complement sequences are placed 'mirror-like' distinct in the phylogenetic tree. If the user observes such a behavior reverse complement sequences should be considered in the input dataset for the individual tools. Therefore, we implemented `ViralClust` to detect reverse-complement sequences and to automatically restore their original orientation, simplifying downstream analysis.

Given the variable results across tools and segments, we adjusted parameter ranges of `ViralClust` accordingly, with sequence similarity threshold between 70 and 90 %, see Tab. S5. CD-HIT-EST only allows for a minimum of 80 %. In the case of HDBSCAN, we tested k-mers from 1 to 7, see Tab. S6. As expected, the number of clusters decreases with decreasing similarity threshold for CD-HIT-EST, SUMACLUST, and VSEARCH (see Fig. 8 and S23). Important to note is, that the number of clusters resulting from `MMSeqs2` stays roughly the same or increase sometimes. We hypothesize, that the fast clustering approach `easy-linclust`, we chose for the `ViralClust` pipeline, is not suitable for such large amounts of data.

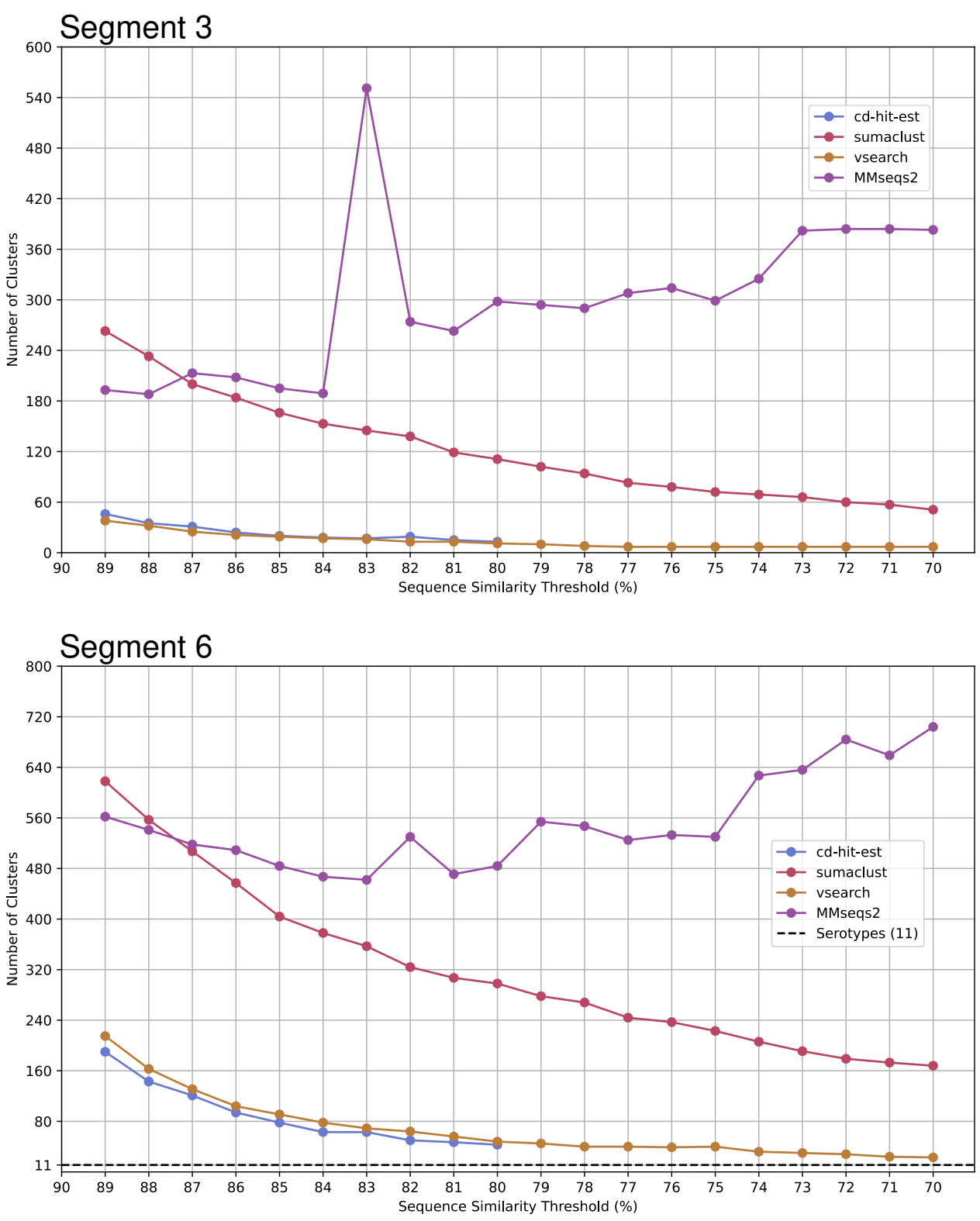

Figure 8. Number of clusters of IAV segments 3 and 6 with sequence similarity thresholds from 70 - 90 %. CD-HIT-EST only allows a minimum of 80 %. Only clusters with with a size greater than one are considered. For more details see Tab. S5 and S6.

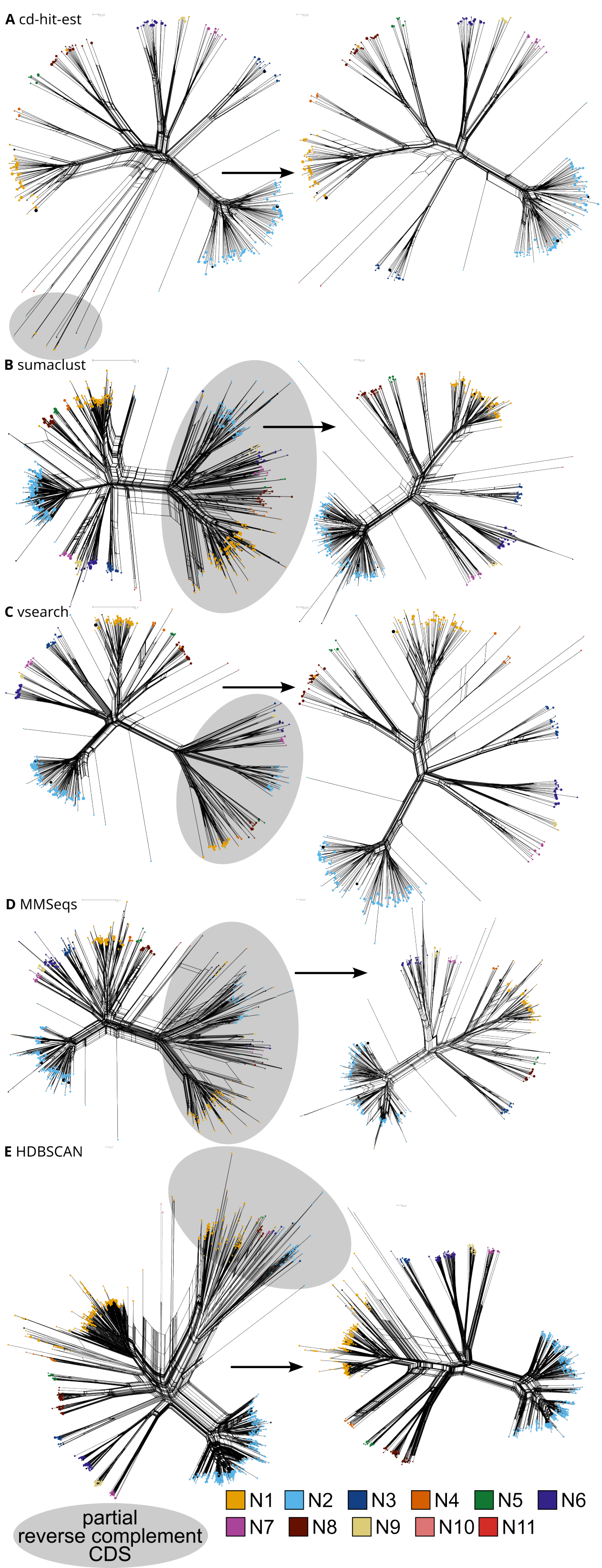

Figure 9. Split graphs of IAV segment 6 (N) representatives of (A) CD-HIT-EST, (B) SUMACLUST, (C) VSEARCH, (D) MMSeqs2, and (E) HDBSCAN. Left – all representatives (including partial and reverse complement); Right – only complete genomes. The node size indicates cluster size. The split graphs are reconstructed using `SplitsTree` based on the MAFFT alignments. HDBSCAN did not select representatives for subtypes N10 and N11. They were manually and marked them as framed circles. For details see Fig. S18–S22.

Monkeypox virus as DNA virus example

To assess the challenges posed by long, repetitive DNA genomes to clustering algorithms, we downloaded 1,077 *Monkeypox virus* genomes (family *Poxviridae*, see Fig S24), with an average length of 197.185 kbp. 92.76 % of the sequences are non-redundant. The main challenge compared to the RNA virus datasets analyzed previously is the combination of much longer genome length and repetitive regions, which can affect both clustering performance and representative selection.

For VSEARCH and CD-HIT-EST, we increased the parameters to accommodate the longer *Monkeypox virus* genomes: `--maxseqlength`

300000 (default: 50,000) and -M 2500 (default: 400). This dataset required the most time and memory overall. Based on the two known genotypes (clade I and II), we would expect two or more clusters. SUMACLUST, HDBSCAN, and VSEARCH produced between 1 and 3 clusters, whereas CD-HIT-EST (14) and MMSeqs2 (23) generated substantially more, see Tab. 1. Notably, SUMACLUST removed about 66 % (662 genomes) of the sequences during clustering, likely due to the presence of non-ACGT characters in repetitive regions. Although CD-HIT-EST generated more clusters, it assigned 917 sequences to a single cluster. In contrast, HDBSCAN and VSEARCH produced largest clusters containing 566 and 565 sequences, respectively. HDBSCAN successfully assigned all sequences to clusters, while the other algorithms left between 1 and 69 sequences unclustered (SUMACLUST and VSEARCH: 1, CD-HIT-EST: 10, MMSeqs2: 69). CD-HIT-EST and MMSeqs2 selected more representatives than the other tools. This difference likely reflects the underlying algorithmic principles: CD-HIT-EST uses a greedy incremental clustering approach that tends to select fewer representatives by aggressively merging similar sequences, whereas MMSeqs2 implements a more sensitive, iterative clustering procedure that preserves more distinct representatives, especially in large, repetitive genomes. The majority of the *Monkeypox virus* dataset consists of genotype IIb (see Fig. 10). The representatives selected by SUMACLUST and HDBSCAN reflect this dominant genotype, whereas VSEARCH representatives cover genotypes Ia and IIb. Both CD-HIT-EST and MMSeqs2 resulted in representatives covering all genotypes present in the dataset. The purpose of the phylogenetic representation is to assess whether the clustering tools can reconstruct the known genotype structure described in the literature, providing a visual benchmark for their performance on long DNA genomes.

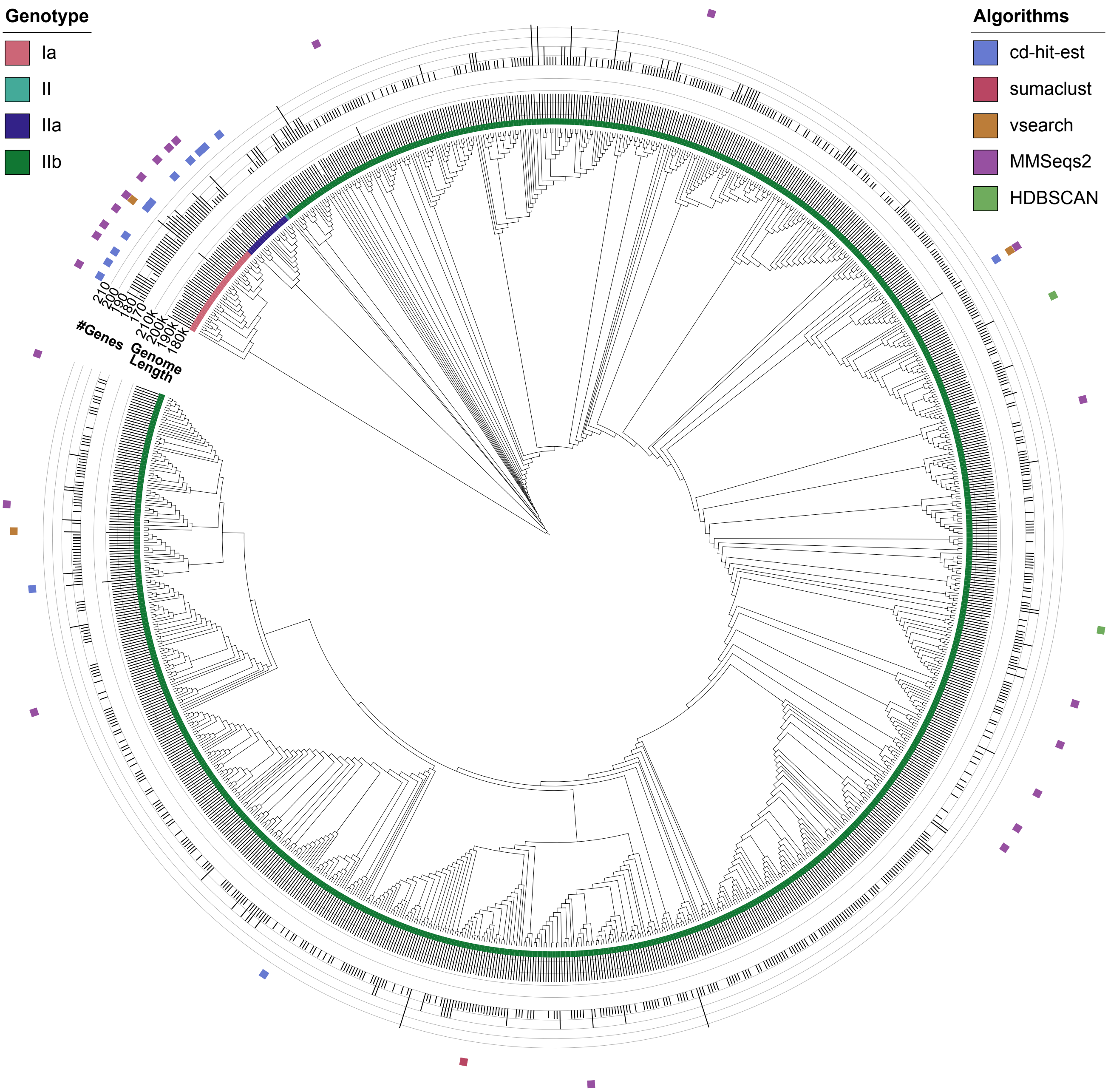

Figure 10. Phylogenetic reconstruction of the *Monkeypox virus* dataset with (i) genotypes, (ii) genome length, (iii) number of genes, and (iv) representative sequences selected by CD-HIT-EST, SUMACLUST, VSEARCH, MMSeqs2, and HDBSCAN. The tree is reconstructed using parsnp based on the complete genome sequences (999 genomes) resulting from the first filtering steps of ViralClust.

Discussion

The rapid increase in publicly available viral genome sequences poses substantial challenges for downstream analyses, including multiple sequence alignment, phylogenetic inference, and comparative genomics. Large and unevenly sampled datasets are often computationally infeasible to analyze in full and are prone to biases introduced by outbreak-driven over-representation and technical inconsistencies. In this study, we address these challenges by presenting `ViralClust`, a modular pipeline designed to reduce large viral genome collections through clustering-based representative selection while preserving genetic diversity.

A key outcome of our analyses is that clustering-based reduction provides a robust alternative to random subsampling. Across six diverse RNA and DNA virus datasets, representative genome sets retained biologically meaningful diversity while reducing dataset size by 95 % on average. Importantly, clustering results were stable across taxonomic scales: representative sets generated at the family level still captured genotypes defined at the species level, as demonstrated for *Hepacivirus hominis* and members of the *Flaviviridae*. This observation indicates that representative selection does not necessarily obscure finer-scale evolutionary structure and can support comparative analyses across broad taxonomic ranges. The evaluation further shows that clustering whole viral genomes is feasible across a wide range of genome sizes and architectures. Even large DNA virus genomes, such as those of *Monkeypox virus*, could be processed efficiently, demonstrating the scalability of the approach. In addition, dedicated pre-processing steps proved essential for obtaining interpretable clustering results. These findings emphasize that representative selection is not solely dependent on the clustering algorithm itself but also on careful data preparation and evaluation.

Rather than identifying a single optimal clustering strategy, `ViralClust` integrates multiple clustering algorithms based on different underlying principles, including compactness and connectedness. This design reflects the absence of a universal ground truth for (viral) clustering and acknowledges that different biological questions may require different clustering assumptions. By enabling direct comparison of clustering outcomes within a unified framework, `ViralClust` supports informed methodological choices without prescribing a "best" solution. In summary, this work demonstrates that scalable, clustering-based representative selection can substantially reduce viral genome datasets while preserving relevant diversity and mitigating common sources of bias. Through its modular design and integrated evaluation, `ViralClust` provides a flexible framework for virology research and serves as a reproducible starting point for downstream analyses of increasingly large viral genome collections.

Potential implications

Representative genome sets generated by `ViralClust` may facilitate a wide range of downstream analyses that are currently limited by dataset size or redundancy. For example, clustering-based representative selection can support the construction of high-quality multiple sequence alignments and enable RNA secondary structure prediction using representative alignments [44, 45, 46] as employed in Rfam [47], and large-scale analyses of endogenous viral elements [48, 49]. Similarly, workflows for virus–host prediction and comparative framework analyses, such as `VIDHOP` [50] and `POSEIDON` [51], may benefit from reduced yet diverse input datasets that remain biologically representative.

More broadly, whole-genome clustering may complement gene-centric approaches commonly used in viral comparative studies. While this work does not aim to redefine virus classification, our results suggest that genome-wide similarity patterns can provide additional context for studying viral diversity across taxonomic levels. We strongly advise that clustering-based representative selection be considered as a standard preprocessing step in future viral genome analyses, enabling more balanced, interpretable, and computationally tractable studies while remaining adaptable to specific research questions and analytical goals.

Methods

Tools integrated into `ViralClust`

We used existing tools for several steps in the `ViralClust` pipeline we designed.

Pre-processing. To remove 100 % redundant genomes in the input dataset, we apply the `easy-linclust` module of `MMSeqs2` (v14) [11, 12], with parameters `--min-seq-id 1.0`.

Compactness Clustering. Per default, `ViralClust` applies five different clustering methods: `CD-HIT-EST` (v4.8.1) [8], `SUMACLUST` (v1.0.31) [9], `VSEARCH` (v2.15) [10] from `vsearch`, `MMSeqs2` (v14) [11, 12], and `HDBSCAN` (v0.8.26) [17]. By default, `ViralClust` instructs `CD-HIT-EST`, `SUMACLUST`, `VSEARCH`, and `MMSeqs2` to cluster sequences with an identity of at least 90 % (default value of `CD-HIT-EST`).

Connectedness Clustering. We have implemented a combination of dimension reduction and clustering in `ViralClust`. For this we used `PCA` or `UMAP` (`umap-learn` v0.4.4) [52], both implemented in `scikit-learn` (v1.2.2) [22] for dimension reduction and `HDBSCAN` for clustering [17]. Per default, we use `PCA` and reduce the dimension to either $d = 50$ or until the sum of the explained variance of the components is at least 0.7. When using `UMAP`, the dimensions are reduced to $d = 50$ with a minimum distance of 0.25, cosine distance as common usage in the context of embeddings and language [53], and random seed state of 42 as used in `UMAP`'s documentation to ensure reproducibility within this stochastic algorithm[3]. `HDBSCAN` can be seen as a hybrid approach of hierarchical-based (connectivity) and density-based (compactness) clustering approaches. For two vectors, `HDBSCAN` first calculates mutual reachability distance. Per default, we use the cosine distance implemented in `SciPy` (v1.4.1) [23] as metric. However, we allow a plethora of pre-defined distances, which can be used via the `--metric` parameter of our `HDBSCAN` module. We implemented the (standardized) Cosine (=default), Euclidean, Manhattan, Chebyshev, Minkowski, Canberra, Braycurtis, and Mahalanobis distance metrics via the `SciPy` library [23]. To accommodate the limitations of the current implementation of `HDBSCAN`, we have employed L2 normalization to preprocess the vectors. This ensures compatibility with distance calculations, as cosine distance is not supported. Subsequently, we use `HDBSCAN` with the Euclidean distance on these normalized vectors[4].

Visualization

Importantly, with `ViralClust`, our aim is not to construct a phylogeny of the viruses, but rather to ensure that the selected virus genomes are distributed evenly across the existing phylogenetic reconstruction, providing a rough overview of their diversity. Therefore, for the validation of our use cases, we additionally visualized the distribution of cluster representatives and NCBI RefSeq genomes across the phylogenetic representation with the Neighbor-net algorithm implemented in `SplitsTree` (v4.19.2) [54, 55], generating split graphs, `FastTree` (v2.1.10) [26] as time-efficient tool for phylogenetic reconstruction of large datasets, and `parsnp`

3 www.umap-learn.readthedocs.io/en/latest/reproducibility.html
4 as communicated in: https://github.com/scikit-learn-contrib/hdbscan/issues/69#issuecomment-317742362

(v2.1.5) [56] for core-genome-based phylogenetic reconstruction (not part of the pipeline for the original data).

Availability of source code and requirements

`ViralClust` is available via GitHub: https://github.com/rnajena/viralclust.

Data availability

The raw sequences and datasets supporting the results of this article are available in the zenodo repository: https://doi.org/10.5281/zenodo.18413373.

Declarations

Ethical Approval

Not applicable

Consent for publication

Not applicable

Competing Interests

No competing interest is declared.

Funding

This work was supported by the Deutsche Forschungsgemeinschaft (DFG, German Research Foundation) under Germany's Excellence Strategy - EXC 2051 - Project-ID 390713860; the BMBF-funded project ADAPTI-M - Projetc-ID 031L0322H, and the Deutsche Forschungsgemeinschaft (DFG, German Research Foundation) under NFDI4Microbiota - NFDI 28/1 - Project-ID 460129525.

Author's Contributions

Sandra Triebel: Data curation; Investigation; Software; Formal analysis; Validation; Visualization; Writing – review & editing. Kevin Lamkiewicz: Data curation; Investigation; Methodology; Software; Formal analysis; Validation; Visualization; Writing – original draft Manja Marz: Supervision; Conceptualization; Validation; Writing – review & editing.

Acknowledgements

We thank the `Nextflow` fairy Marie Lataretu! We further thank Franziska Hufsky for her `InkScape` magic.

References

1. Sharma D, Priyadarshini P, Vrati S. Unraveling the Web of Viroinformatics: Computational Tools and Databases in Virus Research. J Virol 2014;89(3):1489–1501.
2. Paez-Espino D, Eloe-Fadrosh EA, Pavlopoulos GA, Thomas AD, Huntemann M, Mikhailova N, et al. Uncovering Earth's virome. Nature 2016;536:425–430.
3. Roux S, , Brum JR, Dutilh BE, Sunagawa S, Duhaime MB, et al. Ecogenomics and potential biogeochemical impacts of globally abundant ocean viruses. Nature 2016;537(7622):689–693.
4. Wilson WH, Gilg IC, Moniruzzaman M, Field EK, Koren S, LeCleir GR, et al. Genomic exploration of individual giant ocean viruses. The ISME Journal 2017;11(8):1736–1745.
5. Roux S, Adriaenssens EM, Dutilh BE, Koonin EV, Kropinski AM, Krupovic M, et al. Minimum Information about an Uncultivated Virus Genome (MIUViG). Nat Biotechnol 2018;37(1):29–37.
6. Hufsky F, Abecasis AB, Babaian A, Beck S, Brierley L, Dellicour S, et al. The International Virus Bioinformatics Meeting 2023. Viruses 2023;15(10):2031.
7. Göttsch W, Beerenwinkel N, Deng L, Dölken L, Dutilh BE, Erhard F, et al. ITN—VIROINF: Understanding (Harmful) Virus-Host Interactions by Linking Virology and Bioinformatics. Viruses 2021;13(5):766.
8. Li W, Godzik A. Cd-hit: a fast program for clustering and comparing large sets of protein or nucleotide sequences. Bioinformatics 2006;22:1658–1659.
9. SUMATRA and SUMACLUST: fast and exact comparison and clustering of sequences.;. http://metabarcoding.org/sumatra.
10. Rognes T, Flouri T, Nichols B, Quince C, Mahé F. VSEARCH: a versatile open source tool for metagenomics. PeerJ 2016;4:e2584.
11. Steinegger M, Söding J. MMseqs2 enables sensitive protein sequence searching for the analysis of massive data sets. Nature Biotechnology 2017;35(11):1026–1028.
12. Steinegger M, Söding J. Clustering huge protein sequence sets in linear time. Nat Commun 2018;9(1).
13. Expansion of the global RNA virome reveals diverse clades of bacteriophages. Cell 2022 Oct;185(21):4023–4037.e18.
14. SARS-CoV-2 variants evolve convergent strategies to remodel the host response. Cell 2023 Oct;186(21):4597–4614.e26.
15. Du Y, Xia J, Wang Z, Xu J, Ji Y, Jin Y, et al. Evolution of H6N6 viruses in China between 2014 and 2019 involves multiple reassortment events. Emerging Microbes & Infections 2024 Apr;p. 2341142.
16. Handl J, Knowles J, Kell DB. Computational cluster validation in post-genomic data analysis. Bioinformatics 2005;21(15):3201–3212.
17. McInnes L, Healy J, Astels S. HDBSCAN: Hierarchical density based clustering. J Open Source Software 2017;2(11):205.
18. Lefkowitz EJ, Dempsey DM, Hendrickson RC, Orton RJ, Siddell SG, Smith DB. Virus taxonomy: the database of the International Committee on Taxonomy of Viruses (ICTV). Nucleic Acids Research 2017 10;46(D1):D708–D717.
19. Walker PJ, Siddell SG, Lefkowitz EJ, Mushegian AR, Adriaenssens EM, Alfenas-Zerbini P, et al. Recent changes to virus taxonomy ratified by the International Committee on Taxonomy of Viruses (2022). Archives of Virology 2022;167(11):2429–2440.
20. Di Tommaso P, Chatzou M, Floden EW, Barja PP, Palumbo E, Notredame C. Nextflow enables reproducible computational workflows. Nat Biotechnol 2017;35(4):316.
21. Anaconda Software Distribution. Anaconda Inc.; 2020. https://docs.anaconda.com/.
22. Pedregosa F, Varoquaux G, Gramfort A, Michel V, Thirion B, Grisel O, et al. Scikit-learn: Machine Learning in Python. Journal of Machine Learning Research 2011;12:2825–2830.
23. Virtanen P, Gommers R, Oliphant TE, Haberland M, Reddy T, Cournapeau D, et al. SciPy 1.0: fundamental algorithms for scientific computing in Python. Nature methods 2020;17:261–272.
24. NCBI RC. Database Resources of the National Center for Biotechnology Information. Nucleic Acids Res 2017;45(D1):D12.
25. Katoh K, Standley DM. MAFFT multiple sequence alignment software version 7: improvements in performance and usability. Mol Biol Evol 2013;30(4):772–780.
26. Price MN, Dehal PS, Arkin AP. FastTree 2 – Approximately Maximum-Likelihood Trees for Large Alignments. PLoS ONE 2010;5(3):e9490.

27. Junier T, Zdobnov EM. The Newick utilities: high-throughput phylogenetic tree processing in the UNIX shell. Bioinformatics 2010;26(13):1669–1670.
28. JUKES TH, CANTOR CR. CHAPTER 24 - Evolution of Protein Molecules. In: MUNRO HN, editor. Mammalian Protein Metabolism Academic Press; 1969.p. 21–132.
29. Biedenkopf N, Bukreyev A, Chandran K, Di Paola N, Formenty PBH, Griffiths A, et al. ICTV Virus Taxonomy Profile: Filoviridae 2024. Journal of General Virology 2024;105(2).
30. Simmonds P, Becher P, Bukh J, Gould EA, Meyers G, Monath T, et al. ICTV Virus Taxonomy Profile: Flaviviridae. J Gen Virol 2017;98(1):2–3.
31. Simmonds P, Bukh J, Combet C, Deléage G, Enomoto N, Feinstone S, et al. Consensus Proposals for a Unified System of Nomenclature of Hepatitis C Virus Genotypes*. Hepatology 2005;42(4).
32. Smith DB, Bukh J, Kuiken C, Muerhoff AS, Rice CM, Stapleton JT, et al. Expanded classification of hepatitis C virus into 7 genotypes and 67 subtypes: Updated criteria and genotype assignment web resource. Hepatology 2014;59(1):318–327.
33. Borgia SM, Hedskog C, Parhy B, Hyland RH, Stamm LM, Brainard DM, et al. Identification of a Novel Hepatitis C Virus Genotype From Punjab, India: Expanding Classification of Hepatitis C Virus Into 8 Genotypes. The Journal of Infectious Diseases 2018;218(11):1722–1729.
34. Morel V, Descamps V, François C, Fournier C, Brochot E, Capron D, et al. Emergence of a genomic variant of the recombinant 2k/1b strain during a mixed Hepatitis C infection: A case report. Journal of Clinical Virology 2010;47(4):382–386.
35. Newman RM, Kuntzen T, Weiner B, Berical A, Charlebois P, Kuiken C, et al. Whole Genome Pyrosequencing of Rare Hepatitis C Virus Genotypes Enhances Subtype Classification and Identification of Naturally Occurring Drug Resistance Variants. The Journal of Infectious Diseases 2012;208(1):17–31.
36. Hostager R, Ragonnet-Cronin M, Murrell B, Hedskog C, Osinusi A, Susser S, et al. Hepatitis C virus genotype 1 and 2 recombinant genomes and the phylogeographic history of the 2k/1b lineage. Virus Evolution 2019;5(2):vez041.
37. Scheel TKH, Gottwein JM, Carlsen THR, Li YP, Jensen TB, Spengler U, et al. Efficient Culture Adaptation of Hepatitis C Virus Recombinants with Genotype-Specific Core-NS2 by Using Previously Identified Mutations. Journal of Virology 2011;85(6):2891–2906.
38. Li YP, Gottwein JM, Scheel TK, Jensen TB, Bukh J. MicroRNA-122 antagonism against hepatitis C virus genotypes 1–6 and reduced efficacy by host RNA insertion or mutations in the HCV 5' UTR. Proceedings of the National Academy of Sciences 2011;108(12):4991–4996.
39. Gottwein JM, Jensen SB, Serre SBN, Ghanem L, Scheel TKH, Jensen TB, et al. Adapted J6/JFH1-Based Hepatitis C Virus Recombinants with Genotype-Specific NS4A Show Similar Efficacies against Lead Protease Inhibitors, Alpha Interferon, and a Putative NS4A Inhibitor. Antimicrobial Agents and Chemotherapy 2013;57(12):6034–6049.
40. Lu L, Li C, Xu Y, Murphy DG. Full-length genomes of 16 hepatitis C virus genotype 1 isolates representing subtypes 1c, 1d, 1e, 1g, 1h, 1i, 1j and 1k, and two new subtypes 1m and 1n, and four unclassified variants reveal ancestral relationships among subtypes. Journal of General Virology 2014;95(7):1479–1487.
41. Wang B, Krüger L, Machnowska P, Eshetu A, Gunsenheimer-Bartmeyer B, Bremer V, et al. Characterization of a hepatitis C virus genotype 1 divergent isolate from an HIV-1 coinfected individual in Germany assigned to a new subtype 1o. Virology Journal 2019;16(1):28.
42. Jia Y, Yue W, Gao Q, Tao R, Zhang Y, Fu X, et al. Characterization of a Novel Hepatitis C Subtype, 6xj, and Its Consequences for Direct-Acting Antiviral Treatment in Yunnan, China. Microbiology Spectrum 2021;9(1).
43. Tokita H, Okamoto H, Iizuka H, Kishimoto J, Tsuda F, Miyakawa Y, et al. The entire nucleotide sequences of three hepatitis C virus isolates in genetic groups 7-9 and comparison with those in the other eight genetic groups. Journal of General Virology 1998;79(8):1847–1857.
44. Lamkiewicz K, Esquivel Gomez LR, Kühnert D, Marz M. Genome structure, life cycle, and taxonomy of coronaviruses and the evolution of SARS-CoV-2. Viral Fitness and Evolution: Population Dynamics and Adaptive Mechanisms 2023;p. 305–339.
45. Triebel S, Lamkiewicz K, Ontiveros N, Sweeney B, Stadler PF, Petrov AI, et al. Comprehensive survey of conserved RNA secondary structures in full-genome alignment of Hepatitis C virus. Scientific Reports 2024;14(1):15145.
46. Triebel S, Eulenfeld T, Ontiveros-Palacios N, Sweeney B, Tautz N, Marz M. First full-genome alignment representative for the genus *Pestivirus*. RNA 2025;.
47. Ontiveros-Palacios N, Cooke E, Nawrocki EP, Triebel S, Marz M, Rivas E, et al. Rfam 15: RNA families database in 2025. Nucleic Acids Research 2025;53(D1):D258–D267.
48. Ritsch M, Brait N, Harvey E, Marz M, Lequime S. Endogenous viral elements: insights into data availability and accessibility. Virus Evolution 2024;10(1):veae099.
49. Ritsch M, Eulenfeld T, Lamkiewicz K, Schoen A, Weber F, Hölzer M, et al. Endogenous Bornavirus-like Elements in Bats: Evolutionary Insights from the Conserved Riboviral L-Gene in Microbats and Its Antisense Transcription in *Myotis daubentonii*. Viruses 2024;16.
50. Mock F, Viehweger A, Barth E, Marz M. VIDHOP, viral host prediction with Deep Learning. Bioinformatics 2020;37(3):318–325.
51. Hölzer M, Marz M. PoSeiDon: a Nextflow pipeline for the detection of evolutionary recombination events and positive selection. Bioinformatics 2020 07;37(7):1018–1020.
52. McInnes L, Healy J, Melville J. UMAP: Uniform manifold approximation and projection for dimension reduction. arXiv 2018;.
53. Viehweger A, Krautwurst S, Parks DH, König B, Marz M. An encoding of genome content for machine learning. bioRxiv 2019;.
54. Bryant D. Neighbor-Net: An Agglomerative Method for the Construction of Phylogenetic Networks. Molecular Biology and Evolution 2003;21(2):255–265.
55. Huson DH, Bryant D. Application of Phylogenetic Networks in Evolutionary Studies. Molecular Biology and Evolution 2006;23(2):254–267.
56. Kille B, Nute MG, Huang V, Kim E, Phillippy AM, Treangen TJ. Parsnp 2.0: scalable core-genome alignment for massive microbial datasets. Bioinformatics 2024;40(5):btae311.
57. Likos AM, Sammons SA, Olson VA, Frace AM, Li Y, Olsen-Rasmussen M, et al. A tale of two clades: monkeypox viruses. Journal of General Virology 2005 Oct;86(10):2661–2672.
58. Bào Y, Amarasinghe GK, Basler CF, Bavari S, Bukreyev A, Chandran K, et al. Implementation of Objective PASC-Derived Taxon Demarcation Criteria for Official Classification of Filoviruses. Viruses 2017;9.
59. Kuhn JH, Amarasinghe GK, Basler CF, Bavari S, Bukreyev A, Chandran K, et al. ICTV Virus Taxonomy Profile: Filoviridae. The Journal of general virology 2019;100:911–912.

Click here to access/download
Supplementary Material
STriebel_ViralClust_supplementary_information.pdf
