# Supplementary material for: Reducing haystacks to needles—ViralClust: a Nextflow pipeline to cluster viral sequences
Source: Gigascience. 2026 Sep 14;15:giag090. doi: 10.1093/gigascience/giag090 (PMC13660479; doi:10.1093/gigascience/giag090)
Supplement: Supplementary file 4 [file giag090_giga-d-26-00034_revision_1.pdf]

GigaScience

Reducing haystacks to needles - ViralClust: A Nextflow pipeline to cluster viral sequences

--Manuscript Draft--

**Manuscript Number:**	GIGA-D-26-00034R1
**Full Title:**	Reducing haystacks to needles - ViralClust: A Nextflow pipeline to cluster viral sequences
**Article Type:**	Research
**Funding Information:**	Deutsche Forschungsgemeinschaft (Germany´s Excellence Strategy – EXC 2051 – Project-ID 390713860) — Dr. Manja Marz<br>Bundesministerium für Forschung und Technologie (ADAPTI-M - Projetc-ID 031L0322H) — Dr. Manja Marz<br>Deutsche Forschungsgemeinschaft (NFDI 28/1 - Project-ID 460129525) — M. Sc. Sandra Triebel
**Abstract:**	Background: The rapid accumulation of viral genome sequences presents major challenges for downstream analysis tools, including tools for multiple sequence alignments, phylogeny, and genome/alignment visualization, due to computational constraints and sampling biases caused by outbreak-driven over-representation. Selecting representative genomes through clustering offers a principled alternative to random subsampling, yet choosing appropriate clustering strategies remains non-trivial and context dependent.<br>Results: Here, we present ViralClust, a modular Nextflow pipeline for bias-aware representative selection from large viral genome datasets. ViralClust integrates five distinct clustering algorithms (CD-HIT-EST, SUMACLUST, VSEARCH, MMSeqs2, and HDBSCAN) within a unified workflow, enabling direct comparison of clustering outcomes and flexible adaptation to diverse biological questions, considering a balanced phylogenic distribution of the selected sequences. We evaluated ViralClust on six RNA and DNA virus datasets ranging from 632 to 156,586 sequences and spanning genome lengths from 890 to 197,185 nucleotides. Across all datasets, clustering reduced dataset size by 95% or more while preserving genetic diversity across species, genera, and families, and effectively mitigating biases introduced by outbreaks, partial genomes, and sequence orientation artifacts.<br>Conclusions: By supporting whole-genome clustering and scalable representative selection, ViralClust enables efficient and reproducible downstream analyses that would otherwise be computationally infeasible. Our framework provides a flexible foundation for large-scale viral genomics and supports future applications in comparative analysis and virus classification.
**Corresponding Author:**	Sandra Triebel<br>Friedrich Schiller University Jena: Friedrich-Schiller-Universitat Jena<br>Jena, GERMANY
**Corresponding Author Secondary Information:**	
**Corresponding Author's Institution:**	Friedrich Schiller University Jena: Friedrich-Schiller-Universitat Jena
**Corresponding Author's Secondary Institution:**	
**First Author:**	Sandra Triebel
**First Author Secondary Information:**	
**Order of Authors:**	Sandra Triebel<br>Kevin Lamkiewicz<br>Tom Eulenfeld<br>Manja Marz

Powered by Editorial Manager® and ProduXion Manager® from Aries Systems Corporation

**Order of Authors Secondary Information:**	
**Response to Reviewers:**	Dear Editor and Reviewers,<br><br>We would like to express our gratitude for your thoughtful and constructive comments on our paper titled "Reducing haystacks to needles - ViralClust: A Nextflow pipeline to cluster viral sequences".<br><br>In the following sections, we outline your specific comments and provide detailed explanations of the changes we have implemented to address each point. We have highlighted our answers as well as the additions in the manuscript in two colors (blue for reviewer one and magenta for reviewer two).<br><br>Reviewer 1<br>The authors present ViralClust, a modular Nextflow pipeline designed to address the computational challenges and sampling biases associated with the rapid accumulation of viral genome sequences. By integrating five distinct clustering algorithms (CD-HIT-EST, SUMACLUST, VSEARCH, MMSeqs2, and HDBSCAN), the pipeline offers a complete workflow from data pre-processing to representative sequence selection. The authors evaluated the tool on six viral datasets of varying size and type, including Orthoebolavirus, Hepacivirus, Influenza A, and Monkeypox virus.<br>This is a highly practical tool, particularly given the context of rapidly expanding viral genomic databases. The manuscript is well-structured, and the test datasets are representative, covering diverse viral characteristics. However, there is room for improvement regarding the analysis of specific algorithm performance, user guidance, and the presentation of results.<br><br>Specific Comments<br>1. Currently, ViralClust functions more as a "toolbox" of aggregated methods rather than a mature, user-friendly "solution." Although five clustering algorithms are provided, the manuscript lacks sufficient guidance on the specific application scenarios for each. I recommend that the authors design a comprehensive scoring metric to rank the clustering results of each algorithm, providing a reference for users. Alternatively, please provide recommended algorithms or parameter presets (profiles) tailored to different analysis scenarios (e.g., specific profiles for RNA viruses, DNA viruses, or segmented viruses). This would significantly aid users in selecting the most appropriate strategy.<br><br>We thank the reviewer for this valuable comment! We agree that guidance on tool selection is an important aspect of a user-friendly methods paper, and we have addressed this in two complementary ways.<br>First, we have incorporated a comprehensive scoring metric into the benchmarking of ViralClust, comprising five component scores - the Reduction Score (RS), Singleton Penalty Score (SPS), Clustering Quality Score (CQS), Taxonomic Concordance Score (TCS), and Cluster-to-Taxonomy Ratio (CTR) - aggregated into an Overall Score (OS) with a maximum of 5, shown in Table 2 and S3. These metrics are described in detail in the Methods section and applied across all six datasets presented in this study.<br>Second, rather than prescribing a single recommended tool, we use the scoring results together with the per-dataset observations to provide explicit guidance on the strengths and limitations of each algorithm under different biological scenarios. Specifically, based on our evaluation across RNA viruses, segmented viruses, and large DNA virus genomes, we discuss:<br>the tendency of compactness-based tools (CD-HIT-EST, SUMACLUST, VSEARCH) to produce fewer, more compact clusters, which is advantageous for maximal dataset reduction but may collapse rare lineages<br>the higher granularity of MMSeqs2, which better preserves sequence-level diversity at the cost of larger representative sets, with the caveat that its behavior becomes less predictable at very large dataset sizes<br>the suitability of HDBSCAN for datasets with continuous diversity distributions, while noting its fundamental limitation in detecting rare subtypes represented by few sequences, which may be missed as noise.<br>We believe this approach is more informative than fixed parameter presets, as the appropriate clustering strategy depends critically on the biological question, taxonomic scope, and dataset composition - all of which vary substantially across virology

research contexts. The scoring metric provides users with an objective, reproducible basis for making this choice with their own data, while the per-scenario discussion in the Results offers concrete guidance grounded in empirical evaluation.

2. The Results section is currently somewhat verbose and repetitive. As this is a Method/Tool paper, the core focus should be on demonstrating the tool's generalizability, accuracy, and the comparative logic between different algorithms, rather than a monotonous recitation of statistics for every dataset. Please consolidate the Results into 1-2 focused sections. Avoid listing exhaustive statistics for every case study and instead focus on summarizing trends and algorithmic differences.

We appreciate the reviewer's suggestion to streamline the Results section. However, because this is a methods/tool paper comparing clustering approaches, we believe that evaluating only overall generalizability or accuracy would not sufficiently capture the practical differences between algorithms. The performance and reliability of clustering methods strongly depend on the underlying biological question and dataset characteristics.
For this reason, we deliberately structured the Results around several representative scenarios that reflect common questions encountered in virology research. The six datasets were specifically selected to cover distinct biological and technical contexts, including: (1) a small and biased dataset, (2) analyses across different taxonomic levels (species, genus, and family), highlighting how tool behavior changes depending on context, (3) a segmented virus dataset, and (4) a large DNA virus dataset.
We therefore believe that retaining the separate sections is important to demonstrate not only overall performance trends, but also the reliability and limitations of each clustering approach under biologically relevant conditions. Nevertheless, in response to the reviewer's concern, we have revised the text to reduce repetitive reporting of statistics and instead emphasize the overarching trends and methodological differences between algorithms within each scenario.

3. The authors mention filtering out sequences containing less than 90% ACGT characters. Ambiguous bases (e.g., N, R, Y) are very common in consensus sequences, and strict filtering may be inappropriate. For instance, in the Monkeypox analysis, SUMACLUST discarded approximately 66% of the sequences, which is a significant loss of data. Consider relaxing this filter or modifying the strategy to calculate non-ACGT content only within Open Reading Frames (ORFs) to avoid discarding potentially valuable genomic data.

Thank you very much for your valuable comment and suggestion. Sequences containing more than 10% non-ACGT characters are filtered out before the actual clustering process begins. We initially opted for a fixed, high threshold to ensure higher data quality for clustering. We've integrated a parameter (--max_ambiguous) so the user can adjust this filtering process and mentioned this option in the main manuscript. You are absolutely right. SUMACLUST imposes very strict requirements on the input sequences and allows only ACGT characters. Since this is specific to the SUMACLUST algorithm, we cannot change this within our pipeline. Nevertheless, we have integrated SUMACLUST into our ViralClust pipeline because it is a widely used clustering tool. However, we have mentioned this limitation in the manuscript to provide readers and users with all the information they need to select the clustering results appropriate for their individual research questions.

4. The authors noted that the easy-linclust module of MMSeqs2 performed poorly on the large Influenza A dataset and hypothesized that easy-linclust is unsuitable for such data. Since ViralClust is explicitly aimed at handling "large-scale" data, it is insufficient to merely report this failure. I suggest the authors attempt to integrate other MMSeqs2 clustering modules, such as the easy-cluster, to see if results improve. If an alternative module performs better, it should be implemented as an alternative option or fallback within the pipeline.

We thank the reviewer for this constructive suggestion. We would like to clarify the rationale for our original choice of easy-linclust: the MMseqs2 user guide explicitly recommends it for large datasets, stating that "For larger datasets, easy-linclust offers an efficient clustering workflow, scaling linearly with input size.". Accordingly, easy-

Powered by Editorial Manager® and ProduXion Manager® from Aries Systems Corporation

linclust was selected as the default MMseqs2 module in ViralClust to ensure applicability on standard hardware, including laptops and workstations without high-memory compute environments.
Nevertheless, we acknowledge the reviewer's point that the unexpected behavior of easy-linclust on the IAV dataset, where cluster counts did not decrease monotonically with lower similarity thresholds, represents a relevant limitation for large-scale datasets and warrants a practical alternative. We have therefore implemented easy-cluster as an optional alternative to easy-linclust within the ViralClust pipeline. We have briefly tested the easy-cluster module on the IAV segment 4 dataset, and based on the number of clusters, easy-cluster might be a good alternative. The easy-linclust module remains the default for computational efficiency, while easy-cluster can be selected via a pipeline parameter (--mmseqs_module cluster) for users requiring more robust clustering behavior on large datasets. This has been noted in the manuscript accordingly.

5. The evaluation reveals a critical limitation in HDBSCAN, which tends to misclassify biologically significant but low-abundance lineages as noise—evidenced by the failure to identify TAFV and rare Influenza subtypes (H17/H18, N10/N11), alongside a disproportionately high generation of singletons (e.g., 1,333 in Flaviviridae). As the current requirement to manually recover these files undermines the pipeline's automation and poses a risk of data loss, I strongly recommend implementing a parameter (e.g., --keep_singletons) to automatically include unclustered sequences in the final representative output, while also expanding the Discussion/User guidance to explicitly warn users about the trade-offs of using density-based clustering for rare variant detection.

Currently, the results are divided into three Fasta files, and therefore the request of the reviewer is already implemented:
Cluster representatives with a cluster size > 1
Cluster representatives with a cluster size = 1 (singletons)
Cases 1 and 2 are combined into a single file.
The user can choose which file to use for the next steps of their research project. We have described this file generation in the text “Analyses - Determination of representative genomes” - “Clusters of size one are therefore not considered clusters, but named “singletons” here, and are stored in a separate file. The combined set of cluster representatives and “singleton” sequences is provided in a third file. This allows the user to choose which set of sequences is most suitable for the research question.“ We have highlighted this section in the main manuscript.

6. Please include the maximum memory consumption (Peak RAM) for each dataset in Table 1. While runtimes are provided, memory usage is a critical factor for users handling large datasets.

Thank you for this comment. We’ve integrated the maximum memory consumption (Peak RAM) in Table 1 and S1 for every dataset.

Reviewer 2
Sandra Triebel et al. build a modular Nextflow pipeline—ViralClust for clustering large viral genome datasets and selecting representative sequences in a bias-aware way. The pipeline integrates five clustering tools (CD-HIT-EST, SUMACLUST, VSEARCH, MMSeqs2, and HDBSCAN), offers optional evaluation outputs (MSA + rough phylogenetic representations), and produces HTML summaries and per-tool representative sets. While the pipeline sounds a good tool to addresses a genuine need in viral genomics, several major concerns regarding validation, methodological clarity, and practical implementation need to be addressed.

Major Comments
1. Although the author demonstrated that clustering preserves the phylogenetic topology, they did not adequately verify whether the selected representative strains retained biologically relevant traits such as host range, geographic distribution, temporal dynamics, or phenotypic characteristics.

We thank the reviewer for this valuable suggestion. We agree that the biological

relevance of representative selection extends beyond phylogenetic topology, and we have added a paragraph to the Discussion addressing this point.
We note that to the extent biologically relevant traits such as host range, geographic distribution, or temporal dynamics are reflected in genome sequence, clustering-based representative selection inherently captures this variation alongside genetic diversity. Furthermore, the degree to which a representative set covers features of interest will always depend on the underlying research question, and we encourage users to evaluate their representative sets with respect to the metadata most relevant to their specific study.
To illustrate this, we point to our previously published work on Pestiviruses, in which representatives selected by clustering were shown to adequately cover the host range present in the full dataset [1]. This example supports the biological relevance of clustering-based representative selection beyond phylogenetic structure alone, and we have now made this explicit in the revised Discussion.

[1] Triebel S, Eulenfeld T, Ontiveros-Palacios N, Sweeney B, Tautz N, Marz M. First full-genome alignment representative for the genus Pestivirus. RNA. 2026 Feb 17;32(3):311-327. doi: 10.1261/rna.080732.125. PMID: 41436245; PMCID: PMC12908451.

2. The author present results under various parameter combinations but does not adequately explain how users should select appropriate parameters based on specific research questions, which can be confusing for those unfamiliar with the context. For example, when should users use a 70% rather than a 90% sequence similarity threshold? How does this choice affect different downstream analyses?

We thank the reviewer for this important suggestion. We agree that parameter selection is one of the most practically relevant aspects of a clustering tool for users without a deep computational background, and we have addressed this in the revision in several ways.
First, we want to acknowledge that parameter selection in viral genome clustering is inherently context-dependent: the appropriate sequence similarity threshold is not a fixed value but depends critically on the biological question, the taxonomic scope of the dataset, and the evolutionary diversity of the viral clade under study. A 90 % threshold may be appropriate for within-outbreak subsampling of a highly conserved virus, where the goal is to collapse near-identical sequences while retaining all circulating variants. In contrast, a 70 % threshold may be more appropriate for family-level analyses spanning multiple genera, where the aim is to retain one representative per major lineage rather than per sequence cluster. Specifying a universally applicable threshold would therefore be misleading, as it would imply a level of generalizability that does not exist.
That said, we acknowledge the reviewer's point that users need more concrete guidance than "it depends". We have therefore addressed this in two complementary ways in the revision. First, we incorporated a comprehensive scoring framework comprising five component scores (RS, SPS, CQS, TCS, CTR) aggregated into an Overall Score (OS, maximum 5), which allows users to objectively compare clustering results across parameter settings and tools for their specific dataset. This scoring framework is described in the Methods section and applied across all six datasets in this study, providing empirical reference points for parameter choices under different biological scenarios. Second, we have expanded the Results section to explicitly discuss the effect of varying similarity thresholds for each dataset, highlighting where threshold choice had a strong effect on cluster number and representative selection, and where results were robust to parameter variation.
Together, these additions provide users with both a quantitative tool for parameter evaluation and concrete biological context for interpreting their results, without prescribing thresholds that may not generalize beyond the scenarios evaluated here.

3. Recombination occurs across multiple viral families, and the authors did not adequately explain how recombinant sequences are handled during the clustering process. Depending on the genomic regions used to calculate similarity, recombinant genomes may yield divergent clustering outcomes. The authors should discuss whether their method is applicable to highly recombinant viruses and clarify whether additional preprocessing steps (such as recombination detection or removal) are necessary.

We thank the reviewer for highlighting the important issue of recombination in viral genomes. We agree that recombinant sequences can substantially influence clustering outcomes, particularly because similarity-based approaches may produce different results depending on the genomic regions analyzed.
To specifically assess this aspect, we included Deformed wing virus (DWV), a highly recombinant virus, as one of the evaluated datasets. Our analyses showed that none of the tested clustering methods produced consistently robust or biologically stable results for this dataset. This observation highlights the challenges that recombination poses for sequence similarity–based clustering approaches in general and demonstrates an important limitation of the evaluated methods.
Our framework itself does not explicitly detect or correct for recombination events prior to clustering. Instead, clustering reflects the sequence relationships present in the input data and is therefore inherently influenced by the genomic composition of recombinant viruses. Consequently, highly recombinant viruses may exhibit unstable or context-dependent clustering patterns, especially when different genomic regions are analyzed separately.
We further note that this limitation is not unique to our approach, but broadly affects most similarity- and distance-based clustering strategies. The impact of recombination strongly depends on the biological question, the taxonomic level under investigation, and whether analyses are performed on complete genomes or selected genomic regions. For viral groups with frequent recombination, additional preprocessing steps such as recombination detection, masking, or restriction to conserved genomic regions may therefore be advisable prior to clustering.
Following the reviewer's valuable suggestion, we have expanded and improved the Discussion/Conclusion section to more explicitly address the applicability and limitations of clustering approaches for highly recombinant viruses and to clarify under which circumstances additional preprocessing may be necessary.

4. The author mentions that HDBSCAN is not suitable for large datasets and that MMseqs2 is faster but does not provide specific data on computational requirements under different dataset sizes and parameter combinations. For users handling ultra-large datasets, such information is very important for analysis planning.

Thank you for your comment. We have included information about computational requirements in section "Methods - Benchmarking and Scoring Metrics". Further information about time and memory usage is displayed in the supplementary Table S1. To better integrate this information into the main text, we have referenced this table in the caption for Table 1.

Minor Comments
1. The introduction would be more appealing if it briefly discusses existing methods for handling large-scale viral datasets (such as reference-based alignment and k-mer approaches) and explains why cluster-based representative sequence selection offers advantages in certain applications.

Thank you for making our manuscript stronger! We've included a brief description of existing methods in the Background section.

2. The table 4 comparing clustering results would be more informative if it included a column showing the percentage of total diversity captured by each method, perhaps using a metric like phylogenetic diversity (PD) or sum of branch lengths in the representative tree versus the full tree.

Thank you for this suggestion! We included the ratio of the phylogenetic distance (PD) of the full tree and the representative tree.

3. The split graphs of IAV segments showing partial and reverse complement sequences (Figure 7) are informative, but it would benefit from quantitative metrics on how many sequences were affected and how correction improved clustering results. Provide a table summarizing the number of sequences in each category before and after correction.

Powered by Editorial Manager® and ProduXion Manager® from Aries Systems Corporation

	We thank the reviewer for this suggestion. We have added Table S6 to the manuscript, summarising the number of representatives produced by each tool under three conditions for IAV segments 4 (H) and 6 (N): clustering the full non-redundant dataset including reverse-complement sequences, post-hoc removal of reverse-complement representatives from the original results, and full re-clustering after removing all reverse-complement sequences from the input.<br>The results confirm that reverse-complement sequences have a substantial quantitative impact on clustering outcomes, demonstrating that a considerable fraction of clusters in the uncorrected results arose from strand orientation artifacts rather than genuine sequence diversity. Comparable results between post-hoc correction and full re-clustering further support that ViralClust's automatic orientation correction prior to clustering is sufficient to avoid these artifacts without requiring a separate manual filtering step by the user.<br>The corresponding text has been added to the IAV subsection of the Results.<br><br>4. The Figure 8 showing the relationship between sequence similarity threshold and number of clusters are useful, but they only show results for segments 3 and 6. Either show results for all segments in supplementary materials or explain why these two segments were chosen as representatives.<br><br>We created these plots for all segments, but decided to include only segments 3 and 6 in the main manuscript, as these show the most divergent results. The results for the remaining segments are presented in the supplementary Figure S23.<br><br>5. Discussion the statement "Large and unevenly sampled datasets are often computationally infeasible to analyze", is somewhat vague. Specify what types of analyses become infeasible, such as multiple sequence alignment.<br><br>Thank you very much for this suggestion! We have included more detailed information in the discussion and highlighted it in the main text.<br><br>We are confident that our revised manuscript aligns more closely with the objectives of GigaScience, and we hope that it will merit your consideration for publication.<br><br>Sincerely,<br><br>on behalf of all authors<br>Manja Marz (corresponding author) and Sandra Triebel (first author)<br><br>manja@uni-jena.de<br>sandra.triebel@uni-jena.de<br><br>RNA Bioinformatics & High-Throughput Analysis Jena<br>Friedrich Schiller University Jena<br>Faculty of Mathematics and Computer Science<br>Jena, Germany<br>www.rna.uni-jena.de
**Additional Information:**	
**Question**	**Response**
Are you submitting this manuscript to a special series or article collection?	No
**Experimental design and statistics**<br><br>Full details of the experimental design and statistical methods used should be given in the Methods section, as detailed in our Minimum Standards Reporting Checklist. Information essential to interpreting the	Yes

data presented should be made available in the figure legends.<br><br>Have you included all the information requested in your manuscript?	
**Resources**<br><br>A description of all resources used, including antibodies, cell lines, animals and software tools, with enough information to allow them to be uniquely identified, should be included in the Methods section. Authors are strongly encouraged to cite Research Resource Identifiers (RRIDs) for antibodies, model organisms and tools, where possible.<br><br>Have you included the information requested as detailed in our Minimum Standards Reporting Checklist?	Yes
**Availability of data and materials**<br><br>All datasets and code on which the conclusions of the paper rely must be either included in your submission or deposited in publicly available repositories (where available and ethically appropriate), referencing such data using a unique identifier in the references and in the "Availability of Data and Materials" section of your manuscript.<br><br>Have you have met the above requirement as detailed in our Minimum Standards Reporting Checklist?	Yes
GigaScience has policies and guidelines in place for the use of generative AI-writing tools such as ChatGPT. If you have used such writing tools to assist with writing the manuscript this must be declared and cited in the text. Authors should not list AI-writing tools and other AI-assisted technologies as an author or co-author and should acknowledge that they are fully responsible for text	No

generated or refined by AI-writing tools.<p><br><br>A summary of use (particularly in the introduction or among methods) needs to be included at the end of the paper, and the outputs should also be included as a supplementary file hosted in GigaDB or other open repositories. Please read our guidelines for more information. <p><br><br>By submitting to GigaScience, you are aware of the journal's AI-writing tools policy, and if you have declared use of such tools below, you have acknowledged this where appropriate in your manuscript and have made a summary of use and outputs available. <p><br><b>AI-assisted writing tools have been used in the preparation of this manuscript?	

Manuscript

Click here to access/download;Manuscript;STriebel_ViralClust_revised.pdf

GigaScience, 2026, 1–S28

doi: xx.xxxx/xxxx
Manuscript in Preparation
Paper

PAPER

Reducing haystacks to needles – ViralClust: A Nextflow pipeline to cluster viral sequences

Sandra Triebel[1,2], Kevin Lamkiewicz[1,2], Tom Eulenfeld[1] and Manja Marz[1,2,*]

[1]RNA Bioinformatics and High-Throughput Analysis, Friedrich Schiller University Jena, Jena, 07743, Germany and [2]European Virus Bioinformatics Center, Friedrich Schiller University Jena, Jena, 07743, Germany

[*] manja@uni-jena.de

Comments of Reviewer 1, Comments of Reviewer 2

Abstract

Background The rapid accumulation of viral genome sequences presents major challenges for downstream analysis tools, including tools for multiple sequence alignments, phylogeny, and genome/alignment visualization, due to computational constraints and sampling biases caused by outbreak-driven over-representation. Selecting representative genomes through clustering offers a principled alternative to random subsampling, yet choosing appropriate clustering strategies remains non-trivial and context dependent.
Results Here, we present `ViralClust`, a modular `Nextflow` pipeline for bias-aware representative selection from large viral genome datasets. `ViralClust` integrates five distinct clustering algorithms (`CD-HIT-EST`, `SUMACLUST`, `VSEARCH`, `MMSeqs2`, and `HDBSCAN`) within a unified workflow, enabling direct comparison of clustering outcomes and flexible adaptation to diverse biological questions, considering a balanced phylogenic distribution of the selected sequences. We evaluated `ViralClust` on six RNA and DNA virus datasets ranging from 632 to 156,586 sequences and spanning genome lengths from 890 to 197,185 nucleotides. Across all datasets, clustering reduced dataset size by ~95 % or more while preserving genetic diversity across species, genera, and families, and effectively mitigating biases introduced by outbreaks, partial genomes, and sequence orientation artifacts.
Conclusions By supporting whole-genome clustering and scalable representative selection, `ViralClust` enables efficient and reproducible downstream analyses that would otherwise be computationally infeasible. Our framework provides a flexible foundation for large-scale viral genomics and supports future applications in comparative analysis and virus classification.

Key words: Virus; Clustering; Nextflow; Bioinformatics; Viral Evolution

Background

Viral genome sequences are becoming increasingly available [1, 2, 3, 4, 5]. For example, for SARS-CoV-2 alone, nearly 9.2 million uploaded nucleotide sequences are accessible (NCBI Virus, 19.11.2025). The use of such large datasets of viral sequences often poses problems for downstream analyses, such as sequence alignment, phylogeny, and visualization, in terms of memory usage, run time, and visualization [6, 7]. Calculating a multiple sequence alignment (MSA) of available viral genomes of a family is often computationally not feasible. Disregarding the technical demands, an MSA with tens of thousands of entries may not be informative for most users, since the overall alignment quality potentially decreases. Several strategies have been proposed to handle large-scale viral datasets. Reference-based alignment approaches map sequences against a single reference genome, reducing computational overhead but potentially missing diversity in divergent strains [8, 9, 10]. Similarly, k-mer-based methods enable rapid, alignment-free comparison of sequences and have been widely used for taxonomic classification and similarity estimation [11, 12, 13]. However, both approaches are primarily designed for sequence comparison rather than dataset reduction, and neither directly addresses the problem of selecting a representative, diversity-preserving subset of genomes for downstream analysis. Besides the size of the datasets, the over-representation of a certain species due to an outbreak might also introduce a bias into the data. To reduce the number

Compiled on: June 2, 2026.
Draft manuscript prepared by the author.

Key Points

- ViralClust is a modular Nextflow pipeline for selecting representative viral genomes from (very) large sequence datasets.
- It combines multiple clustering approaches to reduce dataset size while minimizing bias and preserving genetic diversity.
- Tests on six RNA and DNA virus datasets show reductions of ~95 % across a wide range of genome sizes and sequence counts.
- ViralClust enables efficient and reproducible downstream analyses that are otherwise impractical with full viral genome collections.

of entries, random sampling of genomes does not solve the introduced bias of over-represented species it may even increase the effect of the bias. Consequently, the aim is to reduce the dataset of viral sequences without losing genetic diversity. In this context, cluster-based representative sequence selection offers a distinct advantage: by grouping sequences according to their similarity and retaining one representative per cluster, the resulting subset reflects the full breadth of genetic diversity present in the original dataset, regardless of sampling biases introduced by outbreaks or sequencing efforts. Instead of random selection, clustering viral genomes based on, e.g., sequence similarity and selecting a representative genome for each cluster results in a better-balanced subset of input viruses. While the idea of clustering is simple, the correct method is often hard to find and depends on the underlying scientific question. Depending on the biological question and distribution of biological data, the principle of *compactness* is most widely used, aiming to keep intra-cluster variation small. It is implemented in tools such as CD-HIT-EST [14], SUMACLUST [15], VSEARCH [16], and MMSeqs2 [17, 18]. In this context, the sequence identity determines whether a sequence belongs to a cluster or forms a new, independent cluster. Thus, compactness clustering is based on sequence homology. In most implementations, a sequence is only compared against one representative of established clusters. This method is used frequently for viral clades [19, 20, 21]. However, evolution is a continuous process, and many novel observed viruses might not be unambiguously assigned to the calculated clusters based on static sequence similarity thresholds alone. An algorithm following the principle of *connectedness* may be suitable for such continuous data. The principle of connectedness exploits the idea that neighboring data points should belong to the same cluster. Therefore, algorithms following this principle tend to detect smaller, local components within the data that are not concentrated around a center point [22]. For datasets with viral genomes such that the data points are not well-described by high-dimensional spheres, as is the case for compactness clusters, we propose a tool such as HDBSCAN [23]. Virologists, mostly lacking computational backgrounds, often find themselves unable to navigate the intricacies of algorithmic advantages and disadvantages, leaving them uncertain about selecting the most suitable tool for their analyses. For bioinformaticians, evaluating which tool to choose presents its own challenges, as the absence of a definitive ground truth complicates the determination of which sequences best represent a cluster.

Here, we present ViralClust, a Nextflow pipeline designed to select representative viral genomes, by a streamline clustering analysis for virology research. Unlike conventional methods, ViralClust integrates clustering via five distinct existing tools, enabling direct and comprehensive comparison of the results. One key feature of ViralClust is its robust data pre-processing capabilities. We address common database errors, such as inconsistencies in sequence orientation (-ssRNA sequences found in both positive and negative orientations) and ambiguous gene arrangements in ambisense viruses. By correcting these mistakes, we enhance the accuracy of clustering outcomes. Moreover, ViralClust introduces innovative methodologies previously unexplored in virology research. For instance, full genome clustering based on k-mers of nucleotide sequences utilizing Hierarchical Density-Based Clustering (HDB) represents a novel approach in virus research, offering previously inaccessible insights. Finally, we provide comprehensive statistics, including cluster sizes, and visualize the sequence selection in rough phylogenetic representations without explicitly generating phylogenies of the viral families. Additionally, our HTML output facilitates easy cluster sorting, including direct links to corresponding NCBI genomes for further analysis. All parts of the developed pipeline are written modular, and the user may adjust parameters or exchange and add complete tools. ViralClust offers the possibility of adding manually additional viral genomes of interest (GOI) to the input data. We evaluated the performance of ViralClust on six different datasets of RNA and DNA viruses varying in number of available sequences, sequence length, and sequence diversity, such as segmentation and orientation.

Data Description

Viral data

We downloaded viral genomes labeled as 'complete' of *Orthoebolavirus*, *Hepacivirus hominis* (HCV), *Orthoflavivirus*, *Flaviviridae*, *Alphainfluenzavirus influenzae* (formerly *Influenza A virus*, IAV), and *Monkeypox virus* from the NCBI Virus database, see Tab. 1. All sequences used in this study are deposited in the zenodo repository (Link: https://doi.org/10.5281/zenodo.18413373) in fasta format.

'Ground Truth' of Virus Phylogeny

To compare the results of ViralClust, we considered the classification of the ICTV [24, 25] as 'ground truth' for *Orthoebolavirus*, *Orthoflavivirus*, *Flaviviridae*. For *Hepacivirus hominis* and *Monkeypox virus* we included the NCBI taxonomy. The number of genera, subgenera and/or species is derived from https://ictv.global/taxonomy, see Tab. S2. For *Alphainfluenzavirus influenzae* we included the classification according to the Centers for Disease Control and Prevention (CDC)[1] into 18 different H subtypes and 11 different N subtypes. This theoretically results in 198 possible subtypes, but 136 subtypes are currently listed in the NCBI taxonomy (23.01.2026).

Analyses

The ViralClust pipeline

Our pipeline ViralClust takes a multiple fasta file as input and applies five different cluster tools to the data to select representative genomes. We calculate and report basic statistics for each of the cluster tools. Based on the respective representative genomes, ViralClust further calculates a multiple sequence alignment (MSA) and a rough phylogenetic representation (parameter --eval). Fig. 1 gives a general overview of the pipeline. Additionally to the clustering step, the user can retrieve an optional basic evaluation of the

1 https://www.cdc.gov/flu/about/viruses-types.html Last Reviewed: 23.01.2026

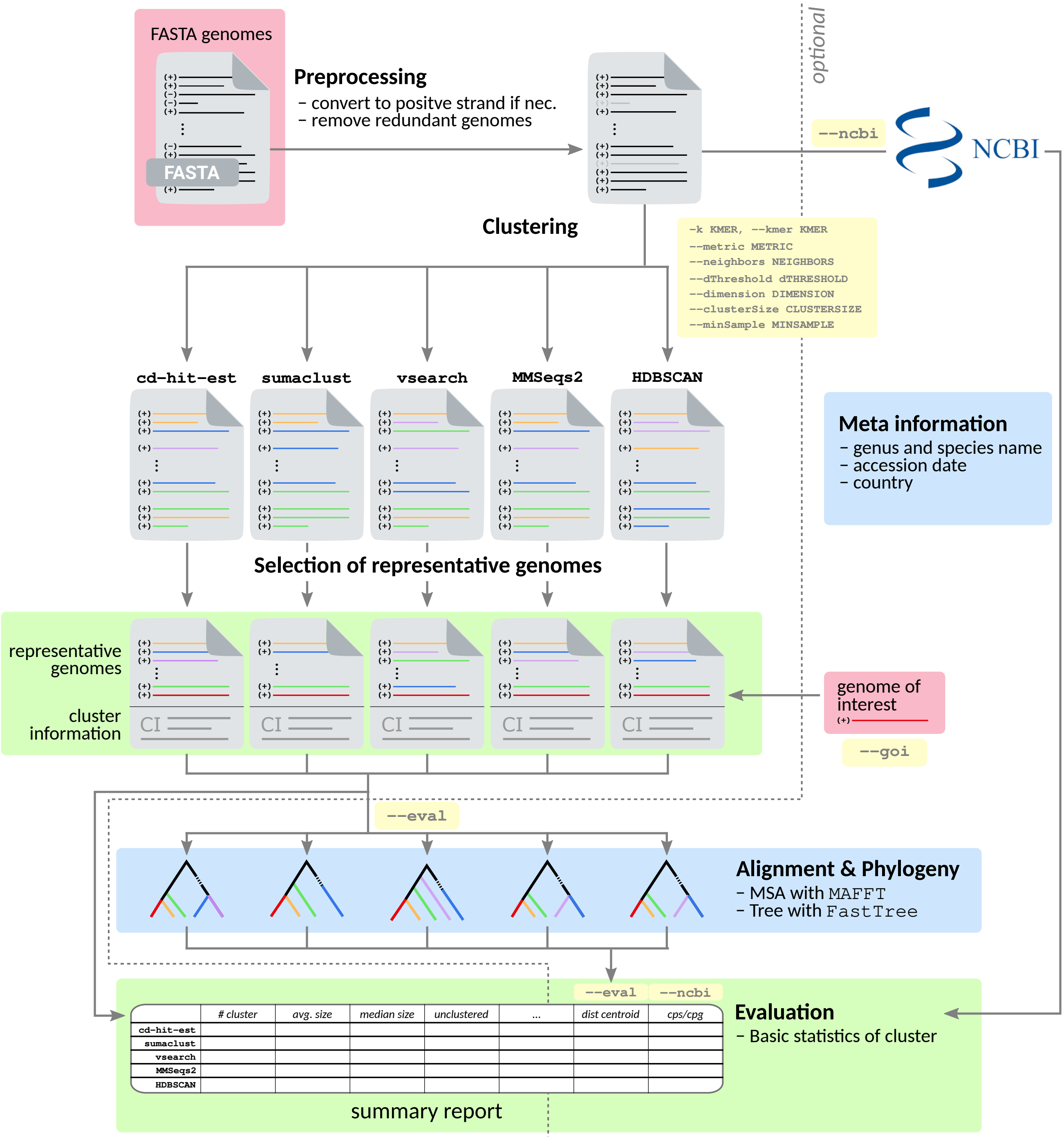

Figure 1. The general `ViralClust` pipeline. *Pre-processing.* Input genomes are filtered for redundant genomes and oriented to the positive strand determined via an ORF density scan. *Clustering.* Five different tools are pre-installed. However, each can be exchanged by other customized clustering tools. For each clustering method applied, `ViralClust` provides an overview of which sequence falls into which cluster. *Representative genomes.* Further, a `fasta` file with representative sequences for each cluster is provided for each tool. `--goi` – Genomes of interest (GOI) or unpublished genomes can be added to the representative genomes. *Evaluation.* The basic statistics of the cluster results, including average and median cluster size and the average distance of two representative genomes are given for each cluster tool. *Optional parameters.* `--ncbi` – For each applicable sequence, *i.e.* for each `fasta` record with a valid GenBank accession ID in its identifier, `ViralClust` retrieves additional information from the NCBI database and includes it in the summary report. `--eval` – For each clustering method `ViralClust` can provide an alignment of representative genomes and a phylogenetic representation of these genomes.

results. This evaluation is no recommendation for any algorithm or tool, as the choice of clustering depends on the underlying scientific question. However, it gives a broad overview of the data and may help determine the appropriate cluster method. If applicable, `ViralClust` finds GenBank accession IDs in the `fasta` records identifier and retrieves meta information from the NCBI database. Lastly, the user can specify a second, optional `fasta` file containing genomes that must be present in the final set of representative genomes. This may prove useful if comparative analyses between viruses are carried out and the user wants to include a specific, unpublished viral strain or isolate in the analysis.

Workflow of `ViralClust`

We implemented `ViralClust` in `Nextflow` (v22.10.06) [26]. We developed our scripts with `Python` (compatible with v3.6+). We employ individual `conda` (v4.9.2) [27] environments for each step per default

to guarantee no conflicts during installation and usage. ViralClust is publicly available at https://github.com/rnajena/viralclust. We further provide a test dataset with a small tutorial on using ViralClust. In the following, we describe the pipeline depicted in Fig. 1 in detail, including implemented tools and parameter configurations.

Pre-processing. To ensure high-quality data for subsequent clustering, ViralClust discards sequences with more than 10 % of non-ACGU characters. The user can adjust this filtering process using the --max_ambiguous parameter. Both strands are then scanned for ORF density to ensure that all sequences have the same orientation. Especially in the case of single-stranded negative RNA viruses, genomes are sometimes uploaded into the database in different orientations. This causes cluster algorithms to separate genomes based on this orientation, potentially jeopardizing the complete clustering approach. We calculate ORF density by translating an input genome into all six reading frames. We define an open reading frame (ORF) as a sequence of at least 200 amino acids between two stop codons or between a single stop codon and the 5' or 3' end of the sequence. This makes the procedure robust against missing stop codons at the end of the sequence and missing start codons. We calculate the forward-strand ORF density by summing the lengths of the ORFs in the three forward reading frames, then dividing by the sequence length. The backward-strand ORF density is calculated analogously. This method accounts for real or erroneous frameshifts. We use the strand with the higher ORF density for further calculations. The step is enabled by default; the user can disable it using the parameter sort_off. To remove 100 % redundant genomes in the input dataset, we apply the easy-linclust module of MMSeqs2 (v14) [17, 18], with parameters --min-seq-id 1.0.

Clustering. Per default, ViralClust applies five different clustering methods. Depending on which scientific virological question is asked, one of the tools might be more suitable than another. Due to the modularity of the pipeline, the user can also choose to switch off the predefined cluster tools and/or add individual cluster methods. Currently, we implemented CD-HIT-EST (v4.8.1) [14], SUMACLUST (v1.0.31) [15], VSEARCH (v2.15) [16] from vsearch, MMSeqs2 (v14) [17, 18], and HDBSCAN (v0.8.26) [23] as individual Nextflow modules. Whenever applicable, we set the parameters of the tools to match each other to make them comparable. By default, ViralClust instructs CD-HIT-EST, SUMACLUST, VSEARCH, and MMSeqs2 to cluster sequences with an identity of at least 90 % (default value of CD-HIT-EST). Since MMSeqs2 offers a variety of clustering modules, we have integrated easy-linclust (default) and easy-cluster.

Connectedness Clustering with HDBSCAN. As a novel approach to cluster viral genomes, we further implemented a combination of PCA and HDBSCAN [23] in ViralClust. *(1) k-mer distributions:* For this approach, we translate each input genome into a vector of k-mer frequencies (default $k = 7$), neglecting k-mers consisting of non-canonical nucleotides. *(2) Dimension reduction:* For the PCA, we use the module as implemented in scikit-learn (v1.2.2) [28]. Instead of the dimension reduction with PCA, we further allow using UMAP for the reduction. *(3) Clustering:* For efficient clustering, we reduce the resulting 4^k-dimensional (default $k = 7$) vector space and thus provide an embedding for the clustering algorithm HDBSCAN. Per default, we either reduce the dimension to $d = 50$ or until the sum of explained variance of the components is at least 0.7. After dimension reduction, each viral genome is represented by a vector of maximal length d ($d = 50$). HDBSCAN can be seen as a hybrid approach of hierarchical-based (connectivity) and density-based (compactness) clustering approaches. For two vectors, HDBSCAN first calculates mutual reachability distance. Per default, we use the cosine distance implemented in SciPy (v1.4.1) [29] as metric. However, we allow for a plethora of pre-defined distances, which can be used via the --metric parameter of our HDBSCAN module. We implemented the (standardized) Euclidean, Manhattan, Chebyshev, Minkowski, Canberra, Braycurtis, and Mahalanobis distance metrics via the SciPy library [29]. Since the used implementation of HDBSCAN cannot deal with the cosine distance, we normalized the vectors using the L2 normalization. Subsequently, we use HDBSCAN with the Euclidean distance on these normalized vectors[2].

The user can override or provide additional parameters for each tool via the nextflow run viralclust.nf command. Exemplarily, for HDBSCAN, the user can use the flag --hdbscan_help to get an overview of implemented options, which can be used using --hdbscan_params. Analogously, help messages and parameters for the other tools can be used and accessed via their respective commands.

Determination of representative genomes. The implemented tools report representative genomes based on sequence length (CD-HIT-EST, SUMACLUST), sequence abundance (VSEARCH), and selection within cascaded clustering (MMSeqs2). For the HDBSCAN module, we calculate centroid sequences based on the average pairwise distance between all vectors in the same cluster. The sequence with the minimum average distance is selected as the representative genome for this cluster. Given a distance metric d, the centroid sequence c of a cluster C with size N is the one that has the lowest average distance $\overline{d}$ to all other sequences S_i ($i \in [1, N]$) of the cluster: $\overline{d_C} = \min_{i \in C} \frac{1}{N-1} \sum_{j=1}^{N} d(S_i, S_j)$. Thus, each cluster is represented by an input genome, not an 'artificial' median genome. Per default, we use the cosine distance as metric d but also allow for other metrics.

In ViralClust, only clusters with a size of more than one are defined as 'real' clusters and the corresponding representatives are saved in a file. Clusters of size one are therefore not considered clusters, but named 'singletons' here, and are stored in a separate file. The combined set of cluster representatives and 'singleton' sequences is provided in a third file. This allows the user to choose which set of sequences is most suitable for the research question.

Optional parameters. (1) Inclusion of a genome of interest: In addition to the general input file, the user can specify a second fasta file with the --goi flag. This fasta file should contain one (or more) genome(s) of interest (GOI). If the user provides such a file, ViralClust will include the contained genomes in the final set of representative genomes. Further, in the result reports of the individual cluster tools, the GOIs are specifically marked. With this option, users can add their unpublished in-lab strain or mutant to downstream analyses and can further determine the closest phylogenetic viruses based on the resulting clusters. This also enables comparisons between the representative sequences and specific reference genomes, which are not necessarily cluster representatives. *(2) Metadata Information:* With the --ncbi flag ViralClust links additional information provided by the NCBI [30]. For this, we can sequence identifiers for GenBank accession ID. If found, we use the accession ID to retrieve additional information about the taxonomy (species and genus name), accession date, and the country the sequence originated from. ViralClust combines this metadata with the overview of clusters for each tool. *(3) Alignment and Phylogeny:* If the user sets the flag --eval of ViralClust, we further create a multiple sequence alignment (default: MAFFT (v7.4) [31]) of the representative genomes for each cluster tool. We subsequently use the alignments to build phylogenetic representations (default: FastTree (v2.1.10) [32]), which we finally visualize using Newick Utilities (v1.6) [33]. If --eval is enabled, we include the average phylogenetic distance calculated by FastTree between two representative genomes in the final summary report. Per default, FastTree uses the Jukes-Cantor distance model [34].

2 as communicated in: https://github.com/scikit-learn-contrib/hdbscan/issues/69#issuecomment-317742362

Table 1. Overview of datasets used in this study. `ViralClust` reduces the number of input genomes to a representative set of genomes. Data was downloaded from NCBI Virus. IAV – *Alphainfluenzavirus influenzae*; ⌀ SeqLen – average sequence length; #Seq – number of input genomes / segments; contains only records labeled as "`complete`"; PreProc – number of sequences after pre-processing; %nr – percentage of non-redundant sequences; Number of genomes after clustering – only considering clusters with a size greater than one; cd – CD-HIT-EST; sum – SUMACLUST; vse – VSEARCH; mmseq – MMSeqs2; hdb – HDBSCAN; Time – run time of `ViralClust` measured by `Nextflow`; Peak RAM – peak RSS (resident set size) of `ViralClust` measured by `Nextflow`. A detailed overview of time and memory consumption is shown in supplementary Tab. S1.

Taxonomic clade	⌀ SeqLen	#Seq	PreProc	%nr	Number of genomes after clustering					Time	Peak RAM
					cd	suma	vse	mmseq	hdb		
Orthoebolavirus	18.9 kb	632	443	70.09	8	7	7	11	11	12m	11.4 G
Hepacivirus hominis	9.6 kb	1,075	1,071	99.63	55	30	58	55	23	14m	10.7 G
Orthoflavivirus	9.2–11 kb	8,052	7,681	95.39	69	71	70	63	101	1h 22m	43.2 G
Flaviviridae	9–13 kb	9,895	9,478	95.79	220	180	221	211	194	2h 47m	52.6 G
IAV (1)	2,341 nt	83,543	56,973	68.20	66	390	90	280	1,412	3h 27m	273.9 G
IAV (2)	2,341 nt	82,569	56,212	68.07	56	338	73	216	1,311	3h 24m	270.9 G
IAV (3)	2,233 nt	83,400	55,862	66.98	63	305	75	265	1,371	3h 16m	268.8 G
IAV (4, H)	1,775 nt	156,586	108,514	69.30	300	1,237	464	1,574	2,307	8h 36m	510.0 G
IAV (5)	1,565 nt	84,653	49,465	58.43	46	262	76	179	1,218	2h 28m	238.3 G
IAV (6, N)	1,409 nt	114,639	73,786	64.36	228	773	329	640	1,622	4h 9m	572.2 G
IAV (7)	1,027 nt	96,928	46,371	47.84	25	185	38	269	981	1h 47m	225.1 G
IAV (8)	890 nt	85,944	42,308	49.23	51	218	69	264	802	1h 28m	207.2 G
Monkeypox virus	190–200 kb	1,077	999	92.76	14	1	3	23	2	16h 0m	6.5 G

Benchmark `ViralClust` by a variety of datasets

We applied `ViralClust` to six viral datasets of single-stranded RNA and double-stranded DNA viruses varying in number of sequences, sequence length, and sequence diversity. For an overview of the datasets used in this study, we refer to Tab. 1, in which we provide the total number of available sequences, the number of sequences after the implemented filtering steps, and the number of clusters resulting from each tool. Given these input sets, we report the number of clusters as calculated by the individual tools in their respective column, run time, and memory usage, see Tab. 1 and S1. All results generated by `ViralClust` are deposited in the zenodo repository: https://doi.org/10.5281/zenodo.18413373.

To provide an objective basis for comparing tool performance across datasets, we evaluated all five clustering algorithms using a scoring framework comprising five component scores: the Reduction Score (RS), Singleton Penalty Score (SPS), Clustering Quality Score (CQS), Taxonomic Concordance Score (TCS), and Cluster-to-Taxonomy Ratio (CTR), aggregated into an Overall Score (OS) with a maximum of 5. A detailed description of each metric is provided in the Methods section. The resulting scores are summarized in Tab. 2. All scores are shown in Tab. S3.

The scores reveal a considerable spread both across tools and across datasets, with OS values ranging from 2.311 (SUMACLUST, *Monkeypox virus*) to 4.996 (HDBSCAN, *Monkeypox virus*), reflecting the diversity of the evaluated datasets rather than a consistent ranking of tools. No single tool dominates across all datasets, and tool choice should therefore be guided by the biological question and dataset composition. Several general trends emerge from the scores and are discussed in detail in the respective subsections below. Compactness-based tools (CD-HIT-EST, SUMACLUST, VSEARCH) tend to score consistently well across RNA virus datasets, producing fewer, more compact clusters that achieve strong dataset reduction, but may collapse rare or biologically meaningful lineages in taxonomically diverse datasets. Noteworthy, SUMACLUST discards sequences containing characters other than ACGT, making it even more dependent on the quality of the input data and possibly difficult to use the results as a representative genomic dataset for subsequent analyses. MMSeqs2 produces higher cluster granularity, better preserving sequence-level diversity at the cost of larger representative sets, though its behavior becomes less predictable at very large dataset sizes. HDBSCAN is particularly well suited for datasets with continuous diversity distributions, but carries a fundamental limitation for datasets containing rare subtypes, which may not form sufficiently dense regions to be recognized as independent clusters and are instead treated as noise. These observations are intended as a starting point for tool selection; the scoring framework provided by `ViralClust` allows users to reproduce the same evaluation on their own datasets.

Table 2. Overall Score (OS) of the five clustering algorithms integrated into `ViralClust` across all evaluated datasets. The OS is the sum of five component scores – Reduction Score (RS), Singleton Penalty Score (SPS), Clustering Quality Score (CQS), Taxonomic Concordance Score (TCS), and Cluster-to-Taxonomy Ratio (CTR) — each in the range [0, 1], giving a maximum OS of 5. Higher values indicate better overall clustering performance with respect to dataset reduction, singleton rate, phylogenetic cluster separation, and taxonomic concordance. IAV – *Alphainfluenzavirus influenzae*; cd – CD-HIT-EST; sum – SUMACLUST; vse – VSEARCH; mmseq – MMSeqs2; hdb – HDBSCAN. All scores are shown in Tab. S3.

Taxonimic clade	Overall Score				
	cd	suma	vse	mmseq	hdb
Orthoebolavirus	4.622	4.682	4.687	3.940	3.720
Hepacivirus hominis	3.926	3.798	4.040	3.963	3.998
Orthoflavivirus	4.331	4.141	4.340	4.508	3.294
Flaviviridae	3.516	3.523	3.540	3.555	3.022
IAV (1)	4.422	4.852	4.930	4.424	4.384
IAV (2)	4.446	4.836	4.909	4.401	4.414
IAV (3)	4.396	4.829	4.911	4.502	4.365
IAV (4, H)	4.655	4.838	4.938	4.561	4.474
IAV (5)	4.385	4.871	4.925	4.415	4.239
IAV (6, N)	4.612	4.859	4.929	4.571	4.385
IAV (7)	4.545	4.687	4.785	4.693	4.131
IAV (8)	4.605	4.808	4.873	4.665	4.247
Monkeypox virus	2.690	2.311	4.161	2.763	4.996
Sum of Scores	55.151	57.035	59.968	54.961	53.669

Small and biased dataset - Orthoebolavirus

We tested the *Orthoebolavirus* dataset consisting of only 632 genomes to see how fine-grained `ViralClust` can work. *Orthoebolavirus* is a genus within the *Filoviridae* family (ICTV phylogeny see Fig. S1). The genomes are negative single-stranded RNA and about 18.9 kb in length [35]. In comparison to other viral clades, this dataset includes a small number (six) of well-described species according to the ICTV, see Tab. 3, with a notable presence with 461/632 sequences of *Orthoebolavirus zairense* (Ebola virus, EBOV), a human pathogen over-represented due to several outbreaks. We aim to investigate the selection of representative genomes with `ViralClust` for such a small, well-defined, and un-

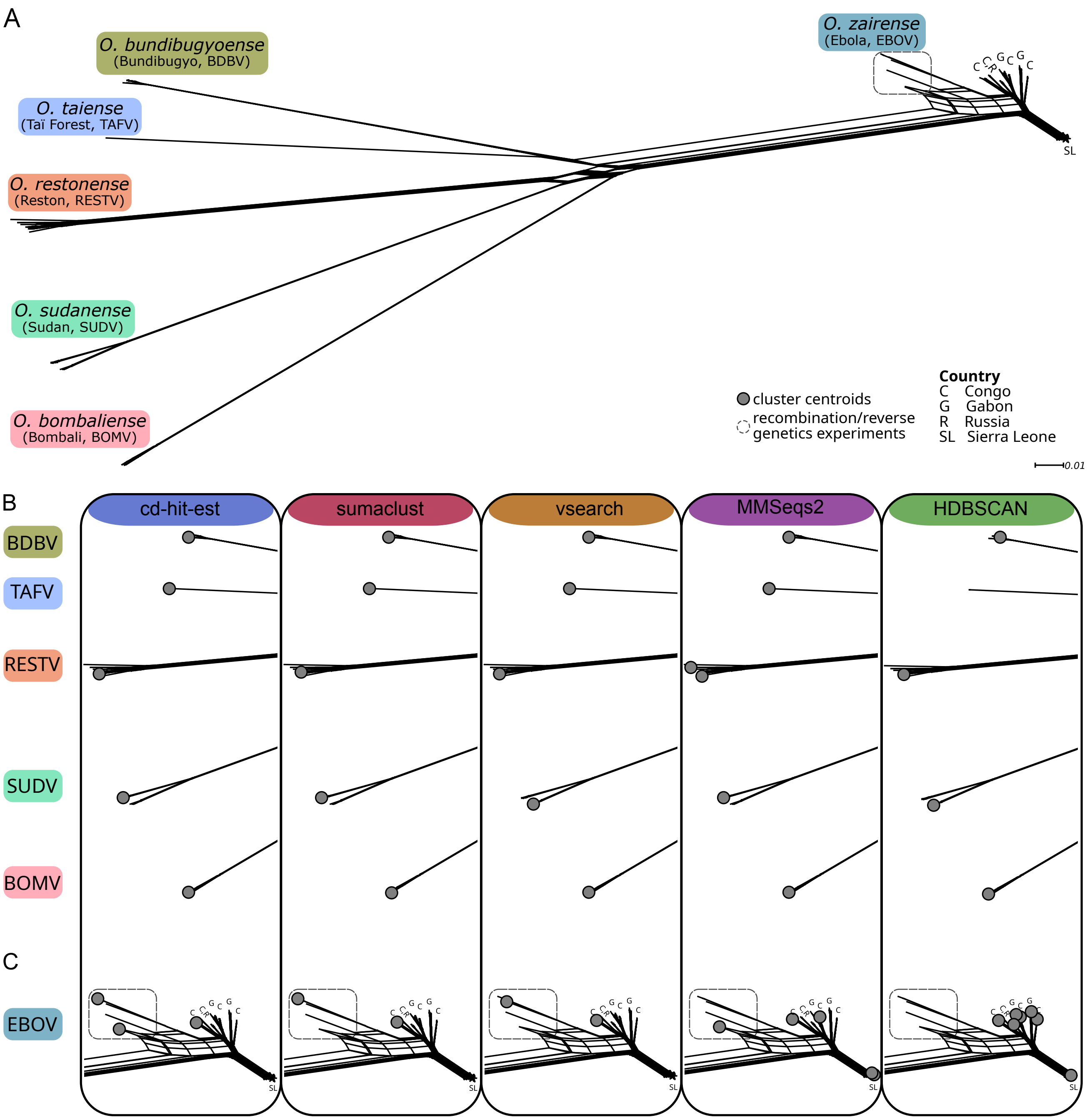

Figure 2. (A) Split graph of the *Orthoebolavirus* dataset with labeled species. (B) Cluster representatives of CD-HIT-EST, SUMACLUST, VSEARCH, MMSeqs2, and HDBSCAN in the subtrees of BDBV, TAFV, RESTV, SUDV, and BOMV (top to bottom). (C) Cluster representatives in the EBOV subtree. Gray dots display the cluster representative sequences selected by the individual tools. The Zaire *Orthoebolavirus* subtree shows a distinction by geographic origin, indicated as follows: C – Congo; G – Gabon; R – Russia; SL – Sierra Leone. The split graph is reconstructed using SplitsTree based on the MAFFT alignment of the complete genome sequences (443 genomes) resulting from the first filtering steps of ViralClust.

Table 3. *Orthoebolavirus* species including virus name and abbreviation (Abbr.) according to ICTV and number of downloaded sequences per species (#Seqs). 93 sequences were unclassified regarding their species.

Species	Virus name	Abbr.	#Seqs
O. bombaliense	Bombali	BOMV	8
O. bundibugyoense	Bundibugyo	BDBV	24
O. restonense	Reston	RESTV	22
O. sudanense	Sudan	SUDV	21
O. taiense	Taï Forest	TAFV	3
O. zairense	Ebola	EBOV	461

balanced dataset. The pre-processing of ViralClust reduced the dataset size by 30 % to 443 genomes. Subsequent clustering resulted in 7 to 11 representative genomes, see Tab. 1. In general, all five clustering tools could distinguish the species in individual clusters, see Fig. 2. A conventional representation of the phylogeny of the representative genomes and the corresponding ICTV classification can be found in Fig. S2. Additionally, in Fig. S3 a RAxML-NG phylogenetic tree based on a full-genome MAFFT alignment can be found as an overview for the 443 individual sequences. CD-HIT-EST, SUMACLUST, VSEARCH and MMSeqs2 revealed correctly one cluster representative for the five species BOMV, BDBV, RESTV, SUDV, and TAFV each. A notable exception is HDBSCAN, which could not identify the species TAFV, Fig. 2B and Fig. S2F. All tools clus-

tered TAFV genomes (light blue) correctly together with BDBV (dark green). Each of the tools selected at least one natural EBOV strains (CD-HIT-EST:1, SUMACLUST:1, VSEARCH:1, MMSeqs2:4, HDBSCAN:7). Additionally, CD-HIT-EST, SUMACLUST, VSEARCH and MMSeqs2 selected up to two representatives of EBOV derived from recombination or reverse genetics experiments (see Fig. 2 dashed gray box). HDBSCAN selected only natural EBOV sequences (isolated from viral infections). Interestingly, these comparatively high number of representative genomes represent geographic and temporal separation of EBOV outbreaks.

Species Hepacivirus hominis and genus Orthoflavivirus from the family Flaviviridae

Flaviviridae is a large family of positive single-stranded RNA viruses consisting of four genera: *Orthoflavivirus*, *Pestivirus*, *Pegivirus* and *Hepacivirus* [36], with a total of 97 species (ICTV, see Fig. S4). *Flaviviridae* includes human pathogenic viruses such as Zika virus, tick-borne encephalitis virus (TBEV), dengue virus, and hepatitis C virus (HCV). Genomes are approximately 9–13 kb in length [36]. We chose three datasets to compare results on species, genus, and family level. On the species level, we chose *Hepacivirus hominis* (HCV infecting humans) as a well-described and phylogenetically closely related species. On a genus level, we chose *Orthoflavivirus* due to the well-described dataset being very diverse (ICTV: 53 species). We chose *Flaviviridae* on the family level. The datasets *Hepacivirus hominis* and *Orthoflavivirus* are complete subsets of the dataset *Flaviviridae*. Finally, our focus was on how the selected representatives from species and genus study are consistent within the selection from the entire virus family. Preprocessing within the ViralClust pipeline reduced the datasets by 1–5 %, see Tab. 1 and 4.

Species Hepacivirus hominis. *Hepacivirus hominis* (HCV) consists of eight genotypes [37, 38, 39], however, only seven are listed in the ICTV, see Fig. S4 yellow rectangle. With the neighbour-net method we gained 13 clearly distinguishable clusters, see Fig. 3. This difference can also be explained by the observations of virologists in the past: (i) Two additional clusters, besides the seven downloaded genotypes, are formed by recombinants: the natural recombinants (e.g. 2k/1b or 2b/1b, Fig. 3 – close related to genotype 1b) [40, 41, 42] and the artificial recombinants (Fig. 3 – between genotype 1 and 2) [43, 44, 45]. Note: further recombinants are mixed into the genotype 2. (ii) Three additional clusters are formed within genotype 1 – in total, divided into genotype 1, 1a, 1b, and rare genotypes 1 (1c–1n). Currently, 14 subtypes are listed in the ICTV [36, 46, 47]; (iii) One additional cluster exists due to the diverse genotype 6: Currently, 33 subtypes are known for genotype 6 according to the ICTV [36, 48], with subtype 6a being the first one discovered [49]. All sequences part of subtype 6a cluster distinguishable from other subtypes, with the notable exception of subtype 6b, which more close to 6a than other subtypes. The tools of ViralClust calculated between 23 (HDBSCAN) and 58 (VSEARCH) representative genomes (see Fig. 3 colored circles, and Tab. 1). Although any of the methods represents 3–8 times more clusters than the currently recognized number of HCV genotypes, using more representatives may be necessary to capture the considerable diversity within genotypes. Among the evaluated methods, HDBSCAN produced the largest clusters (minimum size 5, average size 40.87, median size 14; see Tab. 4), resulting in the lowest total number of clusters. All clustering tools in ViralClust selected representative genomes distributed across the phylogenetic tree. However, substantial differences in the number of representatives were observed for the different genotypes, see Fig. 3. Interestingly, none of the tools was able to identify genotype 7 as an individual cluster.

Across all five clustering algorithms, the largest cluster was consistently identified as genotype 1a. Each tool selected 1–3 representatives for this cluster. While four tools (CD-HIT-EST, VSEARCH, MMSeqs2, HDBSCAN) selected the same representative genome for genotype 1b, SUMACLUST identified seven. Notably, HDBSCAN did not select any rare subtypes of genotype 1, whereas the other tools included one (SUMACLUST) or six (CD-HIT-EST, VSEARCH, MMSeqs2) representatives. This is likely due to the k-mer–based similarity measure used by HDBSCAN, which appears to group the rare genotype 1 subtypes more tightly, leading to no additional representatives. A small subtree annotated as genotype 1 (Fig. 3, bottom right) was only found to be an independent cluster by HDBSCAN, which selected a single genome from these variants. The large number of subtypes within genotype 6 can be explained by the diversity of this genotype, see Fig. 3, even tough the tools showed picked different representatives. The tools CD-HIT-EST, VSEARCH, and MMSeqs2 selected 18 representative genomes for genotype 6, whereas HDBSCAN identified substantially fewer representatives (3). These differences may be attributed to the distinct algorithmic strategies employed: HDBSCAN is based on k-mer similarity, while the other tools combine heuristic approaches with (pairwise) sequence alignments, which may increase sensitivity to finer sequence variation. Interestingly,

Table 4. Cluster statistics for *Hepacivirus hominis* (HCV), *Orthoflavivirus* (*Flavivirus*), and *Flaviviridae* datasets. For each tool, the basic cluster statistics are listed. cd – CD-HIT-EST; sum – SUMACLUST; vse – VSEARCH; mmseq – MMSeqs2; hdb – HDBSCAN; # clstr – number of clusters (with a size greater than one); min/max – size of smallest/largest cluster; avg/med – average/median cluster size; d. cen – average phylogenetic distance between two representative genomes; singl – number of singletons (= cluster with only one element); cps – average cluster per species; cpg – average cluster per genus; PD ratio – ratio between Faith's phylogenetic distance (PD) of the full tree and the representative tree. The values cps and cpg give a measurement of how much a single species/genus is divided into different groups.

	algorithm	# clstr	min	max	avg	med	singl	d. cen	cps	cpg	PD ratio
HCV	cd	55	2	410	17.71	3.0	97	0.804	1.75	23.00	0.170
	suma	30	2	373	24.67	3.0	42	0.637	2.08	15.00	0.072
	vse	58	2	412	16.97	3.0	87	0.806	1.74	24.00	0.173
	mmseq	55	2	410	17.75	3.0	95	0.814	1.82	17.00	0.172
	hdb	23	5	412	40.87	14.0	131	0.594	1.61	21.00	0.058
Flavivirus	cd	69	2	1,642	110.14	5.0	81	1.510	1.44	46.00	0.275
	suma	71	2	1,622	96.44	5.0	86	1.663	1.57	49.00	0.322
	vse	70	2	1,431	108.63	6.0	77	1.493	1.42	46.00	0.308
	mmseq	63	2	1,798	120.68	6.0	78	1.704	1.32	30.00	0.289
	hdb	101	5	1,371	69.19	19.0	693	1.189	2.49	100.00	0.231
Flaviviridae	cd	220	2	1,642	41.54	3.0	339	2.466	1.64	20.60	0.302
	suma	180	2	1,622	44.81	3.0	267	2.739	1.73	21.00	0.268
	vse	221	2	1,431	41.46	3.0	316	2.280	1.62	27.75	0.316
	mmseq	211	2	1,798	43.33	3.0	336	2.520	1.54	18.00	0.311
	hdb	194	5	1,371	41.98	14.0	1,333	1.704	2.09	39.40	0.148

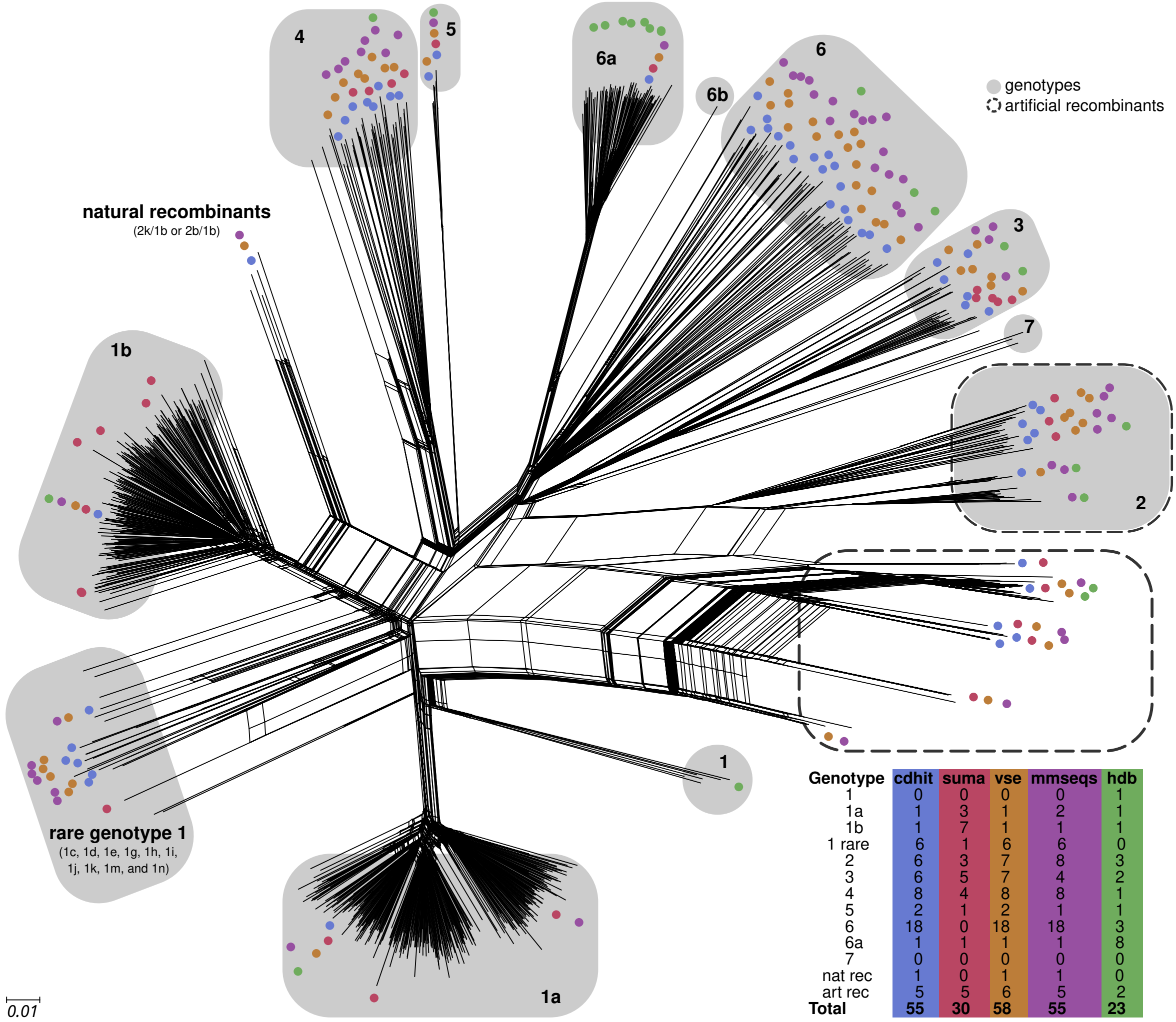

Genotype	cdhit	suma	vse	mmseqs	hdb
1	0	0	0	0	1
1a	1	3	1	2	1
1b	1	7	1	1	1
1 rare	6	1	6	6	0
2	6	3	7	8	3
3	6	5	7	4	2
4	8	4	8	8	1
5	2	1	2	1	1
6	18	0	18	18	3
6a	1	1	1	1	8
7	0	0	0	0	0
nat rec	1	0	1	1	0
art rec	5	5	6	5	2
**Total**	**55**	**30**	**58**	**55**	**23**

Figure 3. Splits graph of HCV with labeled genotypes (gray), artificial (dashed box) and natural recombinants, and cluster representatives of the five tools incorporated in ViralClust (CD-HIT-EST, SUMACLUST, VSEARCH, MMSeqs2, and HDBSCAN). Numbers in gray boxes represent genotypes. The tree can be divided into 13 subtrees, whereas genotype 1 consists of four subtrees (1, 1a, 1b, and the rare genotypes 1c–1n); and all subtype 6a genomes are clearly distinguishable from all other subtypes summarized here as genotype 6. All known natural recombinants (e.g. 2k/1b or 2b/1b) form a separate subtree most closely related to genotypes 1b and 4. Some artificial recombinants mix with genotype 2 genomes, whereas others form a subtree between genotypes 1 and 2. The number of representatives per genotype is shown in the table at the bottom right. The split graph is reconstructed using SplitsTree based on the MAFFT alignment of the complete genome sequences (1,071 genomes) resulting from the first filtering steps of ViralClust.

SUMACLUST did not identify genotype 6 as a distinct cluster. This may be explained by the filtering step in SUMACLUST, which removes sequences containing non-ACGU characters and may have excluded genotype 6 genomes prior to clustering. In contrast, HDBSCAN selected eight representatives for subtype 6a, whereas the remaining tools selected only one. This difference may be influenced by the high abundance and relative homogeneity of subtype 6a genomes, in combination with the density-based clustering approach used by HDBSCAN. Several genomes in the dataset were labeled as recombinants leading to a split in the bottom right part of the tree (Fig. 3, dashed box) and potentially inflating the number of clusters. These recombinant genomes are annotated as a mix between genotypes 1 and 2. Overall, all tools except HDBSCAN produced broadly comparable results. While HDBSCAN tended to select more representatives in densely connected regions (e.g. genotype 6a), it selected fewer genomes in other parts of the tree. When examining individual sequences, they are usually assigned to the same cluster, although the chosen representative may differ slightly, see Fig. 4.

Genus Orthoflavivirus. The clustering of 8,052 *Orthoflavivirus* resulted in 63–101 clusters with the different tools, whereas MMSeqs2 serves with the smallest number of clusters and HDBSCAN with the highest, see Tab. 4. As for HCV, the number of clusters are higher than the number of species (53). The selection of more than one genome per species as representative can be sufficient to cover greater diversity across the whole genus of *Orthoflavivirus*. Despite minor differences, all five clustering algorithms produced broadly similar results, with representatives distributed across the phylogenetic representation of the dataset (see Fig. S5).

When comparing the selected representatives with the ICTV species assignments, which contains exactly one reference genome per species, see Fig. S4 violet background, we find (a) clusters which contain exactly one of the defining ICTV species, as expected; (b) ICTV species representatives not part of any cluster (= singletons); (c) clusters, which have no defining ICTV species, which might point to a generally more fine-grained clustering; and (d) some clusters have been merged by specific tools, so that two (or more) ICTV species could be found in one cluster, which should not have happened in order to represent the current phylogeny well.

The different tools found between 16 and 23 clusters containing exactly one ICTV species representative, see Tab. 5, whereas 18–23 ICTV reference genomes were not assigned to any cluster with more than one sequence, and thus remain as singletons within the ViralClust pipeline. This likely reflects recently added species for which only limited sequence data are currently available. An important detail is that SUMACLUST excludes any sequences containing char-

Accession ID	cd-hit-est	sumaclust	vclust	MMseqs2	HDBSCAN
AB520610	Cluster 17	Cluster 1	Cluster 18	Cluster 70	Cluster 0
AF271632	Cluster 17	Cluster 2	Cluster 18	Cluster 70	Cluster 0
D10749	Cluster 17	Cluster 1	Cluster 18	Cluster 70	Cluster 0
EF621489	Cluster 17	Cluster 2	Cluster 18	Cluster 70	Cluster 0
EU155213	Cluster 17	Cluster 1	Cluster 18	Cluster 70	Cluster 0
EU155214	Cluster 17	Cluster 1	Cluster 18	Cluster 70	Cluster 0
EU155215	Cluster 17	Cluster 2	Cluster 18	Cluster 70	Cluster 0
EU155216	Cluster 17	Cluster 1	Cluster 18	Cluster 70	Cluster 0
EU155233	Cluster 17	Cluster 1	Cluster 18	Cluster 70	Cluster 0
D49374	Cluster 101	Cluster 31	Cluster 97	Cluster 55	Cluster 10
JX227959	Cluster 35	Cluster 32	Cluster 34	Cluster 27	Unclustered
JX227960	Cluster 35	Cluster 32	Cluster 34	Cluster 27	Unclustered
AF238485	Cluster 2	Cluster 33	Cluster 9	Cluster 84	Cluster 6
MN385583	Cluster 145	Cluster 34	Cluster 140	Cluster 54	Cluster 10
KY348757	Cluster 147	Cluster 35	Cluster 142	Cluster 145	Unclustered
AB030907	Cluster 4	Cluster 36	Cluster 4	Cluster 76	Unclustered
AF238486	Cluster 4	Cluster 36	Cluster 4	Cluster 76	Unclustered
JX227964	Cluster 149	Cluster 37	Cluster 143	Cluster 115	Cluster 8
JX227978	Cluster 129	Cluster 38	Cluster 125	Cluster 143	Unclustered

Figure 4. Optional output of `ViralClust` in web-based format (HTML) for the dataset HCV. The interactive visualization allows the user to (i) sort the table by tool results or accession IDs, (ii) view and compare color-coded clusters, and (iii) access the NCBI entry for each sequence (link, if available). For most sequences, the clustering results of the different tools are consistent (top). Differences between the tools become apparent (bottom), where some sequences form single-sequence clusters (e.g. `D49374`), others consistently form small clusters across tools (e.g. `AB030907` and `AF238486`), and individual sequences are not assigned to any cluster by specific tools, such as the `HDBSCAN`'unclustered' sequences. The complete table is provided in the electronic supplement.

Table 5. Comparing the clustering results of the *Orthoflavivirus* dataset with the species chosen for the phylogeny of ICTV, see Fig. S4. ✓ – number of clusters with exactly 1 ICTV representative (in agreement with known phylogeny); Singleton – Number of clusters on containing one ICTV representative; ∅ – number of clusters without ICTV representative (showing additional information for downstream analysis); Discrepancies – (a) number of cluster with >= 2 ICTV representatives (e.g., '1x4' for four ICTV representatives in one cluster); (b) number of ICTV representatives that are 'singletons' (these sequences are alone a cluster); (c) other issues (e.g., `SUMACLUST` removes sequences including nucleotides $n \notin \{A, C, G, T\}$). For a more detailed overview see Tab. S4 and S5.

	✓	Singleton	∅	Discrepancies
cd	23	18	45	1×2
suma	20	20	50	1×2<br>St. Louis encephalitis (`DQ525916`) removed
vse	23	18	46	1×2
mmseq	23	18	39	1×2
hdb	16	23	84	1×4

acters other than `A`, `C`, `G`, or `T`. As a result, the ICTV representative genome for St. Louis encephalitis was removed from the dataset during preprocessing. Between 45 and 84 clusters contained no ICTV reference genome. These clusters likely represent intraspecific diversity or unclassified lineages, providing additional resolution beyond the current ICTV taxonomy. `CD-HIT-EST`, `SUMACLUST`, `VSEARCH`, and `MMSeqs2` agreed to cluster two reference genomes of ICTV together into one cluster: Saboya (`NC_033697`) together with Potiskum (`NC_029054`). `HDBSCAN` also merged Saboya (`NC_033697`) together with other species, but in this case together with Modoc (`NC_003635`), Uganda S (`NC_033698`), and Wesselsbron (`EU707555`). For a more details see Tab. S4 and S5. However, if higher resolution is required, we recommend including singletons as well. In this case, users should be aware that incorporating singletons may lead to over-representation of certain regions within the dataset. `HDBSCAN` often adapts to the density of subtrees: for very dense clusters, it selects several representatives (see Fig. S5 top right), whereas

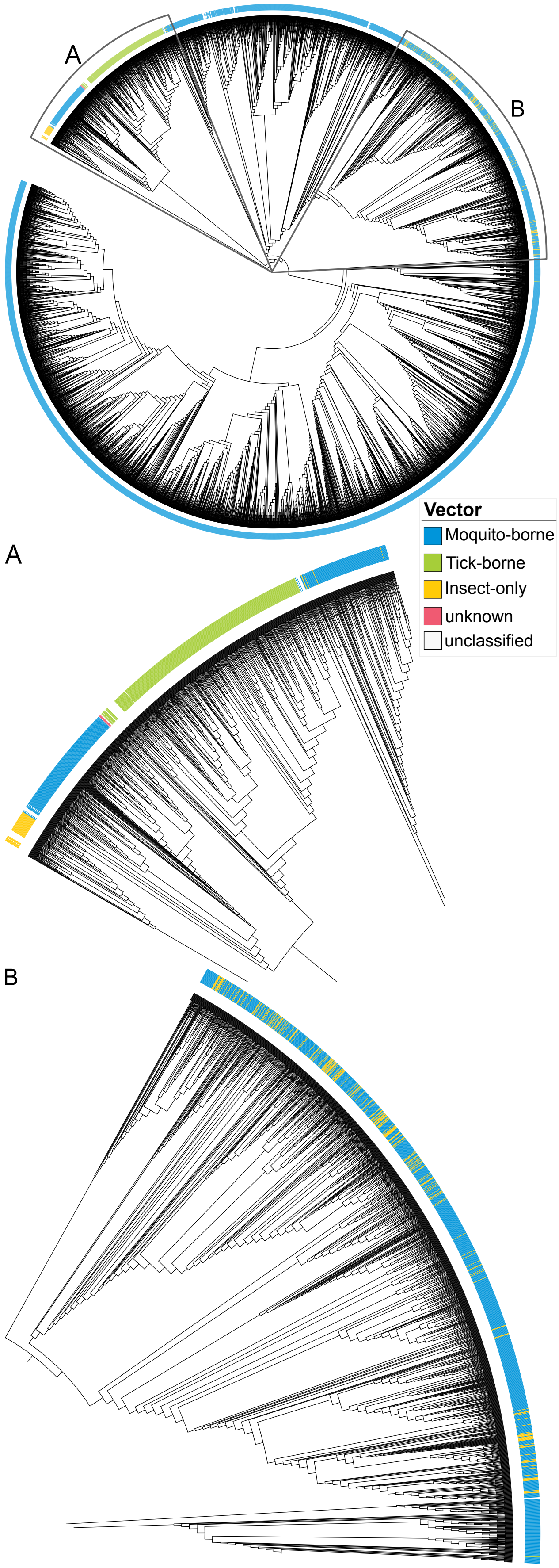

Figure 5. Phylogenetic representation based on the *Orthoflavivirus* dataset with color-coded vectors. Within the subtrees A and B, parts of the tree do not follow a vector-based classification. The complete and enlargeable tree is shown in Fig. S6. The tree is reconstructed using `FastTree` based on the `MAFFT` alignment of the complete genome sequences (7,681 genomes) resulting from the first filtering steps of `ViralClust`.

clusters consisting of a single sequence are typically not chosen as representatives and instead remain unassigned. If such outliers are of interest for downstream analysis, users should manually include these singletons in subsequent steps (as these sequences are saved in a separate file). As a result, HDBSCAN yields the lowest average cluster size (69.19) and the largest number of singletons (693). An important observation concerns the previously proposed subclassification within *Orthoflavivirus* according to their vector type [36]. These analyses are based solely on parts of the the RdRp, see Fig. S4. Our analysis is based on complete genomes, indicating at least for subtrees, that the phylogeny can not be drawn based on the vector-type, see Fig. 5.

Family Flaviviridae. Clustering of all 9,895 *Flaviviridae* species resulted in 180–221 clusters with SUMACLUST generating the lowest number of clusters and VSEARCH the highest, see Tab. 4. These results are twice as much as the reported 97 species from ICTV, Fig. S4, as for the subsets HCV species and *Orthoflavivirus* genus.

The average cluster size remains about the same for all five algorithms. MMSeqs2 produced the overall largest cluster (1,798 sequences), whereas HDBSCAN (1,371) and SUMACLUST (1,431) produced the smallest, with 200–400 fewer sequences. HDBSCAN also produced the largest number of singletons, with a notably high fraction (1,333), see Tab. 4. The ICTV phylogeny (Fig. S4) was constructed using the RdRp region of 125 sequences [36]. To evaluate how well this single-gene phylogeny reflects whole-genome evolutionary relationships, we compared it against a phylogeny inferred from 9,895 complete *Flaviviridae* genomes. While the overall topology of the full genome based phylogeny aligns broadly with the RdRp based ICTV classification, Fig. S7, several notable discrepancies emerged. Specifically, as visible in Fig. 6 (from left to right) (i) 25 genomes annotated as orthoflaviviruses clustered together in a distinct outlier subtree; (ii) 94 HCV genomes appeared more closely related to most of the pegiviruses than to other hepaciviruses; (iii) a single *Pestivirus* genome (Trinbago virus) was located within this divergent HCV cluster (not visible); (iv) another three, more divergent, *Pegivirus* sequences clustered with the majority of HCV; and (v) nine *Orthoflavivirus* genomes were placed nearer to basal pestiviruses (Fig. 6A – blue part between yellow and green). These discrepancies may be partly explained by misannotations in public sequence databases, but they also highlight the inherent limitations of single-gene phylogenies for robust virus taxonomy.

Comparing the datasets. We chose *Flaviviridae* on the family level (9,895 genomes) to test the robustness of the different clustering approaches, especially in comparison to clustering on the species level (HCV, 1,075 genomes true subset of *Flaviviridae*) and on the genus level (*Orthoflavivirus*, 8,052 genomes true subset of *Flaviviridae*), see Tab. 1. The fine-grained clustering observed at the species level is for some clustering algorithms also preserved at the family level, see Tab. 6. For example, within genomes of HCV, the methods CD-HIT-EST, SUMACLUST, and VSEARCH consistently formed the same clusters and selected the same representatives when compared between the HCV only and HCV clustered together with all other *Flaviviridae* datasets. MMSeqs2 shows slight differences in the selection of representatives, see Fig. S8. Clustering the entire *Flaviviridae* family with MMSeqs2 produced 56 clusters for HCV, compared to 55 clusters when only the HCV species was analyzed. Of these, 54 clusters were identical, while one cluster in the HCV clustering was split into two in the *Flaviviridae* analysis. Among the representatives, 49 were identical, and the remaining non-identical ones were phylogenetically close. In contrast, HDBSCAN showed larger differences in both cluster composition (only one identical cluster) and choice of representatives (six identical). When restricted to HCV, HDBSCAN produced twice as many clusters (23) compared to when HCV was analyzed as part of the *Flaviviridae* dataset (12). Genotypes 1a and 1b are represented by multiple genomes in the clustering of HCV, while these are grouped together in the results

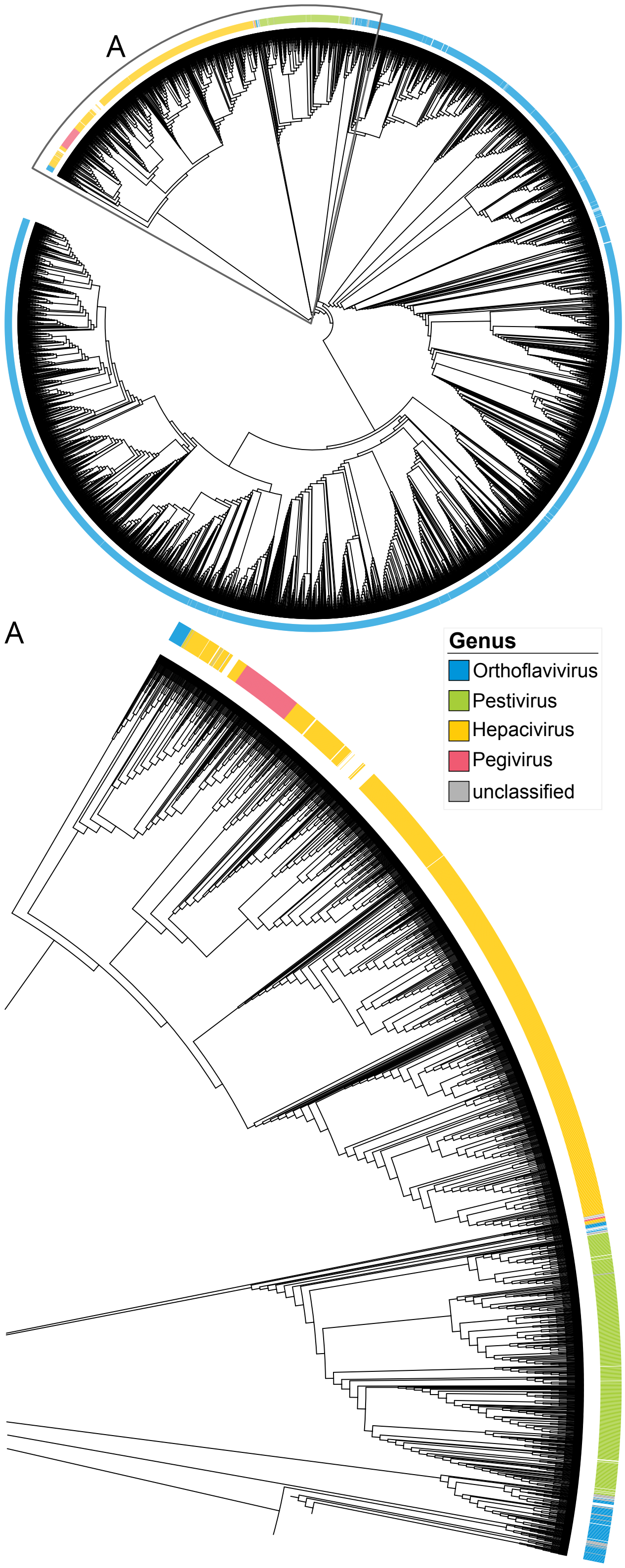

Figure 6. Phylogenetic representation of the *Flaviviridae* dataset with highlighted genera. A) parts of the tree that do not follow the classification by genus. Complete tree shown in Fig. S7. The tree is reconstructed using FastTree based on the MAFFT alignment of the complete genome sequences (9 478 genomes) resulting from the first filtering steps of ViralClust.

of *Flaviviridae*. This suggests that HDBSCAN is more sensitive to the chosen level and number of input sequences and adapts its clustering accordingly. When switching to the comparison on a genus level at the example of *Orthoflavivirus* only vs. *Orthoflavivirus* within *Flaviviridae*, all five algorithms generated the same largest cluster, containing dengue virus 1, see Tab. 4. SUMACLUST and VSEARCH again produced identical clusters and representatives, indicating robustness to the number of input sequences, see Tab. 6. For the genus *Orthoflavivirus*, CD-HIT-EST and MMSeqs2 selected the same total number of representatives – 69 and 63 respectively, as in the corresponding *Orthoflavivirus* subsets of the *Flaviviridae* analysis. While CD-HIT-EST produced identical cluster compositions, 8 representatives differ. Similarly, MMSeqs2 yielded 56 clusters with unchanged composition, but 48 identical representatives. In both cases, the differing representatives were phylogenetically close, and thus these discrepancies are considered minor (Fig. S9). In contrast, HDBSCAN generated substantially more clusters for *Orthoflavivirus* when clustering within the full *Flaviviridae* dataset (156 representatives) compared to clustering *Orthoflavivirus* alone (101 representatives). Of these, 27 clusters and 80 representatives were identical. The largest differences were observed in the mosquito-borne clade, where HDBSCAN selected considerably more representatives than the other algorithms (Fig. S9). The run time does not scale linearly with dataset size, see Tab. S1. Although the *Orthoflavivirus* dataset accounts for 81 % of the *Flaviviridae* sequences (7,681 of 9,478), ViralClust required only 48.5 % of the total run time (1 h 22 m of 2 h 47 m; Tab. 1). For the HCV dataset, the effect was less pronounced: comprising 11.3 % of the sequences in the *Flaviviridae* dataset, it required 8.3 % of the run time (14 m of 2 h 47 m; Tab. 1). The overall memory consumption scales almost linearly when comparing the *Flaviviridae* dataset with the *Orthoflavivirus* dataset (183.9 G, 164.3 G; Tab. S1). In contrast, this is not the case for the HCV dataset, where memory usage is four times higher than for *Flaviviridae* when comparing with the size of the datasets.

Table 6. Comparison of the clustering results of *Hepacivirus hominis* only vs. *Hepacivirus hominis* within *Flaviviridae* and *Orthoflavivirus* only vs. *Orthoflavivirus* within the *Flaviviridae* analysis. clstr-comp. – cluster composition, i.e. number of identical clusters (number of cluster in *Hepacivirus hominis* or *Orthoflavivirus*/number of cluster in *Flaviviridae*); repr – identical representatives; ✓ – results are identical (id.). cd – CD-HIT-EST; sum – SUMACLUST; vse – VSEARCH; mmseq – MMSeqs2; hdb – HDBSCAN.

Hepacivirus hominis: only vs. within *Flaviviridae*		
	clstr-comp.	repr
cd	✓ (56 clstr)	✓
suma	✓ (31 clstr)	✓
vse	✓ (59 clstr)	✓
mmseq	54 id. (55/56)	49 id.
hdb	1 id. (23/12)	6 id.
Orthoflavivirus*: only vs. within *Flaviviridae		
	clstr-comp.	repr
cd	✓ (69 clstr)	57 id.
suma	✓ (71 clstr)	✓
vse	✓ (70 clstr)	✓
mmseq	56 id. (63/74)	48 id.
hdb	27 id. (101/158)	80 id.

Alphainfluenzavirus influenzae as an example for segmented viruses including partial and reverse-complement sequences

Alphainfluenzavirus influenzae (formerly *Influenza A virus*, IAV) provides a representative case for studying segmented viral genomes and handling truncated reverse-complement sequences. Here, we address several key questions relevant for sequence clustering and analysis of such viruses: We first confirm that genotype represents surprisingly well to serotype classification. For the eight genome segments, sequences can be analyzed individually or concatenated; here we show that independent analysis captures meaningful differences. Partial genomes are reliably placed in phylogenetic trees, demonstrating robustness. Finally, reverse-complement sequences are detected through characteristic patterns (in this section highlighted with gray background); the current version of ViralClust automatically restores the positive orientation to simplify analysis.

IAV is a segmented (8 segments), negative-sense, single-stranded RNA virus belonging to the family *Orthomyxoviridae* (see Fig. S10). Classification is traditionally based on the two surface proteins hemagglutinin (H, segment 4) and neuraminidase (N, segment 6), with 18 H and 11 N subtypes reported by the CDC (see Fig. S11 and S12). Our analyses confirm that segment data reflect subtype classification well, demonstrating that genotype approximates subtype and that segment-wise analysis captures meaningful biological differences.

Each of the eight genome segments in our dataset contains on average 98,532 sequences, with the largest datasets corresponding to segments 4 (156,586) and 6 (114,639) and the smallest to segment 2 (82,569). Segment lengths range from 890 nt (segment 8) to 2,341 nt (segments 1 and 2), and non-redundant sequence fractions vary from 64—69 % for segments 1–4 and 6 to 47–49 % for segments 7 and 8.

Clustering results (90 % sequence identity, k-mer length 7) vary widely depending on segment and method, ranging from 25 clusters (CD-HIT-EST, segment 7) to 2,307 (HDBSCAN, segment 4), see Tab. 1, with the largest datasets (segments 4 and 6) also yielding the highest number of clusters across all five tools. This wide range illustrates that tool choice has a substantial impact on the granularity of the resulting representative set, independent of the underlying biological diversity. CD-HIT-EST produced the lowest number of clusters for all segments, followed by VSEARCH. MMSeqs2 and SUMACLUST are close to each other in terms of the number of clusters. MMSeqs2 yielded a lower number of clusters for segments 1, 2, 3, 5 and 6, but more for 4, 7 and 8. HDBSCAN resulted in the highest number of clusters by far. The consistently lower cluster counts of CD-HIT-EST and VSEARCH reflect their more aggressive merging strategies, which may be advantageous when the goal is maximal dataset reduction.

The coverage of known subtypes provides a more biologically grounded basis for comparing tool performance than cluster counts alone. For segments 4 and 6, which encode the surface proteins, CD-HIT-EST, SUMACLUST, VSEARCH, and MMSeqs2 select representatives covering all known subtypes, see Tab. 7 and Fig. 7 and 9. In contrast, HDBSCAN fails to select representatives for H17 and H18 (segment 4) and N10 and N11 (segment 6). These are among the rarest subtypes in the dataset, and their absence from the HDBSCAN representative set is likely a fundamental consequence of density-based clustering: subtypes represented by very few sequences may not form sufficiently dense regions to be recognized as independent clusters, and are instead treated as noise.

Partial genomes are reliably placed at the correct positions within phylogenetic trees, demonstrating the robustness of our approach even when sequences are incomplete. Reverse-complement sequences, however, present a more disruptive challenge. Comprising 19.22 % of the segment 4 dataset and 11.37 % of the segment 6 dataset, they arrange in a characteristic 'mirror-like' configuration in the phylogenetic tree. Such a pattern, if undetected, would cause clustering algorithms to treat forward and reverse-complement copies of the same sequence as unrelated, artificially inflating cluster counts and distorting representative selection. Therefore, we implemented ViralClust to detect reverse-complement sequences and to automatically restore their original orientation, simplifying downstream analysis, ensuring that strand orientation artifacts do not propagate into downstream analyses. To quantify the impact of reverse-complement sequences on clustering results, we compared three scenarios for segments 4 (H) and 6 (N): clustering the

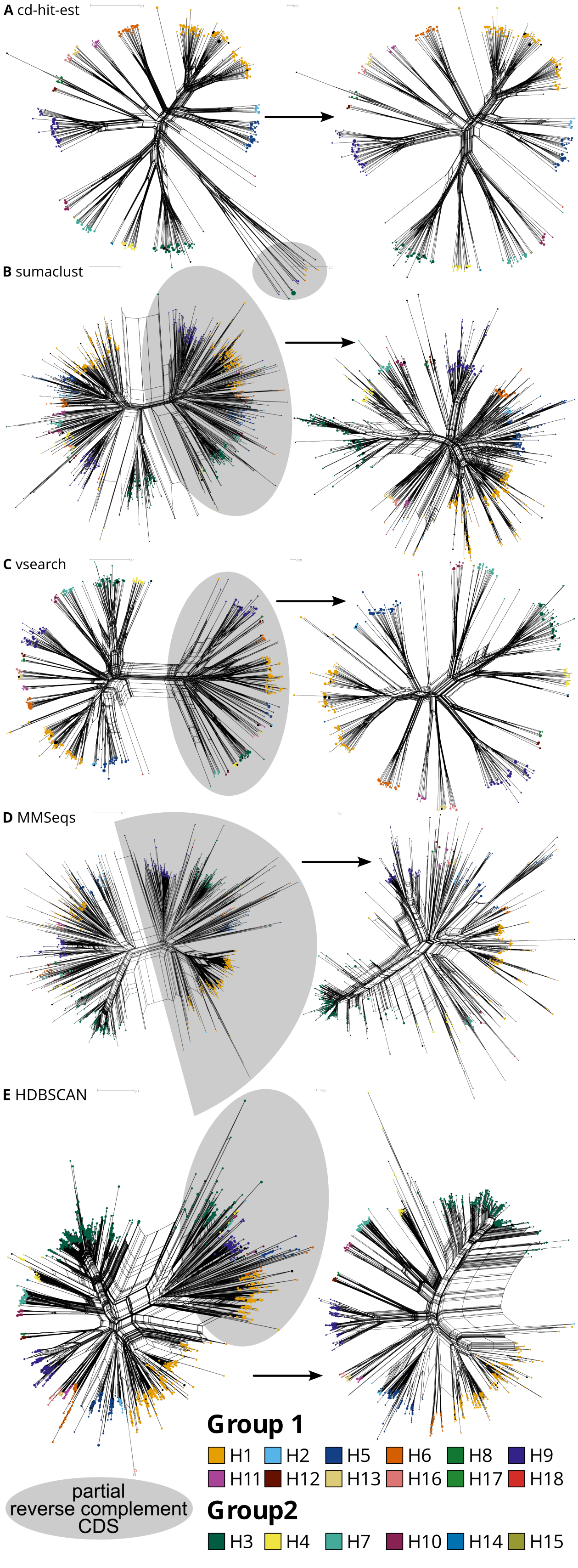

Figure 7. Split graphs of IAV segment 4 (H) representatives of (A) CD-HIT-EST, (B) SUMACLUST, (C) VSEARCH, (D) MMSeqs2, and (E) HDBSCAN. Left – all representatives (including partial and reverse-complement); Right – only complete genomes. The node size indicates cluster size. The split graphs are reconstructed using `SplitsTree` based on the MAFFT alignments. Gray background highlights reverse-complement sequences (19.22 % of the non-redundant dataset). HDBSCAN did not select representatives for subtypes H17 and H18. They were manually and marked them as framed circles. For details see Fig. S13–S17.

Table 7. Number of representatives per subtype for segment 4 (H) and 6 (N). #repr - number of representatives (= number of clusters with a size greater than one); cd – CD-HIT-EST; sum – SUMACLUST; vse – VSEARCH; mmseq – MMSeqs2; hdb – HDBSCAN.

Subtype	#repr				
	cd	suma	vse	mmseq	hdb
**Segment 4 (H)**					
H1	83	352	128	527	613
H2	5	26	10	19	35
H3	34	215	59	460	654
H4	14	44	22	28	58
H5	27	145	43	107	210
H6	19	54	27	33	67
H7	16	37	22	23	100
H8	5	9	7	5	11
H9	44	193	71	242	347
H10	9	27	11	17	42
H11	7	16	10	13	19
H12	4	9	7	3	22
H13	8	20	11	13	21
H14	2	1	1	1	1
H15	2	2	2	2	2
H16	6	13	10	9	13
H17	1	1	1	1	0
H18	1	1	1	1	0
**Segment 6 (N)**					
N1	47	250	73	198	476
N2	90	280	130	276	733
N3	17	29	21	21	27
N4	6	15	10	5	17
N5	7	14	7	10	30
N6	15	45	24	33	94
N7	12	31	19	17	29
N8	20	52	30	26	103
N9	5	20	6	15	37
N10	1	1	1	1	0
N11	1	2	1	2	0

full dataset including reverse-complement sequences, removing reverse-complement representatives post-hoc from the original results, and re-clustering after removing all reverse-complement sequences from the input (see Tab. S6). The presence of reverse-complement sequences substantially inflated the number of representatives across all tools, demonstrating that a considerable fraction of clusters in the uncorrected results arose purely from strand orientation artifacts rather than genuine sequence diversity. Notably, post-hoc removal of reverse-complement representatives and full re-clustering produced comparable results for most tools, suggesting that `ViralClust`'s automatic orientation correction prior to clustering is sufficient to avoid these artifacts without requiring a separate filtering step.

Given the variable results across tools and segments, we adjusted parameter ranges of `ViralClust` accordingly, with sequence similarity threshold between 70 and 90 %, see Tab. S7. CD-HIT-EST only allows for a minimum of 80 %. In the case of HDBSCAN, we tested k-mers from 1 to 7, see Tab. S8. The choice of sequence similarity threshold directly reflects the biological question: a higher threshold (e.g. 90 %) retains finer sequence-level differences and is appropriate when the goal is to collapse near-identical sequences while preserving all circulating variants within a subtype, such as for outbreak subsampling. A lower threshold (e.g. 70 %) merges more divergent sequences into the same cluster and is better suited for broader comparative analyses, such as selecting one representative per subtype or lineage across a diverse dataset. For *Alphainfluenzavirus influenzae*, where 18 H and 11 N subtypes span considerable sequence diversity, the threshold choice determines whether the resulting representative set captures subtype-level or strain-level di-

versity. As expected, the number of clusters decreases with decreasing similarity threshold for CD-HIT-EST, SUMACLUST, and VSEARCH (see Fig. 8 and S23), confirming that these tools respond predictably to threshold changes and offer users intuitive control over clustering granularity. This predictable behavior makes threshold selection straightforward for CD-HIT-EST, SUMACLUST, and VSEARCH: users can inspect the resulting cluster counts at different thresholds, as reported in Tab. S7, and select the threshold that best matches the expected number of biological units for their dataset. MMSeqs2 using the default module implemented in ViralClust, however, shows an atypical pattern: cluster counts remain roughly stable or occasionally increase at lower thresholds. We hypothesize that this behavior stems from the easy-linclust mode used within ViralClust, which is optimized for speed and may not handle the full combinatorial space of pairwise comparisons in datasets of this scale, leading to threshold-insensitive results, despite the fact that this module is described to be designed to handle large datasets. Users working with similarly large virus datasets should be aware of this limitation and consider validating MMSeqs2 results against those of other tools. Moreover, users can choose the easy-cluster module in ViralClust instead (--mmseqs_mode cluster). Among the tools evaluated, CD-HIT-EST and VSEARCH offer the most predictable and computationally tractable behavior for large segmented virus datasets, while HDBSCAN provides the finest resolution but at the cost of potentially missing rare subtypes. SUMACLUST and MMSeqs2 occupy intermediate positions, with MMSeqs2 easy-linclust requiring additional caution at large dataset sizes.

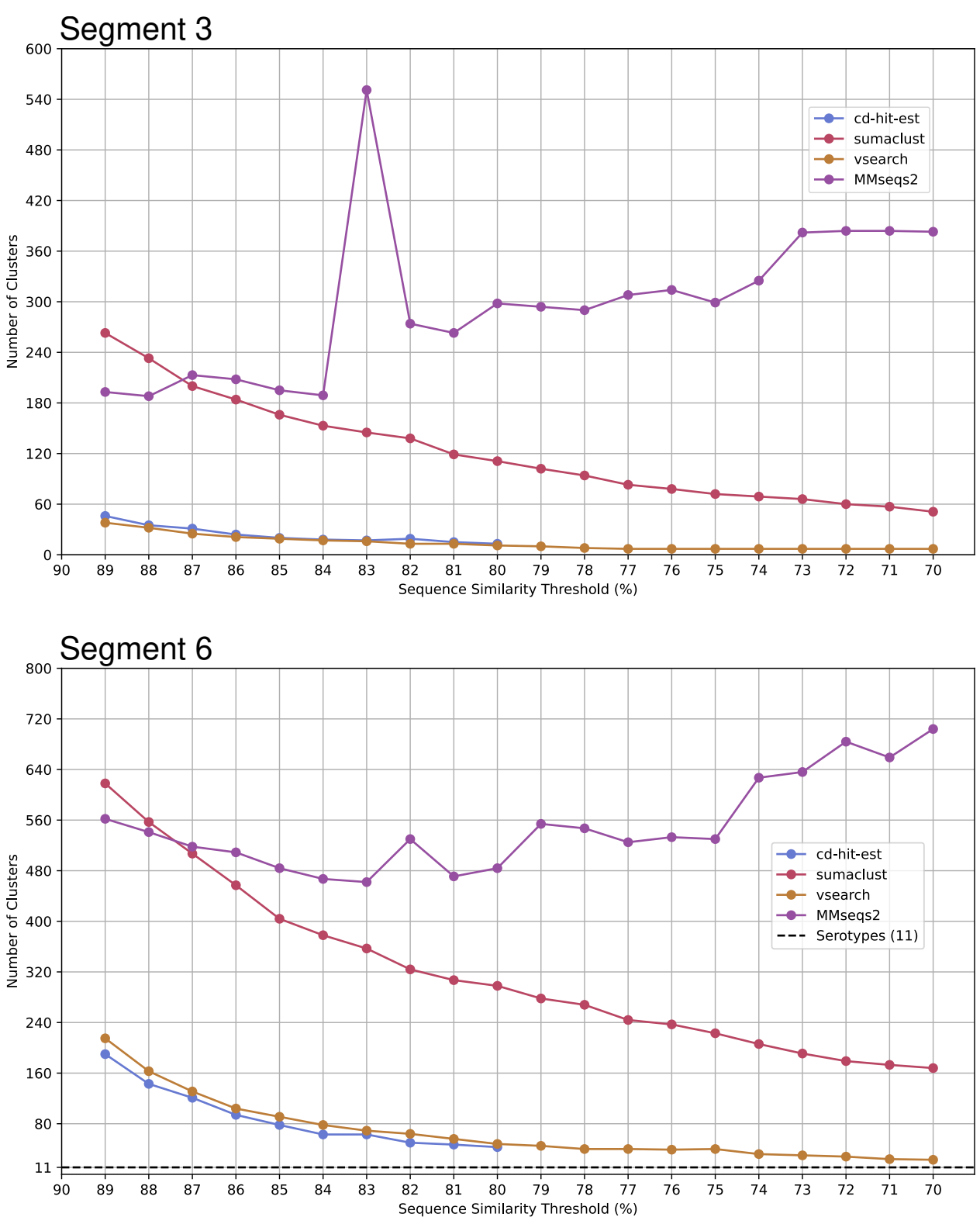

Figure 8. Number of clusters of IAV segments 3 and 6 with sequence similarity thresholds from 70 - 90 %. CD-HIT-EST only allows a minimum of 80 %. Only clusters with with a size greater than one are considered. For more details see Tab. S7 and S8.

Monkeypox virus as DNA virus example

To assess the challenges posed by long, repetitive DNA genomes to clustering algorithms, we downloaded 1,077 *Monkeypox virus* genomes (family *Poxviridae*, see Fig S24), with an average length of 197.185 kbp. 92.76 % of the sequences are non-redundant. The main

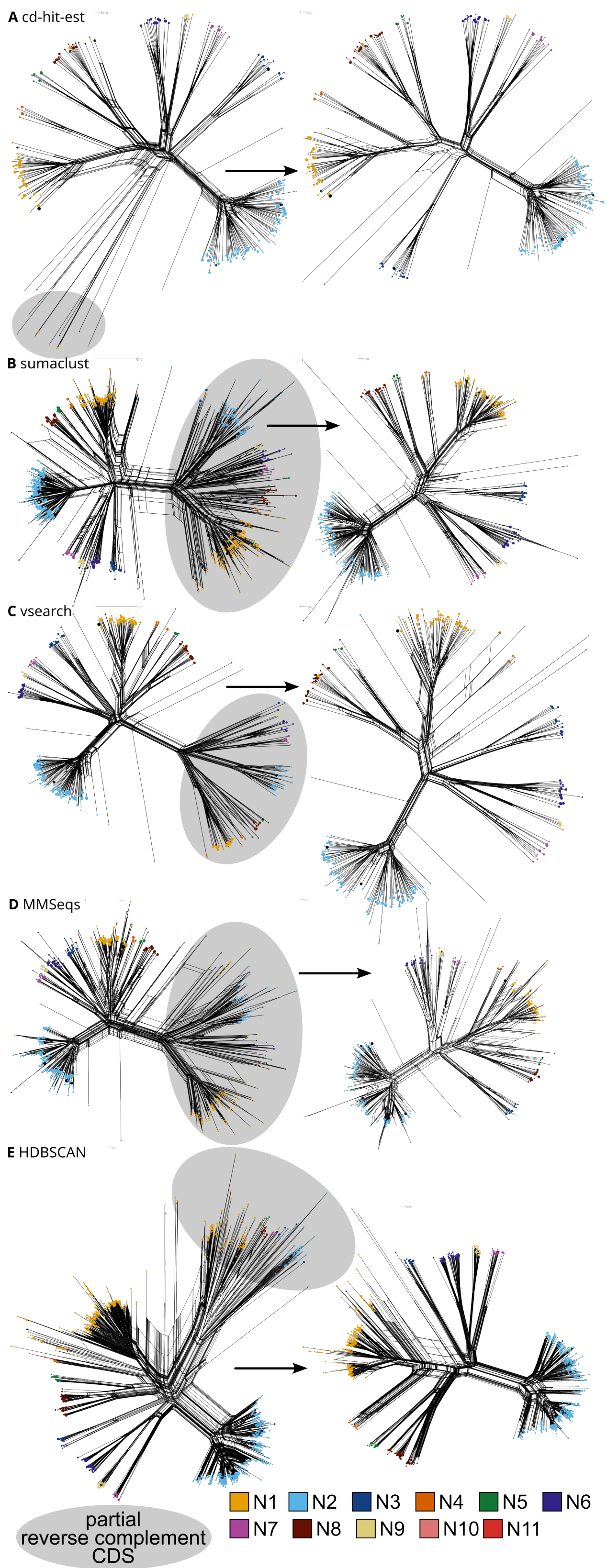

Figure 9. Split graphs of IAV segment 6 (N) representatives of (A) CD-HIT-EST, (B) SUMACLUST, (C) VSEARCH, (D) MMSeqs2, and (E) HDBSCAN. Left – all representatives (including partial and reverse-complement); Right – only complete genomes. The node size indicates cluster size. The split graphs are reconstructed using SplitsTree based on the MAFFT alignments. Gray background highlights reverse-complement sequences (11.37 % of the non-redundant dataset). HDBSCAN did not select representatives for subtypes N10 and N11. They were manually and marked them as framed circles. For details see Fig. S18–S22.

challenge compared to the RNA virus datasets analyzed previously is the combination of much longer genome length and repetitive regions, which can affect both clustering performance and representative selection.

For VSEARCH and CD-HIT-EST, we increased the parameters to accommodate the longer *Monkeypox virus* genomes: `--maxseqlength 300000` (default: 50,000) and `-M 2500` (default: 400). This dataset required the most time and memory overall. Based on the two known genotypes (clade I and II), a biologically meaningful clustering would be expected to produce two or more clusters capturing this known diversity. SUMACLUST, HDBSCAN, and VSEARCH produced between 1 and 3 clusters, whereas CD-HIT-EST (14) and MMSeqs2 (23) generated substantially more, see Tab. 1. The behavior of SUMACLUST is particularly noteworthy: approximately 66 % of sequences (662 genomes) were discarded during clustering, most likely due to non-ACGT characters in repetitive genomic regions. This behavior causes the results from SUMACLUST to be unusable for subsequent analyses, since the cluster representatives cannot serve as representatives for the original dataset. Although CD-HIT-EST generated more clusters, it assigned 917 sequences to a single cluster. In contrast, HDBSCAN and VSEARCH produced largest clusters containing 566 and 565 sequences, respectively. HDBSCAN successfully assigned all sequences to clusters, while the other algorithms left between 1 and 69 sequences unclustered (SUMACLUST and VSEARCH: 1, CD-HIT-EST: 10, MMSeqs2: 69). CD-HIT-EST and MMSeqs2 selected more representatives than the other tools. This difference likely reflects the underlying algorithmic principles: CD-HIT-EST uses a greedy incremental clustering approach that tends to select fewer representatives by aggressively merging similar sequences, whereas MMSeqs2 implements a more sensitive, iterative clustering procedure that preserves more distinct representatives, especially in large, repetitive genomes. Whether finer granularity is desirable depends on the research question. For genotype- or subtype-level analyses, the higher resolution of CD-HIT-EST or MMSeqs2 may be advantageous,

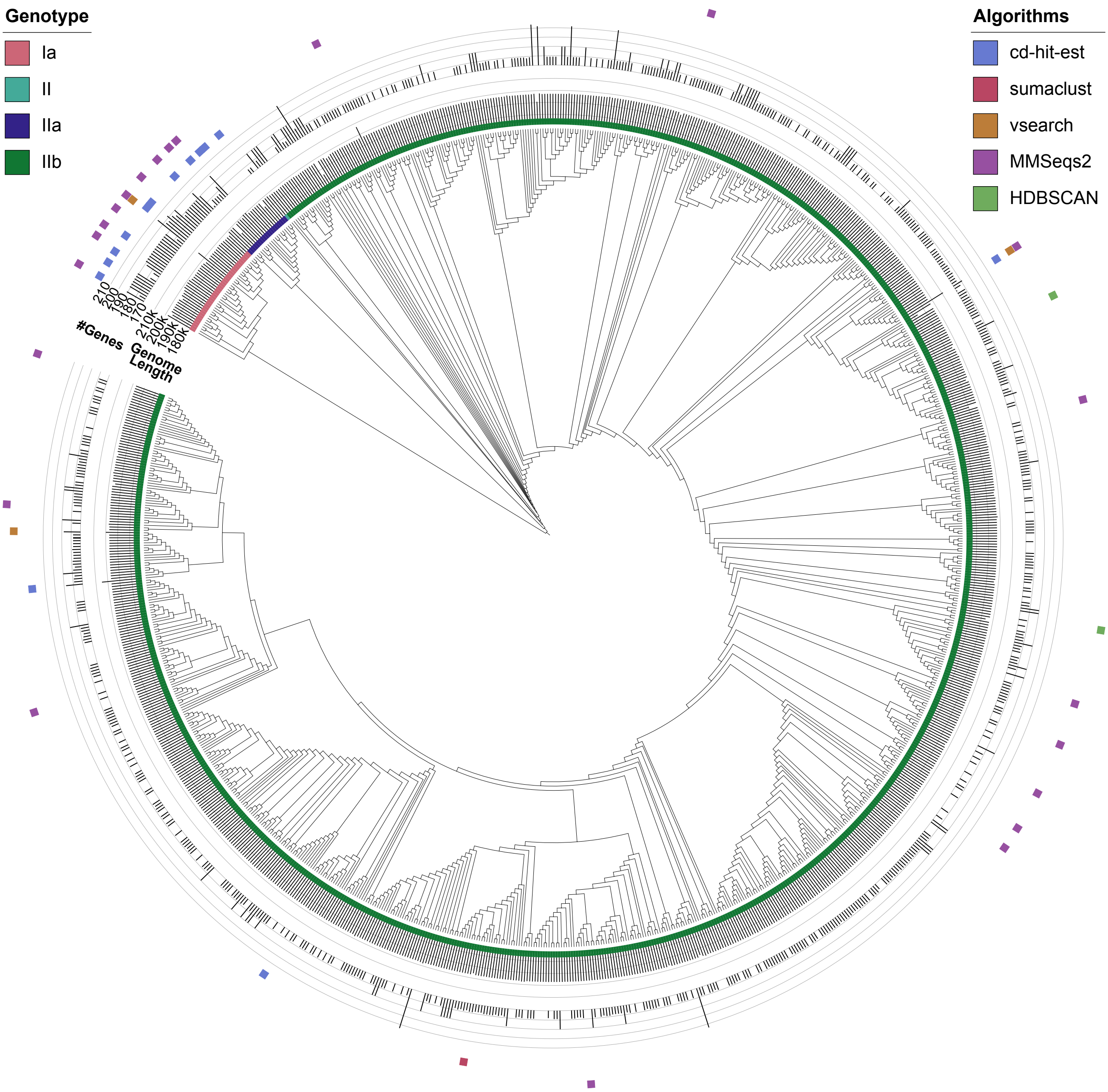

Figure 10. Phylogenetic reconstruction of the *Monkeypox virus* dataset with (i) genotypes, (ii) genome length, (iii) number of genes, and (iv) representative sequences selected by CD-HIT-EST, SUMACLUST, VSEARCH, MMSeqs2, and HDBSCAN. The tree is reconstructed using `parsnp` based on the complete genome sequences (999 genomes) resulting from the first filtering steps of `ViralClust`.

while the compact output of HDBSCAN or VSEARCH may be preferable when the goal is maximal dataset reduction. The majority of the *Monkeypox virus* dataset consists of genotype IIb (see Fig. 10). The representatives selected by SUMACLUST and HDBSCAN reflect this dominant genotype, whereas VSEARCH representatives cover genotypes Ia and IIb. Both CD-HIT-EST and MMSeqs2 resulted in representatives covering all genotypes present in the dataset. This result highlights a broader principle: tools that produce more clusters are not inherently better or worse, but in outbreak-biased datasets dominated by a single genotype, higher cluster granularity may be necessary to ensure that minority lineages are retained in the representative set. For the *Monkeypox virus* dataset, VSEARCH offers the most complete genotypic coverage while preserving a number of clusters close to the number of genoytpes. CD-HIT-EST and MMSeqs2 therefore offer a finer resolution, which may be because of their higher sensitivity to sequence-level variation in repetitive genomic regions.

Discussion

The rapid increase in publicly available viral genome sequences poses substantial challenges for downstream analyses, including multiple sequence alignment, phylogenetic inference, and comparative genomics. Large and unevenly sampled datasets are often computationally infeasible to analyze in full - particularly for analyses such as multiple sequence alignment, phylogenetic inference, and comparative genomics - and are prone to biases introduced by outbreak-driven over-representation and technical inconsistencies. In this study, we address these challenges by presenting ViralClust, a modular pipeline designed to reduce large viral genome collections through clustering-based representative selection while preserving genetic diversity.

A key outcome of our analyses is that clustering-based reduction provides a robust alternative to random subsampling. Across six diverse RNA and DNA virus datasets, representative genome sets retained biologically meaningful diversity while reducing dataset size by 95 % on average. Importantly, clustering results were stable across taxonomic scales: representative sets generated at the family level still captured genotypes defined at the species level, as demonstrated for *Hepacivirus hominis* and members of the *Flaviviridae*. This observation indicates that representative selection does not necessarily obscure finer-scale evolutionary structure and can support comparative analyses across broad taxonomic ranges.

While the present evaluation focused on the preservation of phylogenetic topology and genetic diversity, the utility of representative selection extends to biologically relevant metadata such as host range, geographic distribution, temporal dynamics, and phenotypic characteristics. To the extent that such traits are reflected in genome sequence - as is the case, for example, for host-associated sequence signatures - clustering-based representative selection inherently captures this variation alongside genetic diversity. More broadly, the degree to which a representative set covers features of interest depends on the underlying research question, and we encourage users to evaluate their representative sets accordingly. As an example, we have previously shown in the context of Pestiviruses [50] that representatives selected by clustering adequately covered the host range present in the full dataset, supporting the biological relevance of the reduced sequence set beyond phylogenetic structure alone.

The evaluation further shows that clustering whole viral genomes is feasible across a wide range of genome sizes and architectures. Even large DNA virus genomes, such as those of *Monkeypox virus*, could be processed efficiently, demonstrating the scalability of the approach. In addition, dedicated pre-processing steps proved essential for obtaining interpretable clustering results. These findings emphasize that representative selection is not solely dependent on the clustering algorithm itself but also on careful data preparation and evaluation.

We note that recombination can influence clustering outcomes by generating conflicting similarity patterns across genomic regions, potentially leading to context-dependent results. This limitation is not specific to ViralClust but affects similarity- and distance-based clustering approaches in general. The extent of this effect depends on the biological question, taxonomic scale, and analyzed genomic regions. Therefore, for highly recombinant viruses, preprocessing steps such as recombination detection, masking, or restriction to conserved regions may be advisable prior to clustering, and clustering results should always be interpreted in their biological context.

Rather than identifying a single optimal clustering strategy, ViralClust integrates multiple clustering algorithms based on different underlying principles, including compactness and connectedness. This design reflects the absence of a universal ground truth for (viral) clustering and acknowledges that different biological questions may require different clustering assumptions. By enabling direct comparison of clustering outcomes within a unified framework, ViralClust supports informed methodological choices without prescribing a "best" solution. In summary, this work demonstrates that scalable, clustering-based representative selection can substantially reduce viral genome datasets while preserving relevant diversity and mitigating common sources of bias. Through its modular design and integrated evaluation, ViralClust provides a flexible framework for virology research and serves as a reproducible starting point for downstream analyses of increasingly large viral genome collections. For different biological questions other parameters of the integrated clustering tools might be tested further.

Potential implications

Representative genome sets generated by ViralClust may facilitate a wide range of downstream analyses that are currently limited by dataset size or redundancy. For example, clustering-based representative selection can support the construction of high-quality multiple sequence alignments and enable RNA secondary structure prediction using representative alignments [51, 52, 50] as employed in Rfam [53], and large-scale analyses of endogenous viral elements [54, 55]. Similarly, workflows for virus–host prediction and comparative framework analyses, such as VIDHOP [56] and POSEIDON [57], may benefit from reduced yet diverse input datasets that remain biologically representative.

More broadly, whole-genome clustering may complement gene-centric approaches commonly used in viral comparative studies. While this work does not aim to redefine virus classification, our results suggest that genome-wide similarity patterns can provide additional context for studying viral diversity across taxonomic levels. We strongly advise that clustering-based representative selection be considered as a standard preprocessing step in future viral genome analyses, enabling more balanced, interpretable, and computationally tractable studies while remaining adaptable to specific research questions and analytical goals.

Methods

Tools integrated into ViralClust

We used existing tools for several steps in the ViralClust pipeline we designed.

Pre-processing. To remove 100 % redundant genomes in the input dataset, we apply the easy-linclust module of MMSeqs2 (v14) [17, 18], with parameters `--min-seq-id 1.0`.

Clustering. Per default, `ViralClust` applies five different clustering methods: `CD-HIT-EST` (v4.8.1) [14], `SUMACLUST` (v1.0.31) [15], `VSEARCH` (v2.15) [16] from `vsearch`, `MMSeqs2` (v14) [17, 18], and `HDBSCAN` (v0.8.26) [23]. By default, `ViralClust` instructs `CD-HIT-EST`, `SUMACLUST`, `VSEARCH`, and `MMSeqs2` to cluster sequences with an identity of at least 90 % (default value of `CD-HIT-EST`). Since `MMSeqs2` offers a variety of clustering modules, we have integrated `easy-linclust` (default) and `easy-cluster`.

Connectedness Clustering. We have implemented a combination of dimension reduction and clustering in `ViralClust`. For this we used `PCA` or `UMAP` (`umap-learn` v0.4.4) [58], both implemented in `scikit-learn` (v1.2.2) [28] for dimension reduction and `HDBSCAN` for clustering [23]. Per default, we use `PCA` and reduce the dimension to either $d = 50$ or until the sum of the explained variance of the components is at least 0.7. When using `UMAP`, the dimensions are reduced to $d = 50$ with a minimum distance of 0.25, cosine distance as common usage in the context of embeddings and language [59], and random seed state of 42 as used in `UMAP`'s documentation to ensure reproducibility within this stochastic algorithm[3]. `HDBSCAN` can be seen as a hybrid approach of hierarchical-based (connectivity) and density-based (compactness) clustering approaches. For two vectors, `HDBSCAN` first calculates mutual reachability distance. Per default, we use the cosine distance implemented in `SciPy` (v1.4.1) [29] as metric. However, we allow a plethora of pre-defined distances, which can be used via the `--metric` parameter of our `HDBSCAN` module. We implemented the (standardized) Cosine (=default), Euclidean, Manhattan, Chebyshev, Minkowski, Canberra, Braycurtis, and Mahalanobis distance metrics via the `SciPy` library [29]. To accommodate the limitations of the current implementation of `HDBSCAN`, we have employed L2 normalization to preprocess the vectors. This ensures compatibility with distance calculations, as cosine distance is not supported. Subsequently, we use `HDBSCAN` with the Euclidean distance on these normalized vectors[4].

Visualization

Importantly, with `ViralClust`, our aim is not to construct a phylogeny of the viruses, but rather to ensure that the selected virus genomes are distributed evenly across the existing phylogenetic reconstruction, providing a rough overview of their diversity. Therefore, for the validation of our use cases, we additionally visualized the distribution of cluster representatives and NCBI RefSeq genomes across the phylogenetic representation with the Neighbor-net algorithm implemented in `SplitsTree` (v4.19.2) [60, 61], generating split graphs, `FastTree` (v2.1.10) [32] as time-efficient tool for phylogenetic reconstruction of large datasets, and `parsnp` (v2.1.5) [62] for core-genome-based phylogenetic reconstruction (not part of the pipeline for the original data).

Benchmarking and Scoring Metrics

All `ViralClust` runs were conducted on a Linux-based x86_64 server equipped with four AMD Opteron 6376 processors comprising 64 logical CPU cores in total (8 cores per socket with 2 threads per core) and a maximum clock frequency of 2.3 GHz. The system architecture featured eight NUMA nodes and supported AMD-V virtualization. Computational tasks were executed in a shared-memory environment using the available multi-core CPU resources.

`ViralClust` offers a first evaluation based on several statistics using the parameter `--eval`. To evaluate the performance of the five tools integrated into `ViralClust` across all six datasets presented here, we incorporated additional metrics determining the quality of the clustering output, summarized into one overall score. All scores are defined in the range [0, 1], where higher values indicate better performance, and the overall score has a maximum of 5.

Reduction Score. The Reduction Score (RS) measures how much a given tool reduces the input dataset through clustering, while accounting for sequences discarded by the clustering algorithm:

$$RS = \left(1 - \frac{\#\text{cluster}}{\#\text{sequences}}\right) \cdot \frac{\#\text{used\_sequences}}{\#\text{sequences}} \tag{1}$$

The first factor captures the proportion of sequences replaced by cluster representatives, while the second factor penalizes tools that discard a large fraction of input sequences rather than assigning them to clusters. A score of 1 indicates maximal reduction with no sequence loss.

Singleton Penalty Score. The Singleton Penalty Score (SPS) penalizes algorithms that leave many sequences unclustered, i.e. assigned to singleton clusters of size one:

$$SPS = 1 - \frac{\#\text{unclustered}}{\#\text{sequences}} \tag{2}$$

Singletons do not contribute to dataset reduction and may indicate over-fragmentation or, in the case of density-based methods such as `HDBSCAN`, sequences assigned to noise. A score of 1 indicates that no sequences were left unclustered.

Clustering Quality Score. The Clustering Quality Score (CQS) measures the separation between clusters relative to their internal cohesion, based on pairwise phylogenetic distances derived from a user-provided tree and the cluster assignment file of each tool:

$$CQS = 1 - \frac{\varnothing_{\text{intra}}}{\varnothing_{\text{intra}} + \varnothing_{\text{inter}}} \tag{3}$$

where $\varnothing_{\text{intra}}$ and $\varnothing_{\text{inter}}$ denote the average intra- and inter-cluster distances on the tree, respectively. A score close to 1 indicates well-separated clusters with low internal distances, whereas a score close to 0 indicates poor cluster separation. The CQS requires a Newick-formatted phylogenetic tree and the `.clstr` output file of each tool as input provided by `ViralClust`.

Taxonomic Concordance Score. The Taxonomic Concordance Score (TCS) measures how well the clustering reflects known taxonomy, based on NCBI taxonomic assignments of the input sequences:

$$TCS = \frac{1}{\varnothing_{\text{cluster per species}}} \tag{4}$$

where $\varnothing_{\text{cluster per species}}$ is the average number of clusters a single species is spread across. A score of 1 indicates that each species is entirely contained within a single cluster, with no fragmentation across cluster boundaries. Values below 1 indicate that species are split across multiple clusters. Note that TCS is sensitive to the completeness of NCBI taxonomic annotations and may be less reliable for novel or poorly characterized viruses.

Cluster-to-Taxonomy Ratio. The Cluster-to-Taxonomy Ratio (CTR) evaluates how closely the number of clusters produced by a tool

3 www.umap-learn.readthedocs.io/en/latest/reproducibility.html
4 as communicated in: https://github.com/scikit-learn-contrib/hdbscan/issues/69#issuecomment-317742362

matches the number of species defined by the ICTV:

$$CTR = 1 - \left|1 - \frac{\#\text{cluster}}{\#\text{ICTV species}}\right| \quad (5)$$

A score of 1 indicates that the number of clusters exactly matches the expected number of ICTV-defined species. Scores decrease symmetrically for both over- and under-clustering relative to this expectation, and are clamped to [0, 1].

Overall Score. The Overall Score (OS) aggregates all five metrics into a single summary value:

$$OS = RS + SPS + CQS + TCS + CTR \quad (6)$$

All five component scores contribute equally, each with a maximum of 1, giving an overall maximum of 5. A higher OS indicates a clustering result that simultaneously achieves strong dataset reduction, low singleton rate, good phylogenetic cluster separation, and concordance with known taxonomy at both the sequence annotation and ICTV species level.

Availability of source code and requirements

`ViralClust` is available via GitHub: `https://github.com/rnajena/viralclust`.

Data availability

The raw sequences and datasets supporting the results of this article are available in the zenodo repository: `https://doi.org/10.5281/zenodo.18413373`.

Declarations

Ethical Approval

Not applicable

Consent for publication

Not applicable

Competing Interests

No competing interest is declared.

Funding

This work was supported by the Deutsche Forschungsgemeinschaft (DFG, German Research Foundation) under Germany's Excellence Strategy - EXC 2051 - Project-ID 390713860; the BMBF-funded project ADAPTI-M - Projetc-ID 031L0322H, and the Deutsche Forschungsgemeinschaft (DFG, German Research Foundation) under NFDI4Microbiota - NFDI 28/1 - Project-ID 460129525.

Author's Contributions

Sandra Triebel: Data curation; Investigation; Software; Formal analysis; Validation; Visualization; Writing – review & editing. Tom Eulenfeld: Methodology; Software; Formal analysis. Kevin Lamkiewicz: Data curation; Investigation; Methodology; Software; Formal analysis; Validation; Visualization; Writing – original draft. Manja Marz: Supervision; Conceptualization; Validation; Writing – review & editing.

Acknowledgements

We thank the `Nextflow` fairy Marie Lataretu! We further thank Franziska Hufsky for her `InkScape` magic.

References

1. Sharma D, Priyadarshini P, Vrati S. Unraveling the Web of Viroinformatics: Computational Tools and Databases in Virus Research. J Virol 2014;89(3):1489–1501.
2. Paez-Espino D, Eloe-Fadrosh EA, Pavlopoulos GA, Thomas AD, Huntemann M, Mikhailova N, et al. Uncovering Earth's virome. Nature 2016;536:425–430.
3. Roux S, , Brum JR, Dutilh BE, Sunagawa S, Duhaime MB, et al. Ecogenomics and potential biogeochemical impacts of globally abundant ocean viruses. Nature 2016;537(7622):689–693.
4. Wilson WH, Gilg IC, Moniruzzaman M, Field EK, Koren S, LeCleir GR, et al. Genomic exploration of individual giant ocean viruses. The ISME Journal 2017;11(8):1736–1745.
5. Roux S, Adriaenssens EM, Dutilh BE, Koonin EV, Kropinski AM, Krupovic M, et al. Minimum Information about an Uncultivated Virus Genome (MIUViG). Nat Biotechnol 2018;37(1):29–37.
6. Hufsky F, Abecasis AB, Babaian A, Beck S, Brierley L, Dellicour S, et al. The International Virus Bioinformatics Meeting 2023. Viruses 2023;15(10):2031.
7. Göttsch W, Beerenwinkel N, Deng L, Dölken L, Dutilh BE, Erhard F, et al. ITN—VIROINF: Understanding (Harmful) Virus-Host Interactions by Linking Virology and Bioinformatics. Viruses 2021;13(5):766.
8. Li H. Minimap2: pairwise alignment for nucleotide sequences. Bioinformatics (Oxford, England) 2018;34(18):3094–3100.
9. Moshiri N. ViralMSA: massively scalable reference-guided multiple sequence alignment of viral genomes. Bioinformatics (Oxford, England) 2021;37(5):714–716.
10. Chao J, Tang F, Xu L. Developments in Algorithms for Sequence Alignment: A Review. Biomolecules 2022 Apr;12(4):546.
11. Ondov BD, Treangen TJ, Melsted P, Mallonee AB, Bergman NH, Koren S, et al. Mash: fast genome and metagenome distance estimation using MinHash. Genome Biology 2016;17(1):132.
12. Ren J, Bai X, Lu YY, Tang K, Wang Y, Reinert G, et al. Alignment-Free Sequence Analysis and Applications. Annual Review of Biomedical Data Science 2018;1:93–114.
13. Moeckel C, Mareboina M, Konnaris MA, Chan CSY, Mouratidis I, Montgomery A, et al. A survey of k-mer methods and applications in bioinformatics. Computational and Structural Biotechnology Journal 2024;23:2289–2303.
14. Li W, Godzik A. Cd-hit: a fast program for clustering and comparing large sets of protein or nucleotide sequences. Bioinformatics 2006;22:1658–1659.
15. SUMATRA and SUMACLUST: fast and exact comparison and clustering of sequences.;. http://metabarcoding.org/sumatra.
16. Rognes T, Flouri T, Nichols B, Quince C, Mahé F. VSEARCH: a versatile open source tool for metagenomics. PeerJ 2016;4:e2584.
17. Steinegger M, Söding J. MMseqs2 enables sensitive protein sequence searching for the analysis of massive data sets. Nature Biotechnology 2017;35(11):1026–1028.
18. Steinegger M, Söding J. Clustering huge protein sequence sets in linear time. Nat Commun 2018;9(1).
19. Expansion of the global RNA virome reveals diverse clades of bacteriophages. Cell 2022 Oct;185(21):4023–4037.e18.
20. SARS-CoV-2 variants evolve convergent strategies to remodel

the host response. Cell 2023 Oct;186(21):4597–4614.e26.
21. Du Y, Xia J, Wang Z, Xu J, Ji Y, Jin Y, et al. Evolution of H6N6 viruses in China between 2014 and 2019 involves multiple reassortment events. Emerging Microbes & Infections 2024 Apr;p. 2341142.
22. Handl J, Knowles J, Kell DB. Computational cluster validation in post-genomic data analysis. Bioinformatics 2005;21(15):3201–3212.
23. McInnes L, Healy J, Astels S. HDBSCAN: Hierarchical density based clustering. J Open Source Software 2017;2(11):205.
24. Lefkowitz EJ, Dempsey DM, Hendrickson RC, Orton RJ, Siddell SG, Smith DB. Virus taxonomy: the database of the International Committee on Taxonomy of Viruses (ICTV). Nucleic Acids Research 2017 10;46(D1):D708–D717.
25. Walker PJ, Siddell SG, Lefkowitz EJ, Mushegian AR, Adriaenssens EM, Alfenas-Zerbini P, et al. Recent changes to virus taxonomy ratified by the International Committee on Taxonomy of Viruses (2022). Archives of Virology 2022;167(11):2429–2440.
26. Di Tommaso P, Chatzou M, Floden EW, Barja PP, Palumbo E, Notredame C. Nextflow enables reproducible computational workflows. Nat Biotechnol 2017;35(4):316.
27. Anaconda Software Distribution. Anaconda Inc.; 2020. https://docs.anaconda.com/.
28. Pedregosa F, Varoquaux G, Gramfort A, Michel V, Thirion B, Grisel O, et al. Scikit-learn: Machine Learning in Python. Journal of Machine Learning Research 2011;12:2825–2830.
29. Virtanen P, Gommers R, Oliphant TE, Haberland M, Reddy T, Cournapeau D, et al. SciPy 1.0: fundamental algorithms for scientific computing in Python. Nature methods 2020;17:261–272.
30. NCBI RC. Database Resources of the National Center for Biotechnology Information. Nucleic Acids Res 2017;45(D1):D12.
31. Katoh K, Standley DM. MAFFT multiple sequence alignment software version 7: improvements in performance and usability. Mol Biol Evol 2013;30(4):772–780.
32. Price MN, Dehal PS, Arkin AP. FastTree 2 – Approximately Maximum-Likelihood Trees for Large Alignments. PLoS ONE 2010;5(3):e9490.
33. Junier T, Zdobnov EM. The Newick utilities: high-throughput phylogenetic tree processing in the UNIX shell. Bioinformatics 2010;26(13):1669–1670.
34. JUKES TH, CANTOR CR. CHAPTER 24 - Evolution of Protein Molecules. In: MUNRO HN, editor. Mammalian Protein Metabolism Academic Press; 1969.p. 21–132.
35. Biedenkopf N, Bukreyev A, Chandran K, Di Paola N, Formenty PBH, Griffiths A, et al. ICTV Virus Taxonomy Profile: Filoviridae 2024. Journal of General Virology 2024;105(2).
36. Simmonds P, Becher P, Bukh J, Gould EA, Meyers G, Monath T, et al. ICTV Virus Taxonomy Profile: Flaviviridae. J Gen Virol 2017;98(1):2–3.
37. Simmonds P, Bukh J, Combet C, Deléage G, Enomoto N, Feinstone S, et al. Consensus Proposals for a Unified System of Nomenclature of Hepatitis C Virus Genotypes*. Hepatology 2005;42(4).
38. Smith DB, Bukh J, Kuiken C, Muerhoff AS, Rice CM, Stapleton JT, et al. Expanded classification of hepatitis C virus into 7 genotypes and 67 subtypes: Updated criteria and genotype assignment web resource. Hepatology 2014;59(1):318–327.
39. Borgia SM, Hedskog C, Parhy B, Hyland RH, Stamm LM, Brainard DM, et al. Identification of a Novel Hepatitis C Virus Genotype From Punjab, India: Expanding Classification of Hepatitis C Virus Into 8 Genotypes. The Journal of Infectious Diseases 2018;218(11):1722–1729.
40. Morel V, Descamps V, François C, Fournier C, Brochot E, Capron D, et al. Emergence of a genomic variant of the recombinant 2k/1b strain during a mixed Hepatitis C infection: A case report. Journal of Clinical Virology 2010;47(4):382–386.
41. Newman RM, Kuntzen T, Weiner B, Berical A, Charlebois P, Kuiken C, et al. Whole Genome Pyrosequencing of Rare Hepatitis C Virus Genotypes Enhances Subtype Classification and Identification of Naturally Occurring Drug Resistance Variants. The Journal of Infectious Diseases 2012;208(1):17–31.
42. Hostager R, Ragonnet-Cronin M, Murrell B, Hedskog C, Osinusi A, Susser S, et al. Hepatitis C virus genotype 1 and 2 recombinant genomes and the phylogeographic history of the 2k/1b lineage. Virus Evolution 2019;5(2):vez041.
43. Scheel TKH, Gottwein JM, Carlsen THR, Li YP, Jensen TB, Spengler U, et al. Efficient Culture Adaptation of Hepatitis C Virus Recombinants with Genotype-Specific Core-NS2 by Using Previously Identified Mutations. Journal of Virology 2011;85(6):2891–2906.
44. Li YP, Gottwein JM, Scheel TK, Jensen TB, Bukh J. MicroRNA-122 antagonism against hepatitis C virus genotypes 1–6 and reduced efficacy by host RNA insertion or mutations in the HCV 5' UTR. Proceedings of the National Academy of Sciences 2011;108(12):4991–4996.
45. Gottwein JM, Jensen SB, Serre SBN, Ghanem L, Scheel TKH, Jensen TB, et al. Adapted J6/JFH1-Based Hepatitis C Virus Recombinants with Genotype-Specific NS4A Show Similar Efficacies against Lead Protease Inhibitors, Alpha Interferon, and a Putative NS4A Inhibitor. Antimicrobial Agents and Chemotherapy 2013;57(12):6034–6049.
46. Lu L, Li C, Xu Y, Murphy DG. Full-length genomes of 16 hepatitis C virus genotype 1 isolates representing subtypes 1c, 1d, 1e, 1g, 1h, 1i, 1j and 1k, and two new subtypes 1m and 1n, and four unclassified variants reveal ancestral relationships among subtypes. Journal of General Virology 2014;95(7):1479–1487.
47. Wang B, Krüger L, Machnowska P, Eshetu A, Gunsenheimer-Bartmeyer B, Bremer V, et al. Characterization of a hepatitis C virus genotype 1 divergent isolate from an HIV-1 coinfected individual in Germany assigned to a new subtype 1o. Virology Journal 2019;16(1):28.
48. Jia Y, Yue W, Gao Q, Tao R, Zhang Y, Fu X, et al. Characterization of a Novel Hepatitis C Subtype, 6xj, and Its Consequences for Direct-Acting Antiviral Treatment in Yunnan, China. Microbiology Spectrum 2021;9(1).
49. Tokita H, Okamoto H, Iizuka H, Kishimoto J, Tsuda F, Miyakawa Y, et al. The entire nucleotide sequences of three hepatitis C virus isolates in genetic groups 7-9 and comparison with those in the other eight genetic groups. Journal of General Virology 1998;79(8):1847–1857.
50. Triebel S, Eulenfeld T, Ontiveros-Palacios N, Sweeney B, Tautz N, Marz M. First full-genome alignment representative for the genus *Pestivirus*. RNA 2025;.
51. Lamkiewicz K, Esquivel Gomez LR, Kühnert D, Marz M. Genome structure, life cycle, and taxonomy of coronaviruses and the evolution of SARS-CoV-2. Viral Fitness and Evolution: Population Dynamics and Adaptive Mechanisms 2023;p. 305–339.
52. Triebel S, Lamkiewicz K, Ontiveros N, Sweeney B, Stadler PF, Petrov AI, et al. Comprehensive survey of conserved RNA secondary structures in full-genome alignment of Hepatitis C virus. Scientific Reports 2024;14(1):15145.
53. Ontiveros-Palacios N, Cooke E, Nawrocki EP, Triebel S, Marz M, Rivas E, et al. Rfam 15: RNA families database in 2025. Nucleic Acids Research 2025;53(D1):D258–D267.
54. Ritsch M, Brait N, Harvey E, Marz M, Lequime S. Endogenous viral elements: insights into data availability and accessibility. Virus Evolution 2024;10(1):veae099.
55. Ritsch M, Eulenfeld T, Lamkiewicz K, Schoen A, Weber F, Hölzer M, et al. Endogenous Bornavirus-like Elements in Bats: Evolutionary Insights from the Conserved Riboviral L-Gene in Microbats and Its Antisense Transcription in *Myotis daubentonii*. Viruses 2024;16.
56. Mock F, Viehweger A, Barth E, Marz M. VIDHOP, vi-

ral host prediction with Deep Learning. Bioinformatics 2020;37(3):318–325.
57. Hölzer M, Marz M. PoSeiDon: a Nextflow pipeline for the detection of evolutionary recombination events and positive selection. Bioinformatics 2020 07;37(7):1018–1020.
58. McInnes L, Healy J, Melville J. UMAP: Uniform manifold approximation and projection for dimension reduction. arXiv 2018;.
59. Viehweger A, Krautwurst S, Parks DH, König B, Marz M. An encoding of genome content for machine learning. bioRxiv 2019;.
60. Bryant D. Neighbor-Net: An Agglomerative Method for the Construction of Phylogenetic Networks. Molecular Biology and Evolution 2003;21(2):255–265.
61. Huson DH, Bryant D. Application of Phylogenetic Networks in Evolutionary Studies. Molecular Biology and Evolution 2006;23(2):254–267.
62. Kille B, Nute MG, Huang V, Kim E, Phillippy AM, Treangen TJ. Parsnp 2.0: scalable core-genome alignment for massive microbial datasets. Bioinformatics 2024;40(5):btae311.
63. Likos AM, Sammons SA, Olson VA, Frace AM, Li Y, Olsen-Rasmussen M, et al. A tale of two clades: monkeypox viruses. Journal of General Virology 2005 Oct;86(10):2661–2672.
64. Bào Y, Amarasinghe GK, Basler CF, Bavari S, Bukreyev A, Chandran K, et al. Implementation of Objective PASC-Derived Taxon Demarcation Criteria for Official Classification of Filoviruses. Viruses 2017;9.
65. Kuhn JH, Amarasinghe GK, Basler CF, Bavari S, Bukreyev A, Chandran K, et al. ICTV Virus Taxonomy Profile: Filoviridae. The Journal of general virology 2019;100:911–912.

Click here to access/download
Supplementary Material
STriebel_ViralClust_supplementary_information_revised.
pdf
