# Supplementary material for: Reducing haystacks to needles—ViralClust: a Nextflow pipeline to cluster viral sequences
Source: Gigascience. 2026 Sep 14;15:giag090. doi: 10.1093/gigascience/giag090 (PMC13660479; doi:10.1093/gigascience/giag090)
Supplement: Supplementary file 6 [file giag090_reviewer_1_report_original_submission.pdf]

Reviewer Report

Title: Reducing haystacks to needles - ViralClust: A Nextflow pipeline to cluster viral sequences

Version: Original Submission **Date:** 2/11/2026

Reviewer name: Reviewer 1

Reviewer Comments to Author:

The authors present ViralClust, a modular Nextflow pipeline designed to address the computational challenges and sampling biases associated with the rapid accumulation of viral genome sequences. By integrating five distinct clustering algorithms (CD-HIT-EST, SUMACLUST, VSEARCH, MMSeqs2, and HDBSCAN), the pipeline offers a complete workflow from data pre-processing to representative sequence selection. The authors evaluated the tool on six viral datasets of varying size and type, including Orthoebolavirus, Hepacivirus, Influenza A, and Monkeypox virus.

This is a highly practical tool, particularly given the context of rapidly expanding viral genomic databases. The manuscript is well-structured, and the test datasets are representative, covering diverse viral characteristics. However, there is room for improvement regarding the analysis of specific algorithm performance, user guidance, and the presentation of results.

Specific Comments:

1. Currently, ViralClust functions more as a "toolbox" of aggregated methods rather than a mature, user-friendly "solution." Although five clustering algorithms are provided, the manuscript lacks sufficient guidance on the specific application scenarios for each. I recommend that the authors design a comprehensive scoring metric to rank the clustering results of each algorithm, providing a reference for users. Alternatively, please provide recommended algorithms or parameter presets (profiles) tailored to different analysis scenarios (e.g., specific profiles for RNA viruses, DNA viruses, or segmented viruses). This would significantly aid users in selecting the most appropriate strategy.

2. The Results section is currently somewhat verbose and repetitive. As this is a Method/Tool paper, the core focus should be on demonstrating the tool's generalizability, accuracy, and the comparative logic between different algorithms, rather than a monotonous recitation of statistics for every dataset. Please consolidate the Results into 1-2 focused sections. Avoid listing exhaustive statistics for every case study and instead focus on summarizing trends and algorithmic differences.

3. The authors mention filtering out sequences containing less than 90% ACGT characters. Ambiguous bases (e.g., N, R, Y) are very common in consensus sequences, and strict filtering may be inappropriate. For instance, in the Monkeypox analysis, SUMACLUST discarded approximately 66% of the sequences, which is a significant loss of data. Consider relaxing this filter or modifying the strategy to calculate non-ACGT content only within Open Reading Frames (ORFs) to avoid discarding potentially valuable genomic data.

4. The authors noted that the easy-linclust module of MMSeqs2 performed poorly on the large Influenza A dataset and hypothesized that easy-linclust is unsuitable for such data. Since ViralClust is explicitly aimed at handling "large-scale" data, it is insufficient to merely report this failure. I suggest the authors attempt to integrate other MMSeqs2 clustering modules, such as the easy-cluster, to see if results

improve. If an alternative module performs better, it should be implemented as an alternative option or fallback within the pipeline.

5. The evaluation reveals a critical limitation in HDBSCAN, which tends to misclassify biologically significant but low-abundance lineages as noiseâ€”evidenced by the failure to identify TAFV and rare Influenza subtypes (H17/H18, N10/N11), alongside a disproportionately high generation of singletons (e.g., 1,333 in Flaviviridae). As the current requirement to manually recover these files undermines the pipeline's automation and poses a risk of data loss, I strongly recommend implementing a parameter (e.g., --keep_singletons) to automatically include unclustered sequences in the final representative output, while also expanding the Discussion/User guidance to explicitly warn users about the trade-offs of using density-based clustering for rare variant detection.

6. Please include the maximum memory consumption (Peak RAM) for each dataset in Table 1. While runtimes are provided, memory usage is a critical factor for users handling large datasets.

Methods

Are the methods appropriate to the aims of the study, are they well described, and are necessary controls included? Choose an item.

Conclusions

Are the conclusions adequately supported by the data shown? Choose an item.

Reporting Standards

Does the manuscript adhere to the journal's guidelines on [minimum standards of reporting?]() Choose an item.

Choose an item.

Statistics

Are you able to assess all statistics in the manuscript, including the appropriateness of statistical tests used? Choose an item.

Quality of Written English

Please indicate the quality of language in the manuscript: Choose an item.

Declaration of Competing Interests

Please complete a declaration of competing interests, considering the following questions:

- Have you in the past five years received reimbursements, fees, funding, or salary from an organisation that may in any way gain or lose financially from the publication of this manuscript, either now or in the future?
- Do you hold any stocks or shares in an organisation that may in any way gain or lose financially from the publication of this manuscript, either now or in the future?
- Do you hold or are you currently applying for any patents relating to the content of the manuscript?
- Have you received reimbursements, fees, funding, or salary from an organization that holds or has applied for patents relating to the content of the manuscript?
- Do you have any other financial competing interests?
- Do you have any non-financial competing interests in relation to this paper?

If you can answer no to all of the above, write 'I declare that I have no competing interests' below. If your reply is yes to any, please give details below.

I declare that I have no competing interests

I agree to the open peer review policy of the journal. I understand that my name will be included on my report to the authors and, if the manuscript is accepted for publication, my named report including any attachments I upload will be posted on the website along with the authors' responses. I agree for my report to be made available under an Open Access Creative Commons CC-BY license (http://creativecommons.org/licenses/by/4.0/). I understand that any comments which I do not wish to be included in my named report can be included as confidential comments to the editors, which will not be published.

I agree to the open peer review policy of the journal

To further support our reviewers, we have joined with Publons, where you can gain additional credit to further highlight your hard work (see: https://publons.com/journal/530/gigascience). On publication of this paper, your review will be automatically added to Publons, you can then choose whether or not to claim your Publons credit. I understand this statement.

Yes Choose an item.
