# Supplementary material for: Reducing haystacks to needles—ViralClust: a Nextflow pipeline to cluster viral sequences
Source: Gigascience. 2026 Sep 14;15:giag090. doi: 10.1093/gigascience/giag090 (PMC13660479; doi:10.1093/gigascience/giag090)
Supplement: Supplementary file 9 [file giag090_reviewer_2_report_original_submission.pdf]

Reviewer Report

Title: Reducing haystacks to needles - ViralClust: A Nextflow pipeline to cluster viral sequences

Version: Original Submission **Date:** 3/4/2026

Reviewer name: Reviewer 2

Reviewer Comments to Author:

Sandra Triebel et al. build a modular Nextflow pipelineâ€”ViralClust for clustering large viral genome datasets and selecting representative sequences in a bias-aware way. The pipeline integrates five clustering tools (CD-HIT-EST, SUMACLUST, VSEARCH, MMSeqs2, and HDBSCAN), offers optional evaluation outputs (MSA + rough phylogenetic representations), and produces HTML summaries and per-tool representative sets. While the pipeline sounds a good tool to addresses a genuine need in viral genomics, several major concerns regarding validation, methodological clarity, and practical implementation need to be addressed.

Major Comments.

1. Although the author demonstrated that clustering preserves the phylogenetic topology, they did not adequately verify whether the selected representative strains retained biologically relevant traits such as host range, geographic distribution, temporal dynamics, or phenotypic characteristics.
2. The author present results under various parameter combinations but does not adequately explain how users should select appropriate parameters based on specific research questions, which can be confusing for those unfamiliar with the context. For example, when should users use a 70% rather than a 90% sequence similarity threshold? How does this choice affect different downstream analyses?
3. Recombination occurs across multiple viral families, and the authors did not adequately explain how recombinant sequences are handled during the clustering process. Depending on the genomic regions used to calculate similarity, recombinant genomes may yield divergent clustering outcomes. The authors should discuss whether their method is applicable to highly recombinant viruses and clarify whether additional preprocessing steps (such as recombination detection or removal) are necessary.
4. The author mentions that HDBSCAN is not suitable for large datasets and that MMseqs2 is faster but does not provide specific data on computational requirements under different dataset sizes and parameter combinations. For users handling ultra-large datasets, such information is very important for analysis planning.

Minor Comments.

1. The introduction would be more appealing if it briefly discusses existing methods for handling large-scale viral datasets (such as reference-based alignment and k-mer approaches) and explains why cluster-based representative sequence selection offers advantages in certain applications.
2. The table 4 comparing clustering results would be more informative if it included a column showing the percentage of total diversity captured by each method, perhaps using a metric like phylogenetic diversity (PD) or sum of branch lengths in the representative tree versus the full tree.
3. The split graphs of IAV segments showing partial and reverse complement sequences (Figure 7) are informative, but it would benefit from quantitative metrics on how many sequences were affected and

how correction improved clustering results. Provide a table summarizing the number of sequences in each category before and after correction.
4. The Figure 8 showing the relationship between sequence similarity threshold and number of clusters are useful, but they only show results for segments 3 and 6. Either show results for all segments in supplementary materials or explain why these two segments were chosen as representatives.
5. Discussion the statement "Large and unevenly sampled datasets are often computationally infeasible to analyze", is somewhat vague. Specify what types of analyses become infeasible, such as multiple sequence alignment.

Methods

Are the methods appropriate to the aims of the study, are they well described, and are necessary controls included? Choose an item.

Conclusions

Are the conclusions adequately supported by the data shown? Choose an item.

Reporting Standards

Does the manuscript adhere to the journal's guidelines on [minimum standards of reporting?]() Choose an item.

Choose an item.

Statistics

Are you able to assess all statistics in the manuscript, including the appropriateness of statistical tests used? Choose an item.

Quality of Written English

Please indicate the quality of language in the manuscript: Choose an item.

Declaration of Competing Interests

Please complete a declaration of competing interests, considering the following questions:

- Have you in the past five years received reimbursements, fees, funding, or salary from an organisation that may in any way gain or lose financially from the publication of this manuscript, either now or in the future?
- Do you hold any stocks or shares in an organisation that may in any way gain or lose financially from the publication of this manuscript, either now or in the future?

- Do you hold or are you currently applying for any patents relating to the content of the manuscript?
- Have you received reimbursements, fees, funding, or salary from an organization that holds or has applied for patents relating to the content of the manuscript?
- Do you have any other financial competing interests?
- Do you have any non-financial competing interests in relation to this paper?

If you can answer no to all of the above, write 'I declare that I have no competing interests' below. If your reply is yes to any, please give details below.

I declare that I have no competing interests

I agree to the open peer review policy of the journal. I understand that my name will be included on my report to the authors and, if the manuscript is accepted for publication, my named report including any attachments I upload will be posted on the website along with the authors' responses. I agree for my report to be made available under an Open Access Creative Commons CC-BY license (http://creativecommons.org/licenses/by/4.0/). I understand that any comments which I do not wish to be included in my named report can be included as confidential comments to the editors, which will not be published.

I agree to the open peer review policy of the journal

To further support our reviewers, we have joined with Publons, where you can gain additional credit to further highlight your hard work (see: https://publons.com/journal/530/gigascience). On publication of this paper, your review will be automatically added to Publons, you can then choose whether or not to claim your Publons credit. I understand this statement.

Yes Choose an item.
