# Supplementary material for: Reducing haystacks to needles—ViralClust: a Nextflow pipeline to cluster viral sequences
Source: Gigascience. 2026 Sep 14;15:giag090. doi: 10.1093/gigascience/giag090 (PMC13660479; doi:10.1093/gigascience/giag090)
Supplement: Supplementary file 11 [file giag090_supplemental_file.pdf]

Supplementary Information

`ViralClust` statistics

Table S1. Run time and memory usage of `ViralClust` steps measured by `Nextflow`. cd – `CD-HIT-EST`; sum – `SUMACLUST`; vse – `VSEARCH`; mmseq – `MMSeqs2`; hdb – `HDBSCAN`; #Sequences – number of input genomes/segments with accession date; only records labeled as `"complete"`; rem.-red. – remove redundancy; ∅ aln – average time/memory of alignment construction of representative genomes using `MAFFT`; ∅ tree – average time/memory of phylogenetic tree reconstruction of representative genomes using `FastTree`; eval – average time/memory of calculating basic cluster statistics; Peak RAM – peak RSS (resident set size) of `ViralClust` measured by `Nextflow`.

Taxonomic clade	#Sequences		Run time									
			Pre-processing		Clustering					Evaluation		
	13.10.2022	17.11.2025	sort	rem.-red.	cd	suma	vse	mmseq	hdb	∅ aln	∅ tree	eval
Orthoebolavirus	632	691	13''	7''	20''	31''	4'21''	1''	27''	58''	3''	13''
Hepacivirus hominis	1,075	1,135	9''	54''	1'20''	1'23''	5'18''	4''	37''	31''	9''	10''
Orthoflavivirus	8,052	10,332	1'14''	3' 4''	2'29''	21'13''	30'45''	5''	13'36''	1'21''	24''	11''
Flaviviridae	9,895	12,676	1'35''	4'28''	11'36''	44'35''	55'53''	10''	22'54''	3'12''	1'41''	12''
Alphainfluenzavirus influenzae (1)	83,543	155,568	2'46''	7'25''	34''	6'21''	4'32''	8''	3h 00'19''	7''	33''	15''
Alphainfluenzavirus influenzae (2)	82,569	152,464	2'42''	7' 2''	35''	5'55''	4'48''	8''	2h 58'16''	6''	28''	14''
Alphainfluenzavirus influenzae (3)	83,400	154,548	2'39''	6'12''	42''	5'16''	4'29''	8''	2h 52'25''	5''	28''	15''
Alphainfluenzavirus influenzae (4, H)	156,586	238,758	3'38''	4'58''	1'30''	36'39''	4'38''	27''	7h 24'11''	10''	3'21''	28''
Alphainfluenzavirus influenzae (5)	84,653	158,491	2' 2''	3'18''	18''	2'38''	1'57''	6''	2h 14'38''	3''	16''	13''
Alphainfluenzavirus influenzae (6, N)	114,639	191,872	2'23''	3'22''	52''	13'37''	2'24''	10''	3h 41'39''	4''	35''	16''
Alphainfluenzavirus influenzae (7)	96,928	172,393	1'28''	58''	10''	38''	1' 4''	4''	1h 39'35''	2''	15''	13''
Alphainfluenzavirus influenzae (8)	85,944	160,084	1'10''	49''	11''	39''	49''	3''	1h 22'15''	1''	9''	12''
Monkeypox virus	1,077	4,070	9'50''	3'15''	1h 17'00''	1h 38'54''	18 h 9'10''	2'45''	33'32''	1h 14'57''	2'19''	50''

Taxonomic clade	Memory usage										Peak RAM
	Pre-processing		Clustering					Evaluation			
	sort	rem.-red.	cd	suma	vse	mmseq	hdb	∅ aln	∅ tree	eval	
Orthoebolavirus	13M	58M	169M	21M	31M	3.5M	335M	1.8G	88M	6.2G	11.4G
Hepacivirus hominis	13M	47M	179M	23M	37M	46M	443M	2.3G	75M	6.3G	10.7G
Orthoflavivirus	13M	290M	279M	161M	170M	278M	5.5G	4.6G	148M	6.2G	43.2G
Flaviviridae	12M	528M	315M	193M	224M	327M	6.6G	5.1G	327M	6.5G	52.6G
Alphainfluenzavirus influenzae (1)	12M	796M	293M	286M	600M	619M	3.3G	316M	78M	6.2G	273.9G
Alphainfluenzavirus influenzae (2)	12M	792M	291M	282M	590M	605M	3.3G	303M	69M	6.1G	270.9G
Alphainfluenzavirus influenzae (3)	12M	775M	283M	270M	558M	586M	3.3G	217M	73M	6.0G	268.8G
Alphainfluenzavirus influenzae (4, H)	12M	1.1G	347M	397M	785M	752M	5.5G	298M	142M	6.0G	510.0G
Alphainfluenzavirus influenzae (5)	12M	600M	236M	183M	339M	268M	3.0G	205M	48M	6.3G	238.3G
Alphainfluenzavirus influenzae (6, N)	12M	720M	269M	247M	456M	358M	4.1G	239M	81M	6.1G	572.2G
Alphainfluenzavirus influenzae (7)	12M	347M	206M	126M	258M	17M	2.7G	142M	31M	6.0G	225.1G
Alphainfluenzavirus influenzae (8)	12M	275M	196M	105M	183M	45M	2.6G	132M	32M	6.3G	207.2G
Monkeypox virus	20M	1.2G	2.8G	259M	619M	1.2G	4.5G	260M	1.3G	6.4G	6.5G

ICTV

Table S2. "Ground truth" of virus phylogeny. Number of genera, subgenera, species, subtypes, and/or genotypes according to ICTV, NCBI, and CDC. g – genera; sg – subgenera; s – species; st – subtypes; gt – genotypes; * – `https://www.cdc.gov/flu/about/viruses-types.html` Last Reviewed: 18.09.2024

Taxonomic clade	# of	Source	Shown in	Link to ICTV
Orthoebolavirus	6 s	ICTV [35]	Fig. S1	*Orthoebolavirus*
Hepacivirus hominis	8 gt	NCBI [38, 39]	Fig. S4	*Hepacivirus*
Orthoflavivirus	53 s	ICTV [36]	Fig. S4	*Orthoflavivirus*
Flaviviridae	4 g, 97 s	ICTV [36]	Fig. S4	*Flaviviridae*
Alphainfluenzavirus influenzae	137 st (> 130)	NCBI (CDC*)	Fig. S10	*Orthomyxoviridae*
Monkeypox virus	2 gt	NCBI [63]	Fig. S24	*Orthopoxvirus*

Benchmark `ViralClust` by a variety of datasets

Table S3. Scoring metric sorted by dataset. RS – Reduction Score; SPS – Singleton Penalty Scor; CQS – Clustering Quality Score; TCS – Taxonomic Concordance Score (based on NCBI species); Overall Score – weighted sum of all scores above; IAV – *Alphainfluenzavirus influenzae*; cd – CD-HIT-EST; sum – SUMACLUST; vse – VSEARCH; mmseq – MMSeqs2; hdb – HDBSCAN;

Taxonimic clade	Algorithm	RS	SPS	CQS	TCS	CTR	OS
	cd	0.982	0.995	0.978	1.000	0.667	4.622
	suma	0.871	1.000	0.978	1.000	0.833	4.682
Orthoebolavirus	vse	0.984	1.000	0.977	0.893	0.833	4.687
	mmseq	0.975	0.991	0.987	0.820	0.167	3.940
	hdb	0.975	0.959	0.994	0.625	0.167	3.720
	cd	0.948	0.910	0.831	0.565	0.672	3.926
	suma	0.709	0.961	0.899	0.521	0.708	3.798
Hepacivirus hominis	vse	0.945	0.919	0.908	0.575	0.693	4.040
	mmseq	0.949	0.911	0.831	0.599	0.673	3.963
	hdb	0.979	0.878	0.835	0.633	0.673	3.998
	cd	0.991	0.989	0.959	0.694	0.698	4.331
	suma	0.894	0.989	0.961	0.637	0.660	4.141
Orthoflavivirus	vse	0.991	0.990	0.976	0.704	0.679	4.340
	mmseq	0.992	0.990	0.957	0.758	0.811	4.508
	hdb	0.987	0.910	0.901	0.402	0.094	3.294
	cd	0.977	0.964	0.965	0.610	0.000	3.516
	suma	0.862	0.972	0.967	0.578	0.144	3.523
Flaviviridae	vse	0.977	0.967	0.979	0.617	0.000	3.540
	mmseq	0.978	0.965	0.963	0.649	0.000	3.555
	hdb	0.980	0.859	0.705	0.478	0.000	3.022
	cd	0.999	0.999	0.550	0.990	0.884	4.422
	suma	0.929	0.994	0.956	0.990	0.983	4.852
IAV (1)	vse	0.998	1.000	0.956	0.990	0.986	4.930
	mmseq	0.995	0.982	0.619	0.943	0.885	4.424
	hdb	0.975	0.673	0.897	0.962	0.877	4.384
	cd	0.999	0.999	0.569	0.990	0.889	4.446
	suma	0.930	0.994	0.942	0.990	0.980	4.836
IAV (2)	vse	0.999	1.000	0.938	0.990	0.982	4.909
	mmseq	0.996	0.982	0.591	0.952	0.880	4.401
	hdb	0.977	0.689	0.903	0.962	0.883	4.414
	cd	0.999	0.999	0.529	0.990	0.879	4.396
	suma	0.933	0.994	0.934	0.990	0.978	4.829
IAV (3)	vse	0.999	1.000	0.940	0.990	0.982	4.911
	mmseq	0.995	0.982	0.625	1.000	0.900	4.502
	hdb	0.975	0.663	0.892	0.962	0.873	4.365
	cd	0.997	0.999	0.738	0.990	0.931	4.655
	suma	0.908	0.990	0.976	0.980	0.984	4.838
IAV (4)	vse	0.996	0.999	0.975	0.980	0.988	4.938
	mmseq	0.985	0.887	0.787	0.990	0.912	4.561
	hdb	0.979	0.689	0.940	0.971	0.895	4.474
	cd	0.999	0.999	0.520	0.990	0.877	4.385
	suma	0.952	0.995	0.951	0.990	0.983	4.871
IAV (5)	vse	0.998	1.000	0.952	0.990	0.985	4.925
	mmseq	0.996	0.988	0.586	0.962	0.883	4.415
	hdb	0.975	0.591	0.863	0.962	0.848	4.239
	cd	0.997	0.999	0.704	0.990	0.922	4.612
	suma	0.918	0.990	0.975	0.990	0.986	4.859
IAV (6)	vse	0.996	0.999	0.968	0.980	0.986	4.929
	mmseq	0.991	0.960	0.735	0.971	0.914	4.571
	hdb	0.978	0.652	0.907	0.971	0.877	4.385
	cd	0.999	1.000	0.637	1.000	0.909	4.545
	suma	0.955	0.996	0.800	0.990	0.946	4.687
IAV (7)	vse	0.999	1.000	0.829	1.000	0.957	4.785
	mmseq	0.994	0.978	0.782	1.000	0.939	4.693
	hdb	0.979	0.545	0.810	0.971	0.826	4.131
	cd	0.999	0.999	0.696	0.990	0.921	4.605
	suma	0.956	0.997	0.896	0.990	0.969	4.808
IAV (8)	vse	0.998	1.000	0.910	0.990	0.975	4.873
	mmseq	0.994	0.980	0.778	0.980	0.933	4.665
	hdb	0.981	0.583	0.863	0.971	0.849	4.247
	cd	0.986	0.990	0.514	0.200	0.000	2.690
	suma	0.337	0.999	0.475	0.000	0.500	2.311
Monkeypox virus	vse	0.997	0.999	0.998	0.667	0.500	4.161
	mmseq	0.978	0.931	0.604	0.250	0.000	2.763
	hdb	0.998	1.000	0.998	1.000	1.000	4.996

Orthoebolavirus

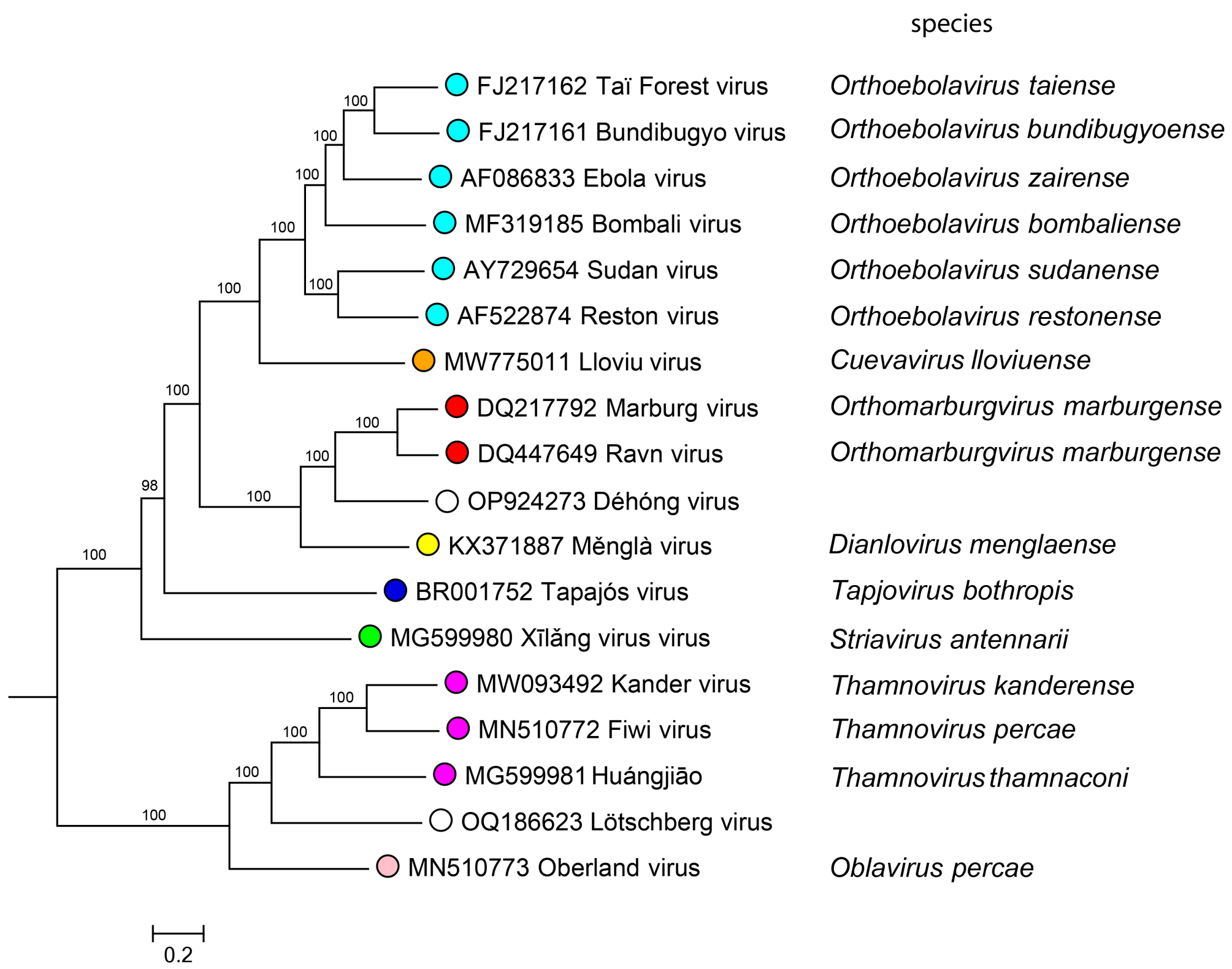

Figure S1. Phylogenetic tree of *Filoviridae* (ICTV) including *Orthoebolavirus* (cyan).

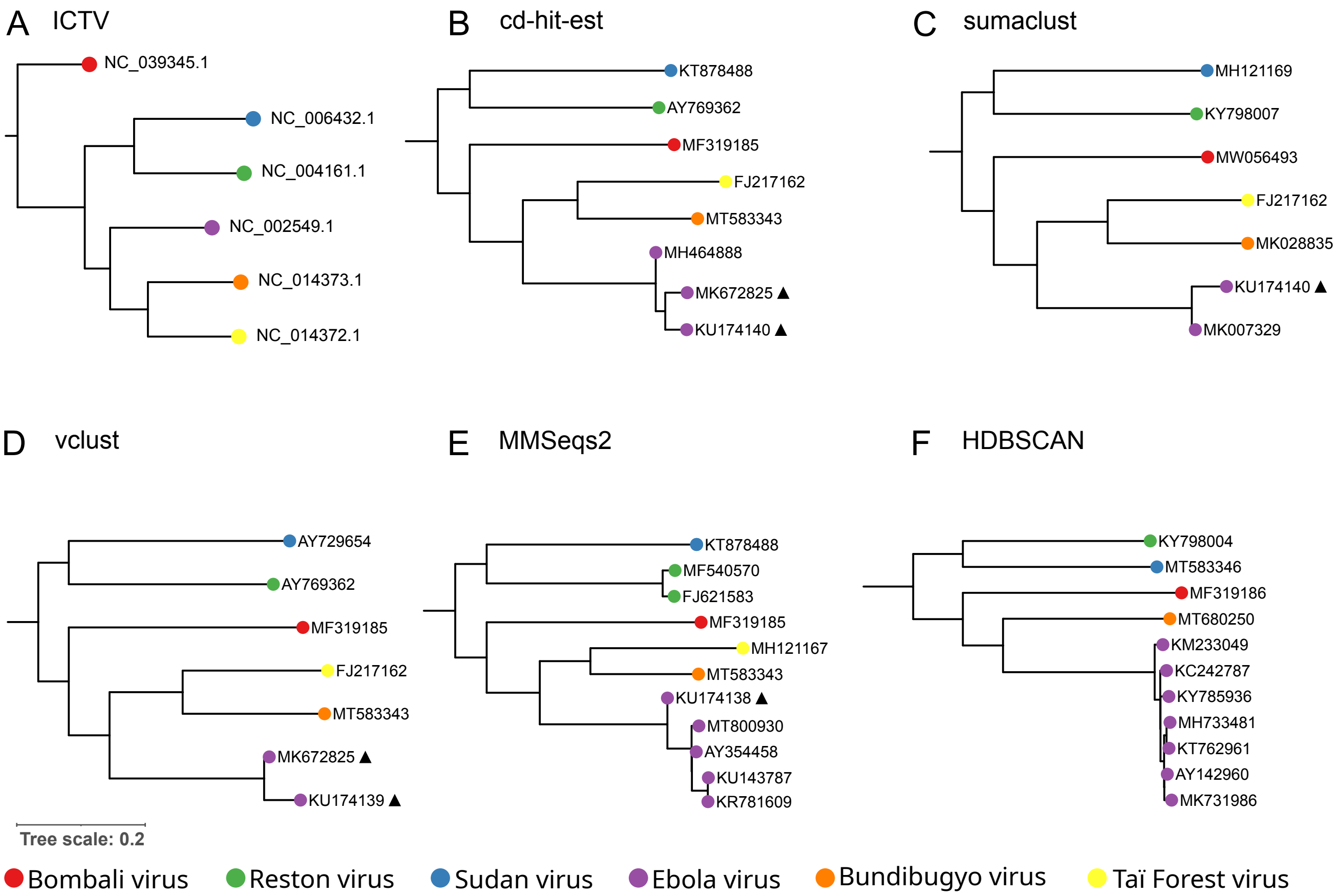

Figure S2. (A) Phylogenetic representation of ebolaviruses retrieved from the NCBI RefSeq database. The tree is based on the complete nucleotide sequence as described in the literature [64, 65]. (B–F) Phylogenetic representation of all representative ebolaviruses determined with `CD-HIT-EST`, `SUMACLUST`, `VSEARCH`, `MMSeqs2`, and `HDBSCAN`, respectively. Genomes derived from recombination or reverse genetics experiments are labeled with a triangle. Multiple sequence alignment and tree were calculated with `MAFFT` and `FastTree`, respectively.

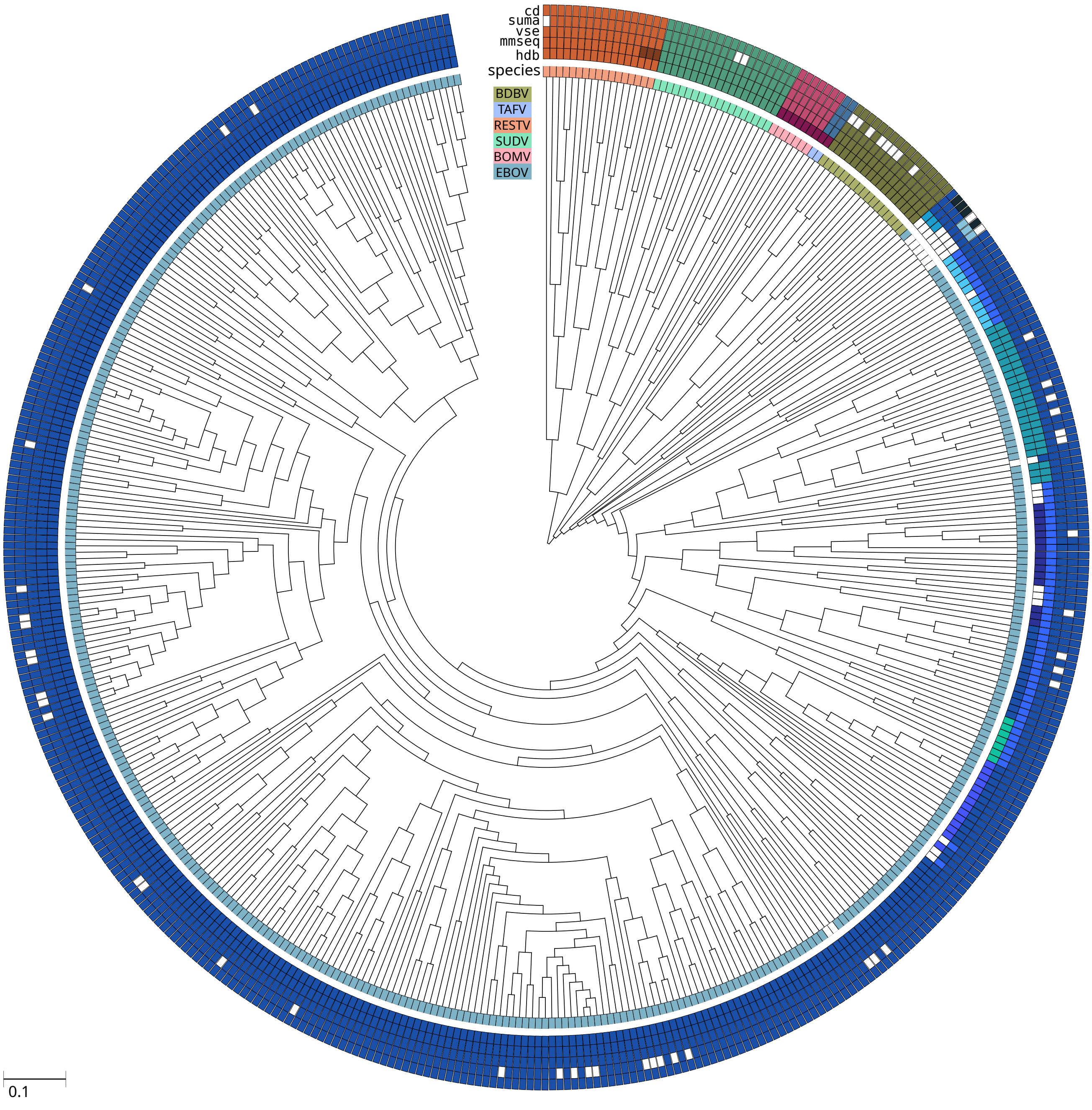

Figure S3. Phylogenetic representation of the *Orthoebolavirus* dataset. The tree is reconstructed using `RAxML-NG` based on the `MAFFT` alignment of the complete genome sequences (443 genomes) resulting from the first filtering steps of `ViralClust`. For detailed listing of parameters, we refer to the Methods section. The inner track around the tree represents the species information as retrieved from the NCBI database, serving as the 'ground truth'. Empty rectangles mark genomes for which no species information is available in the metadata. The top five tracks show the resulting clusters from `CD-HIT-EST`, `SUMACLUST`, `VSEARCH`, `MMSeqs2`, and `HDBSCAN`, respectively. Colors indicate the individual clusters identified by each tool and highlighting their level of agreement. We present singletons with empty rectangles.

Flaviviridae

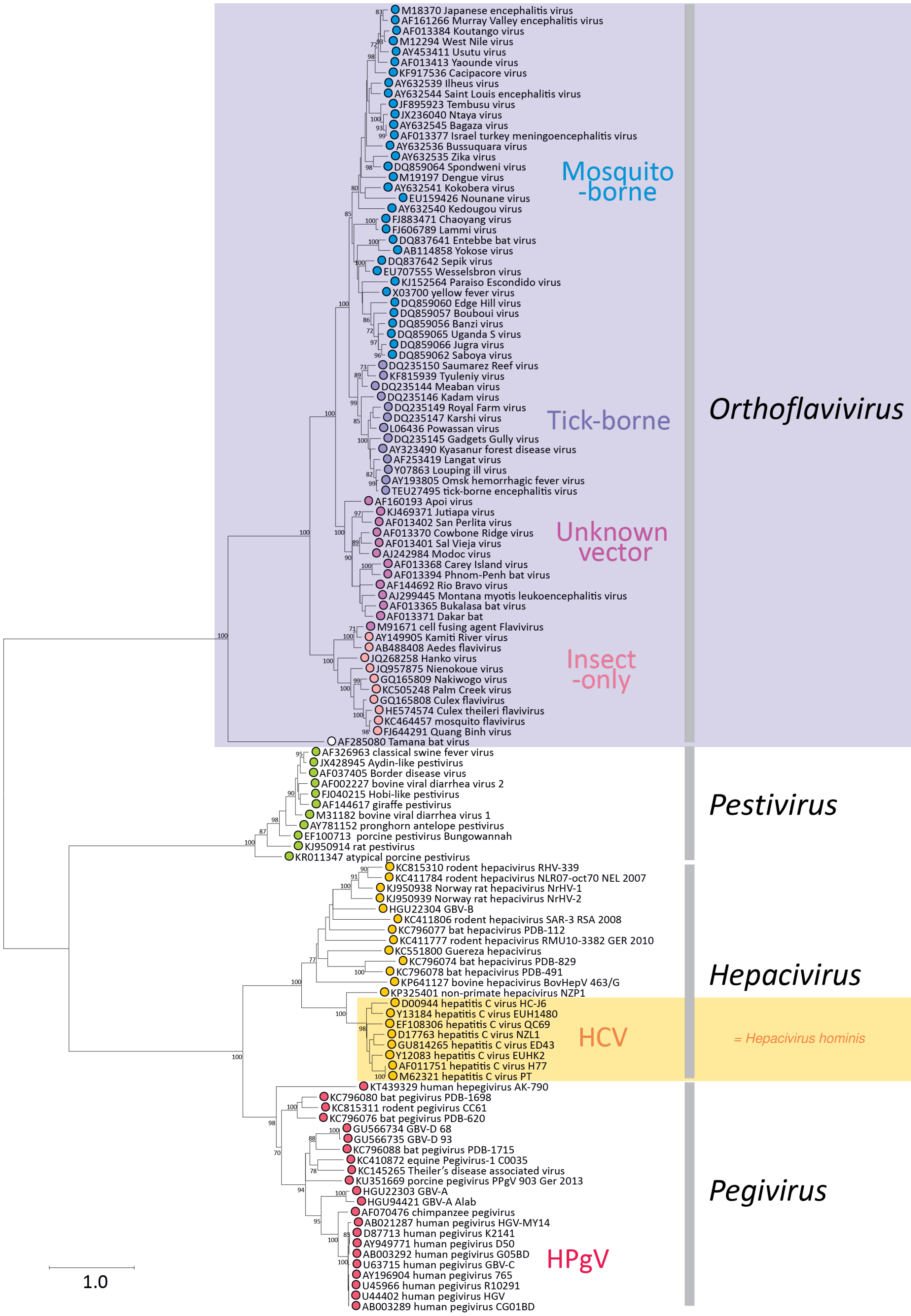

Figure S4. Phylogenetic tree of the family *Flaviviridae* (ICTV) including genus *Orthoflavivirus* (violet background) and species *Hepacivirus hominis* (HCV, yellow background). *Flaviviridae* consists of 4 genera including 97 species; *Orthoflavivirus* comprises 53 species; and *Hepacivirus hominis* includes 8 genotypes (only 7 genotypes are displayed in the tree). This tree is based on the partial gene sequences between positions 8,040–8,897 (numbered using positions in the HCV sequence, AF011751).

Table S4. Comparing the clustering results of the *Orthoflavivirus* dataset with the ICTV. ✓ – cluster with 1 ICTV representative; ∅ – number of clusters without ICTV representative; Problems – cluster with >= 2 ICTV representatives

Begin of Table

	✓	∅	Discrepancies
cd	clstr 29 - Bagaza (AY632545)	clstr 5, clstr 11, clstr 14,	clstr 143 - Saboya (NC_033697), Potiskum (NC_029054)
	clstr 30 - Dengue 2 (KF955373)	clstr 16, clstr 17, clstr 20,	18 singletons: Aroa (AY632536)
	clstr 12 - Dengue 1 (NC_001477)	clstr 22, clstr 23, clstr 26,	Banzi (NC_043110)
	clstr 76 - Dengue 3 (M93130)	clstr 27, clstr 28, clstr 31,	Bouboui (NC_033693)
	clstr 86 - Dengue 4 (AF326573)	clstr 32, clstr 34, clstr 35,	Cacipacoré (KF917536)
	clstr 64 - Yellow fever (X03700)	clstr 37, clstr 40, clstr 46,	Edge Hill (NC_030289)
	clstr 88 - Ilhéus (AY632539)	clstr 47, clstr 51, clstr 52,	Gadgets Gully (NC_033723)
	clstr 33 - Japanese encephalitis (M18370)	clstr 54, clstr 55, clstr 57,	Jugra (NC_033699)
	clstr 58 - Kokobera (AY632541)	clstr 61, clstr 62, clstr 66,	Jutiapa (KJ469371)
	clstr 85 - Kyasanur Forest disease (NC_039218)	clstr 67, clstr 68, clstr 69,	Kadam (NC_033724)
	clstr 43 - Langat (AF253419)	clstr 72, clstr 73, clstr 82,	Kédougou (AY632540)
	clstr 45 - St. Louis encephalitis (DQ525916)	clstr 87, clstr 95, clstr 99,	Meaban (NC_033721)
	clstr 56 - Louping ill (Y07863)	clstr 102, clstr 104, clstr 106,	Modoc (NC_003635)
	clstr 25 - Murray Valley encephalitis (AF161266)	clstr 107, clstr 112, clstr 113,	Montana myotis leukoencephalitis (NC_004119)
	clstr 21 - West Nile (M12294)	clstr 121, clstr 128, clstr 145	Royal Farm (NC_039219)
	clstr 44 - Ntaya (JX236040)		Saumarez Reef (NC_033726)
	clstr 19 - Omsk hemorrhagic fever (AY193805)		Tyuleniy (NC_023424)
	clstr 70 - Powassan (L06436)		Uganda S (NC_033698)
	clstr 24 - Tembusu (JF895923)		Yokose (AB114858)
	clstr 15 - Usutu (AY453411)		
	clstr 75 - Wesselsbron (EU707555)		
	clstr 77 - Zika (AY632535)		
	clstr 13 - Zika (KY766069, KJ776791, KX377337)		
suma	clstr 20 - Bagaza (AY632545)	clstr 3, clstr 4, clstr 5,	clstr 114 - Saboya (NC_033697), Potiskum (NC_029054)
	clstr 0 - Dengue 2 (KF955373)	clstr 6, clstr 7, clstr 10,	20 singletons: Aroa (AY632536)
	clstr 8 - Dengue 1 (NC_001477)	clstr 11, clstr 13, clstr 15,	Banzi (NC_043110)
	clstr 9 - Dengue 3 (M93130)	clstr 16, clstr 17, clstr 21,	Bouboui (NC_033693)
	clstr 1 - Dengue 4 (AF326573)	clstr 23, clstr 27, clstr 32,	Cacipacoré (KF917536)
	clstr 14 - Yellow fever (X03700)	clstr 33, clstr 35, clstr 39,	Edge Hill (NC_030289)
	clstr 24 - Ilhéus (AY632539)	clstr 42, clstr 44, clstr 48,	Gadgets Gully (NC_033723)
	clstr 31 - Japanese encephalitis (M18370)	clstr 49, clstr 51, clstr 52,	Jugra (NC_033699)
	clstr 54 - Langat (AF253419)	clstr 55, clstr 56, clstr 57,	Jutiapa (KJ469371)
	clstr 47 - Louping ill (Y07863)	clstr 58, clstr 59, clstr 63,	Kadam (NC_033724)
	clstr 34 - Murray Valley encephalitis (AF161266)	clstr 70, clstr 71, clstr 76,	Kédougou (AY632540)
	clstr 2 - West Nile (M12294)	clstr 80, clstr 81, clstr 83,	Kokobera (AY632541)
	clstr 29 - Ntaya (JX236040)	clstr 85, clstr 86, clstr 88,	Kyasanur Forest disease (NC_039218)
	clstr 50 - Omsk hemorrhagic fever (AY193805)	clstr 89, clstr 92, clstr 93,	Meaban (NC_033721)
	clstr 41 - Powassan (L06436)	clstr 95, clstr 96, clstr 100,	Modoc (NC_003635)
	clstr 26 - Tembusu (JF895923)	clstr 118, clstr 120, clstr 128,	Montana myotis leukoencephalitis (NC_004119)
	clstr 18 - Usutu (AY453411)	clstr 139, clstr 142	Royal Farm (NC_039219)
	clstr 68 - Wesselsbron (EU707555)		Saumarez Reef (NC_033726)
	clstr 84 - Zika (AY632535)		Tyuleniy (NC_023424)
	clstr 12 - Zika (KY766069, KJ776791, KX377337)		Uganda S (NC_033698)
			Yokose (AB114858)
			sequence of St. Louis encephalitis (DQ525916) removed by algorithm
vse	clstr 29 - Bagaza (AY632545)	clstr 5, clstr 11, clstr 14,	clstr 137 - Saboya (NC_033697), Potiskum (NC_029054)
	clstr 31 - Dengue 2 (KF955373)	clstr 16, clstr 17, clstr 20,	18 singletons: Aroa (AY632536)

Continuation of Tab. S4

	clstr 12 - Dengue 1 (NC_001477)	clstr 22, clstr 23, clstr 26,	Banzi (NC_043110)
	clstr 76 - Dengue 3 (M93130)	clstr 27, clstr 28, clstr 32,	Bouboui (NC_033693)
	clstr 111 - Dengue 4 (AF326573)	clstr 34, clstr 35, clstr 37,	Cacipacoré (KF917536)
	clstr 64 - Yellow fever (X03700)	clstr 40, clstr 46, clstr 47,	Edge Hill (NC_030289)
	clstr 87 - Ilhéus (AY632539)	clstr 51, clstr 52, clstr 54,	Gadgets Gully (NC_033723)
	clstr 33 - Japanese encephalitis (M18370)	clstr 55, clstr 57, clstr 61,	Jugra (NC_033699)
	clstr 58 - Kokobera (AY632541)	clstr 62, clstr 66, clstr 67,	Jutiapa (KJ469371)
	clstr 84 - Kyasanur Forest disease (NC_039218)	clstr 68, clstr 69, clstr 72,	Kadam (NC_033724)
	clstr 43 - Langat (AF253419)	clstr 73, clstr 80, clstr 82,	Kédougou (AY632540)
	clstr 45 - St. Louis encephalitis (DQ525916)	clstr 85, clstr 86, clstr 97,	Meaban (NC_033721)
	clstr 56 - Louping ill (Y07863)	clstr 99, clstr 103, clstr 104,	Modoc (NC_003635)
	clstr 25 - Murray Valley encephalitis (AF161266)	clstr 105, clstr 110, clstr 113,	Montana myotis leukoencephalitis (NC_004119)
	clstr 21 - West Nile (M12294)	clstr 117, clstr 121, clstr 125,	Royal Farm (NC_039219)
	clstr 44 - Ntaya (JX236040)	clstr 141	Saumarez Reef (NC_033726)
	clstr 19 - Omsk hemorrhagic fever (AY193805)		Tyuleniy (NC_023424)
	clstr 70 - Powassan (L06436)		Uganda S (NC_033698)
	clstr 24 - Tembusu (JF895923)		Yokose (AB114858)
	clstr 15 - Usutu (AY453411)		
	clstr 75 - Wesselsbron (EU707555)		
	clstr 78 - Zika (AY632535)		
	clstr 13 - Zika (KY766069, KJ776791, KX377337)		
mmseq	clstr 11 - Bagaza (AY632545)	clstr 1, clstr 4, clstr 5,	clstr 40 - Saboya (NC_033697), Potiskum (NC_029054)
	clstr 121 - Dengue 2 (KF955373)	clstr 16, clstr 22, clstr 30,	18 singletons: Aroa (AY632536)
	clstr 51 - Dengue 1 (NC_001477)	clstr 34, clstr 37, clstr 41,	Banzi (NC_043110)
	clstr 101 - Dengue 3 (M93130)	clstr 42, clstr 43, clstr 44,	Bouboui (NC_033693)
	clstr 12 - Dengue 4 (AF326573)	clstr 45, clstr 46, clstr 49,	Cacipacoré (KF917536)
	clstr 118 - Yellow fever (X03700)	clstr 52, clstr 53, clstr 54,	Edge Hill (NC_030289)
	clstr 110 - Ilhéus (AY632539)	clstr 58, clstr 62, clstr 63,	Gadgets Gully (NC_033723)
	clstr 108 - Japanese encephalitis (M18370)	clstr 71, clstr 84, clstr 86,	Jugra (NC_033699)
	clstr 83 - Kokobera (AY632541)	clstr 88, clstr 92, clstr 95,	Jutiapa (KJ469371)
	clstr 50 - Kyasanur Forest disease (NC_039218)	clstr 98, clstr 103, clstr 104,	Kadam (NC_033724)
	clstr 120 - Langat (AF253419)	clstr 107, clstr 109, clstr 112,	Kédougou (AY632540)
	clstr 128 - St. Louis encephalitis (DQ525916)	clstr 124, clstr 126, clstr 127,	Meaban (NC_033721)
	clstr 119 - Louping ill (Y07863)	clstr 132, clstr 133, clstr 135	Modoc (NC_003635)
	clstr 137 - Murray Valley encephalitis (AF161266)		Montana myotis leukoencephalitis (NC_004119)
	clstr 13 - West Nile (M12294)		Royal Farm (NC_039219)
	clstr 91 - Ntaya (JX236040)		Saumarez Reef (NC_033726)
	clstr 10 - Omsk hemorrhagic fever (AY193805)		Tyuleniy (NC_023424)
	clstr 116 - Powassan (L06436)		Uganda S (NC_033698)
	clstr 73 - Tembusu (JF895923)		Yokose (AB114858)
	clstr 31 - Usutu (AY453411)		
	clstr 117 - Wesselsbron (EU707555)		
	clstr 139 - Zika (AY632535)		
	clstr 20 - Zika (KY766069, KJ776791, KX377337)		
hdb	clstr 77 - Bagaza (AY632545)	clstr 0, clstr 1, clstr 3, clstr 4,	clstr 59 - Modoc (NC_003635), Saboya (NC_033697),
	clstr 71 - Dengue 2 (KF955373)	clstr 5, clstr 6, clstr 7, clstr 10,	Uganda S (NC_033698), Wesselsbron (EU707555)
	clstr 53 - Dengue 1 (NC_001477)	clstr 11, clstr 12, clstr 13, clstr 14,	23 singletons: Aroa (AY632536)
	clstr 23 - Dengue 3 (M93130)	clstr 15, clstr 16, clstr 17, clstr 18,	Banzi (NC_043110)
	clstr 20 - Dengue 4 (AF326573)	clstr 19, clstr 21, clstr 22, clstr 24,	Bouboui (NC_033693)

Continuation of Tab. S4		
clstr 45 - Yellow fever (X03700)	clstr 25, clstr 26, clstr 27, clstr 28,	Cacipacoré (KF917536)
clstr 83 - Kyasanur Forest disease (NC_039218)	clstr 30, clstr 31, clstr 32, clstr 33,	Edge Hill (NC_030289)
clstr 78 - St. Louis encephalitis (DQ525916)	clstr 34, clstr 35, clstr 36, clstr 37,	Gadgets Gully (NC_033723)
clstr 61 - Murray Valley encephalitis (AF161266)	clstr 38, clstr 39, clstr 40, clstr 41,	Ilhéus (AY632539)
clstr 43 - West Nile (M12294)	clstr 42, clstr 44, clstr 46, clstr 47,	Japanese encephalitis (M18370)
clstr 85 - Omsk hemorrhagic fever (AY193805)	clstr 48, clstr 49, clstr 50, clstr 51,	Jugra (NC_033699)
clstr 29 - Powassan (L06436)	clstr 52, clstr 54, clstr 55, clstr 56,	Jutiapa (KJ469371)
clstr 76 - Tembusu (JF895923)	clstr 57, clstr 58, clstr 60, clstr 62,	Kadam(NC_033724)
clstr 9 - Usutu (AY453411)	clstr 63, clstr 64, clstr 65, clstr 66,	Kédougou (AY632540)
clstr 8 - Zika (AY632535)	clstr 67, clstr 68, clstr 69, clstr 70,	Kokobera (AY632541)
clstr 2 - Zika (KY766069, KJ776791, KX377337)	clstr 72, clstr 73, clstr 74, clstr 75,	Langat (AF253419)
	clstr 79, clstr 80, clstr 81, clstr 82,	Louping ill (Y07863)
	clstr 84, clstr 86, clstr 87, clstr 88,	Meaban (NC_033721)
	clstr 89, clstr 90, clstr 91, clstr 92,	Montana myotis leukoencephalitis (NC_004119)
	clstr 93, clstr 94, clstr 95, clstr 96,	Ntaya (JX236040)
	clstr 97, clstr 98, clstr 99, clstr 100	Royal Farm (NC_039219)
	Potiskum (NC_029054)	
	Saumarez Reef (NC_033726)	
	Tyuleniy (NC_023424)	
	Yokose (AB114858)	

End of Table

Table S5. *Orthoflavivirus* species, their ICTV representatives & NCBI RefSeqs and the cluster number in which they were clustered. GenBank – GenBank accession ID; NCBI RefSeq – NCBI RefSeq accession ID; ICTV – category in the ICTV (E – exemplar genome, A – additional genome); Nucl Comp – Nucleotide completeness (C – Complete, CC – Coding-complete P – Partial); dataset – presence of the genome in our *Orthoflavivirus* dataset (G – GenBank in our dataset, N – NCBI RefSeq in our dataset); cd – `CD-HIT-EST`; sum – `SUMACLUST`; vse – `VSEARCH`; mmseq – `MMSeqs2`; hdb – `HDBSCAN`;

Begin of Table

Virus Name	Abbr	Accession ID		ICTV	Nucl Comp	dataset	Cluster Nr.				
		GenBank	NCBI RefSeq				cd	suma	vse	mmseq	hdb
Apoi virus	APOIV	AF160193	NC_003676	E	C						
Aroa virus	AROAV	AY632536	NC_009026	E	C	G	74	69	74	61	-1
Aroa virus	AROAV	AF013362		A	P						
Bussuquara virus	BSQV	AF013366		A	P						
Iguape virus	IGUV	AF013375		A	P						
Naranjal virus	NJLV	AF013390		A	P						
Bagaza virus	BAGV	AY632545	NC_012534	E	C	G	29	20	29	11	77
Banzi virus	BANV	DQ859056	NC_043110	E	C	N	140	113	136	74	-1
Bouboui virus	BOUV	DQ859057	NC_033693	E	C	N	142	112	138	7	-1
Rio Bravo virus	RBV	AF144692	NC_003675	E	C						
Bukalasa bat virus	BBV	AF013365	NC_043111	E	P						
Cacipacoré virus	CPCV	KF917536	NC_026623	E	C	G	131	117	126	76	-1
Carey Island virus	CIV	AF013368	NC_043112	E	P						
Cowbone Ridge virus	CRV	AF013370	NC_043113	E	P						
Dakar bat virus	DBV	AF013371	NC_043114	E	P						
Dengue virus type 2	DENV-2	U87411 (KF955373)	NC_001474	E	C	*	30	0	31	121	71
Dengue virus type 1	DENV-1	U88536	NC_001477	A	C	N	12	8	12	51	53
Dengue virus type 3	DENV-3	M93130		A	C	G	76	9	76	101	23
Dengue virus type 4	DENV-4	AF326573		A	C	G	86	1	111	12	20
Edge Hill virus	EHV	DQ859060	NC_030289	E	C	N	139	102	134	111	-1
Tick-borne encephalitis virus - European	TBEV-Eur	U27495	NC_001672	E	C						
Tick-borne encephalitis virus - Far Eastern	TBEV-FE	X07755		A	P						
Tick-borne encephalitis virus - Siberian	TBEV-Sib	L40361		A	C	2024					

Continuation of Tab. S5

Entebbe bat virus	ENTV	DQ837641	NC_008718	E	C						
Sokuluk virus	SOKV	AF013405		A	P						
Yellow fever virus	YFV	X03700	NC_002031	E	C	G	64	14	64	118	45
Gadgets Gully virus	GGYV	DQ235145	NC_033723	E	C	N	135	99	130	21	-1
Ilhéus virus	ILHV	AY632539	NC_009028	E	C	G	88	24	87	110	-1
Rocio virus	ROCV	AF013397		A	P						
Israel turkey meningoencephalomyelitis virus	ITV	AF013377	NC_043115	E	P						
Japanese encephalitis virus	JEV	M18370	NC_001437	E	C	G	33	31	33	108	-1
Jugra virus	JUGV	DQ859066	NC_033699	E	C	N	144	109	139	125	-1
Jutiapa virus	JUTV	KJ469371	NC_026620	E	C	G	146	105	142	105	-1
Kadam virus	KADV	DQ235146	NC_033724	E	C	N	138	101	133	60	-1
Kédougou virus	KEDV	AY632540	NC_012533	E	C	G	101	22	101	35	-1
Kokobera virus	KOKV	AY632541	NC_009029	E	C	G	58	37	58	83	-1
Stratford virus	STRV	AF013407		A	P						
Koutango virus	KOUV	AF013384	NC_043116	E	P						
Kyasanur Forest disease virus	KFDV	AY323490	NC_039218	E	C	N	85	106	84	50	83
Alkhumra hemorrhagic fever virus	AHFV	AF331718		A	C						
Langat virus	LGTV	AF253419	NC_003690	E	C	G	43	54	43	120	-1
St. Louis encephalitis virus	SLEV	DQ525916	NC_007580	E	C	G	45		45	128	78
Louping ill virus	LIV	Y07863	NC_001809	E	C	G	56	47	56	119	-1
Louping ill virus - British	GGEV	D12937		A	P						
Louping ill virus - Irish	LIV-Brit	X86784		A	P						
Louping ill virus - Spanish	LIV-Ir	DQ235152		A	CC						
Greek goat encephalitits virus	LIV-Spain	DQ235153		A	CC						
Turkish sheep encephalitis virus	TSEV	DQ235151		A	CC						
Meaban virus	MEAV	DQ235144	NC_033721	E	C	N	133	103	128	6	-1
Modoc virus	MODV	AJ242984	NC_003635	E	C	N	120	73	116	66	59
Montana myotis leukoencephalitis virus	MMLV	AJ299445	NC_004119	E	C	N	108	74	106	32	-1
Murray Valley encephalitis virus	MVEV	AF161266	NC_000943	E	C	G	25	34	25	137	61
Alfuy virus	ALFV	AF013360		A	P						
West Nile virus	WNV	M12294	NC_001563	E	C	G	21	2	21	13	43
Kunjin virus	KUNV	D00246		A	C						
Ntaya virus	NTAV	JX236040	NC_018705	E	C	G	44	29	44	91	-1
Omsk hemorrhagic fever virus	OHFV	AY193805	NC_005062	E	C	G	19	50	19	10	85
San Perlita virus	SPV	AF013402	NC_043118	E	P						
Phnom Penh bat virus	PPBV	AF013394	NC_074777	E	P						
Batu Cave virus	BCV	AF013369		A	P						
Powassan virus	POWV	L06436	NC_003687	E	C	G	70	41	70	116	29
Deer tick virus	DTV	AF311056		A	CC						
Royal Farm virus	RFV	DQ235149	NC_039219	E	C	N	134	108	129	122	-1
Saboya virus	SABV	DQ859062	NC_033697	E	C	N	143	114	137	40	59
Potiskum virus	POTV	DQ859067	NC_029054	A	CC	N	143	114	137	40	-1
Saumarez Reef virus	SREV	DQ235150	NC_033726	E	C	N	132	104	127	15	-1
Sepik virus	SEPV	DQ837642	NC_008719	E	C						
Tembusu virus	TMUV	JF895923	NC_015843	E	C	G	24	26	24	73	76
Tyuleniy virus	TYUV	KF815939	NC_023424	E	C	N	111	140	109	130	-1
Uganda S virus	UGSV	DQ859065	NC_033698	E	C	N	141	111	135	64	59
Usutu virus	USUV	AY453411	NC_006551	E	C	G	15	18	15	31	9
Sal Vieja virus	SVV	AF013401	NC_043117	E	P						
Wesselsbron virus	WESSV	EU707555	NC_012735	E	C	G	75	68	75	117	59
Yaoundé virus	YAOV	AF013413	NC_074778	E	P						
Yokose virus	YOKV	AB114858	NC_005039	E	C	G	65	61	65	113	-1
Zika virus	ZIKV	AY632535	NC_012532	E	C	G	77	84	78	139	8
Zika virus	ZIKV	KY766069		A	C	G	13	12	13	20	2
Zika virus	ZIKV	KJ776791		A	C	G	13	12	13	20	2
Zika virus	ZIKV	KX377337		A	C	G	13	12	13	20	2

End of Table

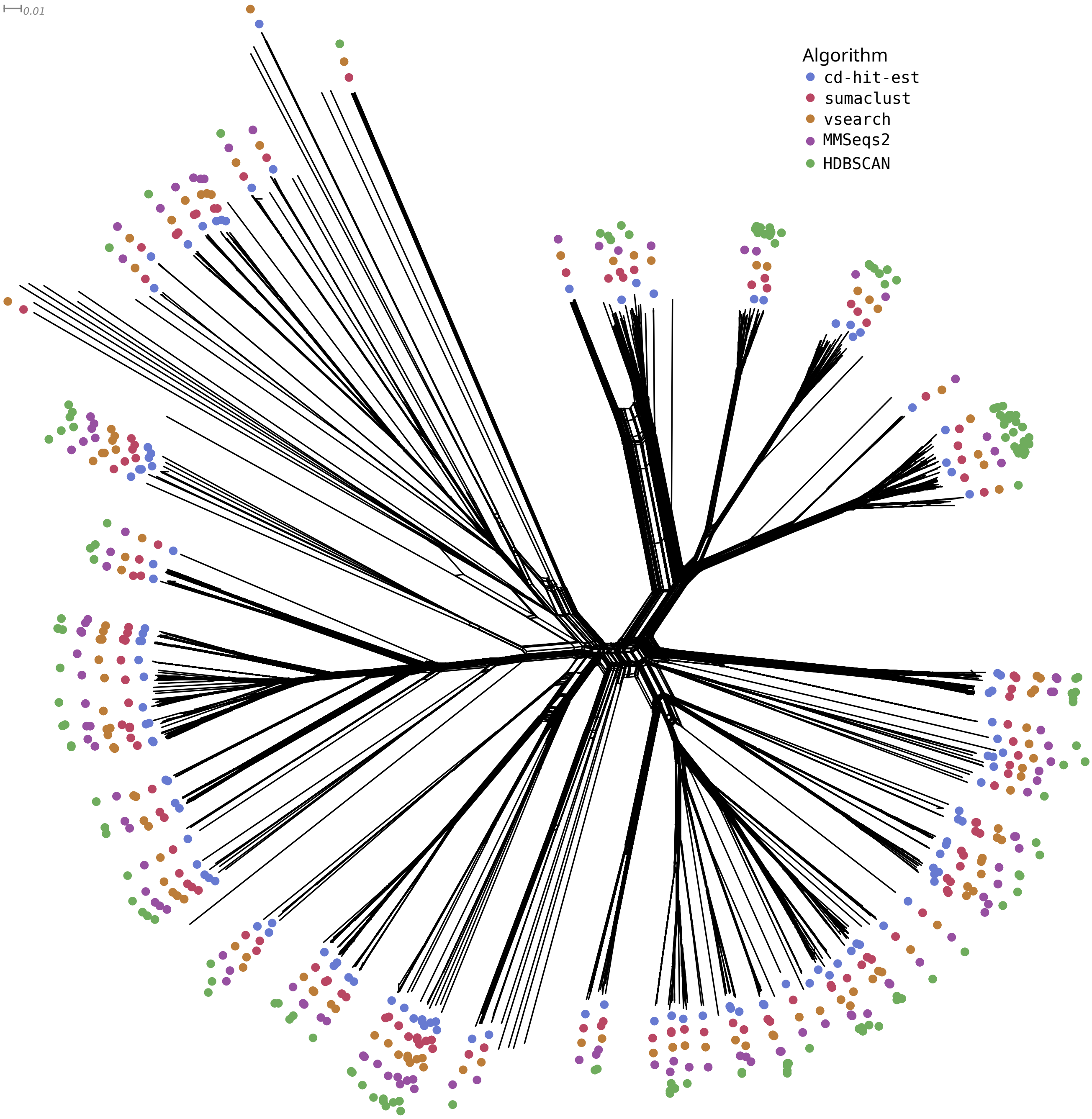

Figure S5. Split graph of *Orthoflavivirus* with highlighted cluster representatives of CD-HIT-EST, SUMACLUST, VSEARCH, MMSeqs2, and HDBSCAN. The split graph is reconstructed using SplitsTree based on the MAFFT alignment of the complete genome sequences (7,681 genomes) resulting from the first filtering steps of ViralClust.

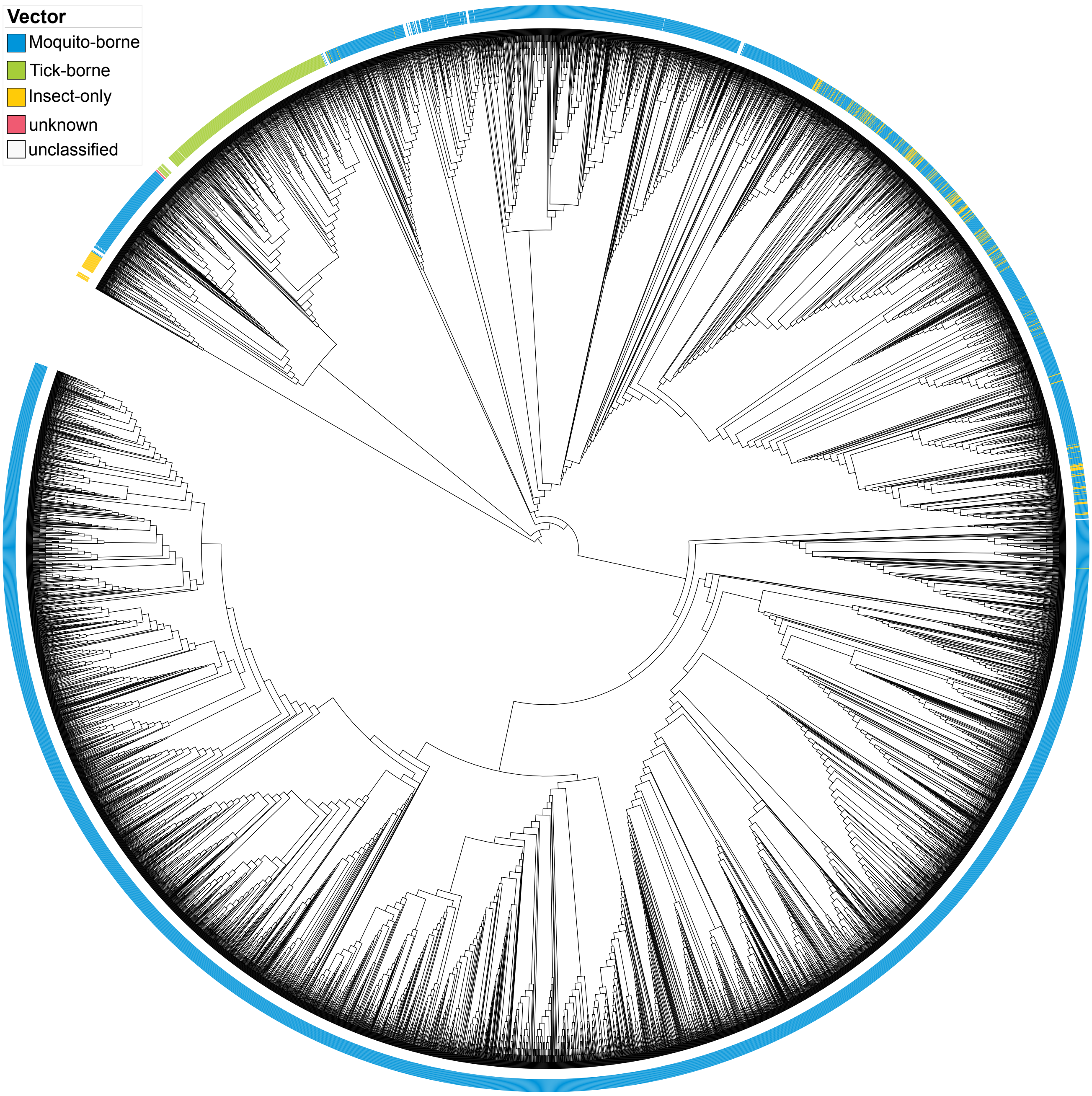

Figure S6. Phylogenetic representation of *Orthoflavivirus* showing vectors (Mosquito-borne, Tick-borne, Insect-only, and unknown). The tree is reconstructed using `FastTree` based on the `MAFFT` alignment of the complete genome sequences (7,681 genomes) resulting from the first filtering steps of `ViralClust`. More details in Fig. 5.

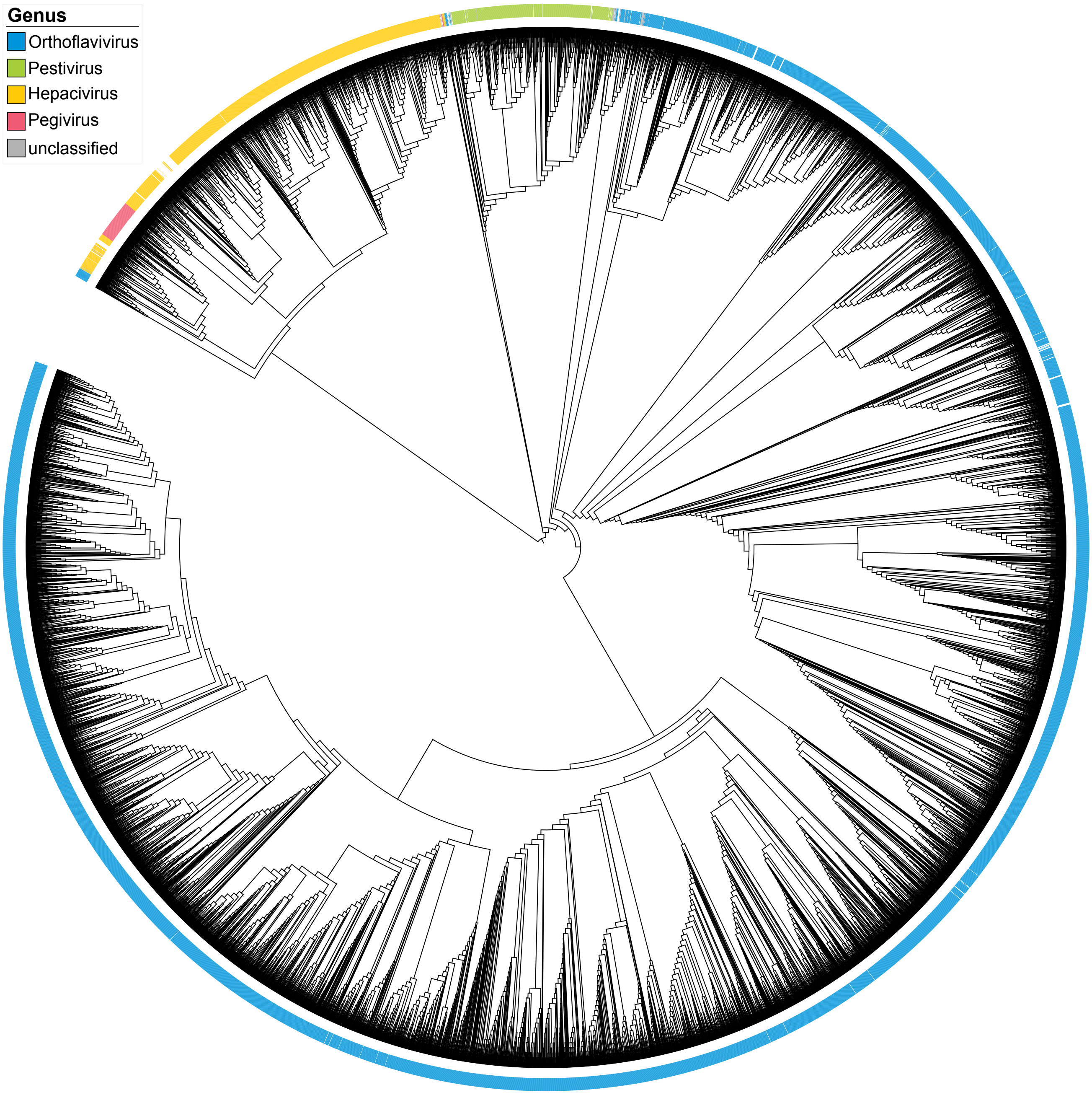

Figure S7. Phylogenetic representation of *Flaviviridae* with labeled genera: *Orthoflavivirus*, *Pestivirus*, *Hepacivirus*, and *Pegivirus*. The tree is reconstructed using `FastTree` based on the `MAFFT` alignment of the complete genome sequences (9,478 genomes) resulting from the first filtering steps of `ViralClust`. More details in Fig. 6.

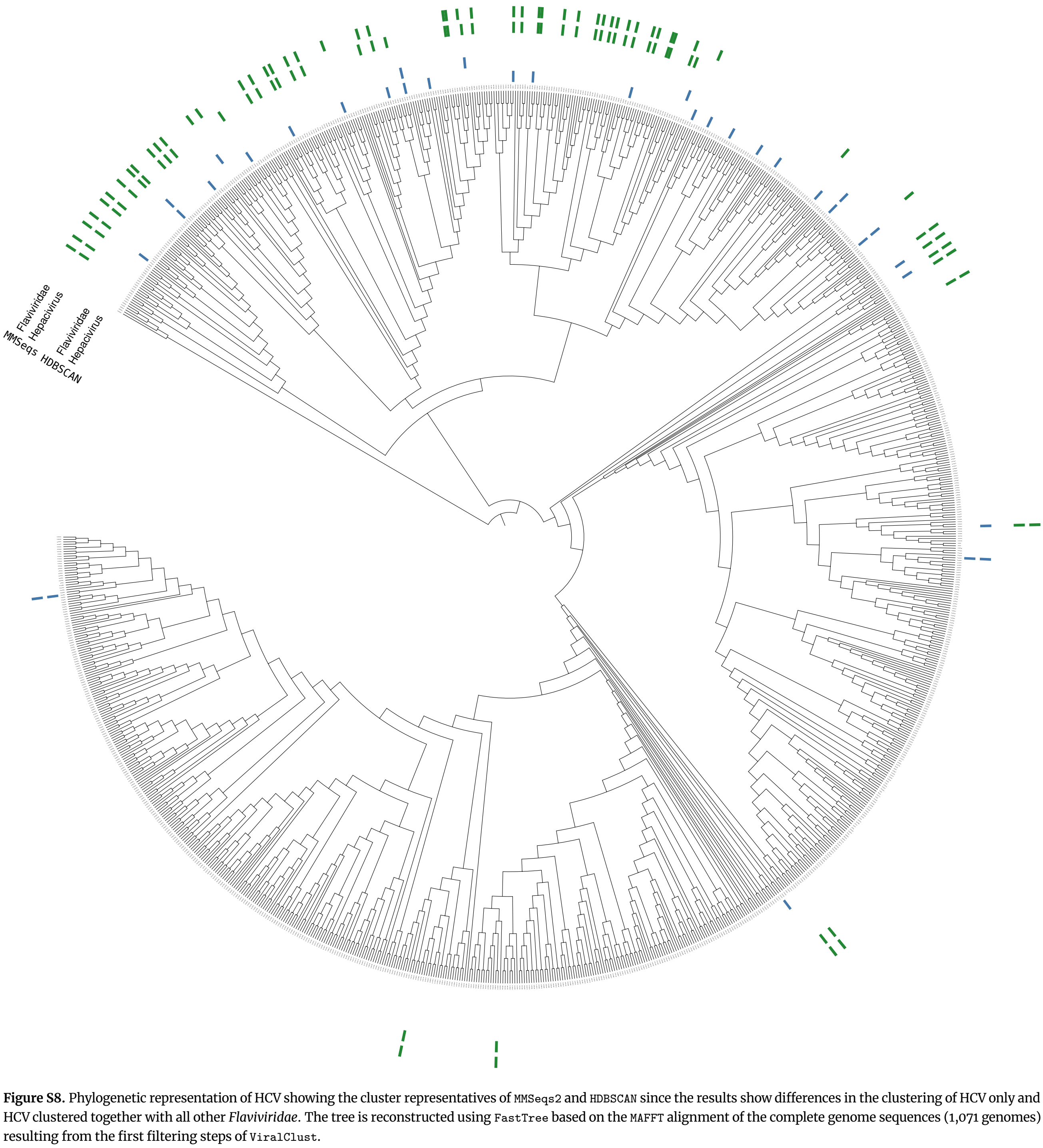

Figure S8. Phylogenetic representation of HCV showing the cluster representatives of `MMSeqs2` and `HDBSCAN` since the results show differences in the clustering of HCV only and HCV clustered together with all other *Flaviviridae*. The tree is reconstructed using `FastTree` based on the `MAFFT` alignment of the complete genome sequences (1,071 genomes) resulting from the first filtering steps of `ViralClust`.

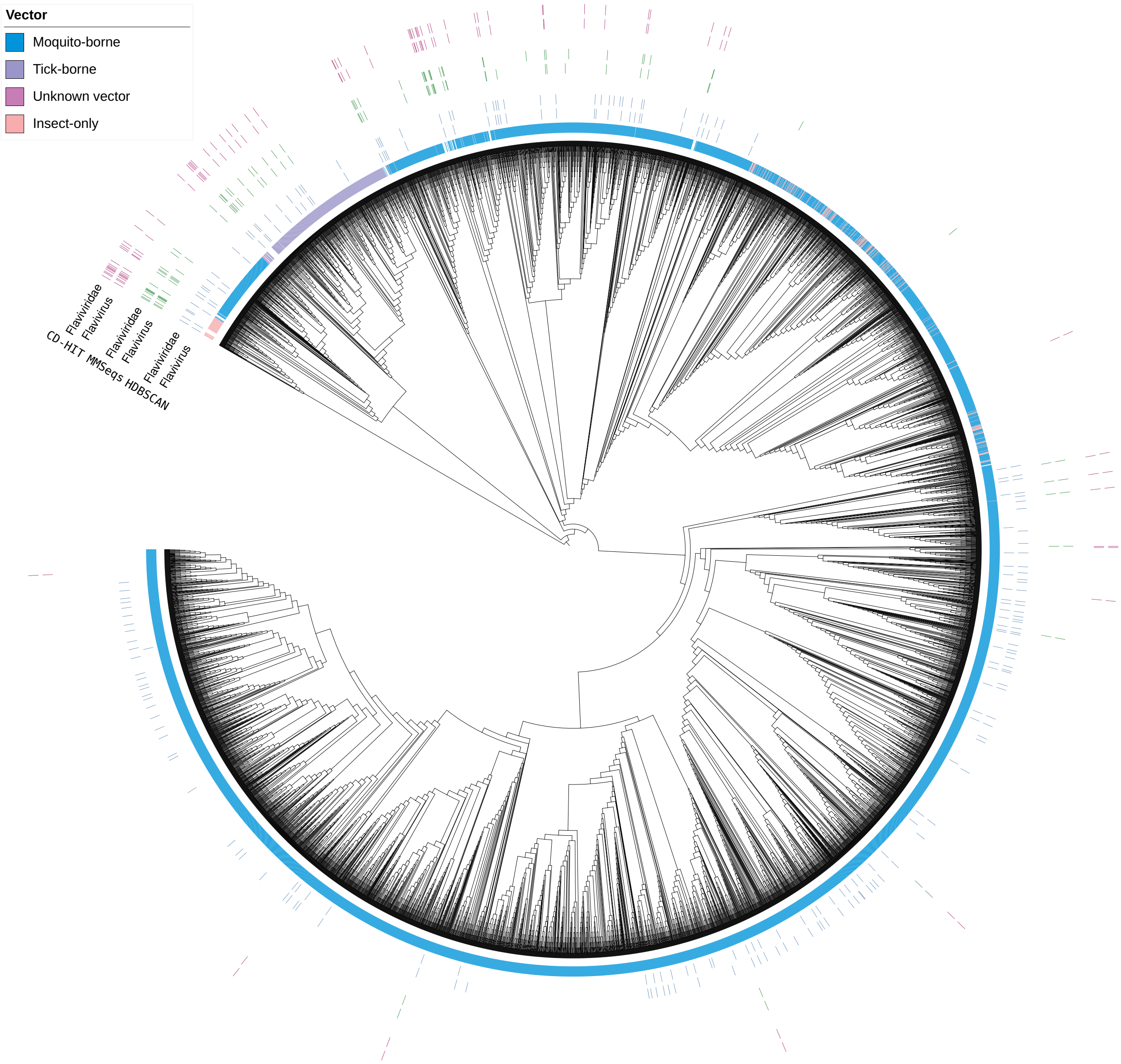

Figure S9. Phylogenetic representation of *Orthoflavivirus* showing the cluster representatives and labeled vectors (Mosquito-borne, Tick-borne, Unknown vector, and Insect-only). The clustering of the *Orthoflavivirus* only and the *Orthoflavivirus* among the entire *Flaviviridae* dataset showed differences in the results of `CD-HIT-EST`, `MMSeqs2`, and `HDBSCAN`. The tree is reconstructed using `FastTree` based on the `MAFFT` alignment of the complete genome sequences (7,681 genomes) resulting from the first filtering steps of `ViralClust`.

Alphainfluenzavirus influenzae

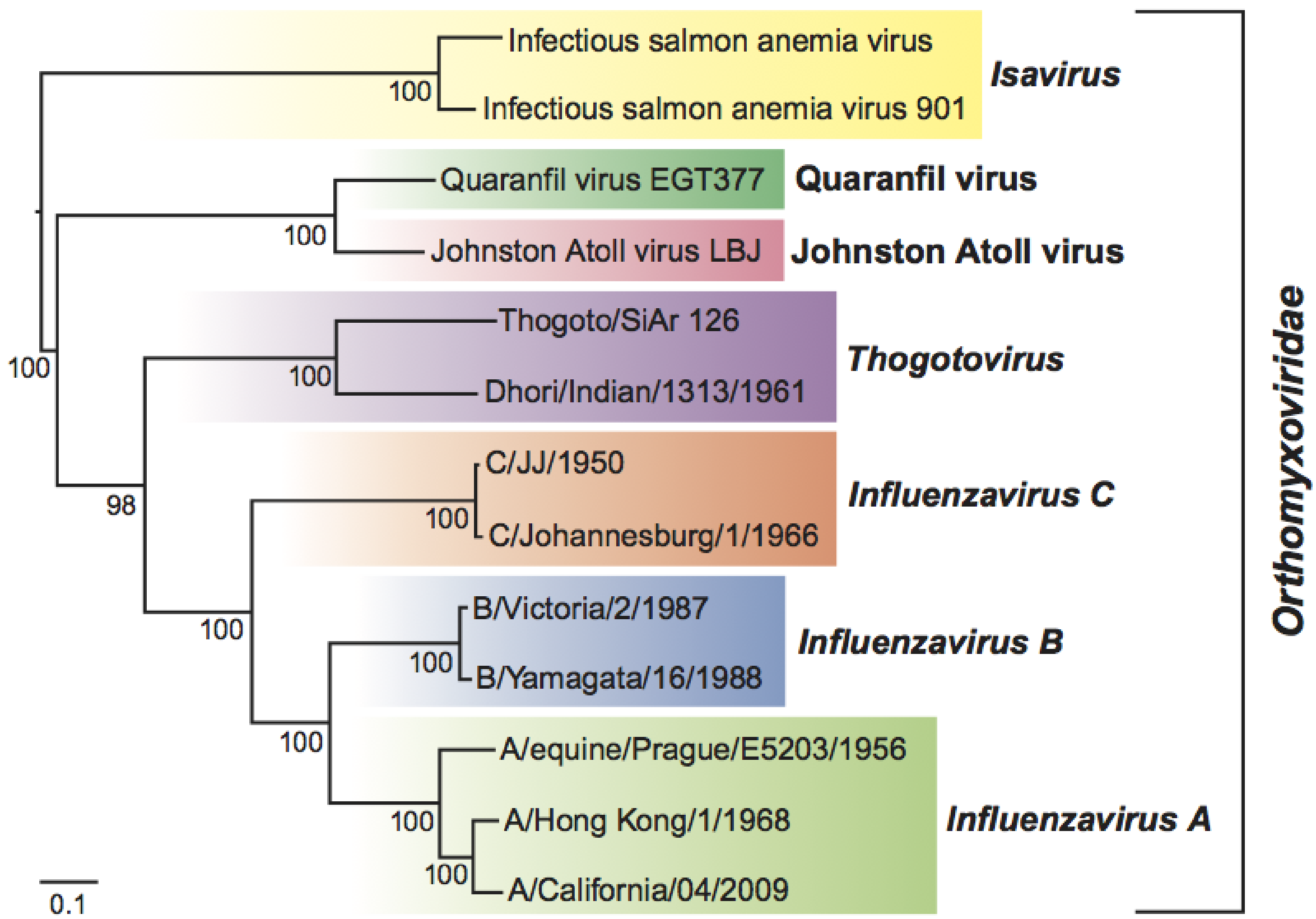

Figure S10. Phylogenetic tree of *Orthomyxoviridae* (ICTV) including IAV.

Table S6. Clustering IAV with and without (truncated) reverse-complement sequences. Dataset - dataset used as input; rc rep rem – reverse-complement representatives were removed from the representative dataset and used as new results; rc rem – reverse-complement sequences were removed from the non-redundant dataset and used as input for a new clustering run; #Seq – number of input genomes for clustering with `ViralClust`; Number of genomes after clustering – only considering clusters with a size greater than one; cd – `CD-HIT-EST`; sum – `SUMACLUST`; vse – `VSEARCH`; mmseq – `MMSeqs2`; hdb – `HDBSCAN`.

Segment	Dataset (#Seq)	Number of genomes after clustering				
		cd	suma	vse	mmseq	hdb
4 (H)	nr (108,514)	300	1,237	464	1,574	2,307
	rc rep rem	282	672	289	757	1,806
	rc rem (87,653)	933	677	300	728	1,767
6 (N)	nr (73,786)	228	773	329	640	1,622
	rc rep rem	217	430	231	385	1,386
	rc rem (65,393)	786	444	233	453	1,357

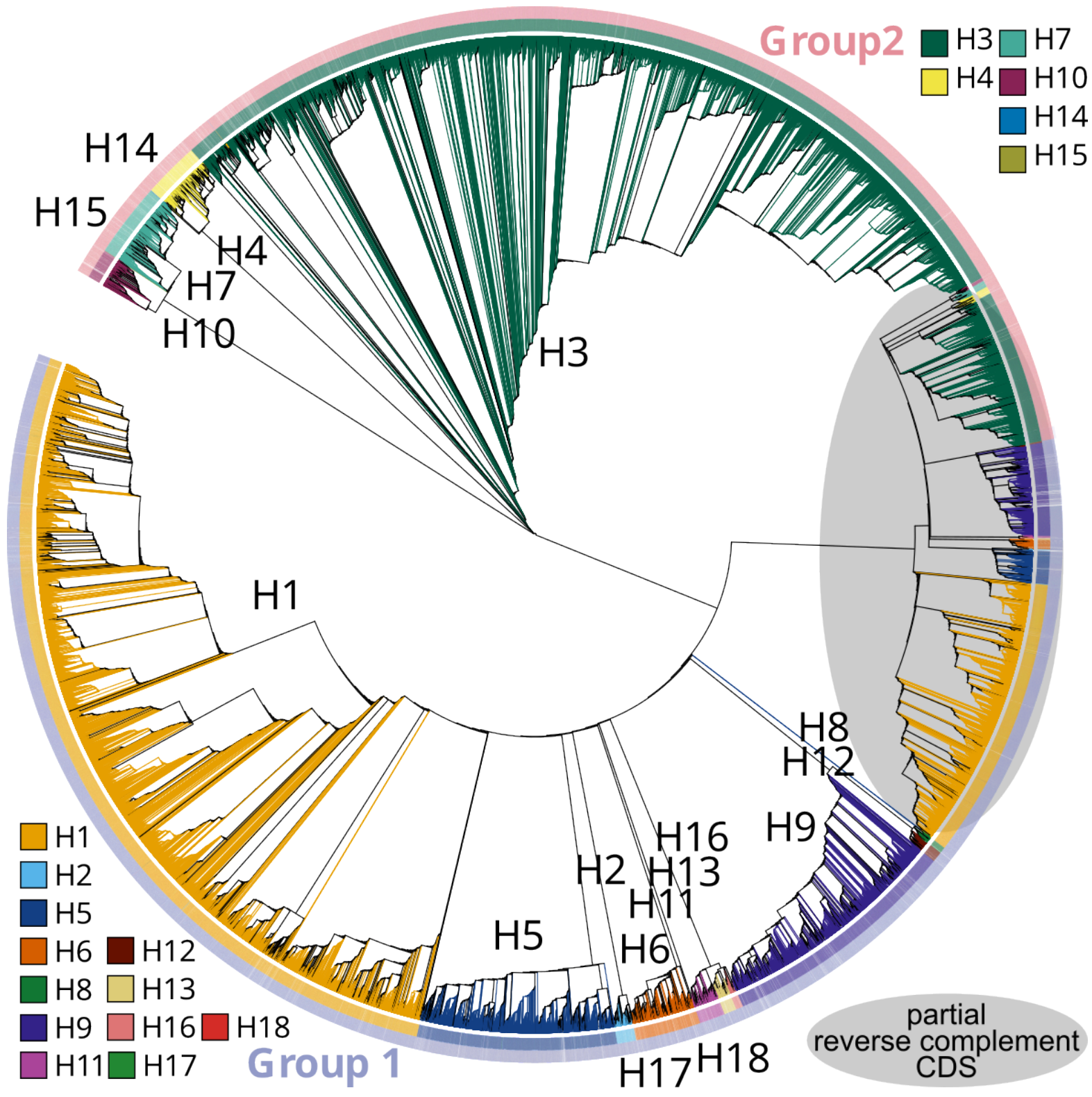

Figure S11. Phylogenetic tree of IAV segment 4 (H) with highlighted subtypes H1–H18. Gray area marks genomes with partial reverse complement CDS. That subtree 'mirrors' the rest of the tree. The tree is reconstructed using `FastTree` based on the `MAFFT` alignment of the complete genome sequences (108,514 genomes) resulting from the first filtering steps of `ViralClust`.

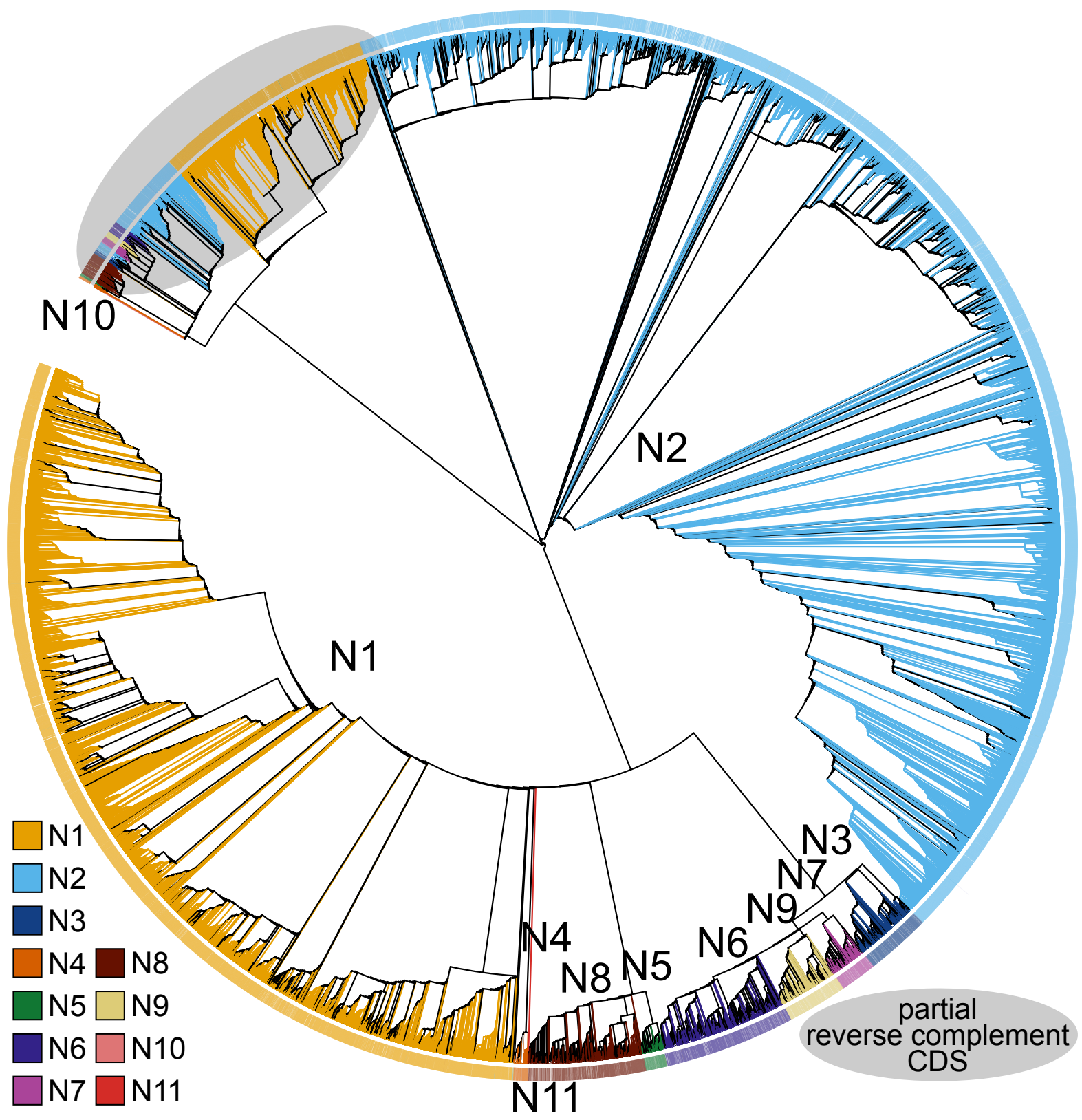

Figure S12. Phylogenetic tree of IAV segment 6 (N) with highlighted subtypes N1–N11. Gray area marks genomes with partial reverse complement CDS. That subtree 'mirrors' the rest of the tree. The tree is reconstructed using `FastTree` based on the `MAFFT` alignment of the complete genome sequences (73,786 genomes) resulting from the first filtering steps of `ViralClust`.

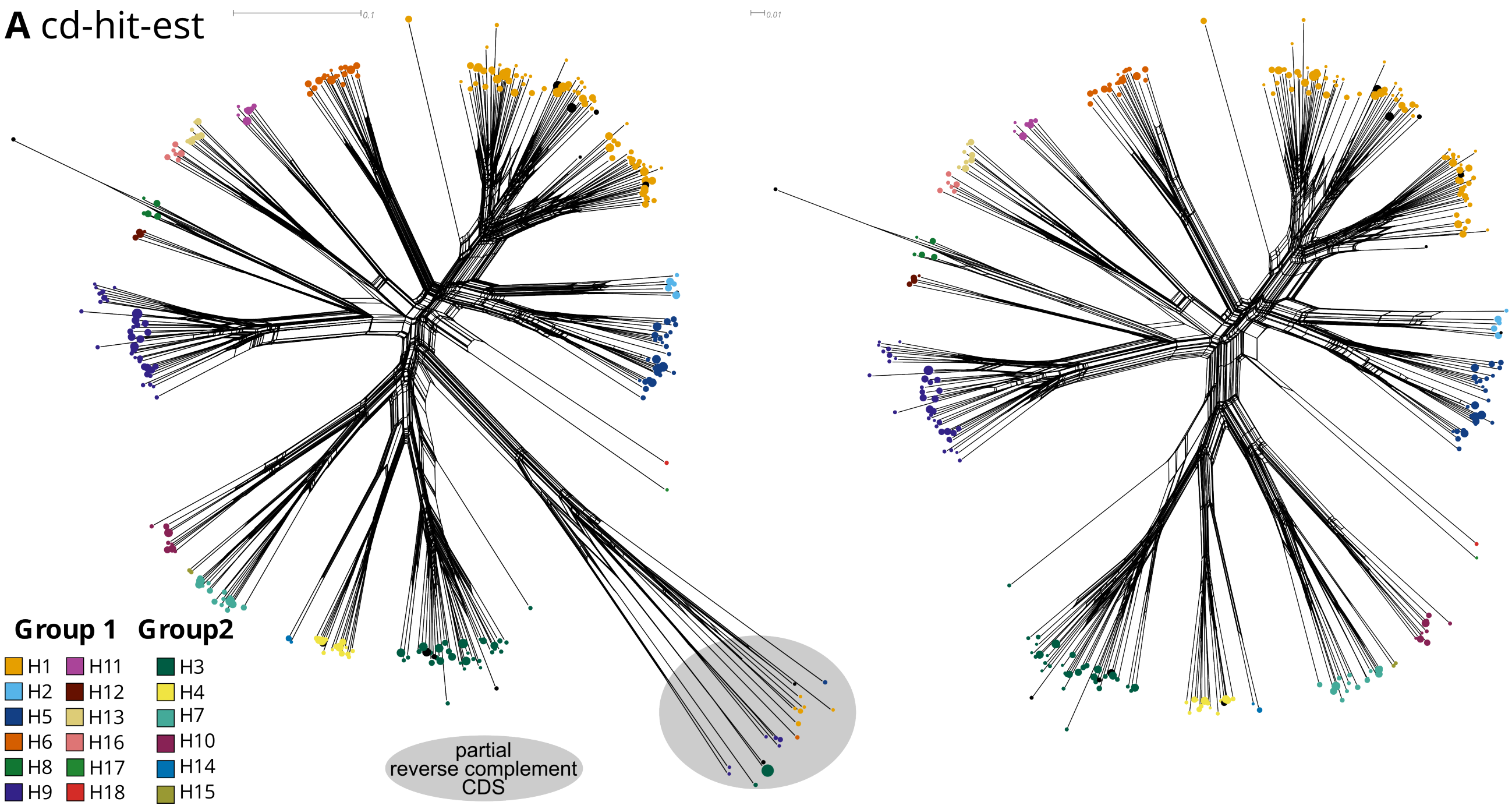

Figure S13. Split graphs of IAV segment 4 (H) representatives of (A) CD-HIT-EST. Left – all representatives (including partial CDS); Right – only complete genomes. The node size indicates the cluster size. Subtypes (H1–H18) are color-coded. The split graph is reconstructed using SplitsTree based on the MAFFT alignment of the complete genome sequences (108,514 genomes) resulting from the first filtering steps of ViralClust.

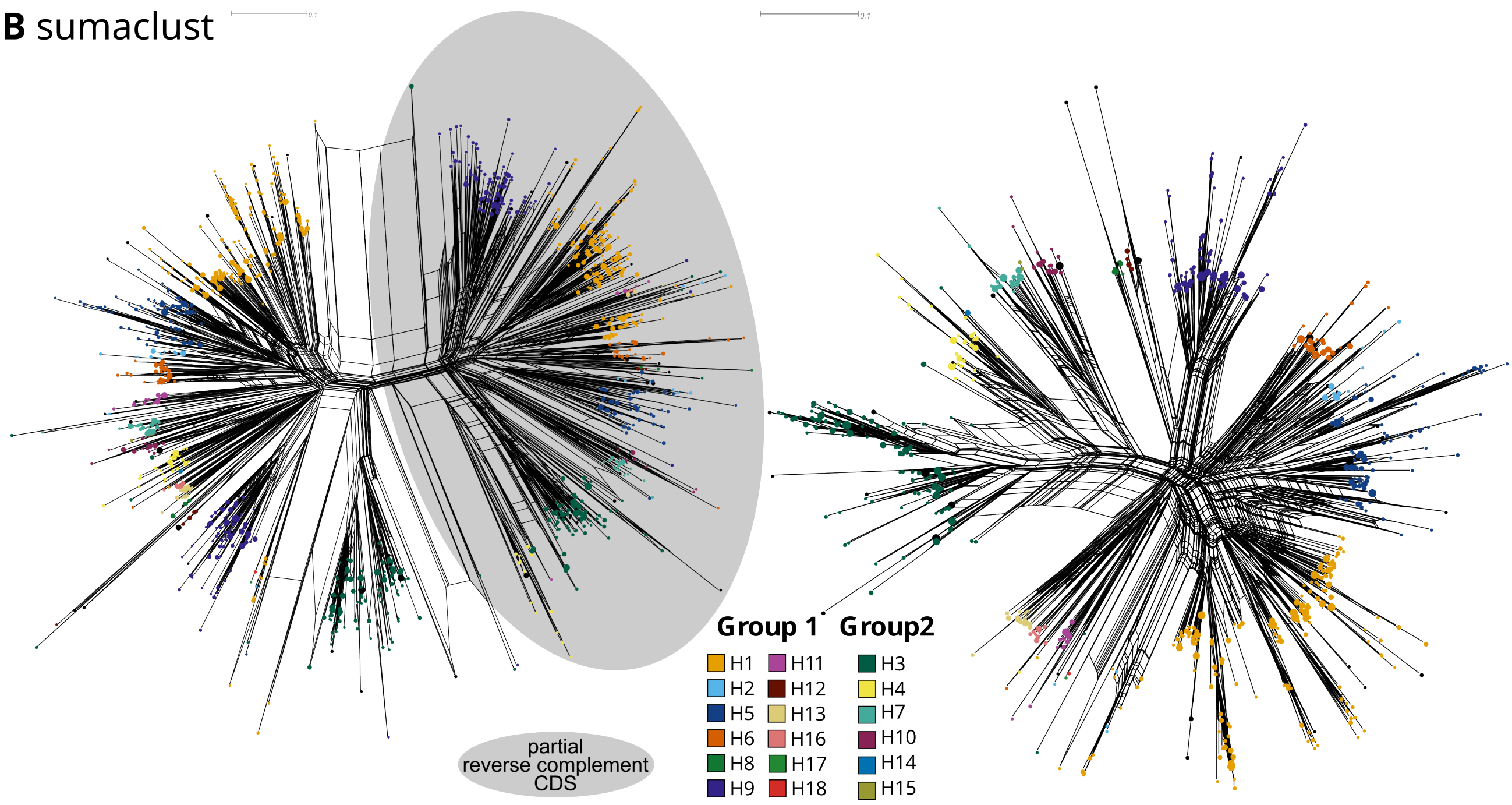

Figure S14. Split graphs of IAV segment 4 (H) representatives of (B) SUMACLUST. Left – all representatives (including partial CDS); Right – only complete genomes. The node size indicates the cluster size. Subtypes (H1–H18) are color-coded. The split graph is reconstructed using SplitsTree based on the MAFFT alignment of the complete genome sequences (108,514 genomes) resulting from the first filtering steps of ViralClust.

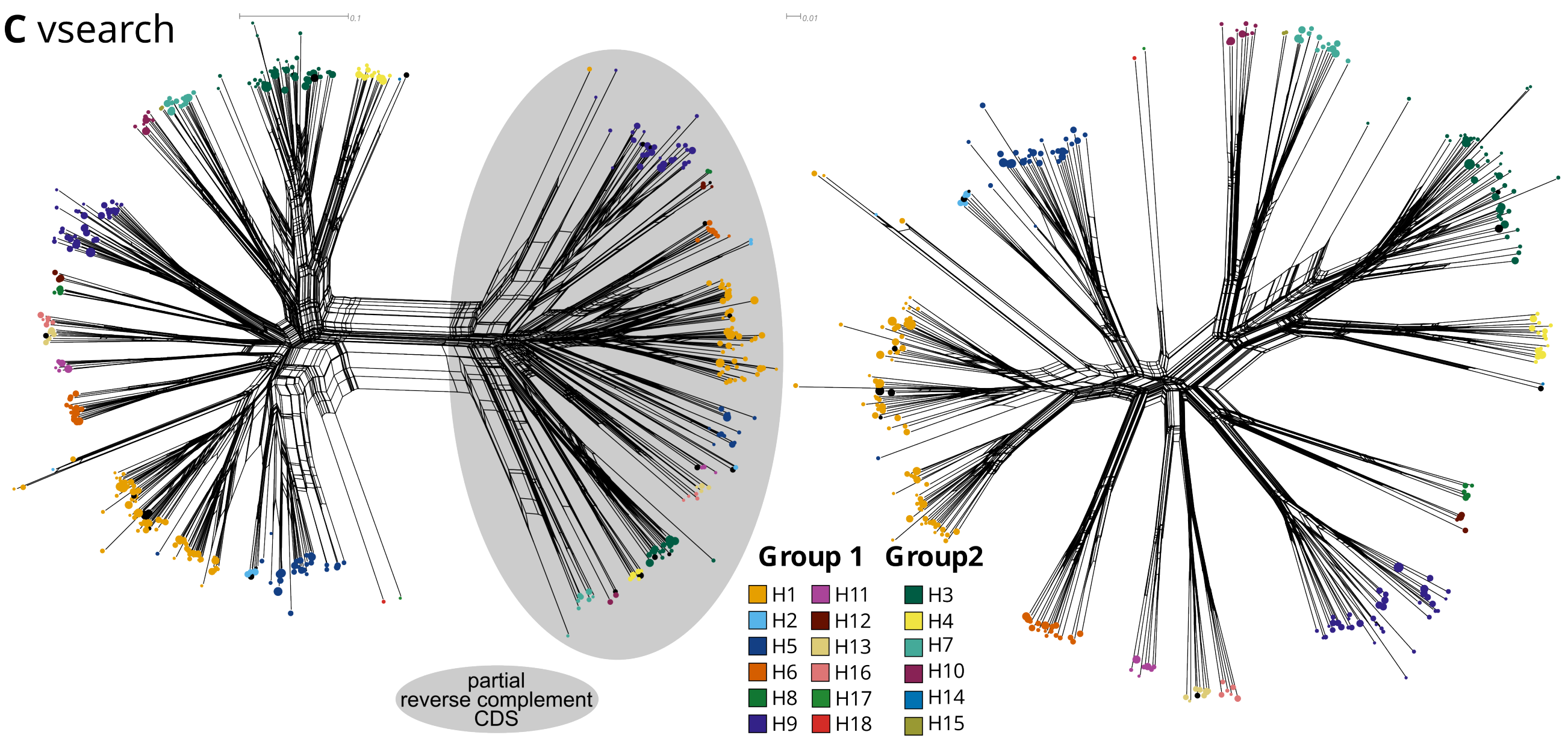

Figure S15. Split graphs of IAV segment 4 (H) representatives of (C) VSEARCH. Left – all representatives (including partial CDS); Right – only complete genomes. The node size indicates the cluster size. Subtypes (H1–H18) are color-coded. The split graph is reconstructed using SplitsTree based on the MAFFT alignment of the complete genome sequences (108,514 genomes) resulting from the first filtering steps of ViralClust.

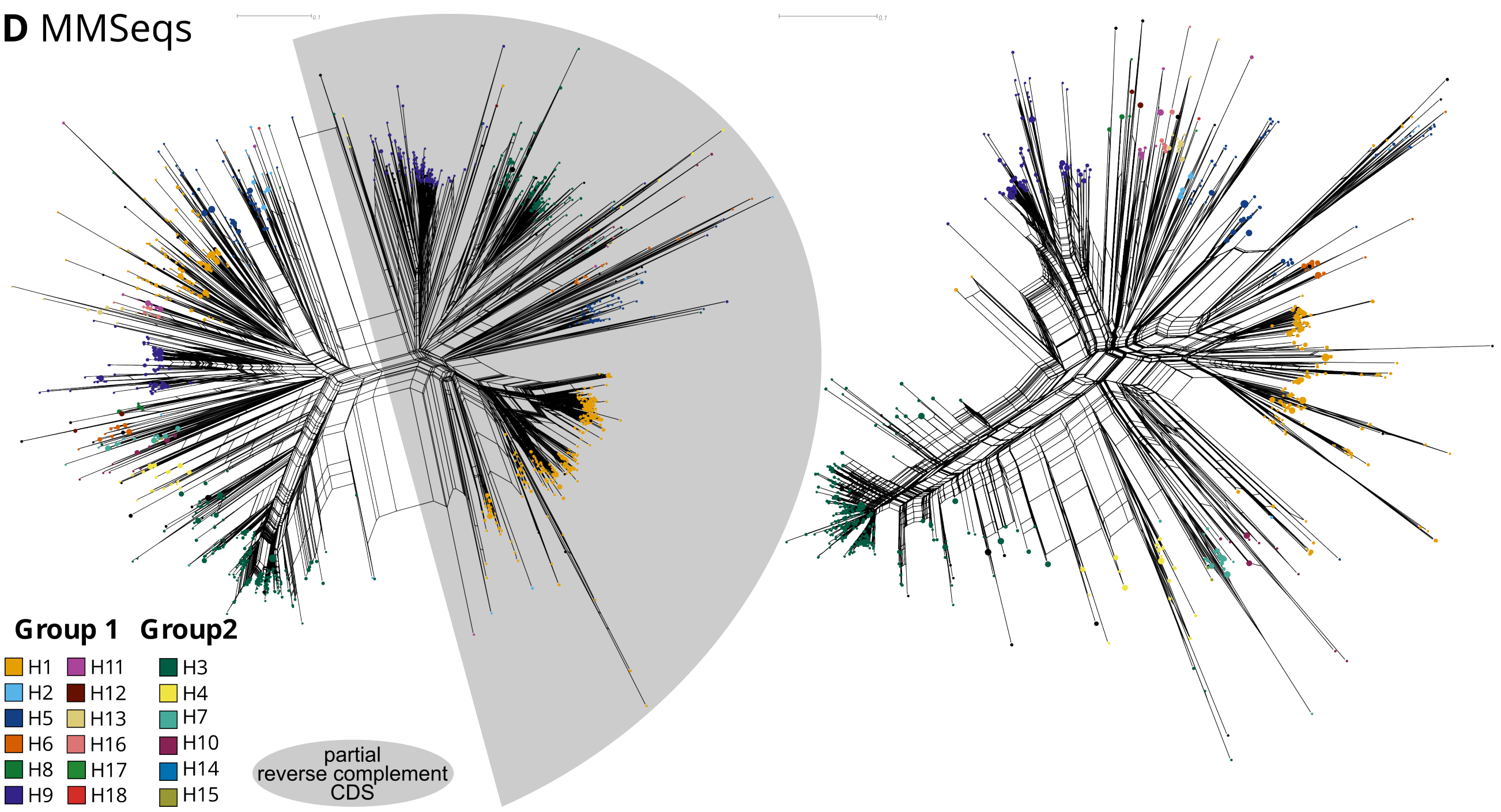

Figure S16. Split graphs of IAV segment 4 (H) representatives of (D) MMSeqs2. Left – all representatives (including partial CDS); Right – only complete genomes. The node size indicates the cluster size. Subtypes (H1–H18) are color-coded. The split graph is reconstructed using SplitsTree based on the MAFFT alignment of the complete genome sequences (108,514 genomes) resulting from the first filtering steps of ViralClust. HDBSCAN did not select any representatives for subtypes H17 and H18. We added these manually to the alignment for split graph reconstruction and marked them as framed circles.

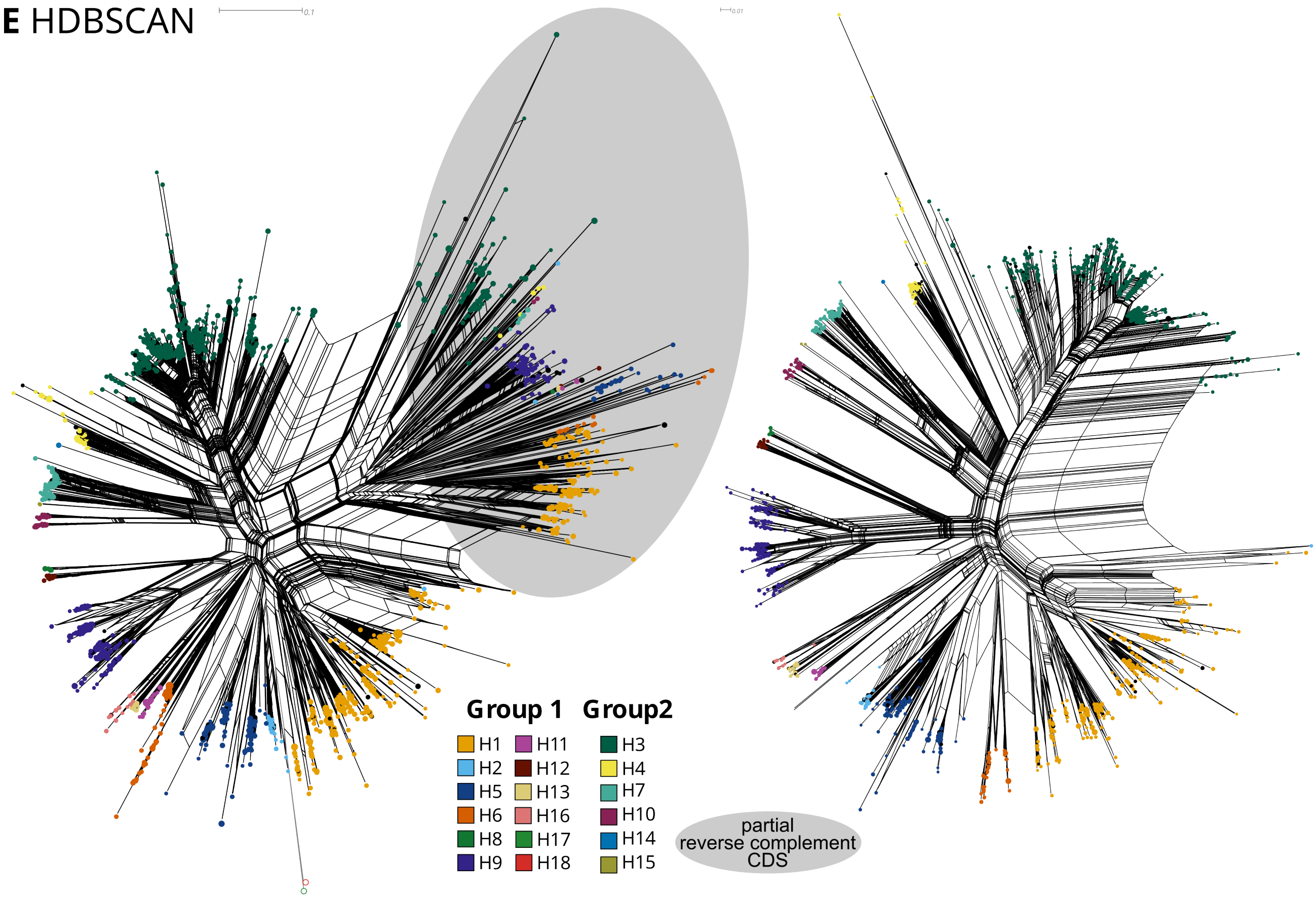

Figure S17. Split graphs of IAV segment 4 (H) representatives of (E) HDBSCAN. Left – all representatives (including partial CDS); Right – only complete genomes. The node size indicates the cluster size. Subtypes (H1–H18) are color-coded. The split graph is reconstructed using `SplitsTree` based on the `MAFFT` alignment of the complete genome sequences (108,514 genomes) resulting from the first filtering steps of `ViralClust`.

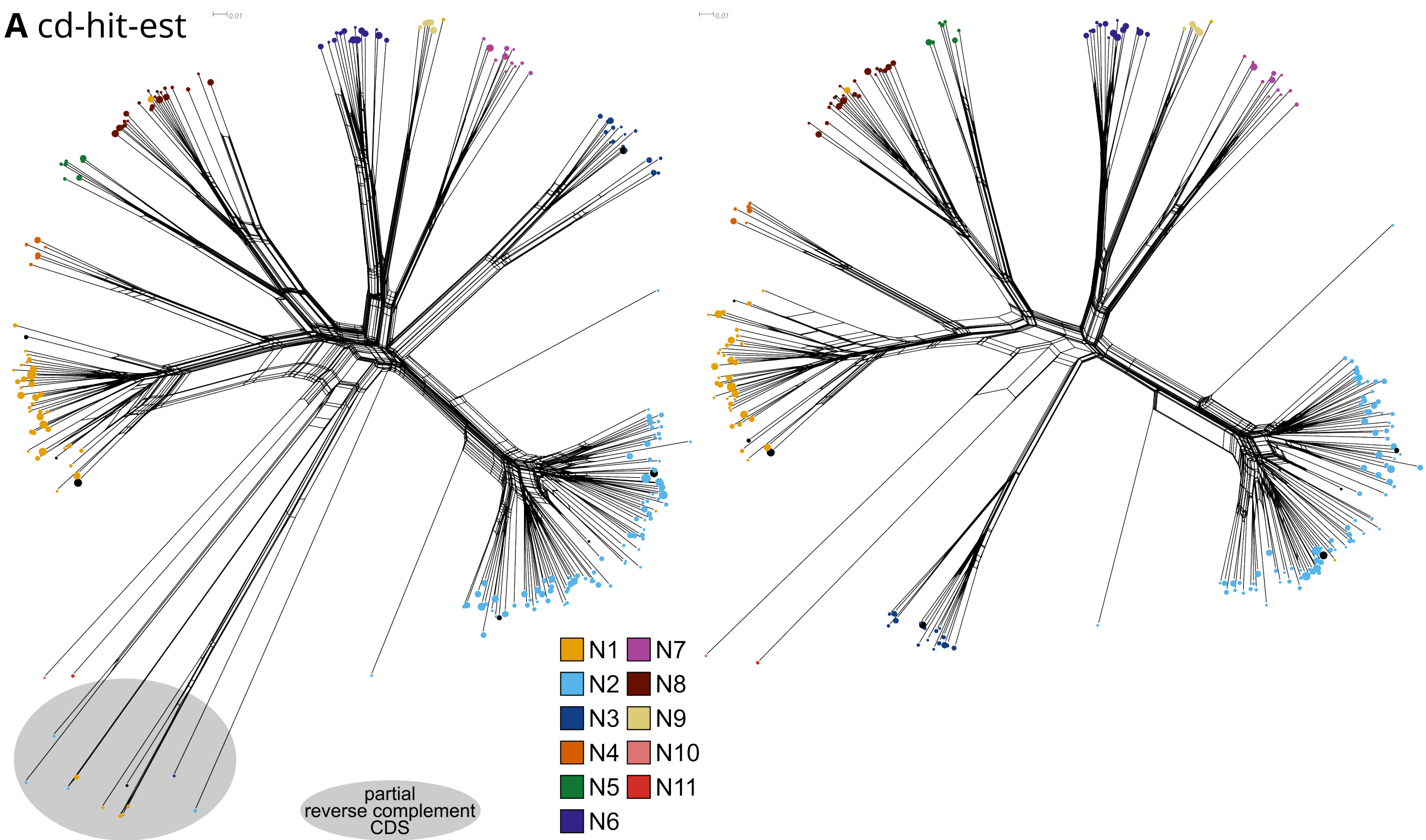

Figure S18. Split graphs of IAV segment 6 (N) representatives of (A) CD-HIT-EST. Left – all representatives (including partial CDS); Right – only complete genomes. The node size indicates the cluster size. Subtypes (N1–N11) are color-coded. The split graph is reconstructed using `SplitsTree` based on the `MAFFT` alignment of the complete genome sequences (108,514 genomes) resulting from the first filtering steps of `ViralClust`.

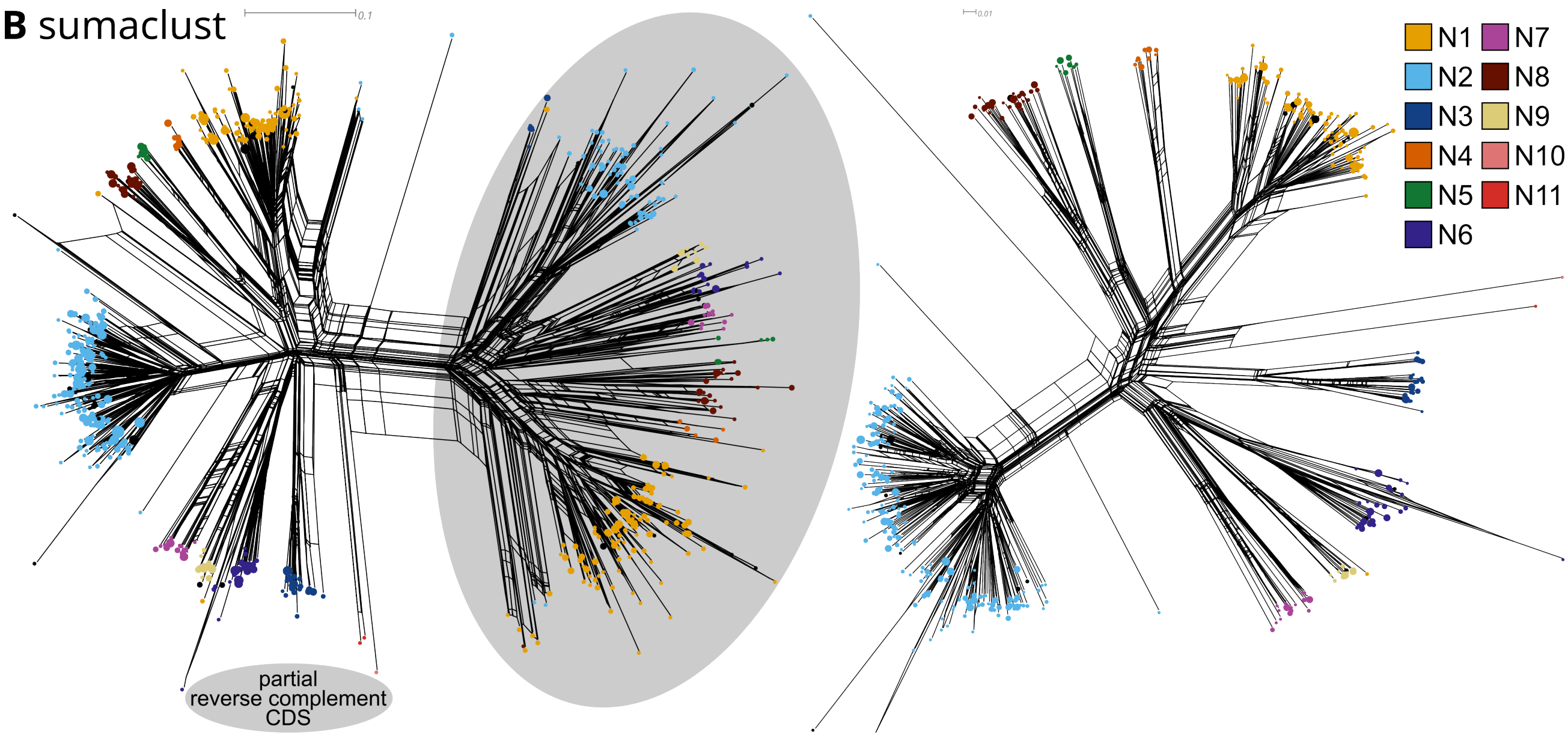

Figure S19. Split graphs of IAV segment 6 (N) representatives of (B) SUMACLUST. Left – all representatives (including partial CDS); Right – only complete genomes. The node size indicates the cluster size. Subtypes (N1–N11) are color-coded. The split graph is reconstructed using `SplitsTree` based on the `MAFFT` alignment of the complete genome sequences (108,514 genomes) resulting from the first filtering steps of `ViralClust`.

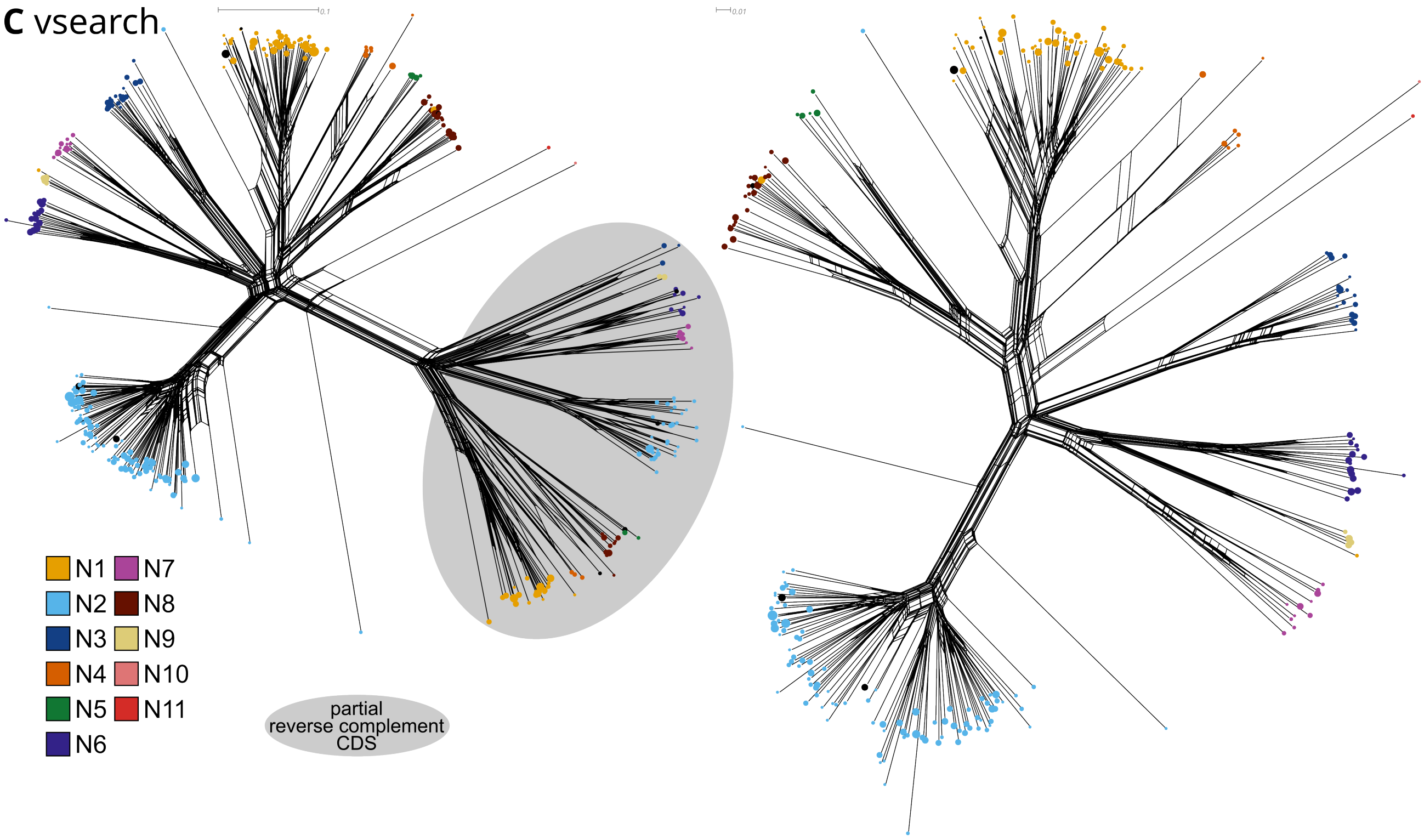

Figure S20. Split graphs of IAV segment 6 (N) representatives of (C) VSEARCH. Left – all representatives (including partial CDS); Right – only complete genomes. The node size indicates the cluster size. Subtypes (N1–N11) are color-coded. The split graph is reconstructed using `SplitsTree` based on the `MAFFT` alignment of the complete genome sequences (108,514 genomes) resulting from the first filtering steps of `ViralClust`.

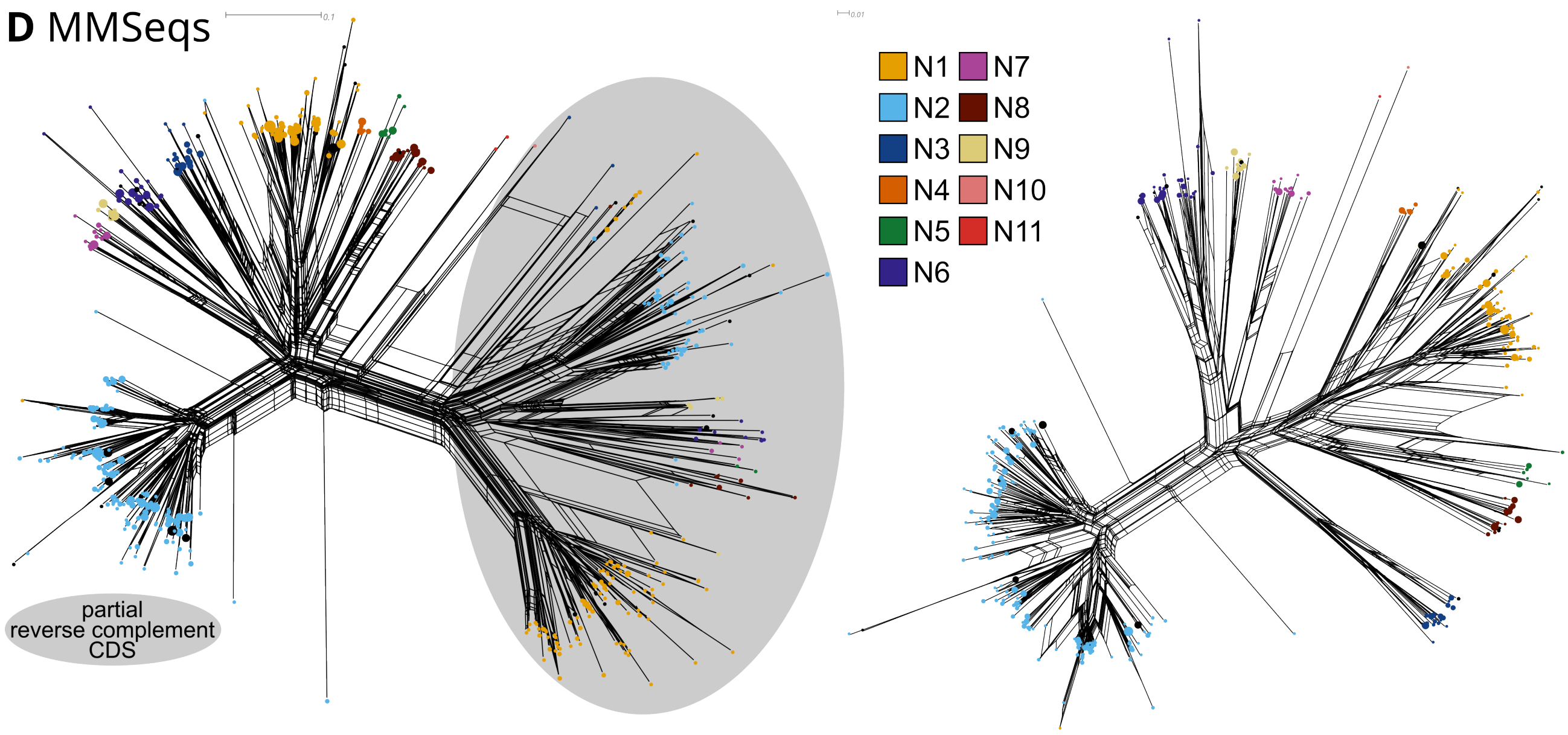

Figure S21. Split graphs of IAV segment 6 (N) representatives of (D) `MMSeqs2`. Left – all representatives (including partial CDS); Right – only complete genomes. The node size indicates the cluster size. Subtypes (N1–N11) are color-coded. The split graph is reconstructed using `SplitsTree` based on the `MAFFT` alignment of the complete genome sequences (108,514 genomes) resulting from the first filtering steps of `ViralClust`.

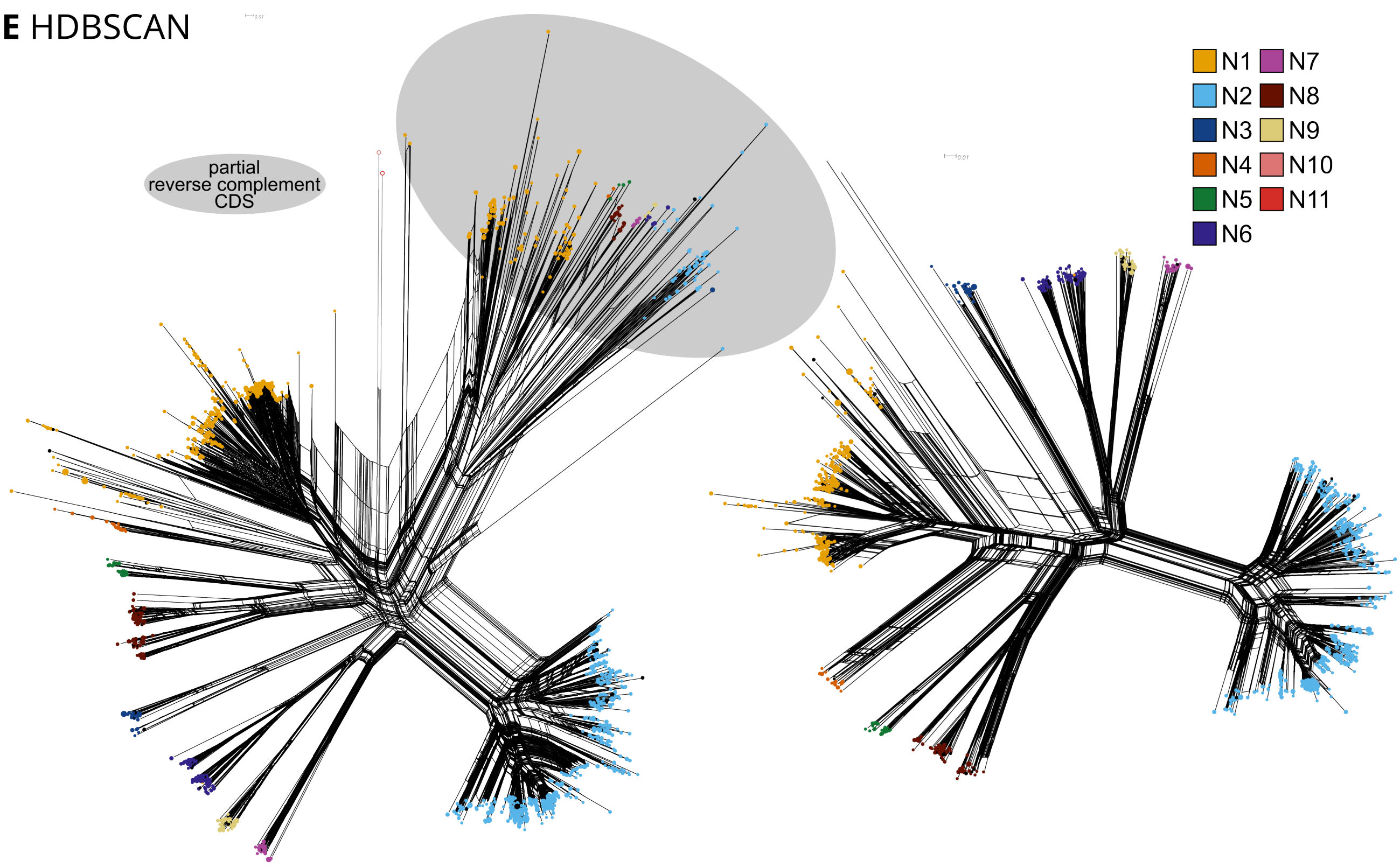

Figure S22. Split graphs of IAV segment 6 (N) representatives of (E) `HDBSCAN`. Left – all representatives (including partial CDS); Right – only complete genomes. The node size indicates the cluster size. Subtypes (N1–N11) are color-coded. The split graph is reconstructed using `SplitsTree` based on the `MAFFT` alignment of the complete genome sequences (108,514 genomes) resulting from the first filtering steps of `ViralClust`. `HDBSCAN` did not select any representatives for subtypes N10 and N11. We added these manually to the alignment for split graph reconstruction and marked them as framed circles.

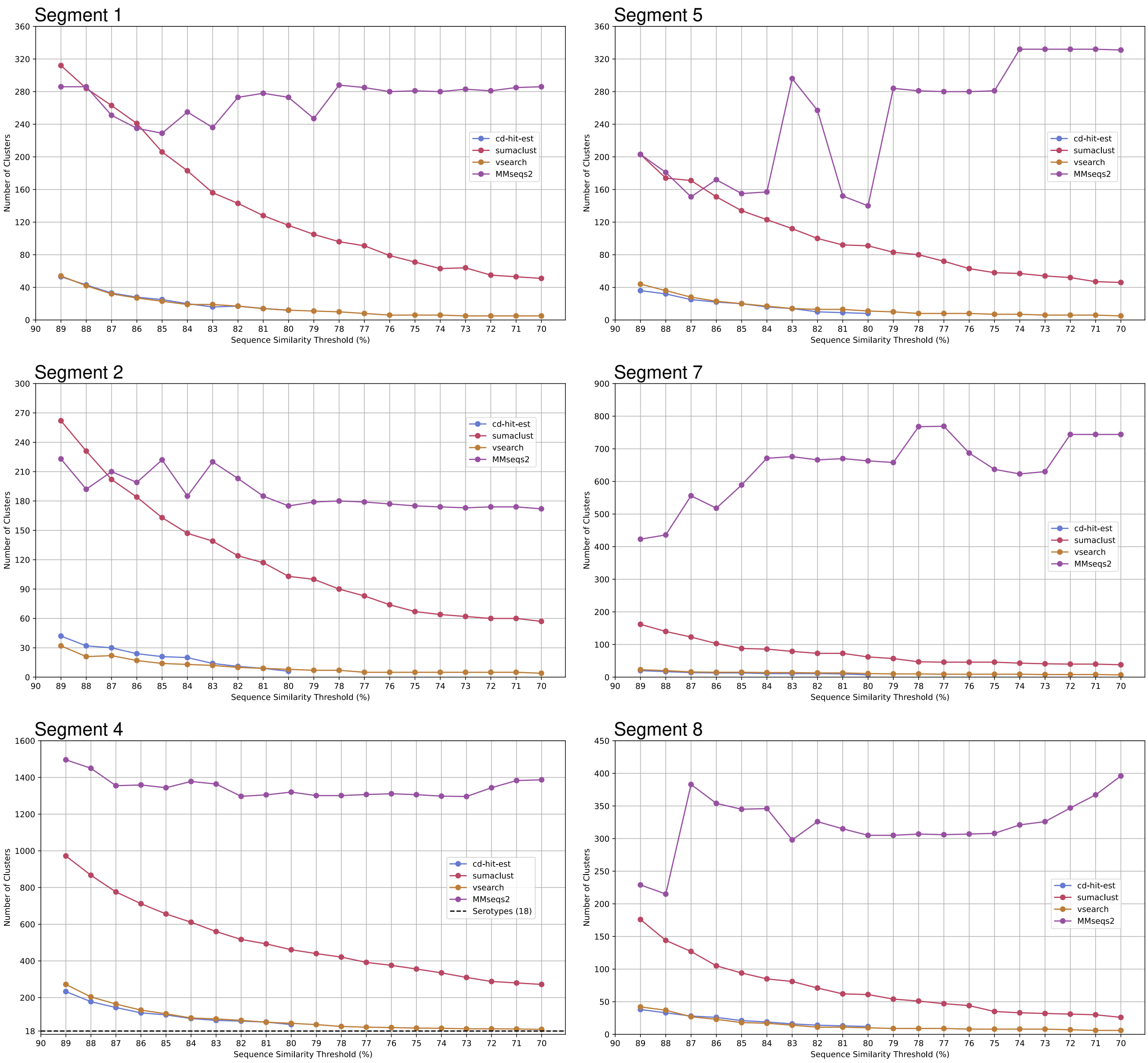

Figure S23. Number of clusters of IAV segments 1, 2, 4, 5, 7, and 8 with sequence similarity thresholds from 70 - 90 %. CD-HIT-EST only allows a minimum of 80 %. Only clusters with with a size greater than one are considered.

Table S7. Overview of IAV clustering with 70–90 % sequence similarity. Seg – segment; Algo – clustering algorithm; cd – CD-HIT-EST; sum – SUMACLUST; vse – VSEARCH; mmseq – MMSeqs2; Number of genomes after clustering – only considering clusters with a size greater than one.

Seg	Algo	Sequence similarity threshold (%)																				
		90	89	88	87	86	85	84	83	82	81	80	79	78	77	76	75	74	73	72	71	70
1	cd	66	53	43	33	28	25	20	16	17	14	12										
	suma	390	312	284	263	241	206	183	156	143	128	116	105	96	91	79	71	63	64	55	53	51
	vse	90	54	42	32	27	23	19	19	17	14	12	11	10	8	6	6	6	5	5	5	5
	mmseq	280	286	286	251	235	229	255	236	273	278	273	247	288	285	280	281	280	283	281	285	286
2	cd	56	42	32	30	24	21	20	14	11	9	6										
	suma	338	262	231	202	184	163	147	139	124	117	103	100	90	83	74	67	64	62	60	60	57
	vse	73	32	21	22	17	14	13	12	10	9	8	7	7	5	5	5	5	5	5	5	4
	mmseq	216	223	192	210	199	222	185	220	203	185	175	179	180	179	177	175	174	173	174	174	172
3	cd	63	46	35	31	24	20	18	17	19	15	13										
	suma	305	263	233	200	184	166	153	145	138	119	111	102	94	83	78	72	69	66	60	57	51
	vse	75	38	32	25	21	19	17	16	13	13	11	10	8	7	7	7	7	7	7	7	7
	mmseq	265	193	188	213	208	195	189	551	274	263	298	294	290	308	314	299	325	382	384	384	383
4 (H)	cd	300	233	178	146	117	106	86	76	72	67	53										
	suma	1,237	972	867	776	712	656	611	560	517	493	461	440	421	392	376	356	335	310	288	280	272
	vse	464	272	204	165	132	112	89	84	76	66	60	53	43	39	37	34	33	30	30	29	27
	mmseq	1,574	1,496	1,450	1,355	1,359	1,344	1,378	1,364	1,297	1,305	1,320	1,301	1,301	1,307	1,311	1,306	1,298	1,296	1,344	1,383	1,387
5	cd	46	36	32	25	22	20	16	14	10	9	8										
	suma	262	203	174	171	151	134	123	112	100	92	91	83	80	72	63	58	57	54	52	47	46
	vse	76	44	36	28	23	20	17	14	13	13	11	10	8	8	8	7	7	6	6	6	5
	mmseq	179	203	181	151	172	155	157	296	257	152	140	284	281	280	280	281	332	332	332	332	331
6 (N)	cd	228	190	143	121	94	78	63	63	50	47	43										
	suma	773	618	557	507	457	404	378	357	324	307	298	278	268	244	237	223	206	191	179	173	168
	vse	329	215	163	131	104	91	78	69	64	56	48	45	40	40	39	40	32	30	28	24	23
	mmseq	640	562	541	518	509	484	467	462	530	471	484	554	547	525	533	530	627	636	684	659	704
7	cd	25	20	17	14	13	13	11	11	11	10	7										
	suma	185	162	140	123	103	88	86	79	73	73	62	57	47	46	46	46	43	41	40	40	38
	vse	38	23	20	16	15	15	14	14	13	13	11	10	10	9	9	9	9	8	8	8	7
	mmseq	981	423	436	556	518	589	671	676	666	670	663	658	768	769	687	637	623	630	744	744	744
8	cd	51	38	33	28	26	21	19	16	14	13	12										
	suma	185	176	144	127	105	94	85	81	71	62	61	54	51	47	44	35	33	32	31	30	26
	vse	38	42	37	27	23	18	17	14	11	11	10	9	9	9	8	8	8	8	7	6	6
	mmseq	802	229	215	383	354	345	346	298	326	315	305	305	307	306	307	308	321	326	347	367	396

Table S8. Overview of IAV clustering with HDBSCAN and k-mer length 1–7. Seg – segment; Number of genomes after clustering – only considering clusters with a size greater than one.

Seg	k-mer						
	7	6	5	4	3	2	1
1	1,412	1,426	1,130	1,158	687	1,267	3,883
2	1,311	1,373	1,286	1,034	720	1,346	3,789
3	1,371	1,298	1,263	1,148	798	1,168	3,968
4 (H)	2,307	2,333	2,298	1,854	1,560	1,466	7,103
5	1,218	1,082	1,006	739	563	3,157	2
6 (N)	1,622	1,351	1,369	1,167	929	1,528	4,880
7	981	948	854	744	354	588	3,084
8	802	590	548	483	295	923	2

Monkeypox virus

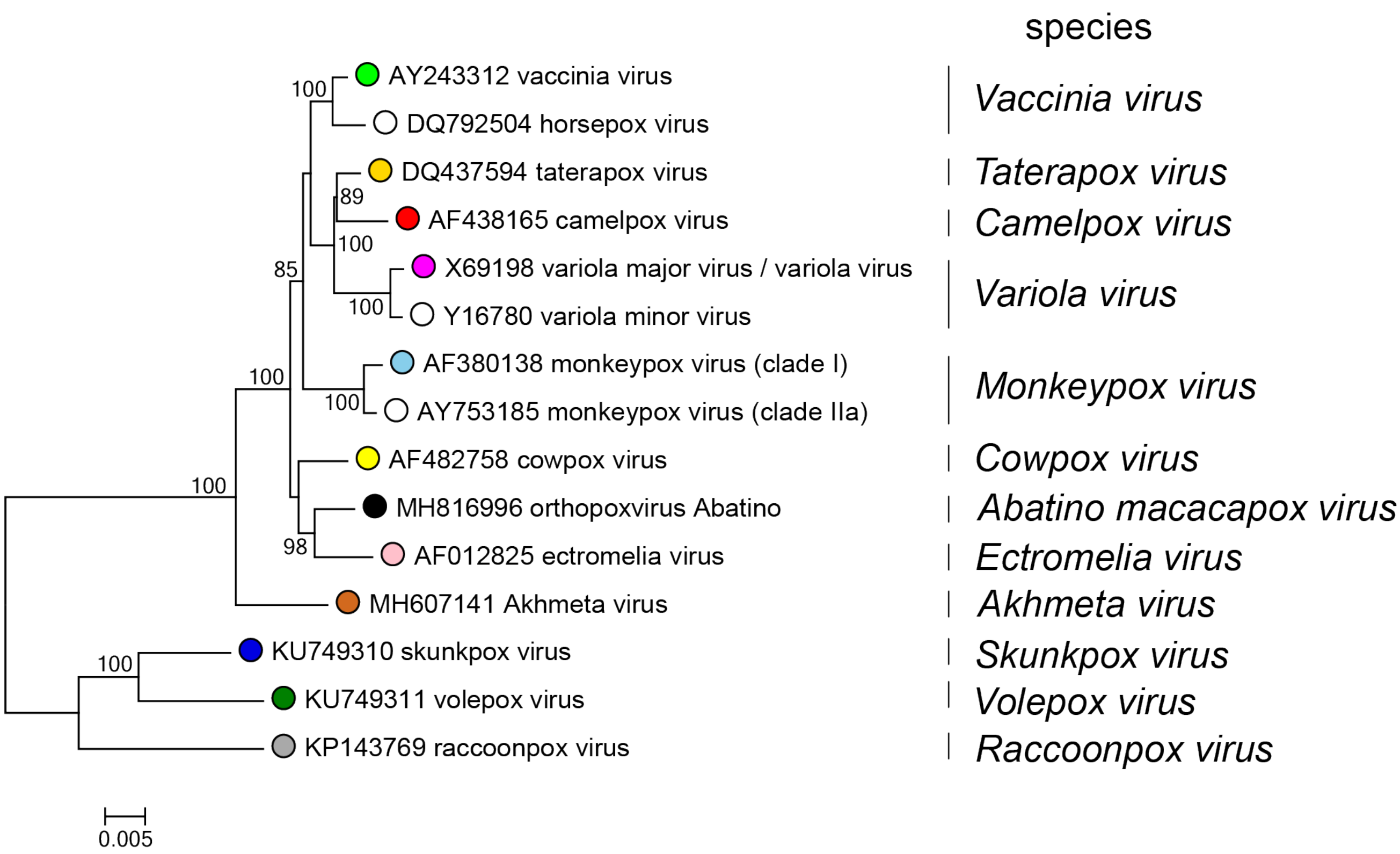

Figure S24. Phylogenetic tree of *Orthopoxvirus* (ICTV) including *Monkeypox virus*.

Table S9. Number of sequences of the *Monkeypox virus* dataset passing the filtering of non-ACGU characters (`--max_ambiguous`) with several parameter settings. The default setting is highlighted in bold.

non-ACGU character allowed	#Seq
30 %	1,077
25 %	1,077
20 %	1,077
15 %	1,077
**10 %**	1,077
5 %	1,077
0 %	410
